# Supplementary figures and images for: FBXO24 modulates mRNA alternative splicing and MIWI degradation and is required for normal sperm formation and male fertility (part 1 of 2)
Source: eLife. 2024 Mar 12;12:RP91666. doi: 10.7554/eLife.91666 (PMC10932545; doi:10.7554/eLife.91666)

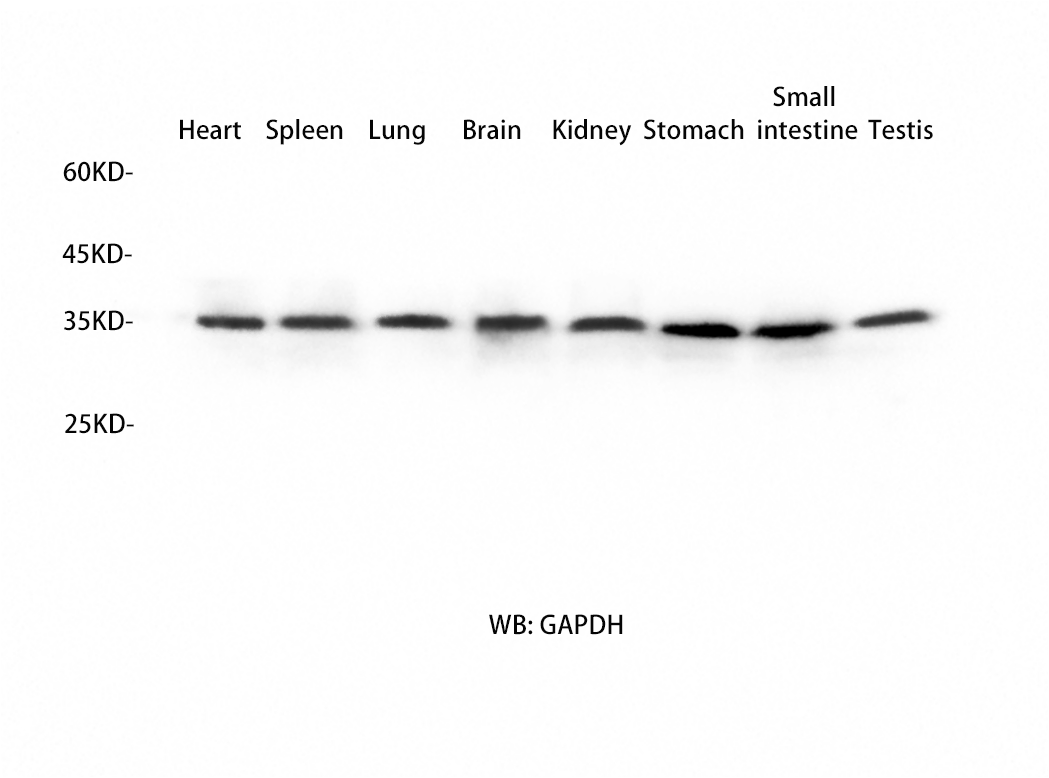

Supplement: Figure 1—source data 1. [file elife-91666-fig1-data1.zip › Figure 1-source data 1/GAPDH-labelled.tif]

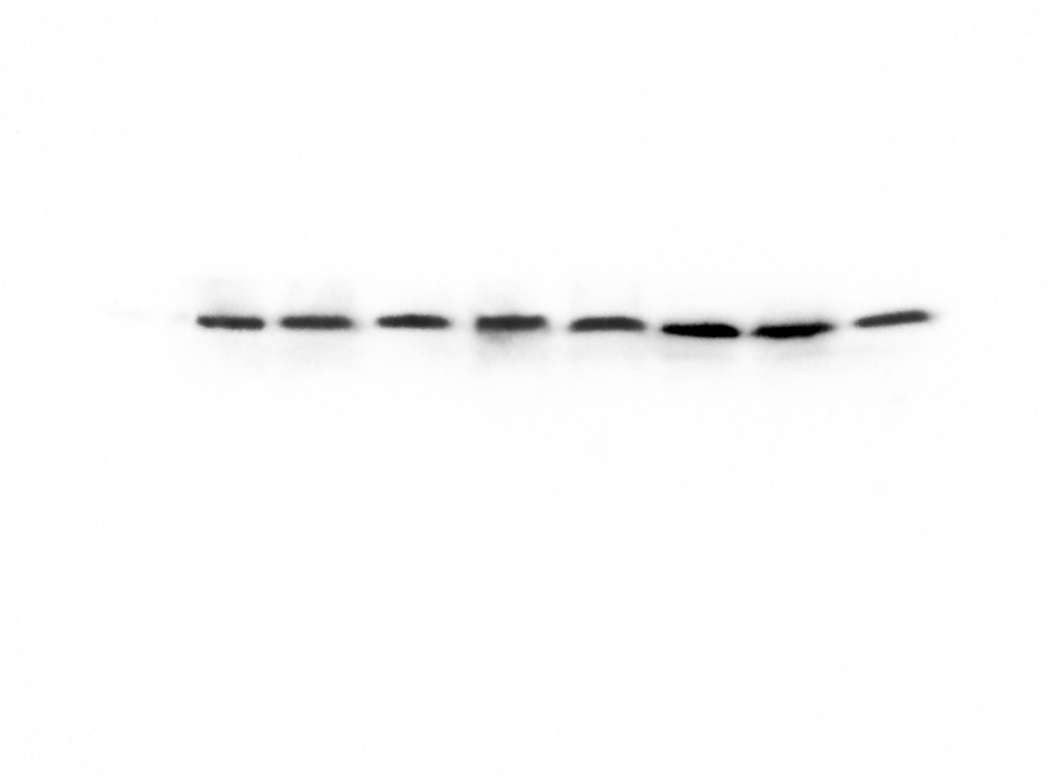

Supplement: Figure 1—source data 1. [file elife-91666-fig1-data1.zip › Figure 1-source data 1/GAPDH-unedited.tif]

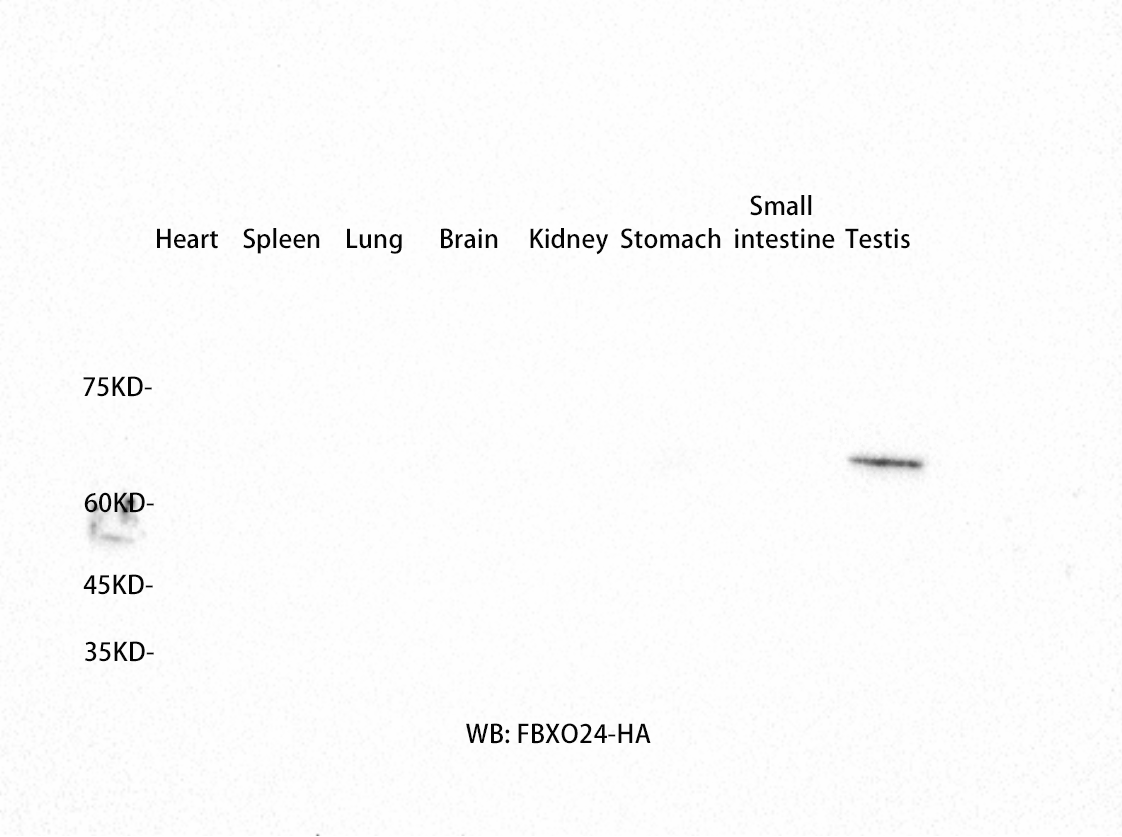

Supplement: Figure 1—source data 1. [file elife-91666-fig1-data1.zip › Figure 1-source data 1/HA-labelled.tif]

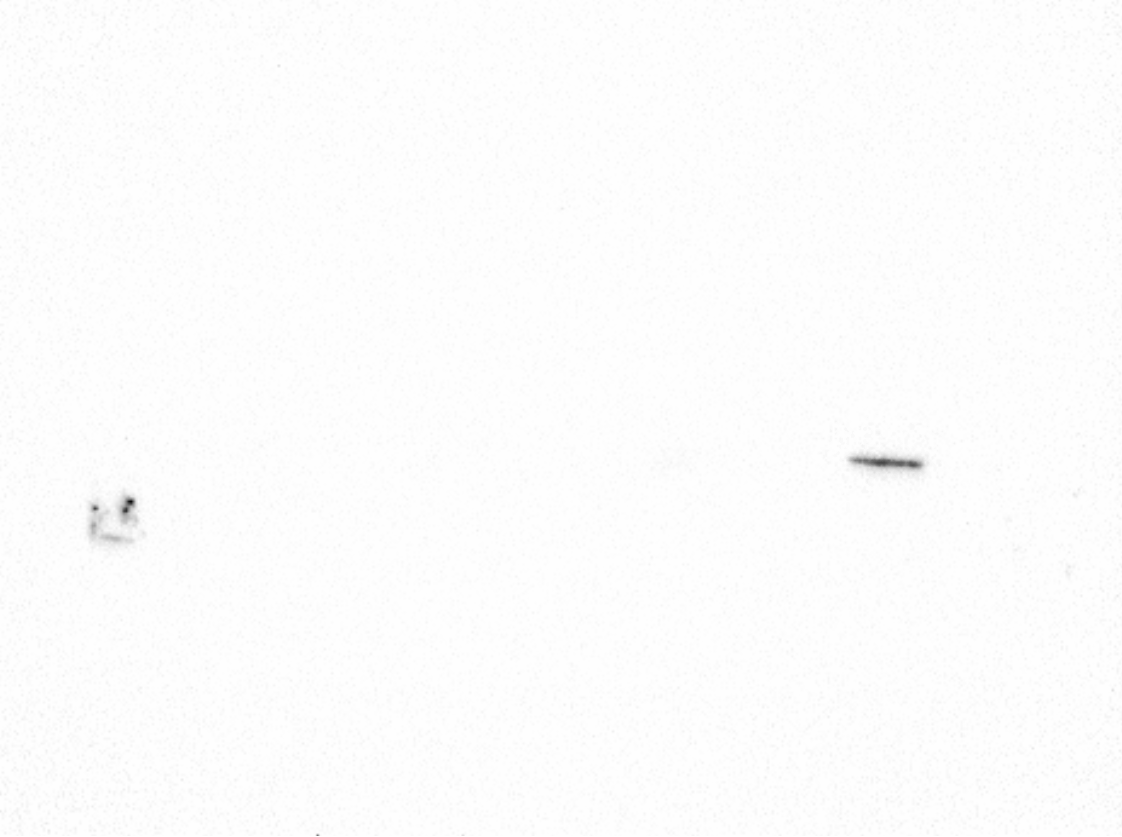

Supplement: Figure 1—source data 1. [file elife-91666-fig1-data1.zip › Figure 1-source data 1/HA-unedited.tif]

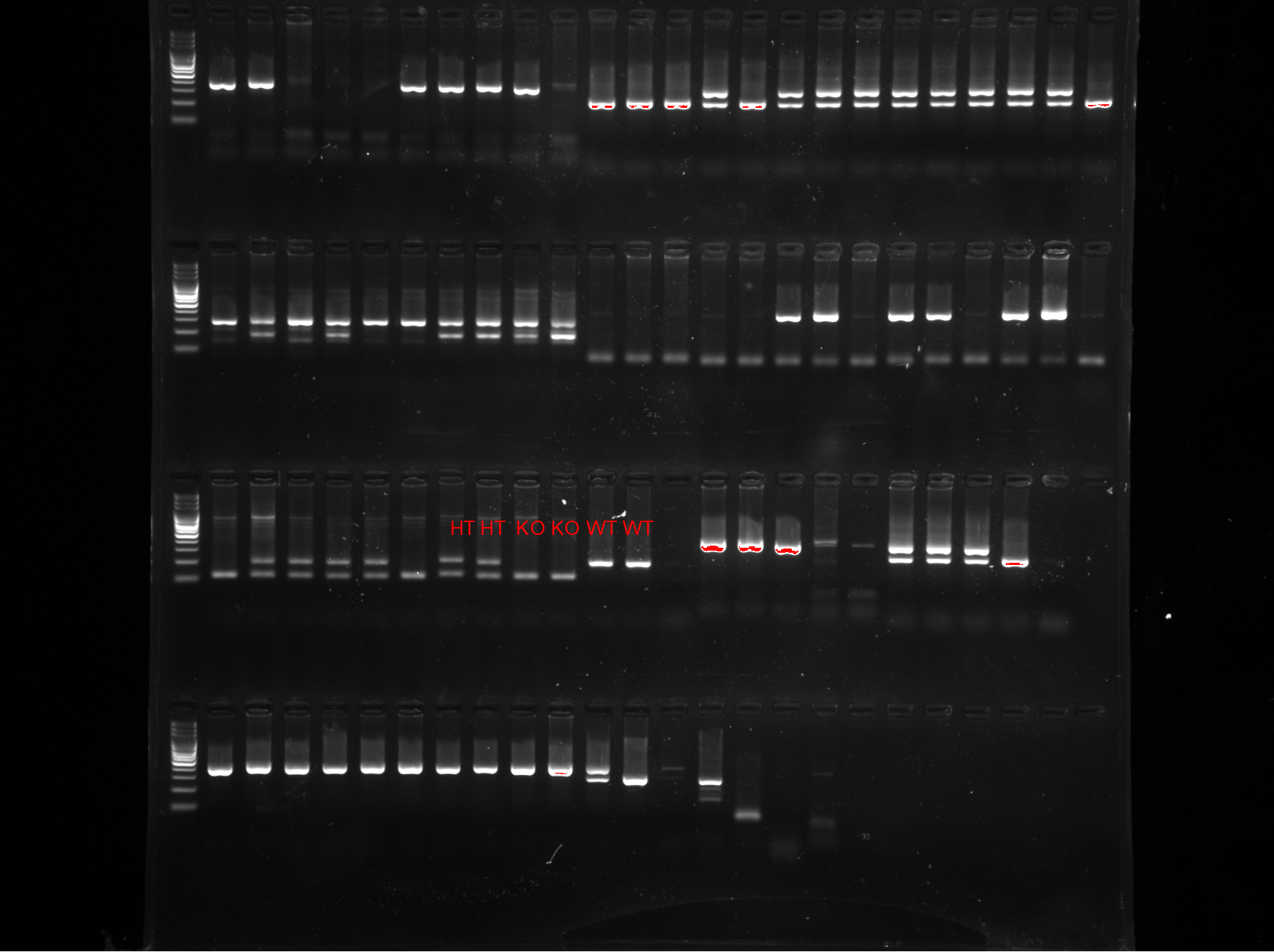

Supplement: Figure 2—figure supplement 1—source data 1. [file elife-91666-fig2-figsupp1-data1.zip › Figure 2-Figure supplement 2-source data 1/genotype-labelled.tif]

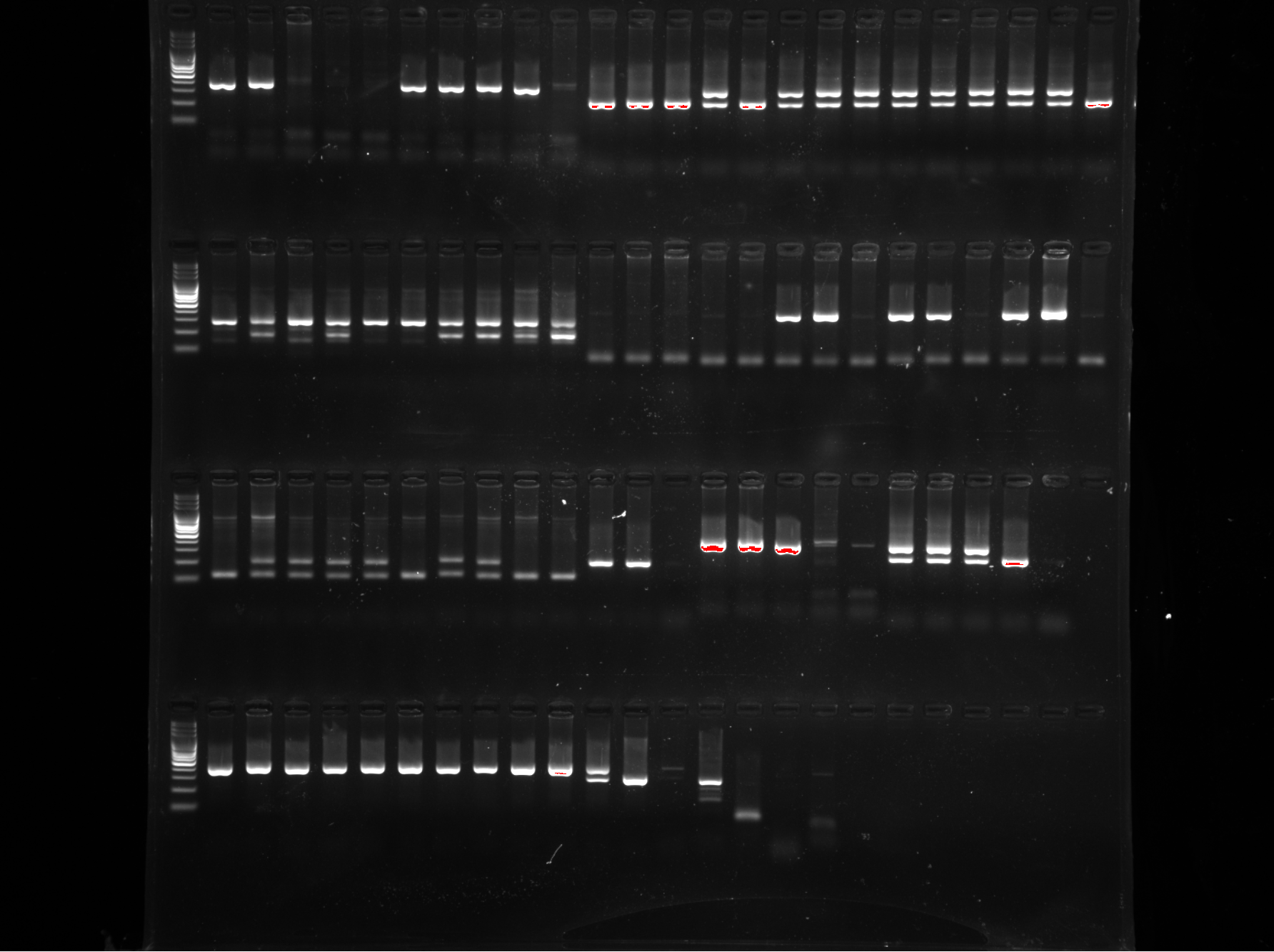

Supplement: Figure 2—figure supplement 1—source data 1. [file elife-91666-fig2-figsupp1-data1.zip › Figure 2-Figure supplement 2-source data 1/genotype-unedited.tif]

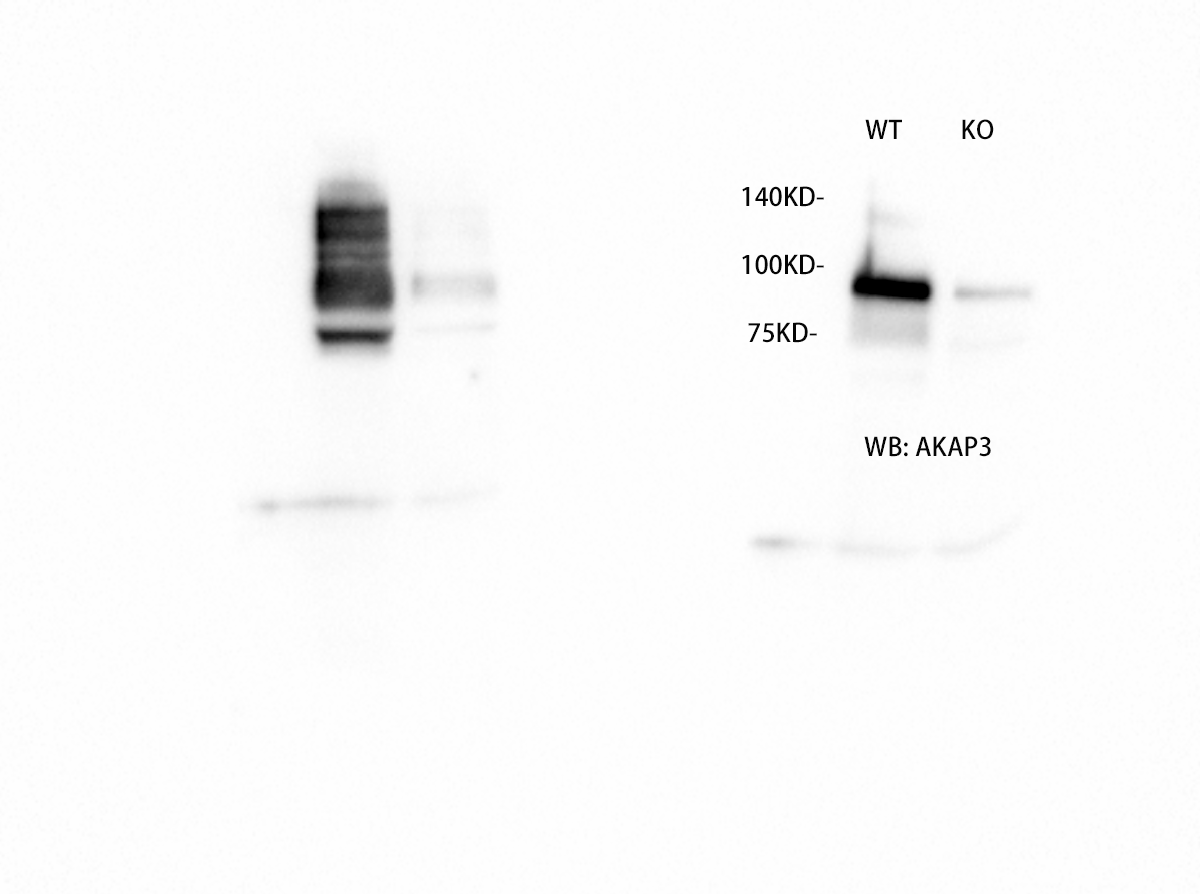

Supplement: Figure 3—source data 1. [file elife-91666-fig3-data1.zip › Figure 3-source data 1/AKAP3-labelled.tif]

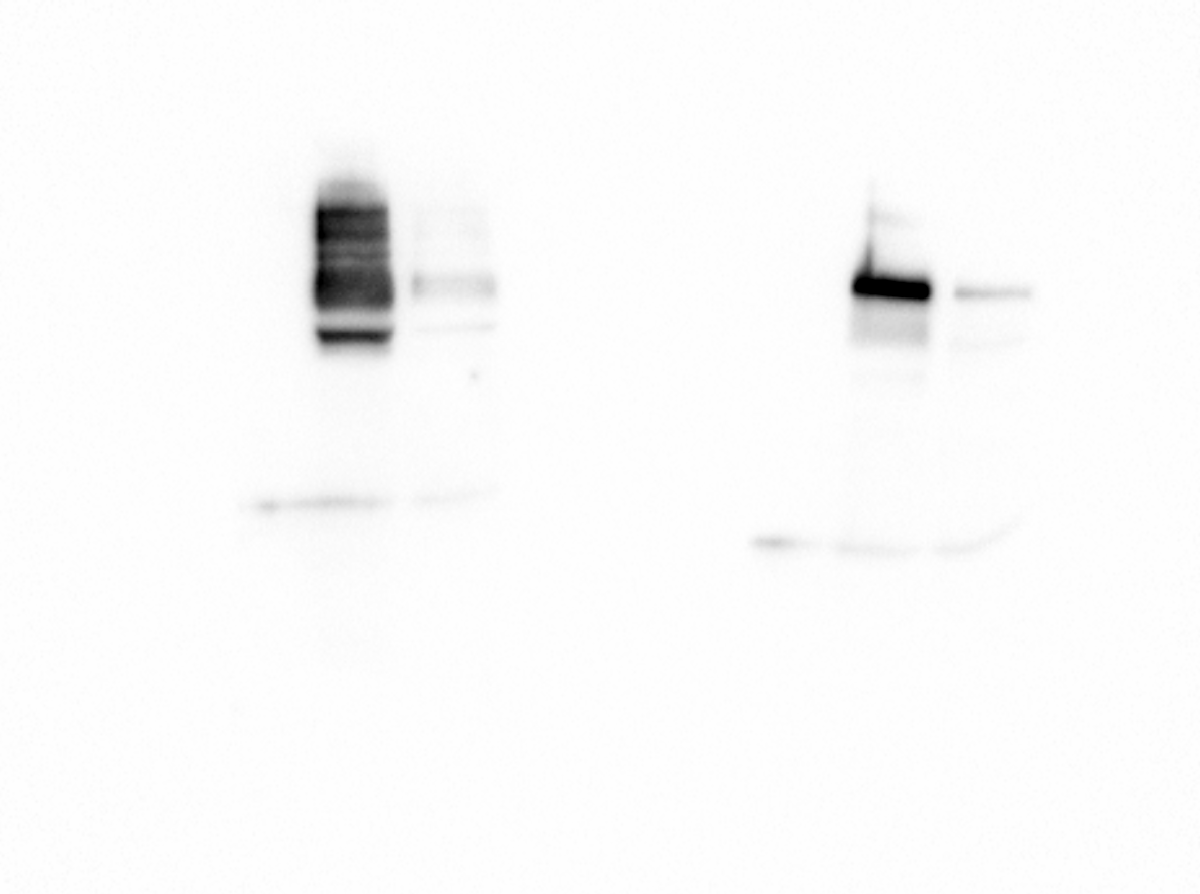

Supplement: Figure 3—source data 1. [file elife-91666-fig3-data1.zip › Figure 3-source data 1/AKAP3-unedited.tif]

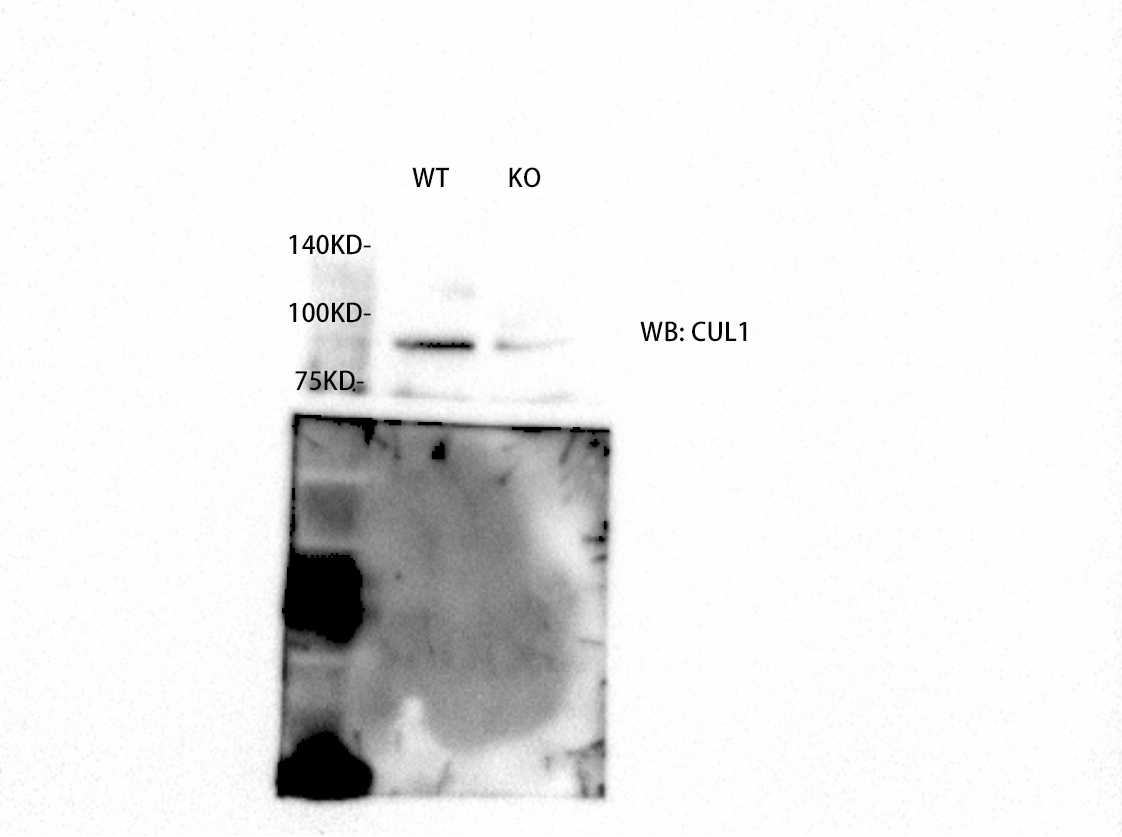

Supplement: Figure 3—source data 1. [file elife-91666-fig3-data1.zip › Figure 3-source data 1/CUL1-labelled.tif]

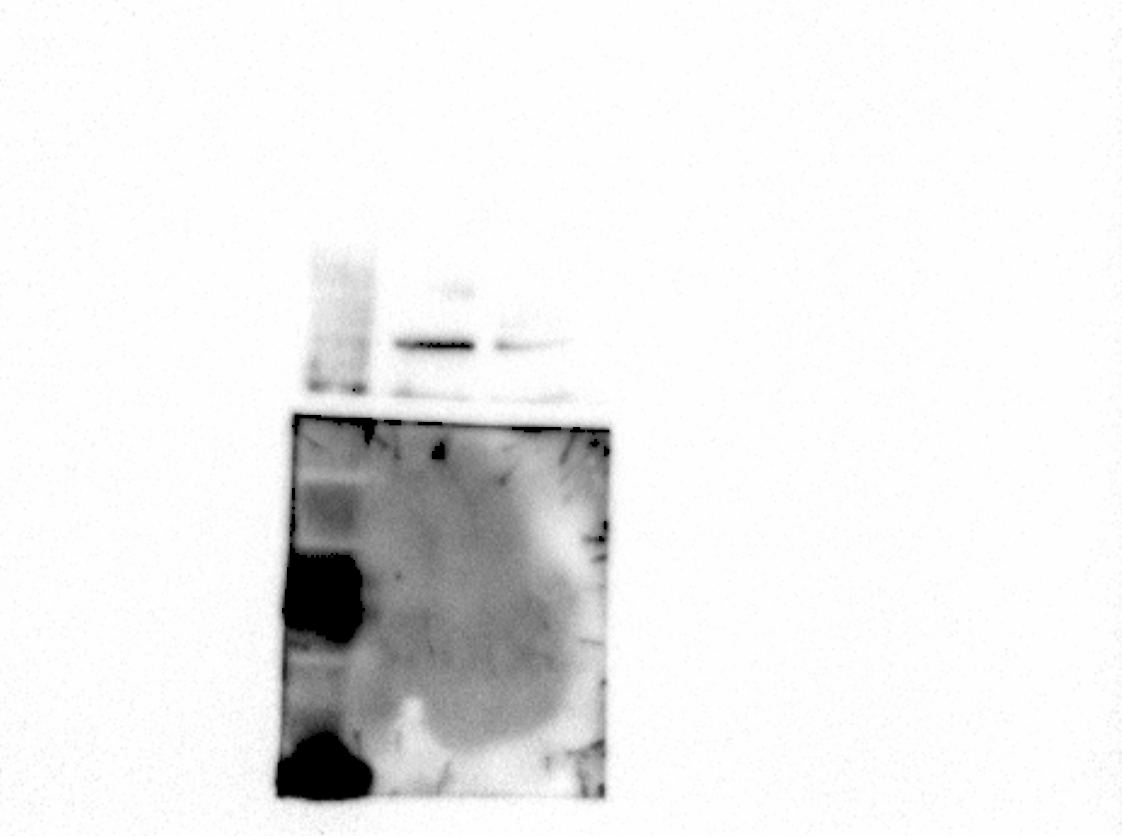

Supplement: Figure 3—source data 1. [file elife-91666-fig3-data1.zip › Figure 3-source data 1/CUL1-unedited.tif]

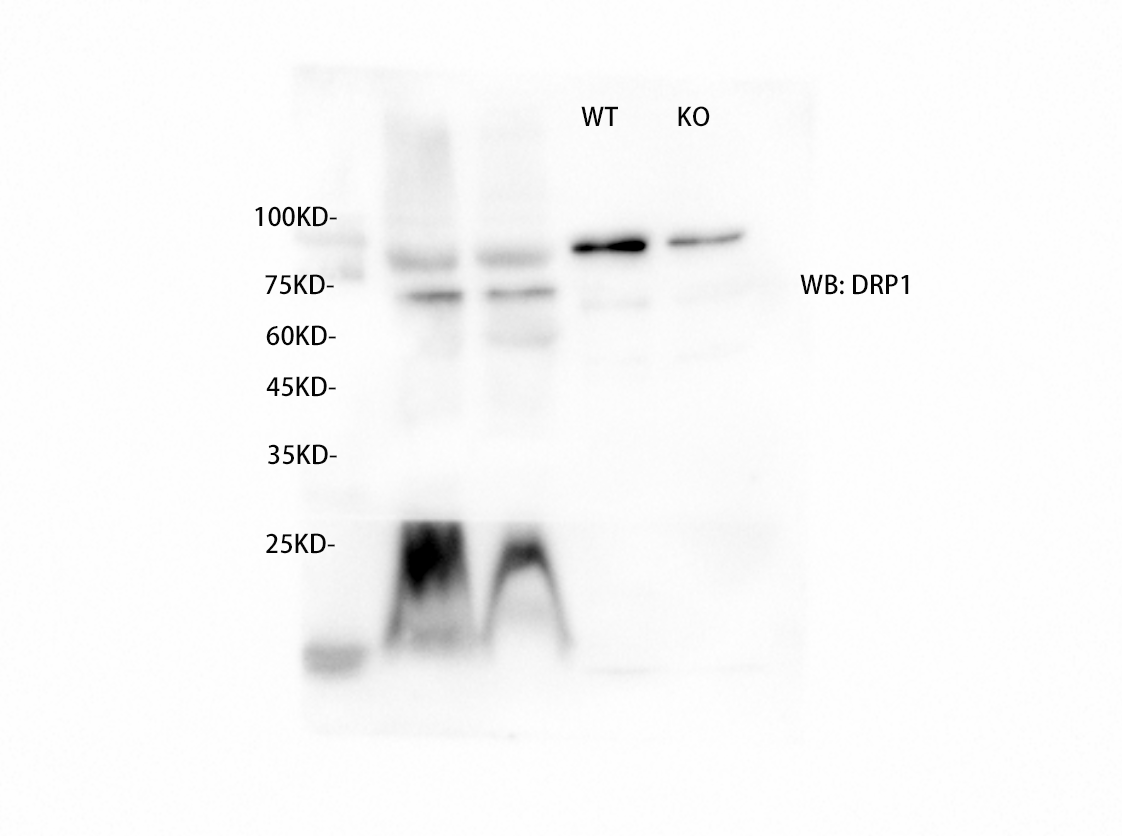

Supplement: Figure 3—source data 1. [file elife-91666-fig3-data1.zip › Figure 3-source data 1/DRP1-labelled.tif]

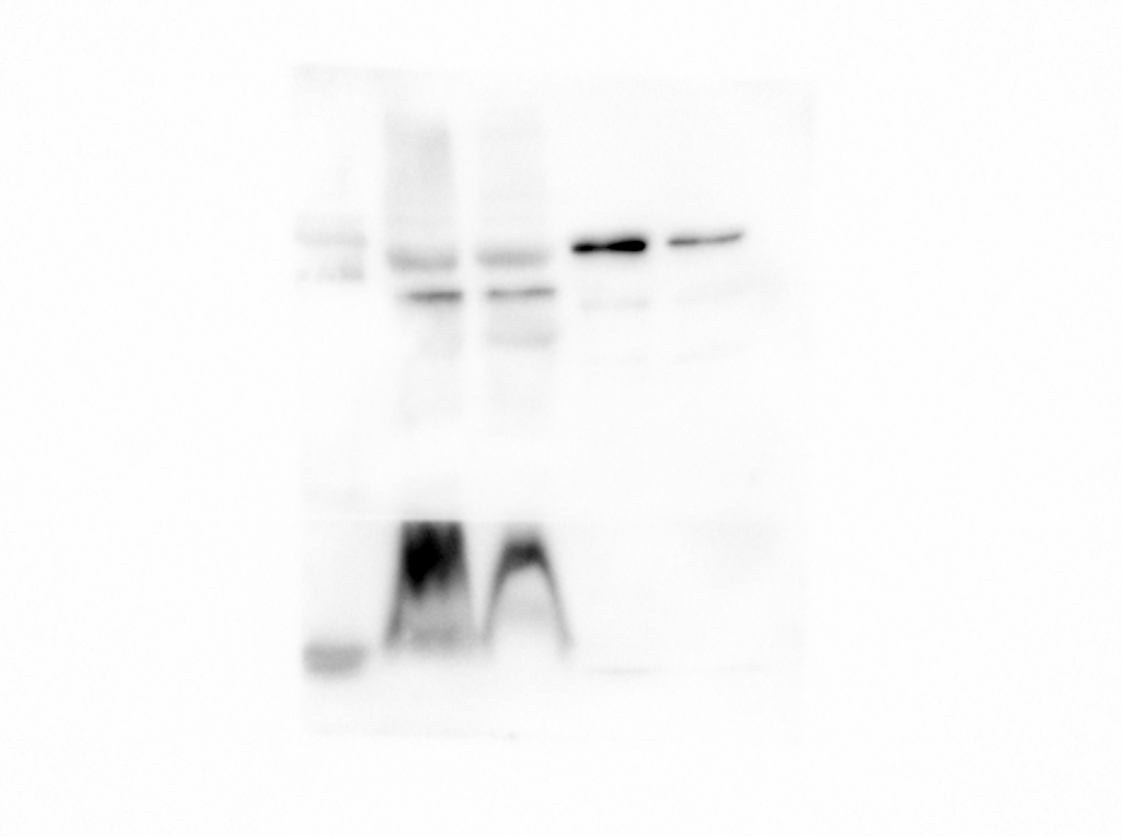

Supplement: Figure 3—source data 1. [file elife-91666-fig3-data1.zip › Figure 3-source data 1/DRP1-unedited.tif]

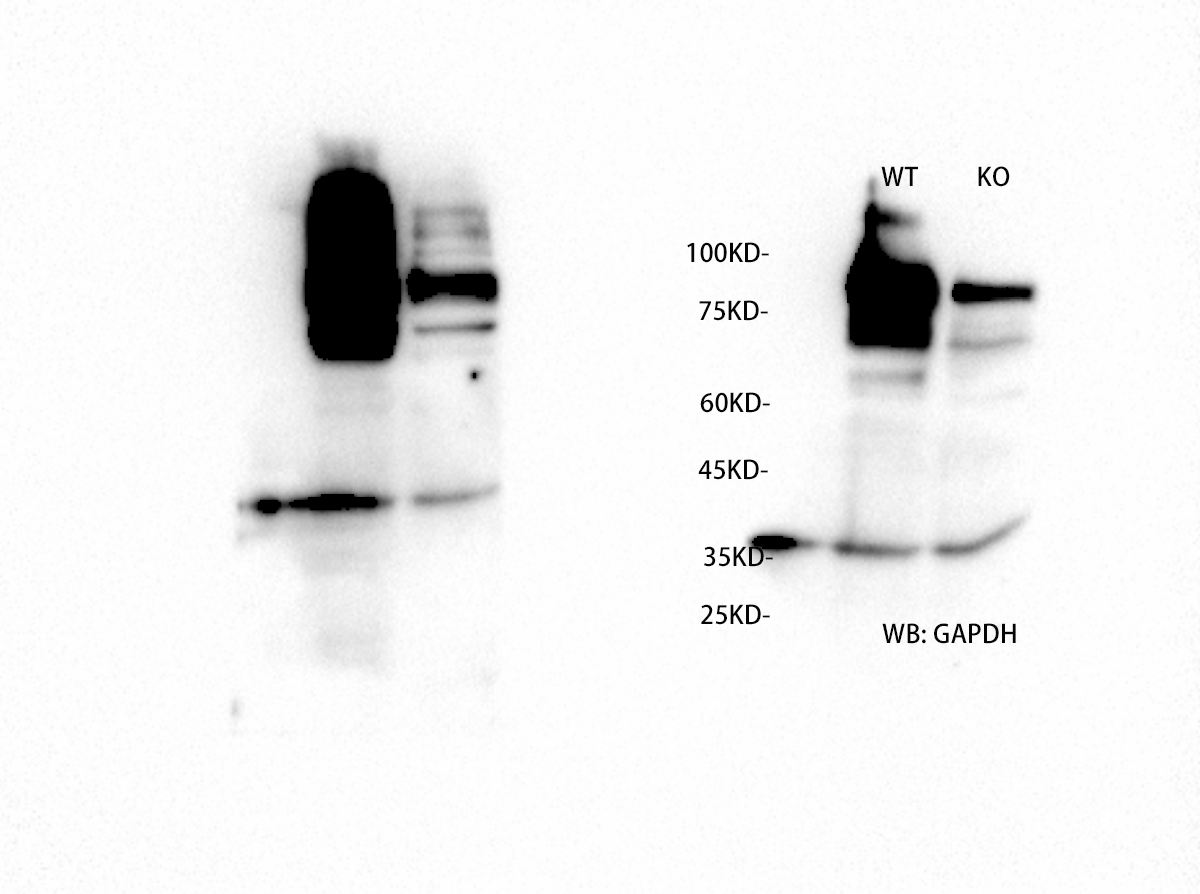

Supplement: Figure 3—source data 1. [file elife-91666-fig3-data1.zip › Figure 3-source data 1/GAPDH(2)-labelled.tif]

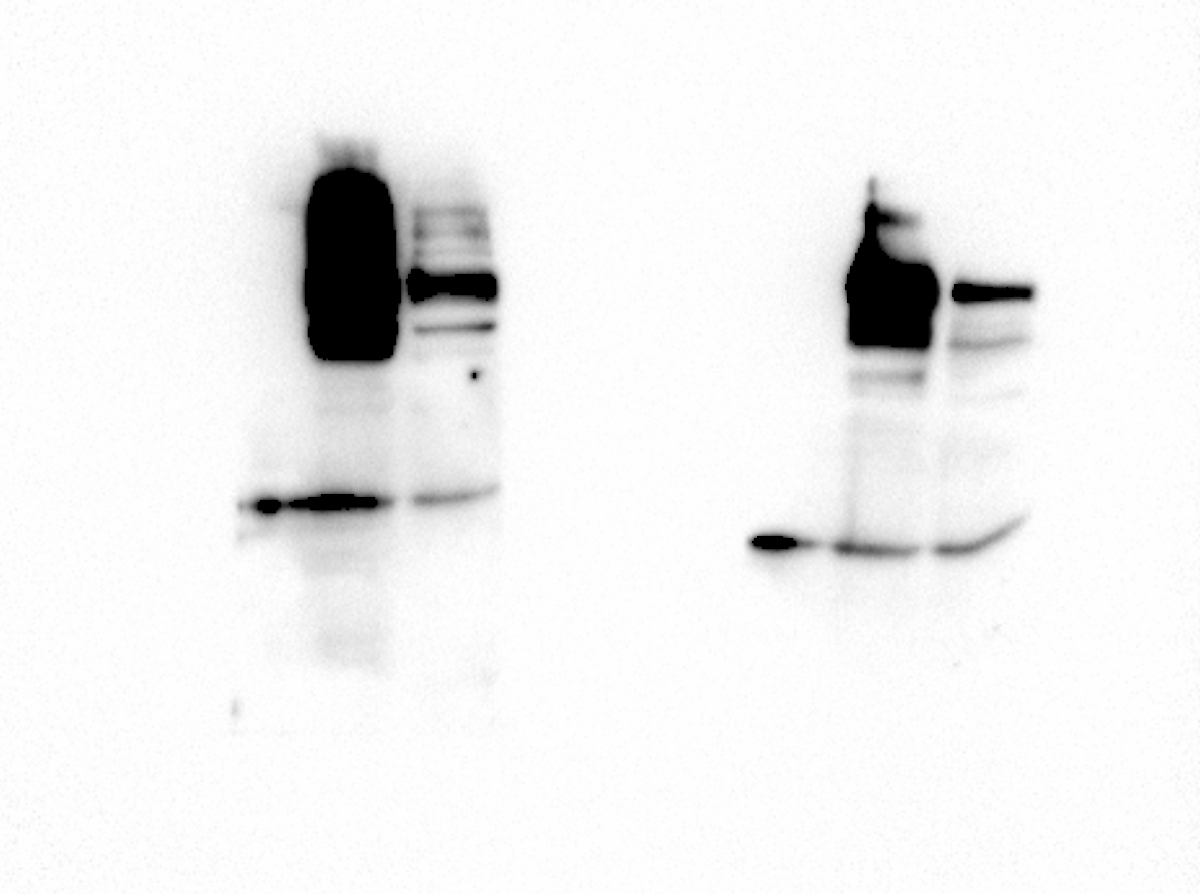

Supplement: Figure 3—source data 1. [file elife-91666-fig3-data1.zip › Figure 3-source data 1/GAPDH(2)-unedited.tif]

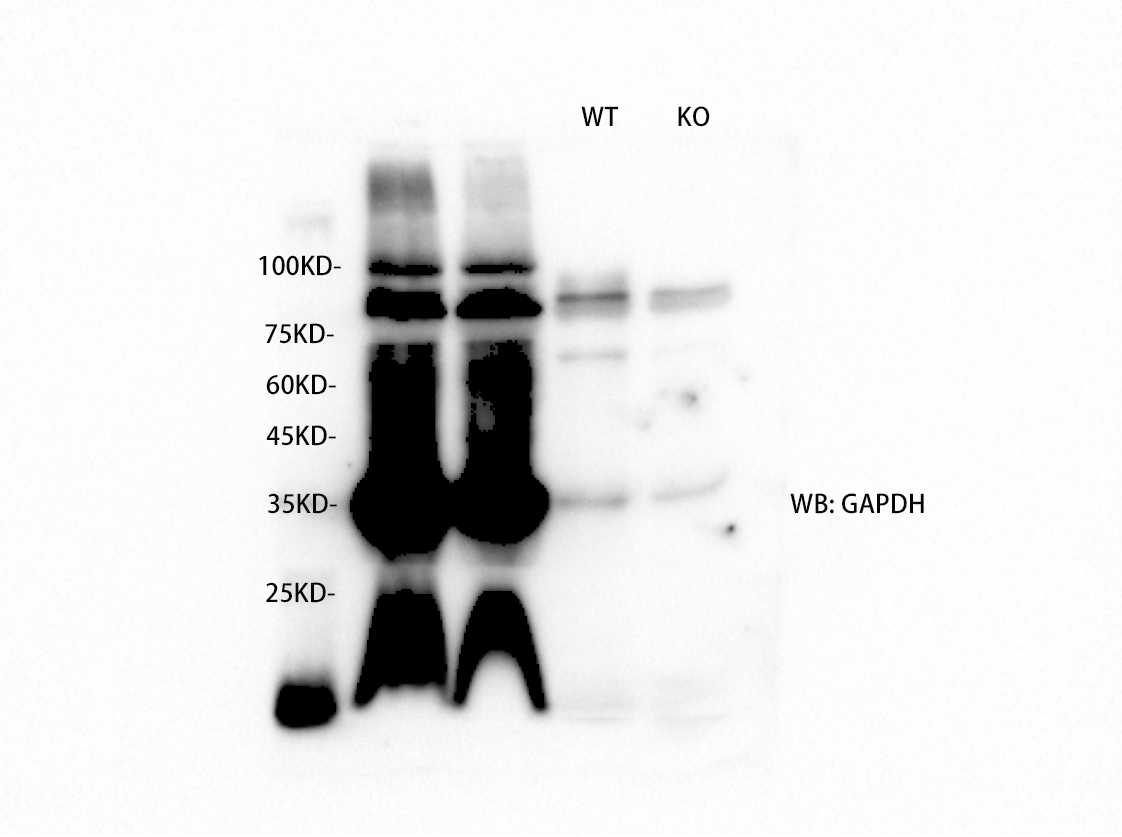

Supplement: Figure 3—source data 1. [file elife-91666-fig3-data1.zip › Figure 3-source data 1/GAPDH-labelled.tif]

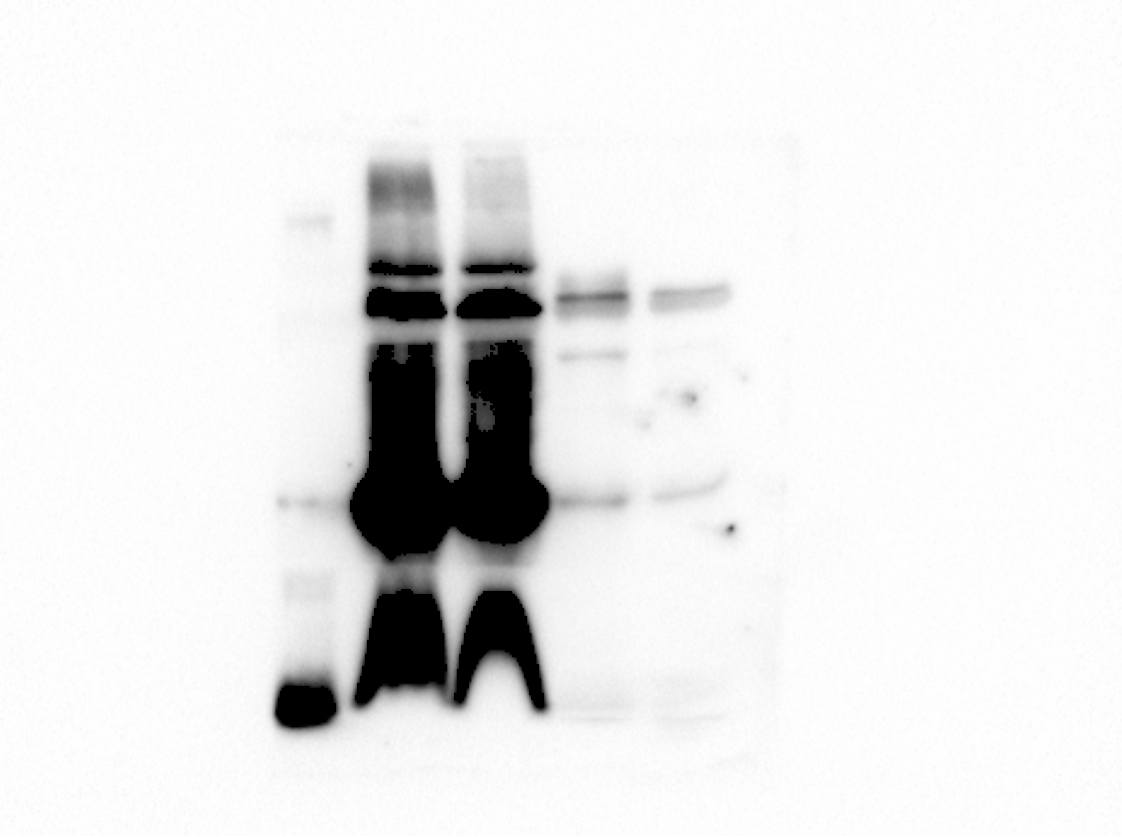

Supplement: Figure 3—source data 1. [file elife-91666-fig3-data1.zip › Figure 3-source data 1/GAPDH-unedited.tif]

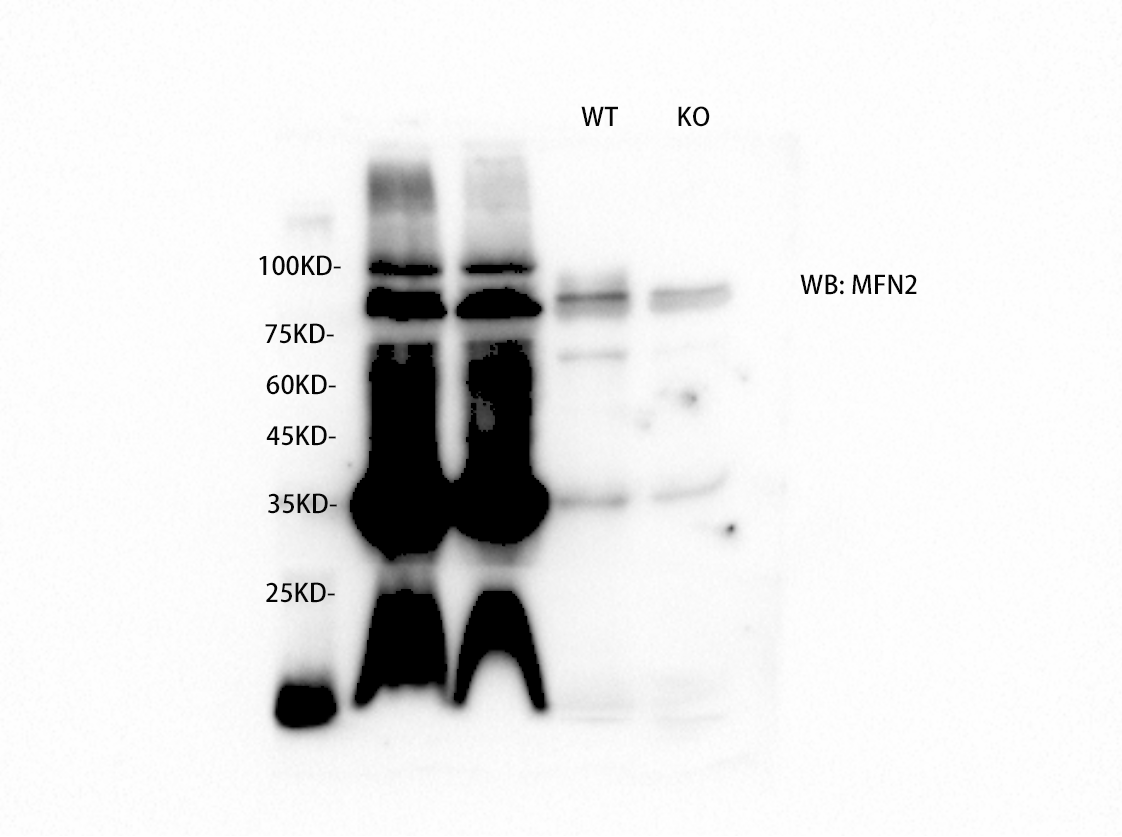

Supplement: Figure 3—source data 1. [file elife-91666-fig3-data1.zip › Figure 3-source data 1/MFN2-labelled.tif]

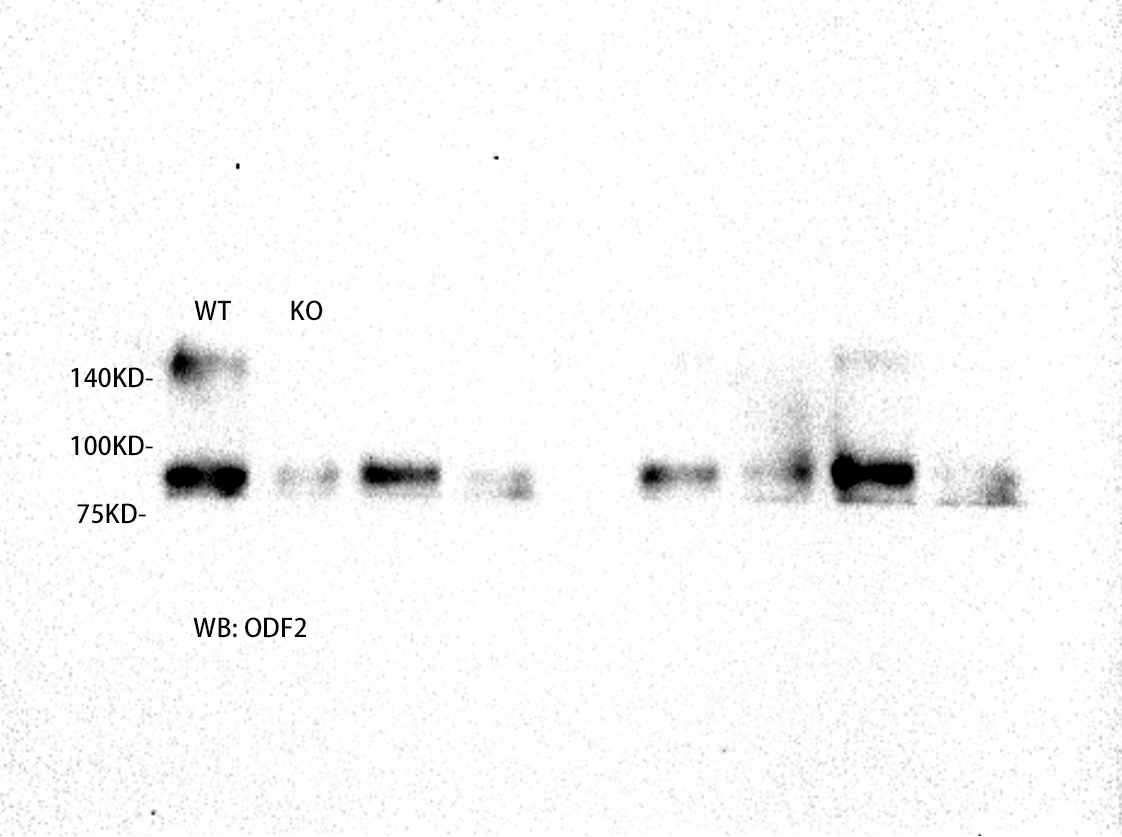

Supplement: Figure 3—source data 1. [file elife-91666-fig3-data1.zip › Figure 3-source data 1/ODF2-labelled.tif]

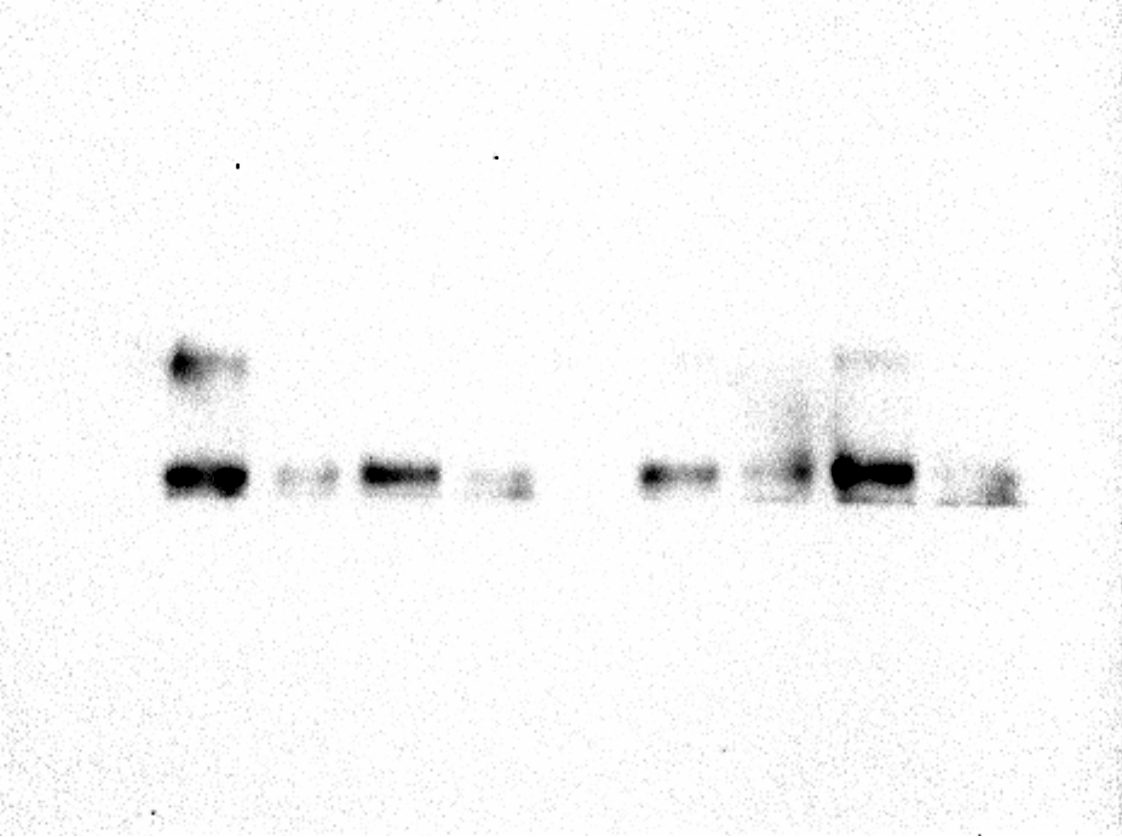

Supplement: Figure 3—source data 1. [file elife-91666-fig3-data1.zip › Figure 3-source data 1/ODF2-unedited.tif]

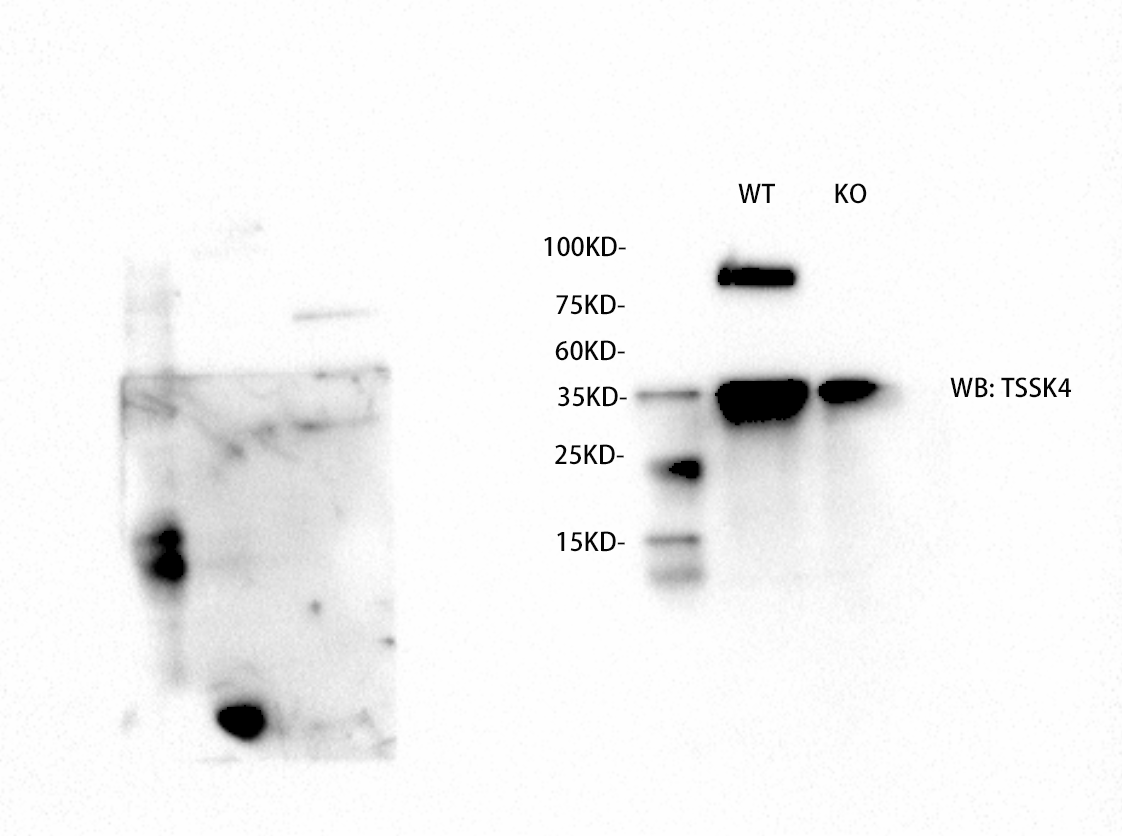

Supplement: Figure 3—source data 1. [file elife-91666-fig3-data1.zip › Figure 3-source data 1/TSSK4-labelled.tif]

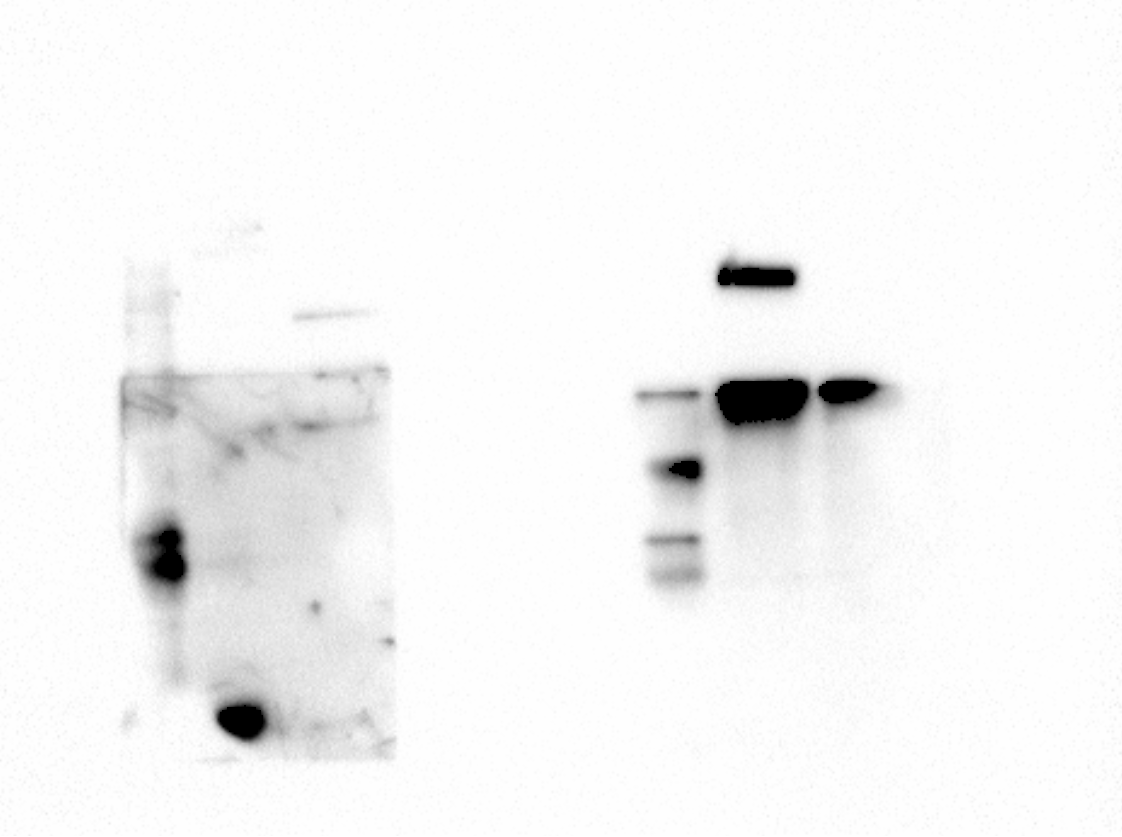

Supplement: Figure 3—source data 1. [file elife-91666-fig3-data1.zip › Figure 3-source data 1/TSSK4-unedited.tif]

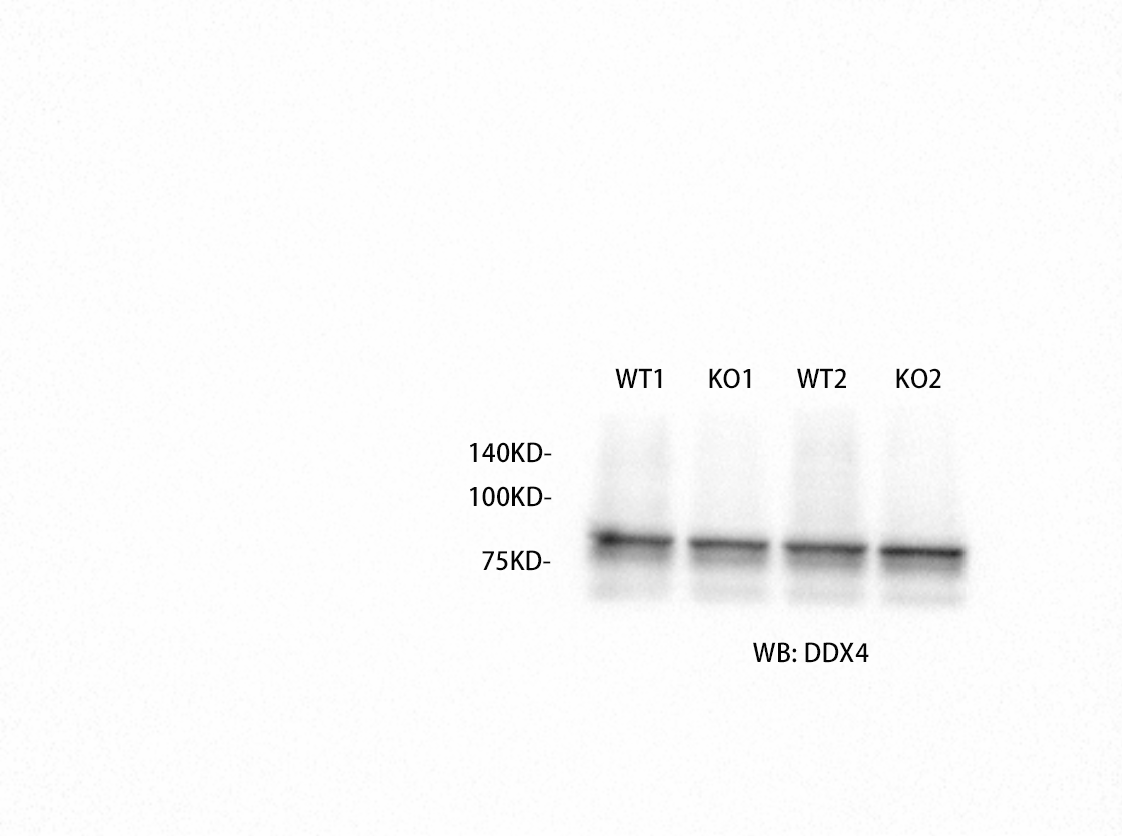

Supplement: Figure 4—source data 1. [file elife-91666-fig4-data1.zip › Figure 4-source data 1/DDX4-labelled.tif]

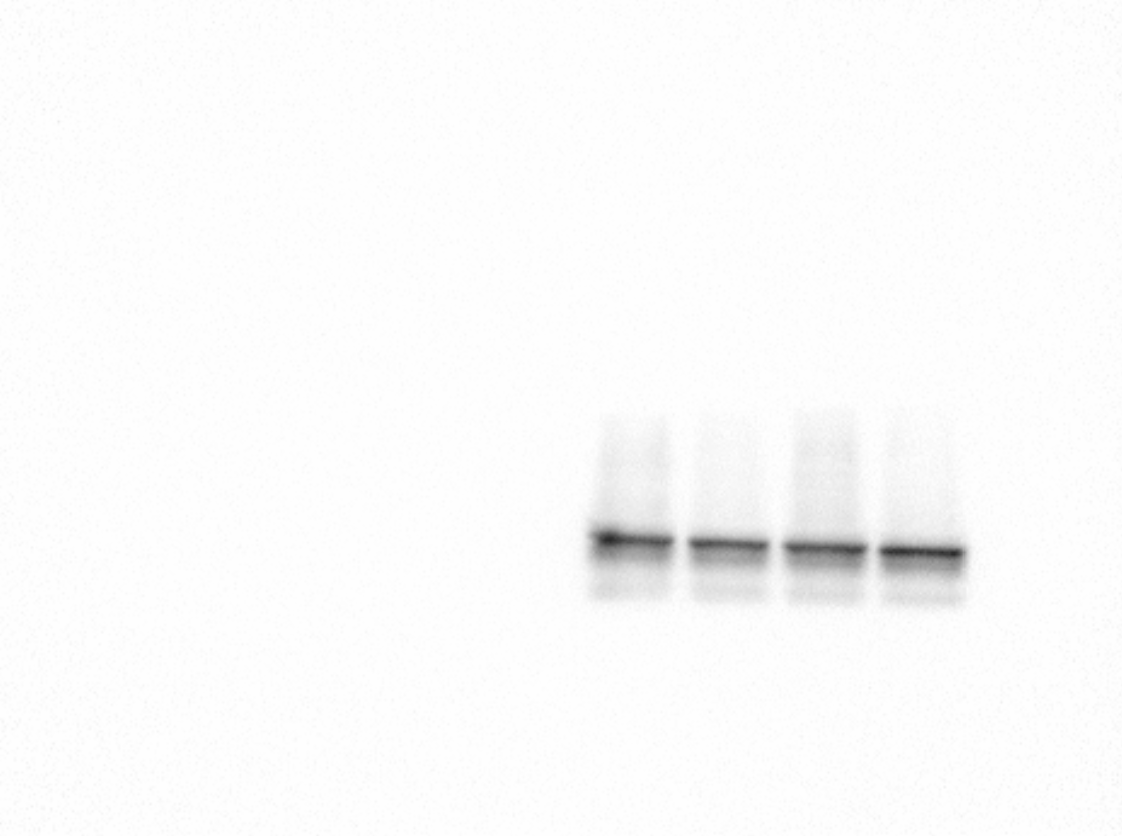

Supplement: Figure 4—source data 1. [file elife-91666-fig4-data1.zip › Figure 4-source data 1/DDX4-unedited.tif]

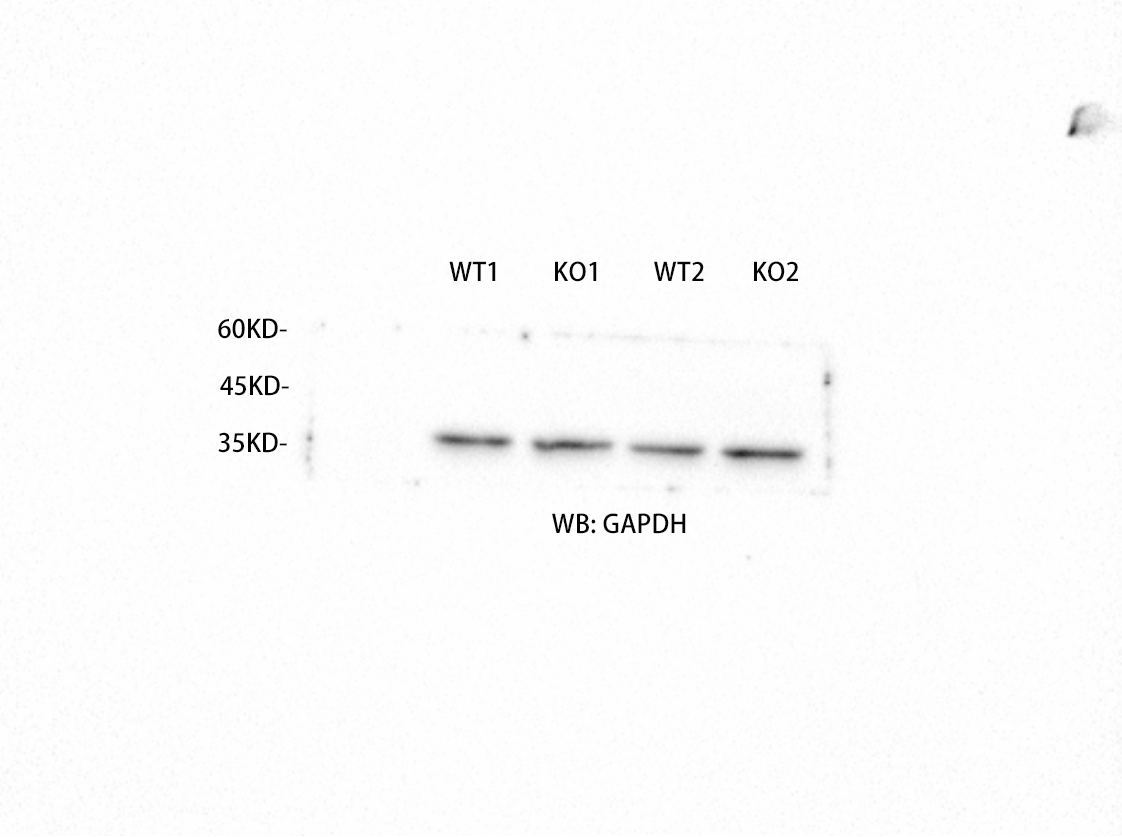

Supplement: Figure 4—source data 1. [file elife-91666-fig4-data1.zip › Figure 4-source data 1/GAPDH(2)-labelled.tif]

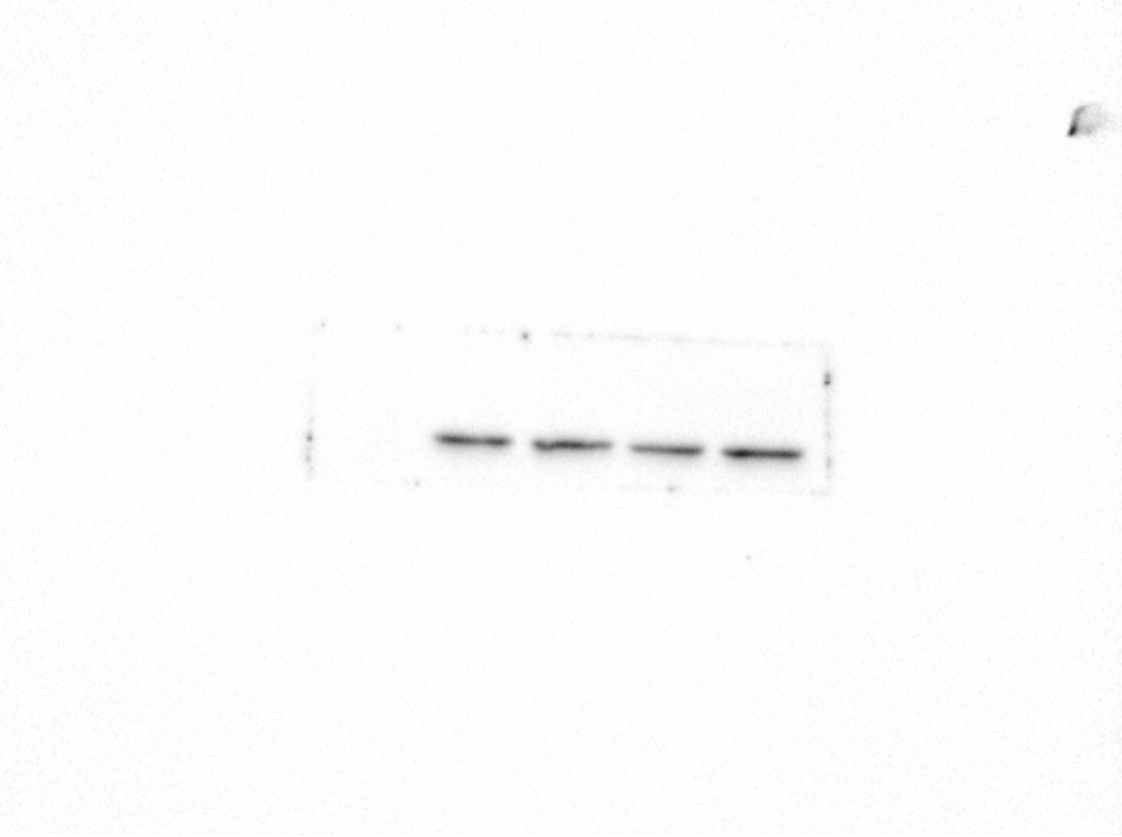

Supplement: Figure 4—source data 1. [file elife-91666-fig4-data1.zip › Figure 4-source data 1/GAPDH(2)-unedited.tif]

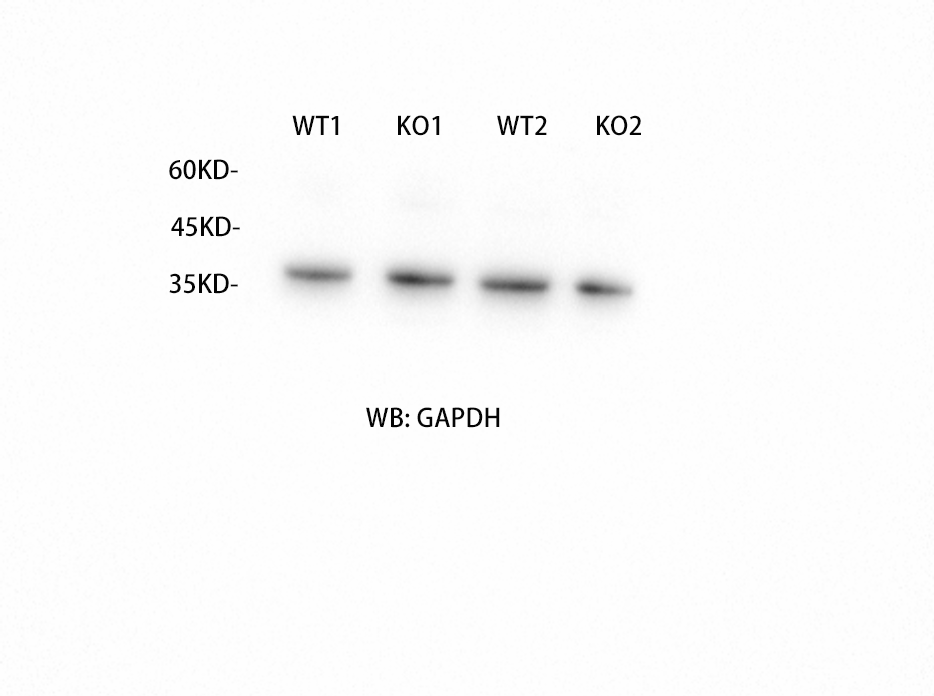

Supplement: Figure 4—source data 1. [file elife-91666-fig4-data1.zip › Figure 4-source data 1/GAPDH(3)-labelled.tif]

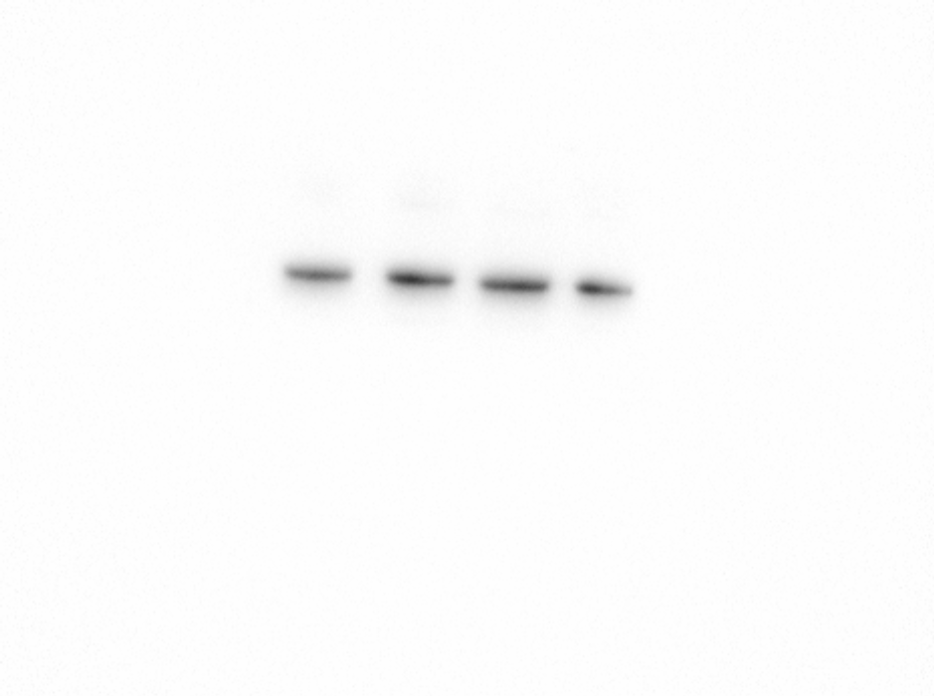

Supplement: Figure 4—source data 1. [file elife-91666-fig4-data1.zip › Figure 4-source data 1/GAPDH(3)-unedited.tif]

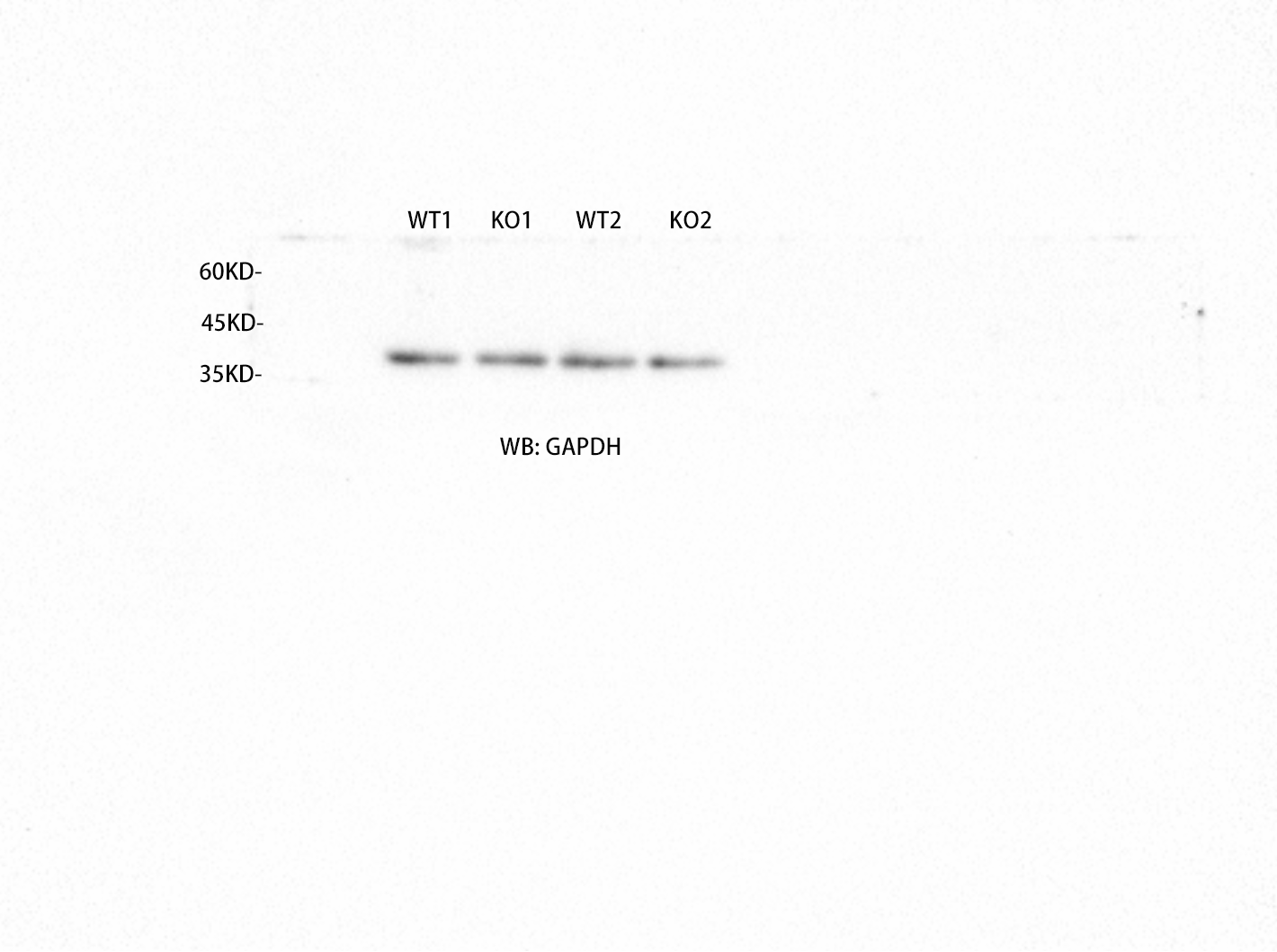

Supplement: Figure 4—source data 1. [file elife-91666-fig4-data1.zip › Figure 4-source data 1/GAPDH-labelled.tif]

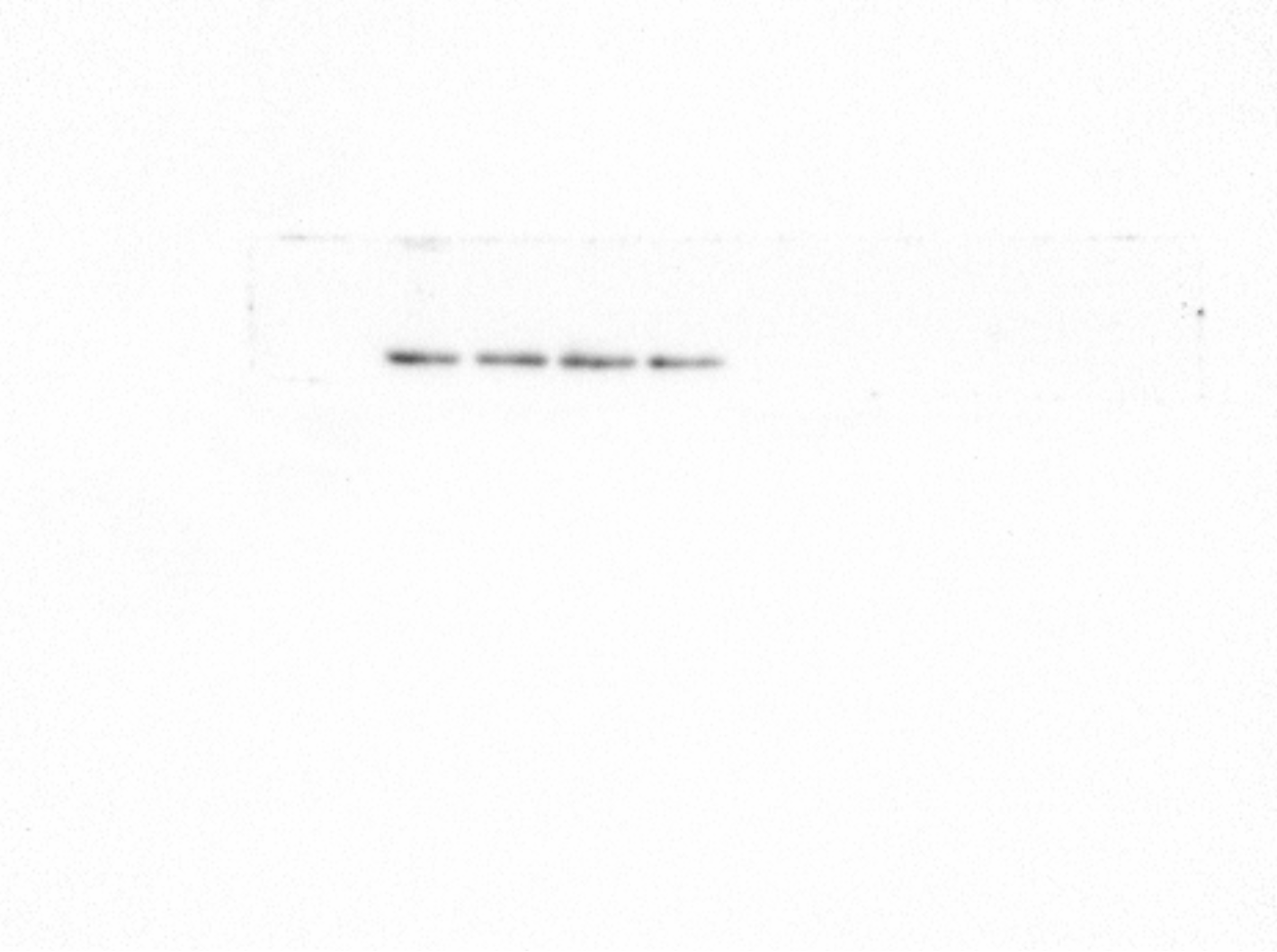

Supplement: Figure 4—source data 1. [file elife-91666-fig4-data1.zip › Figure 4-source data 1/GAPDH-unedited.tif]

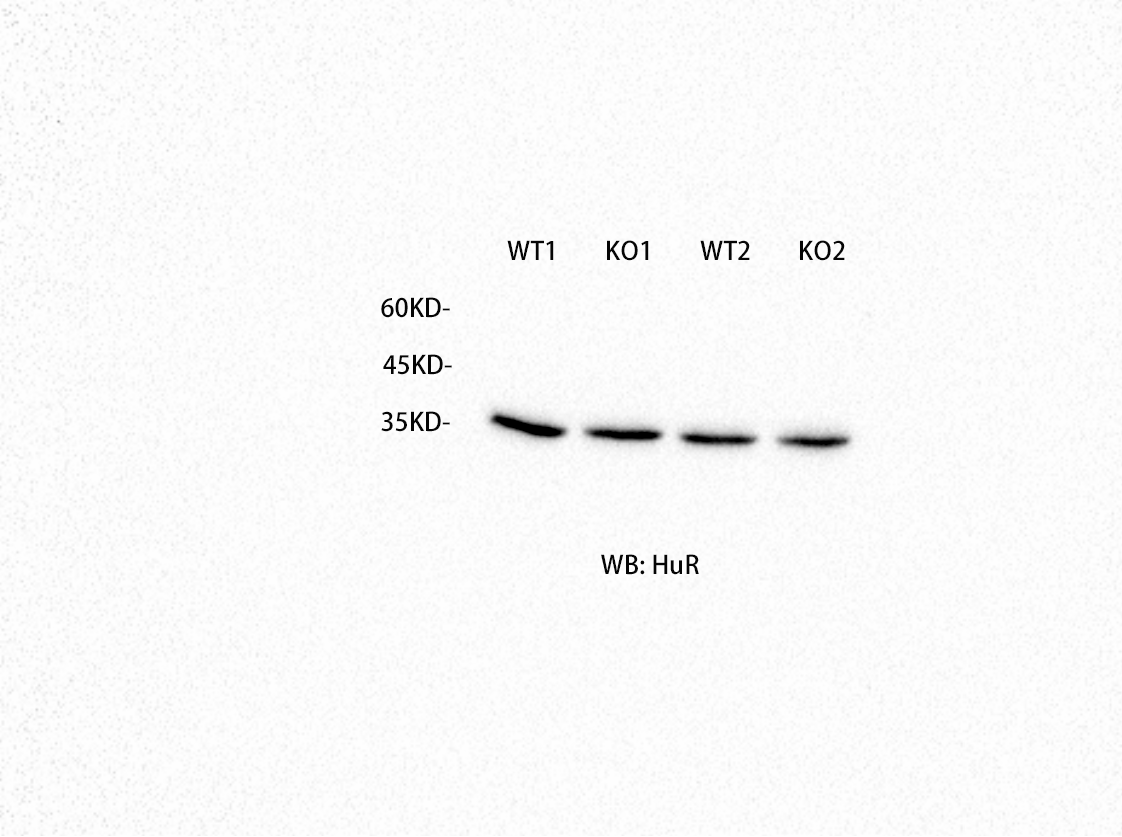

Supplement: Figure 4—source data 1. [file elife-91666-fig4-data1.zip › Figure 4-source data 1/HuR-labelled.tif]

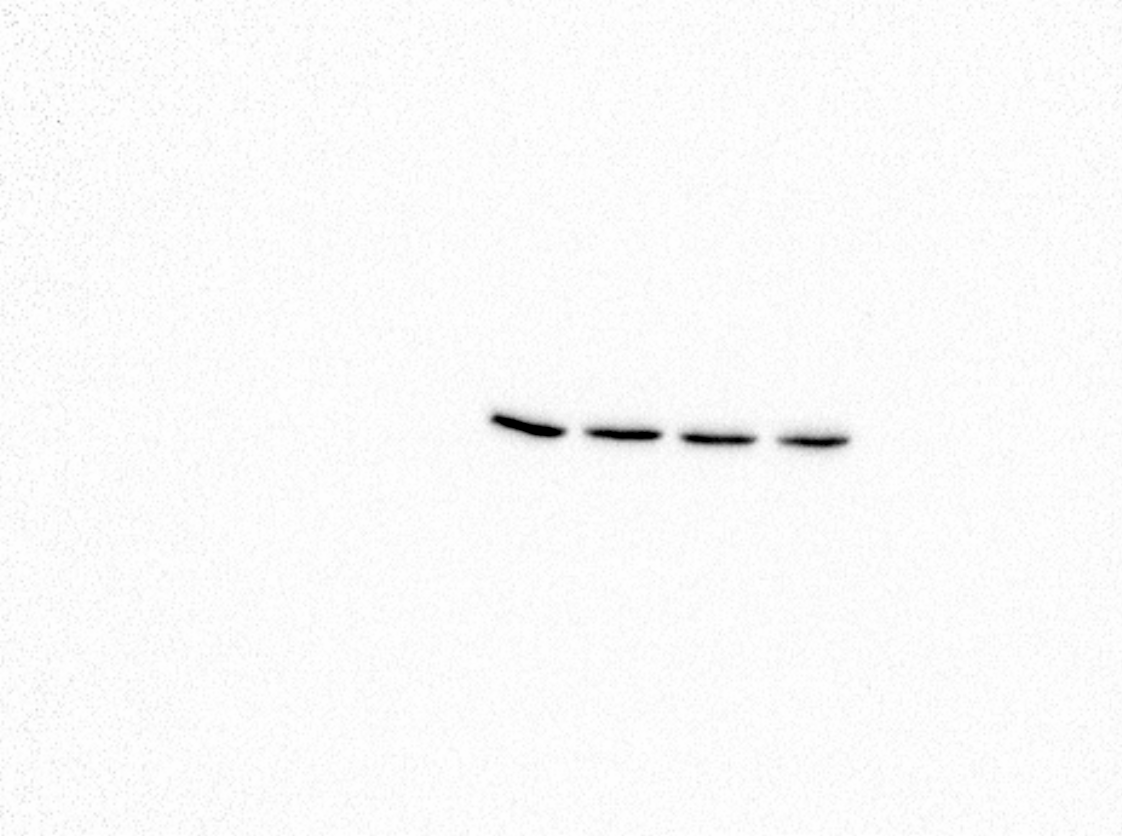

Supplement: Figure 4—source data 1. [file elife-91666-fig4-data1.zip › Figure 4-source data 1/HuR-unedited.tif]

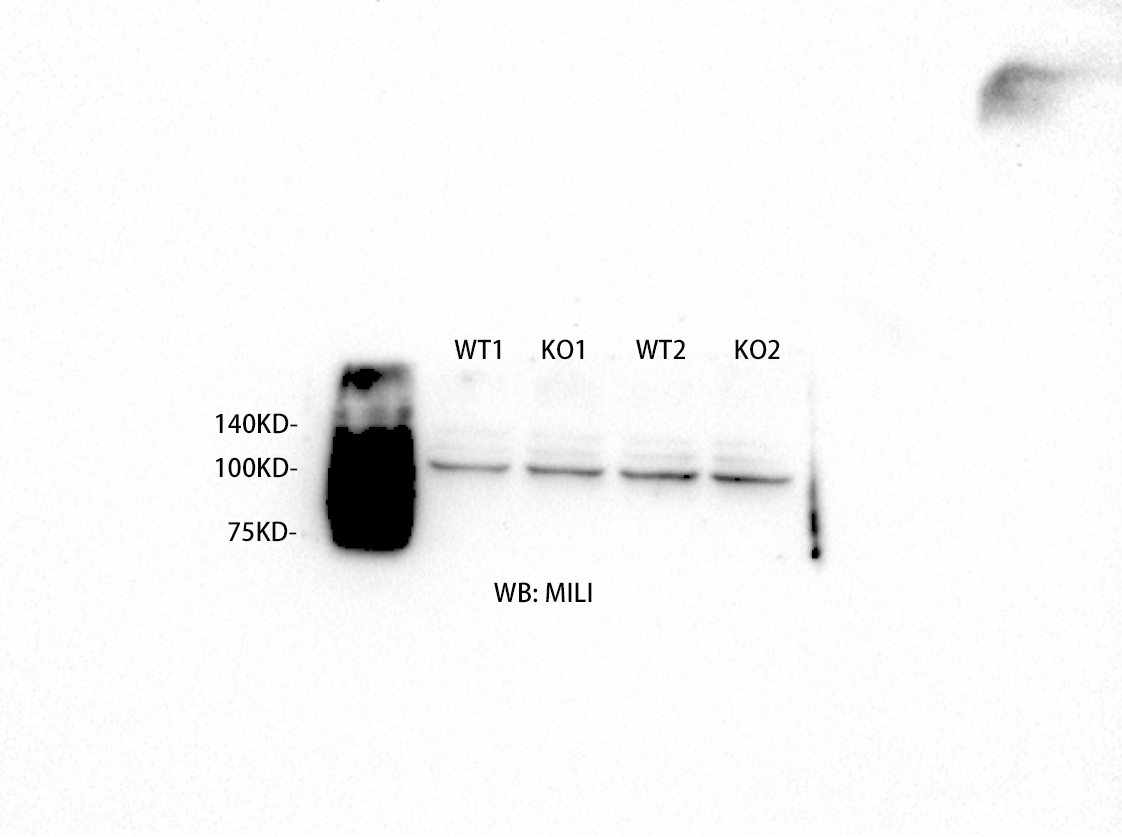

Supplement: Figure 4—source data 1. [file elife-91666-fig4-data1.zip › Figure 4-source data 1/MILI-labelled.tif]

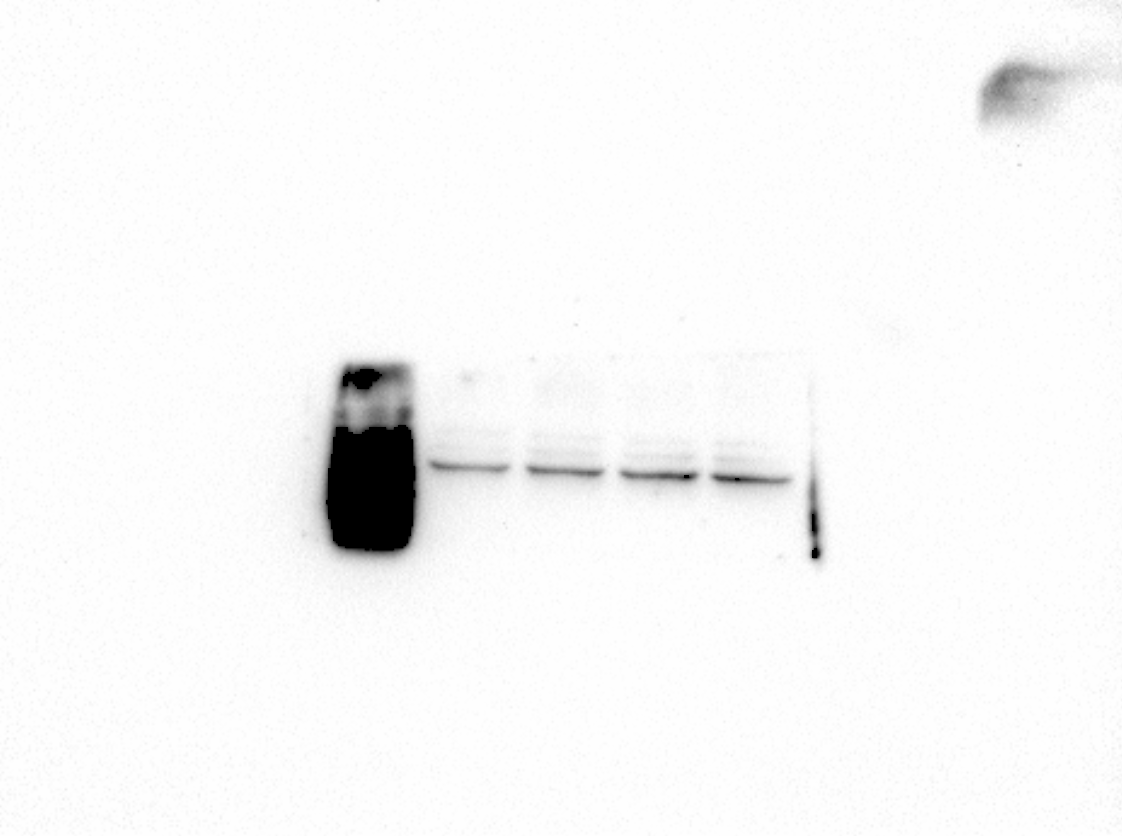

Supplement: Figure 4—source data 1. [file elife-91666-fig4-data1.zip › Figure 4-source data 1/MILI-unedited.tif]

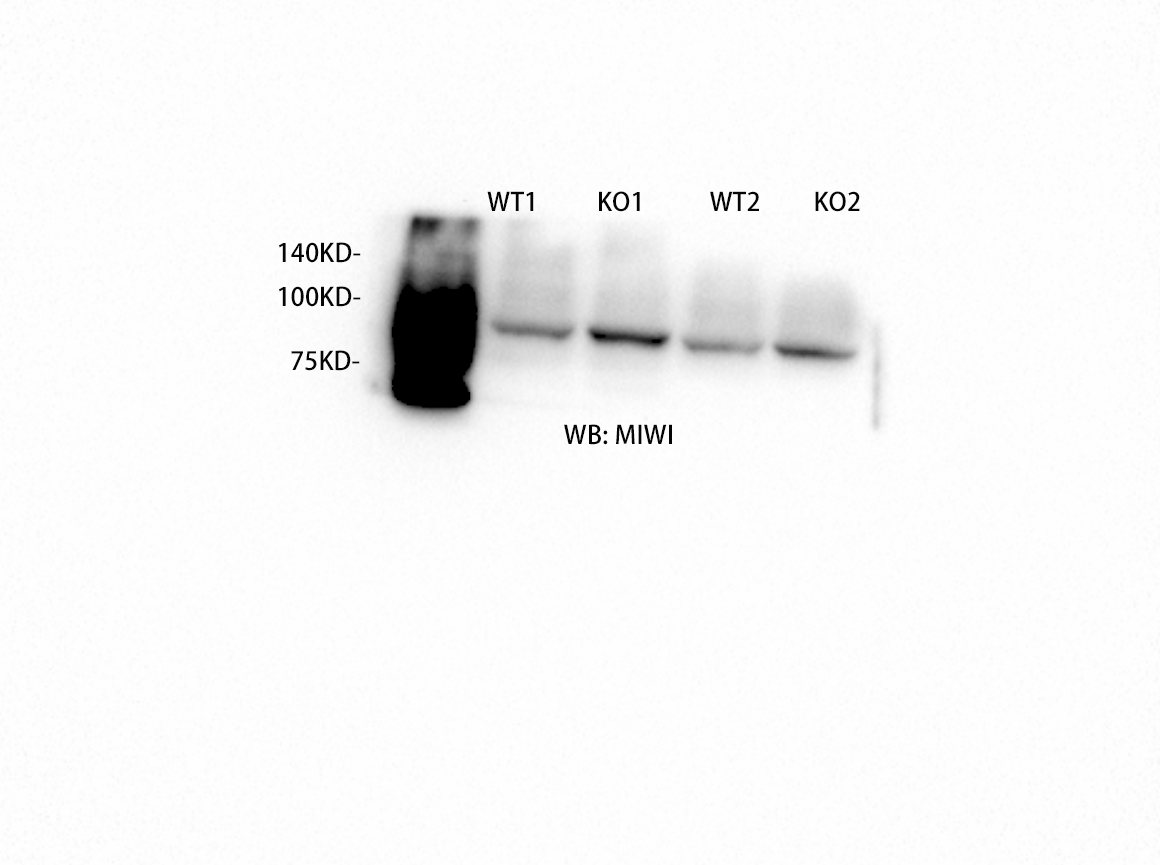

Supplement: Figure 4—source data 1. [file elife-91666-fig4-data1.zip › Figure 4-source data 1/MIWI-labelled.tif]

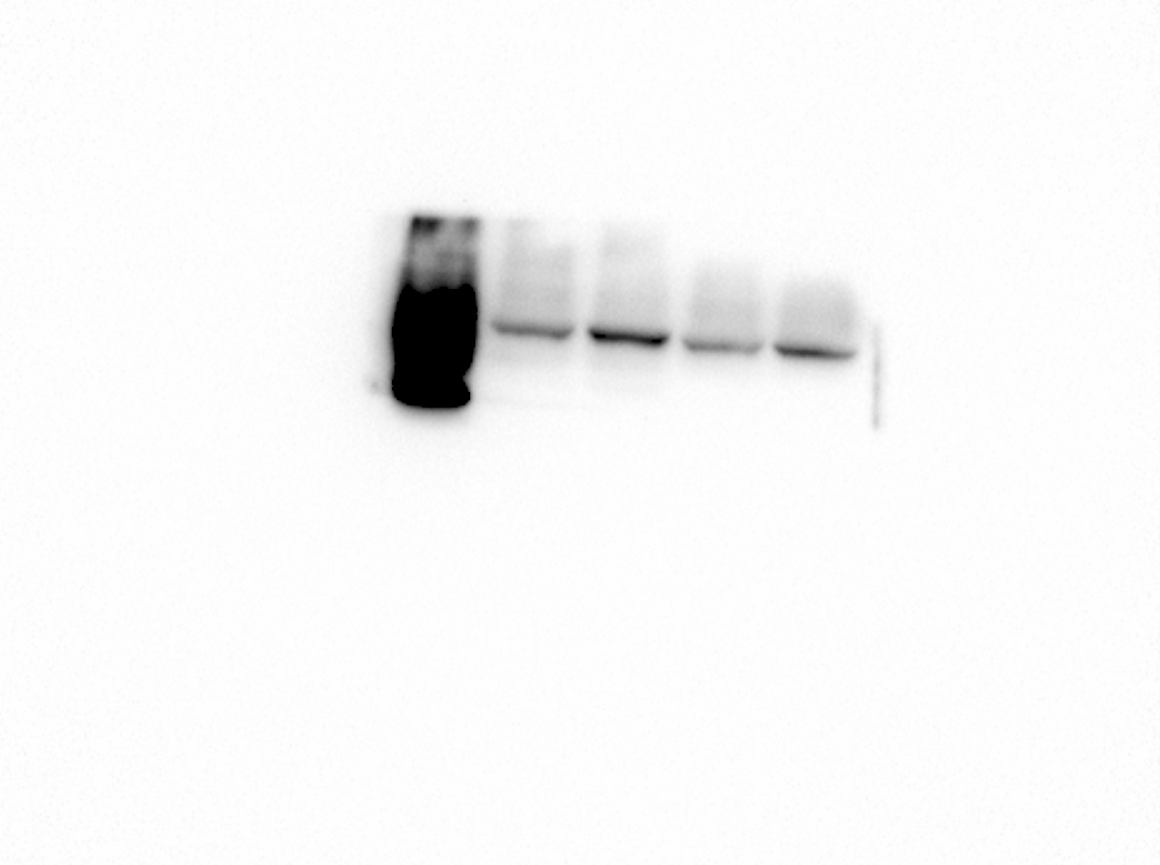

Supplement: Figure 4—source data 1. [file elife-91666-fig4-data1.zip › Figure 4-source data 1/MIWI-unedited.tif]

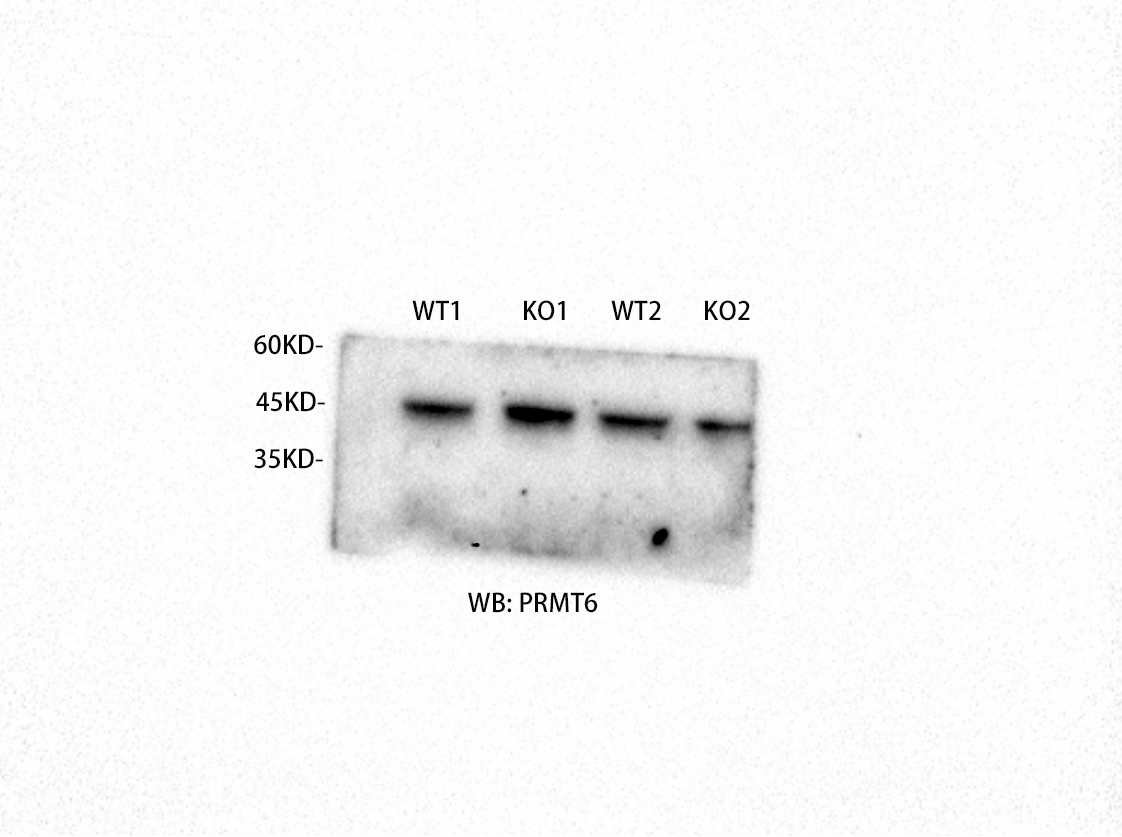

Supplement: Figure 4—source data 1. [file elife-91666-fig4-data1.zip › Figure 4-source data 1/PRMT6-labelled8.tif]

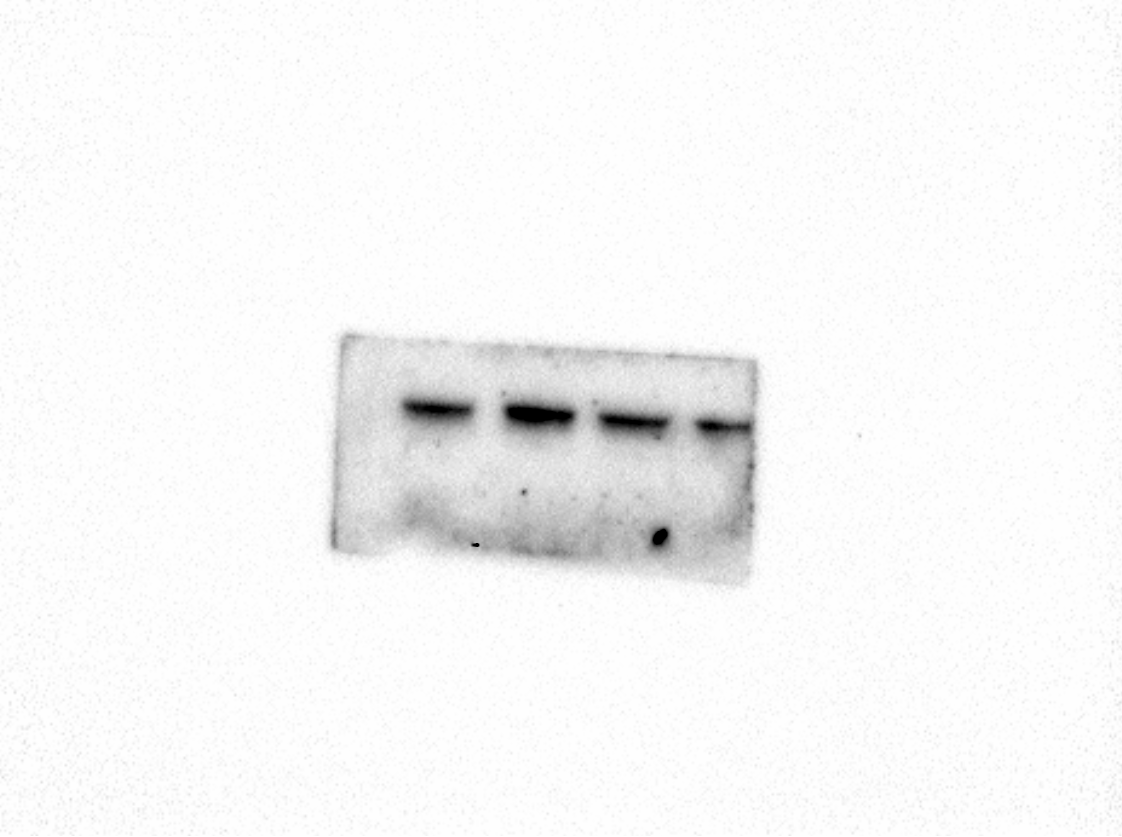

Supplement: Figure 4—source data 1. [file elife-91666-fig4-data1.zip › Figure 4-source data 1/PRMT6-unedited.tif]

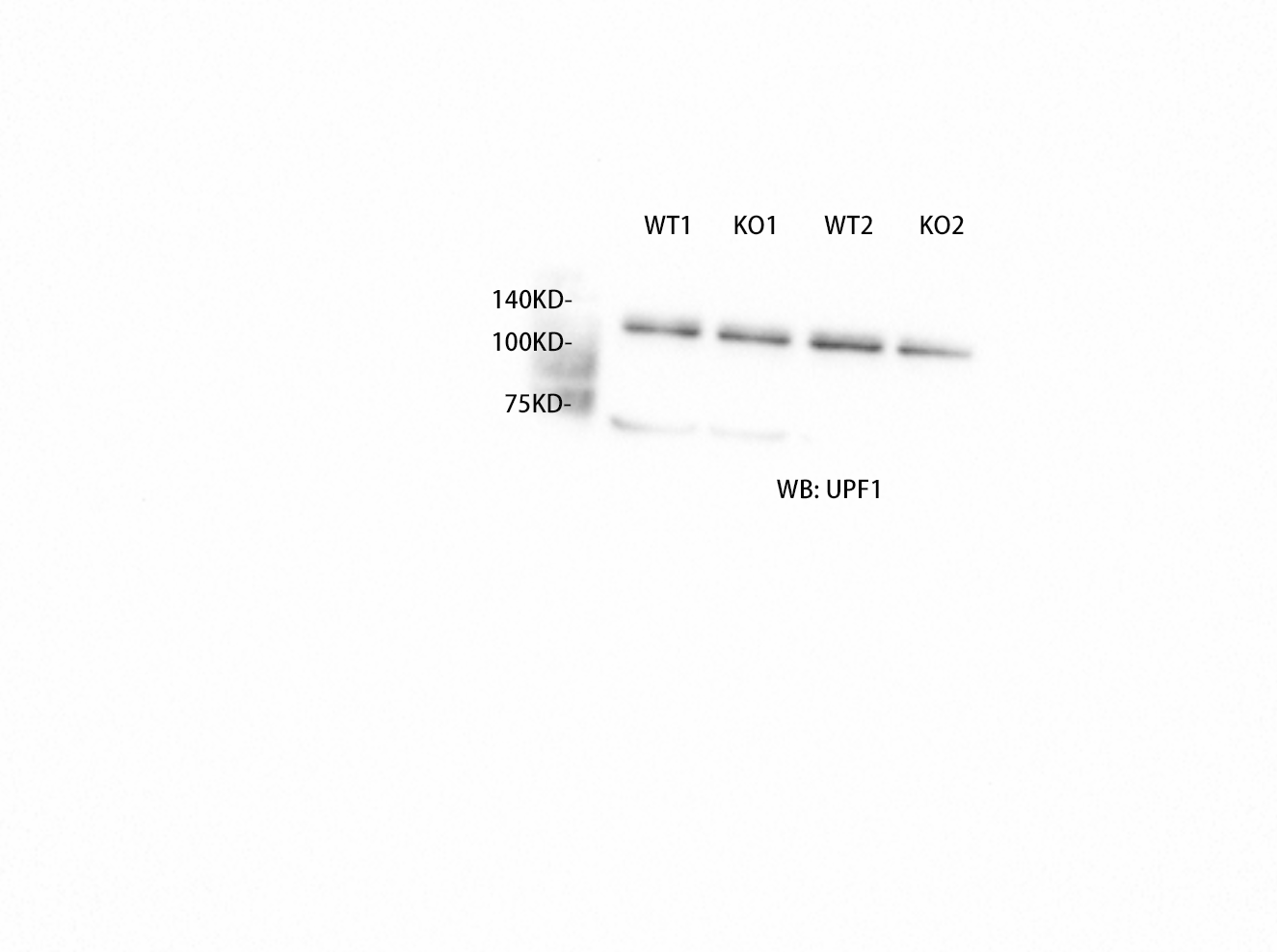

Supplement: Figure 4—source data 1. [file elife-91666-fig4-data1.zip › Figure 4-source data 1/UPF1-labelled.tif]

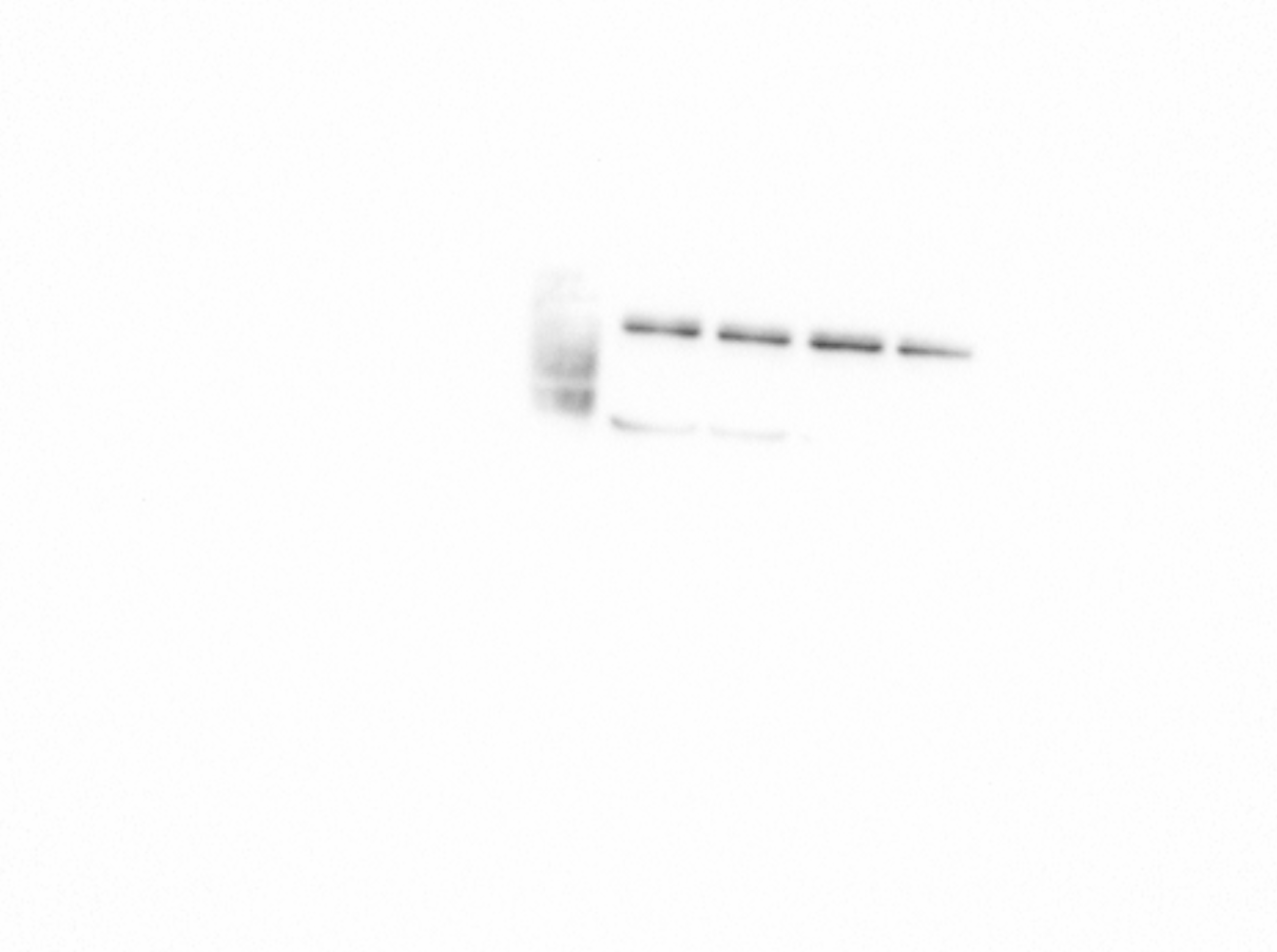

Supplement: Figure 4—source data 1. [file elife-91666-fig4-data1.zip › Figure 4-source data 1/UPF1-unedited.tif]

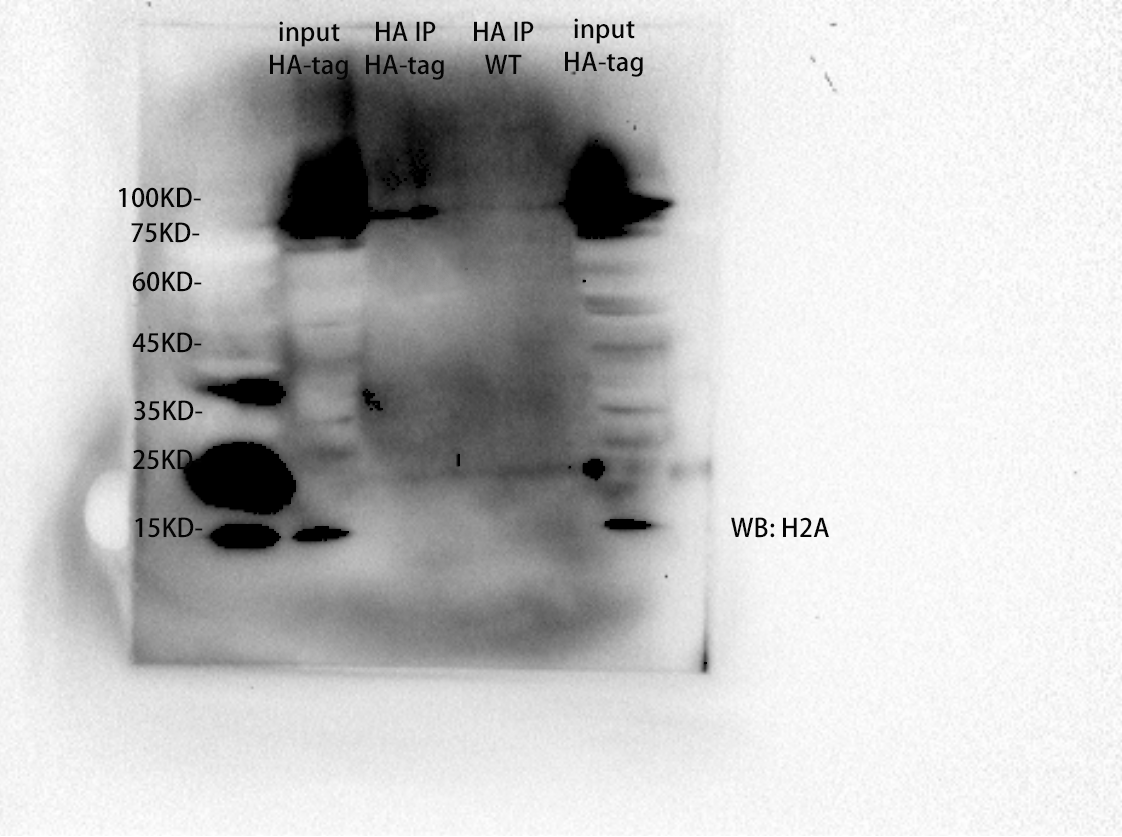

Supplement: Figure 5—source data 1. [file elife-91666-fig5-data1.zip › Figure 5-Figure supplement 4-source data 1/H2A-labelled.tif]

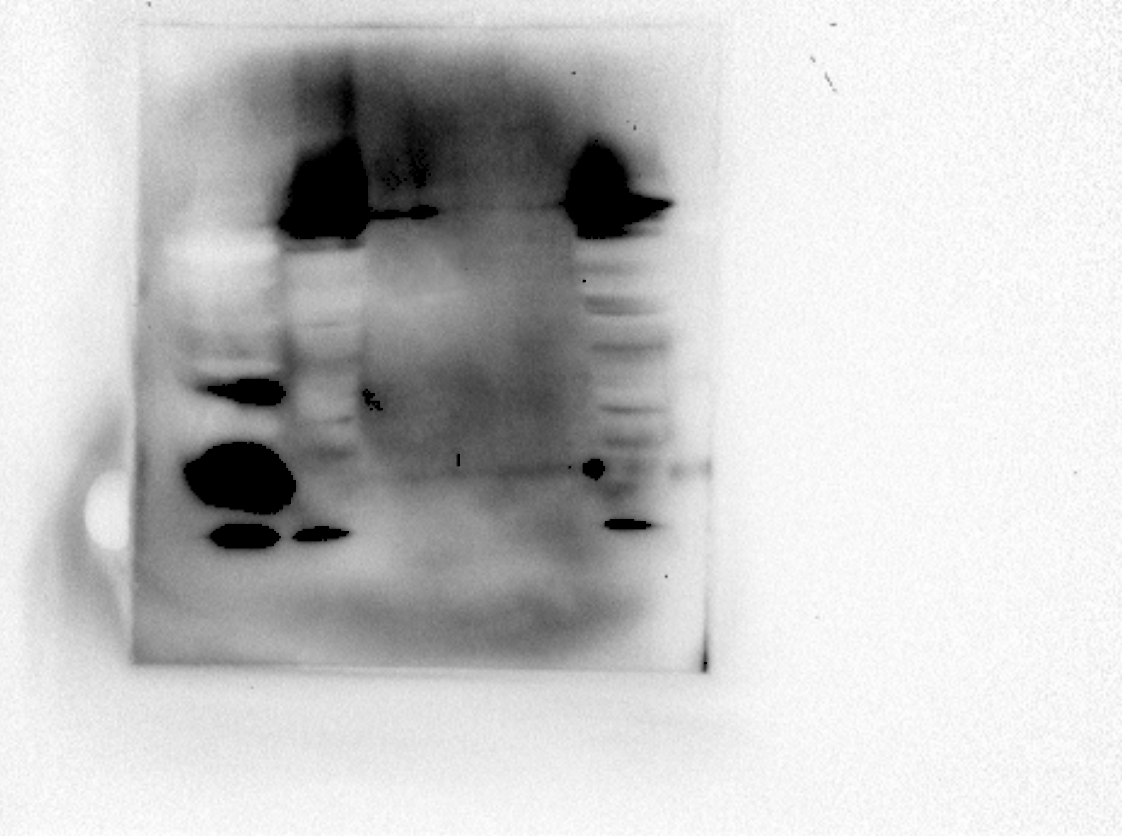

Supplement: Figure 5—source data 1. [file elife-91666-fig5-data1.zip › Figure 5-Figure supplement 4-source data 1/H2A-unedited.tif]

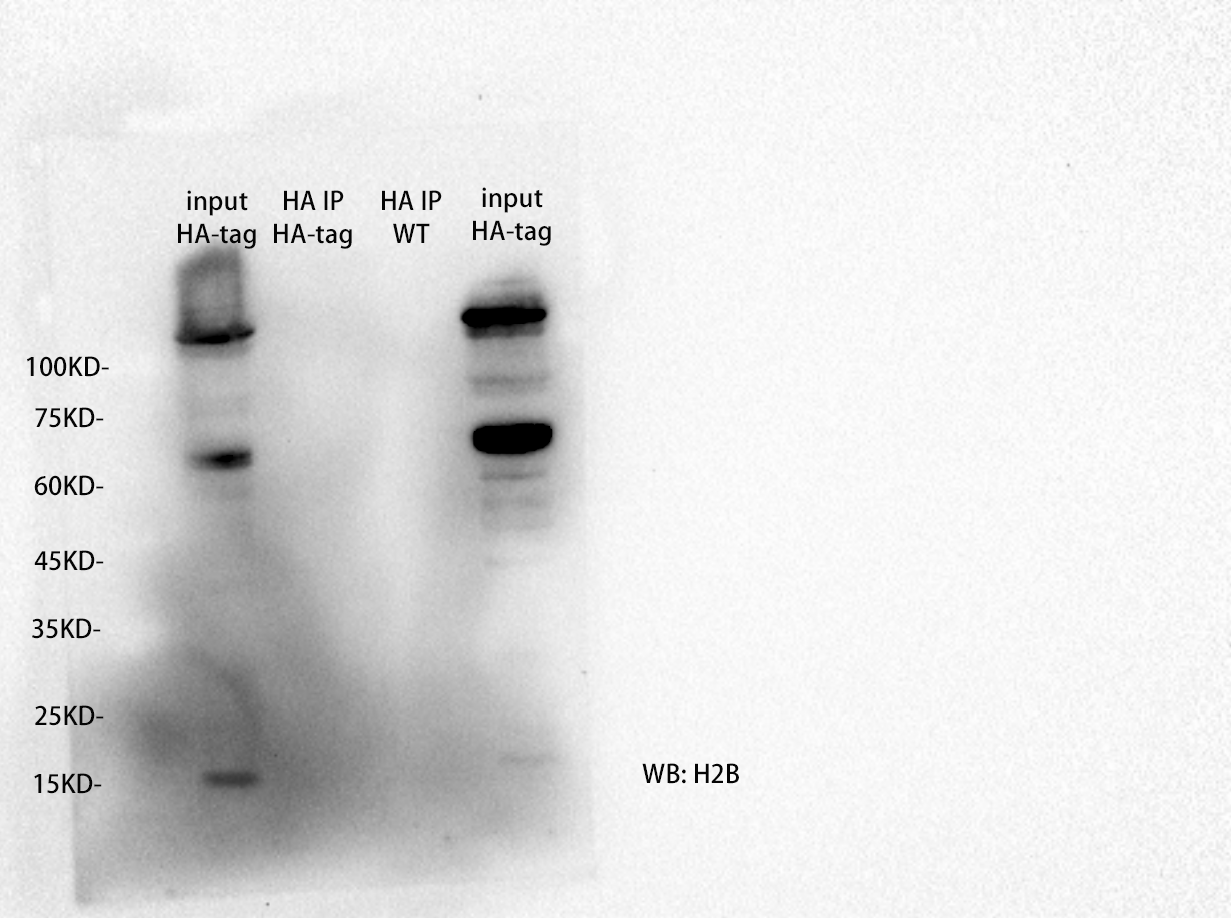

Supplement: Figure 5—source data 1. [file elife-91666-fig5-data1.zip › Figure 5-Figure supplement 4-source data 1/H2B-labelled.tif]

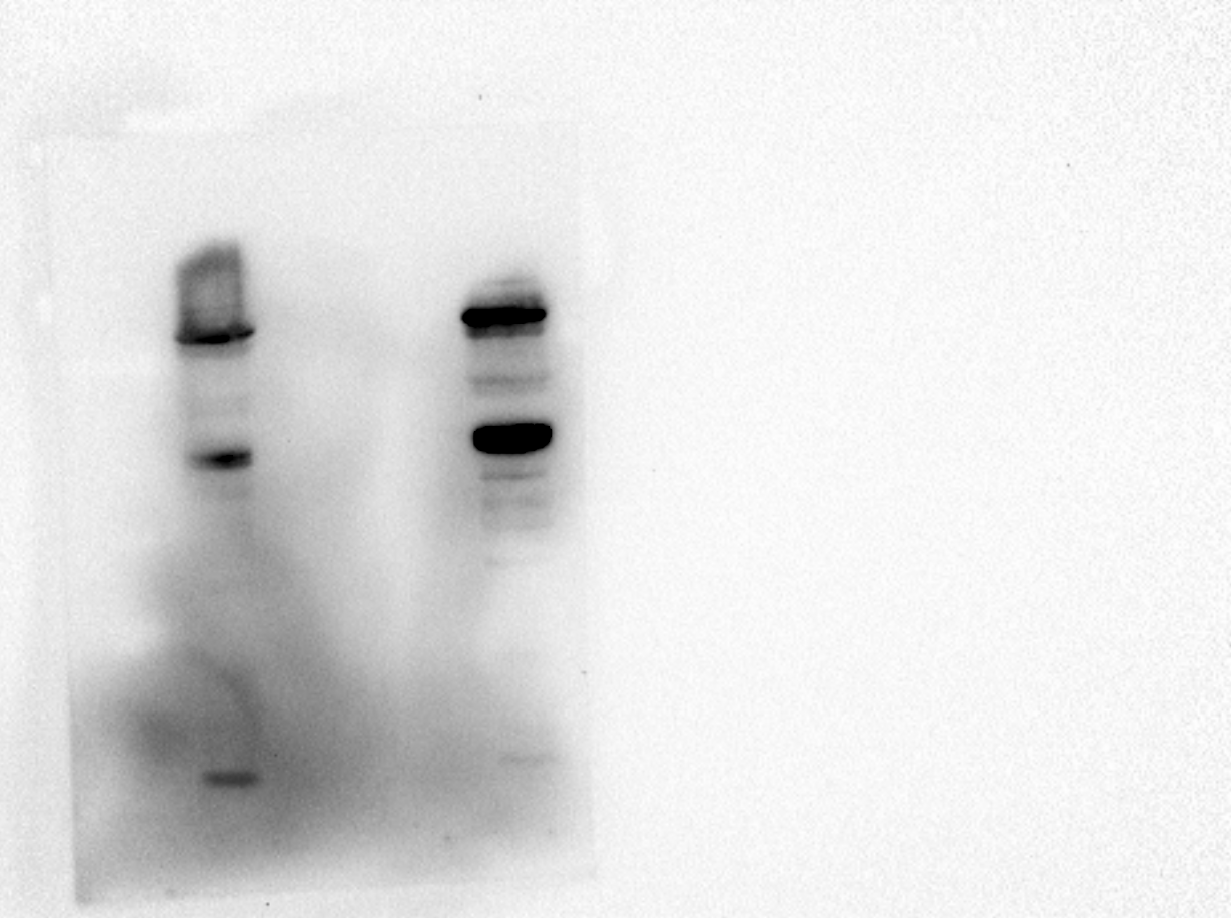

Supplement: Figure 5—source data 1. [file elife-91666-fig5-data1.zip › Figure 5-Figure supplement 4-source data 1/H2B-unedited.tif]

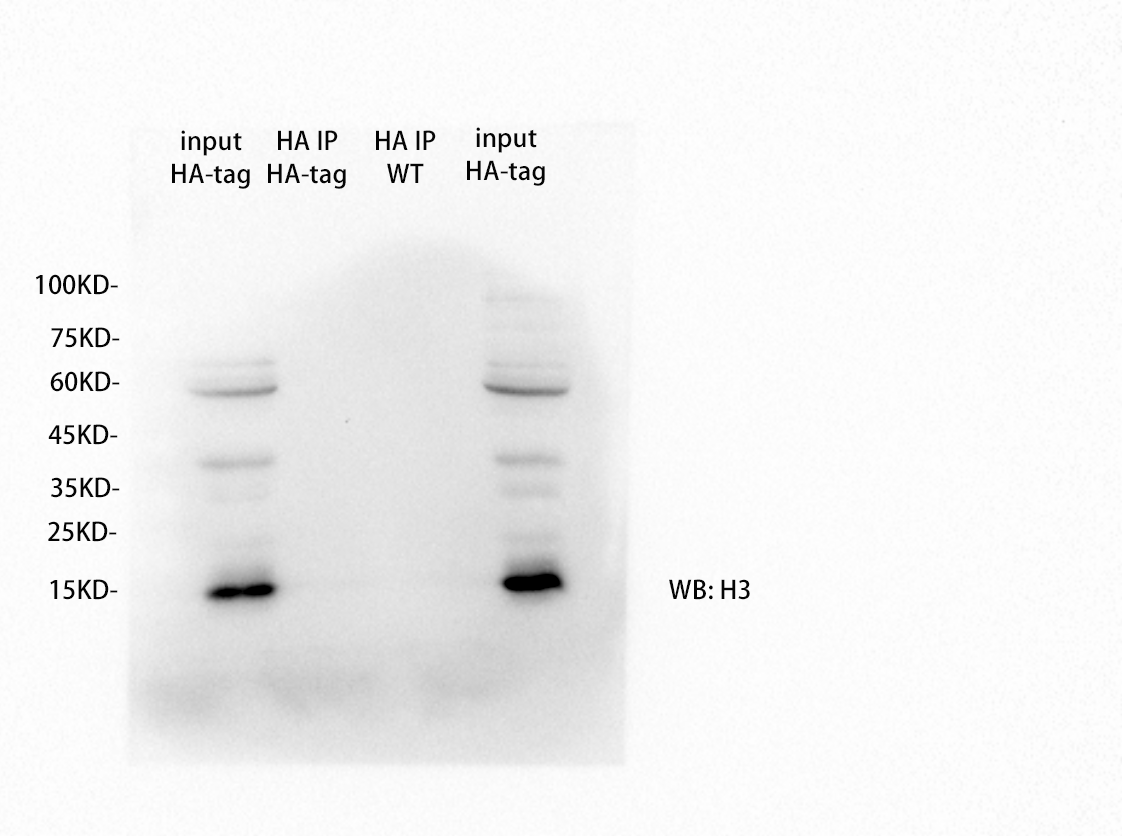

Supplement: Figure 5—source data 1. [file elife-91666-fig5-data1.zip › Figure 5-Figure supplement 4-source data 1/H3-labelled.tif]

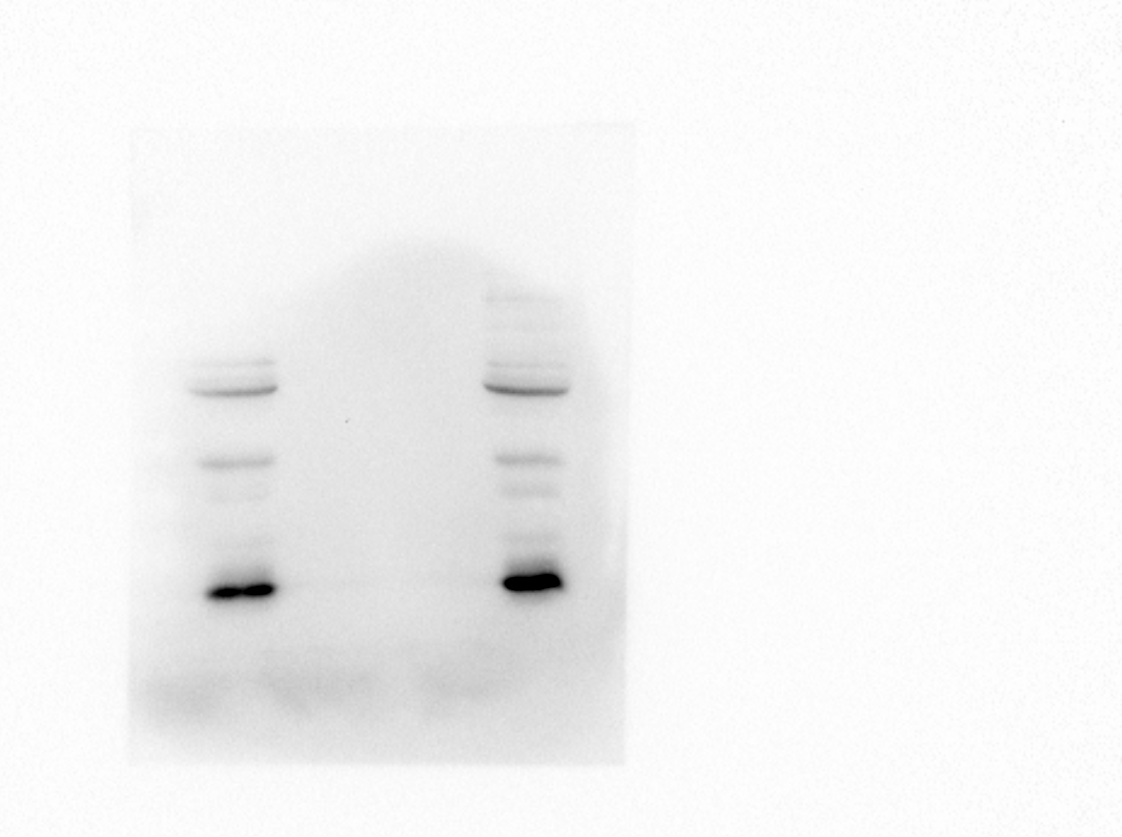

Supplement: Figure 5—source data 1. [file elife-91666-fig5-data1.zip › Figure 5-Figure supplement 4-source data 1/H3-unedited.tif]

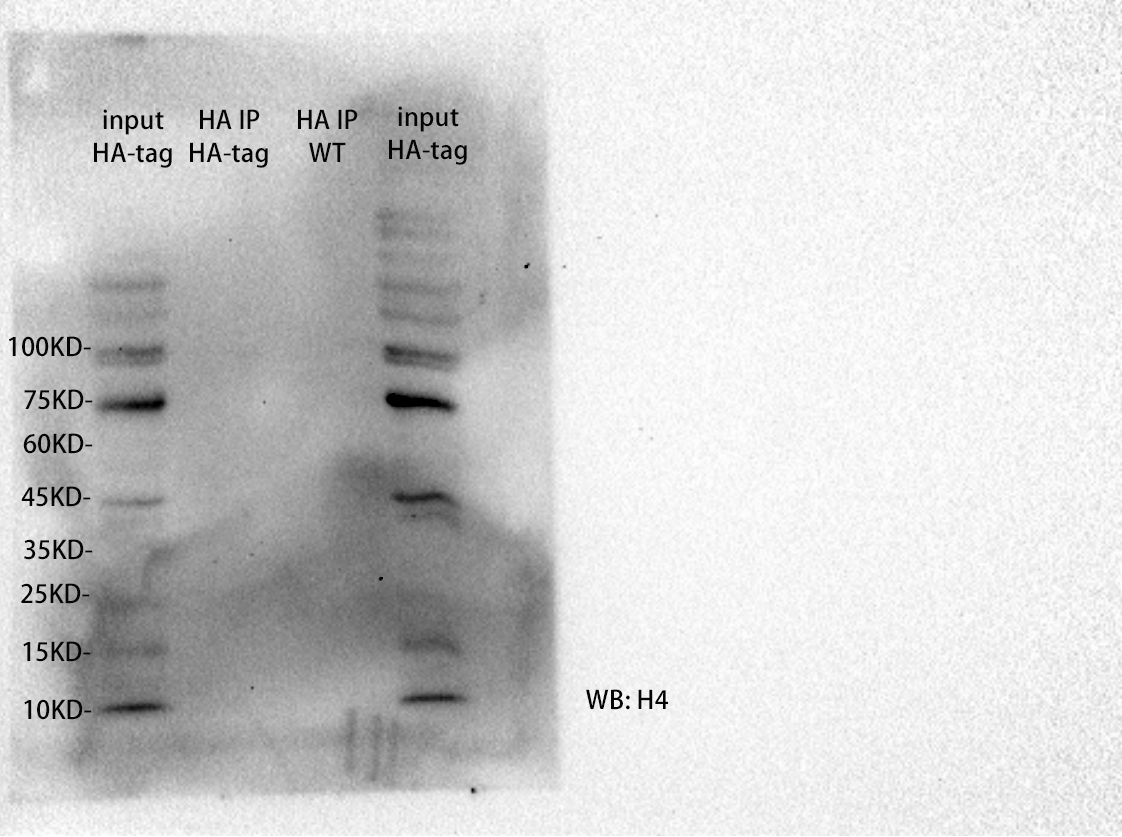

Supplement: Figure 5—source data 1. [file elife-91666-fig5-data1.zip › Figure 5-Figure supplement 4-source data 1/H4-labelled.tif]

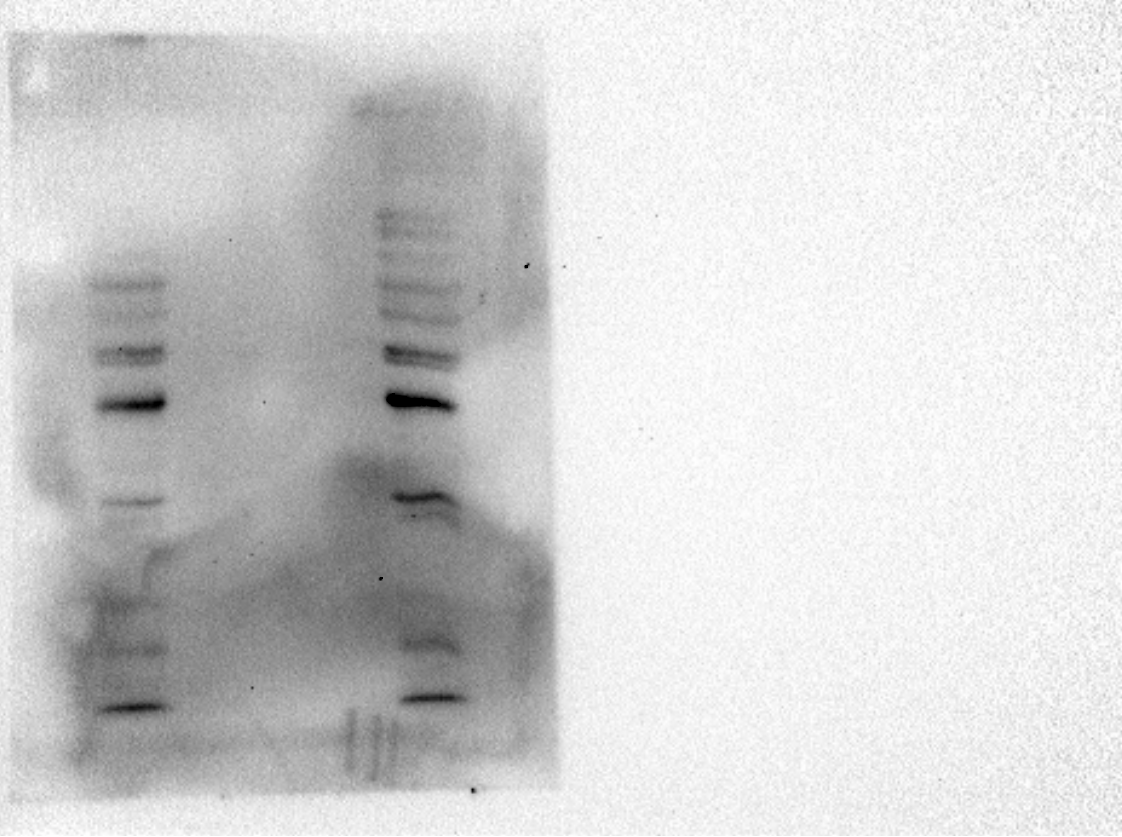

Supplement: Figure 5—source data 1. [file elife-91666-fig5-data1.zip › Figure 5-Figure supplement 4-source data 1/H4-unedited.tif]

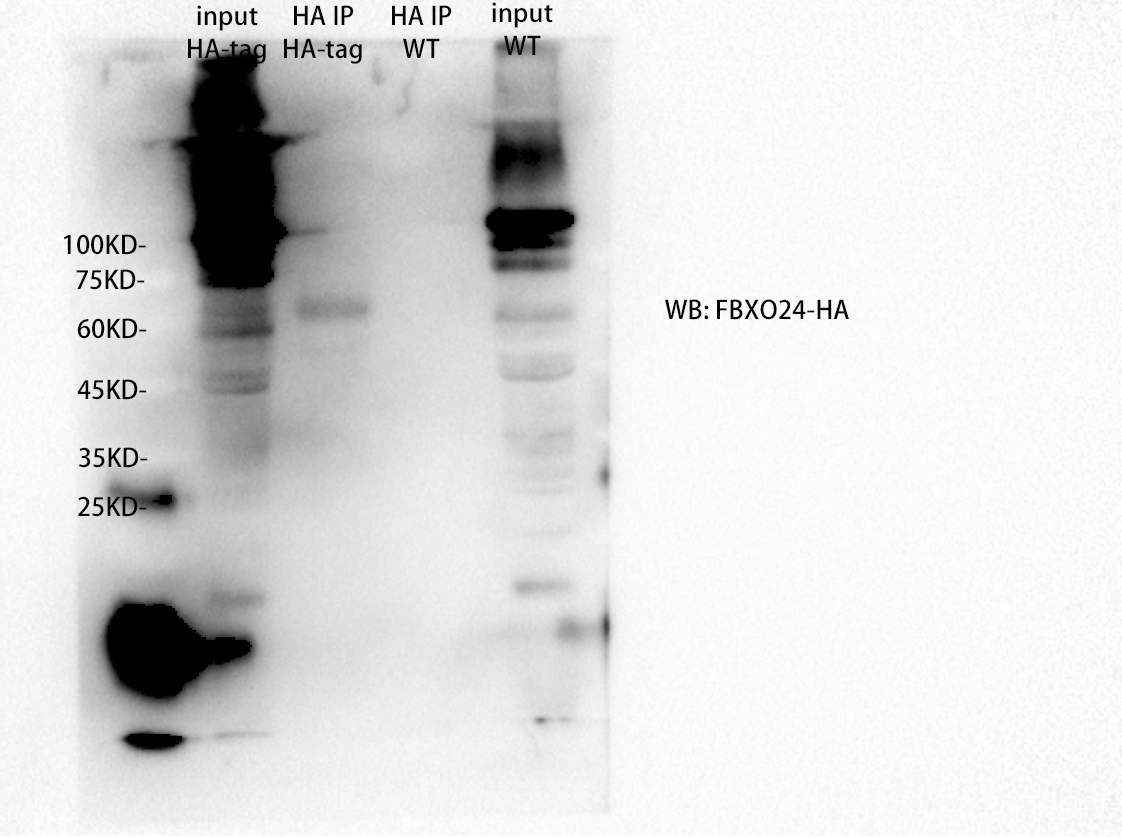

Supplement: Figure 5—source data 1. [file elife-91666-fig5-data1.zip › Figure 5-Figure supplement 4-source data 1/HA(2)-labelled.tif]

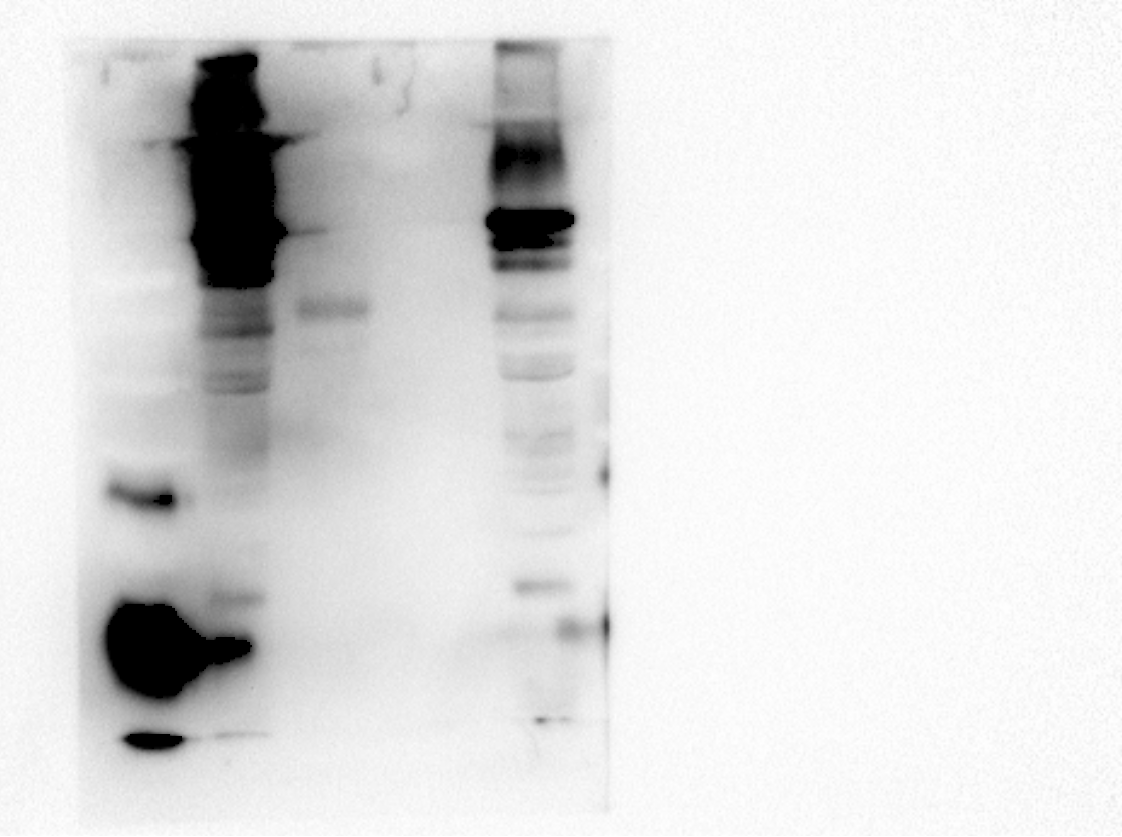

Supplement: Figure 5—source data 1. [file elife-91666-fig5-data1.zip › Figure 5-Figure supplement 4-source data 1/HA(2)-unedited.tif]

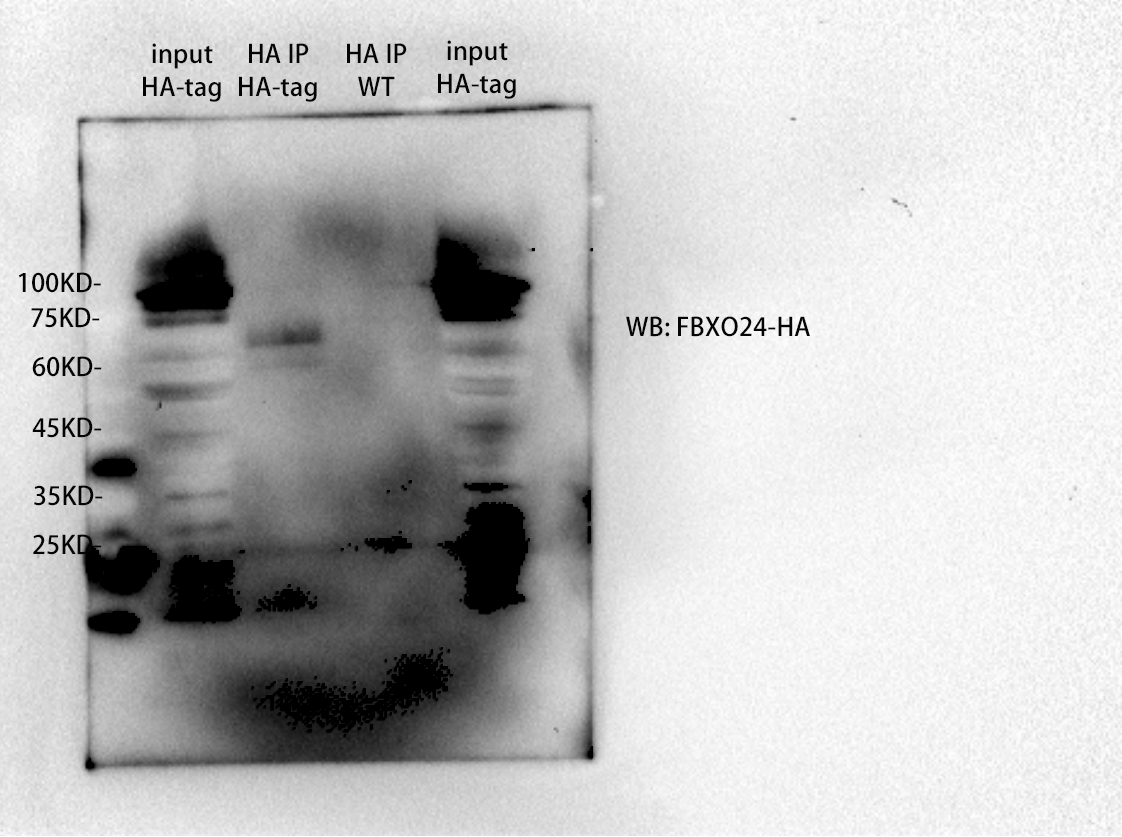

Supplement: Figure 5—source data 1. [file elife-91666-fig5-data1.zip › Figure 5-Figure supplement 4-source data 1/HA-labelled.tif]

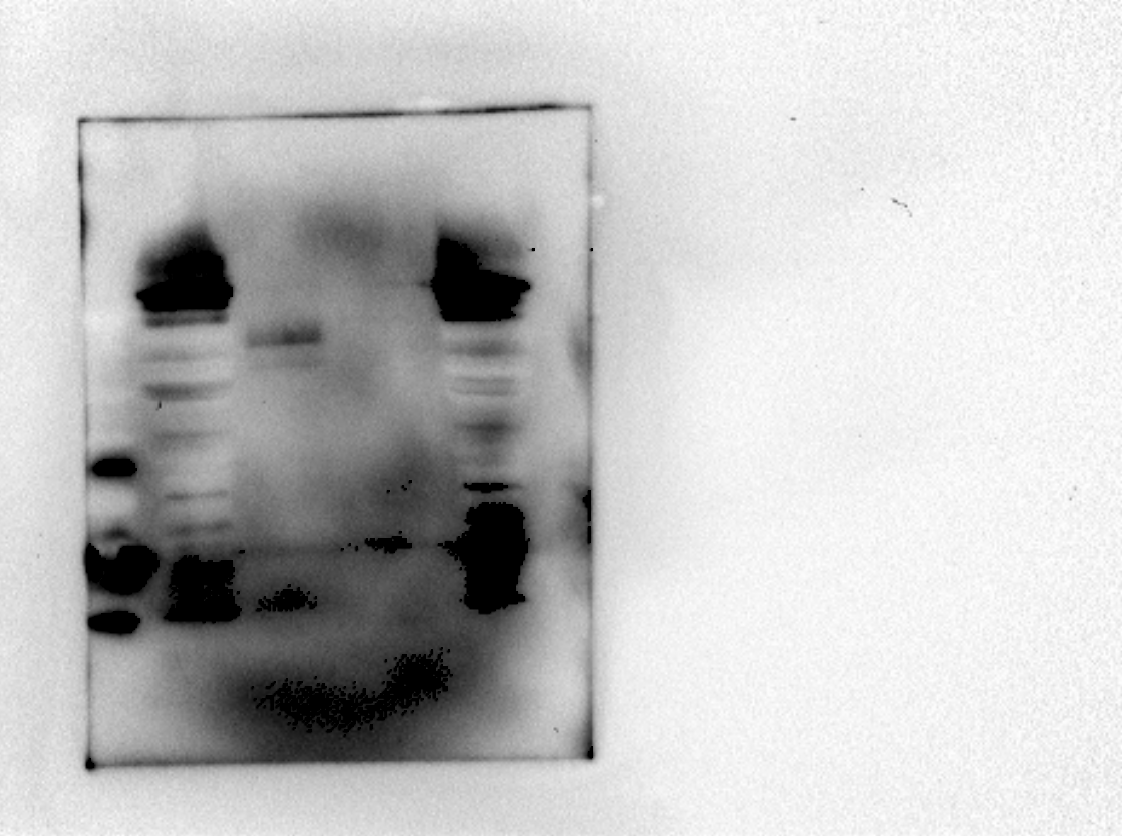

Supplement: Figure 5—source data 1. [file elife-91666-fig5-data1.zip › Figure 5-Figure supplement 4-source data 1/HA-unedited.tif]

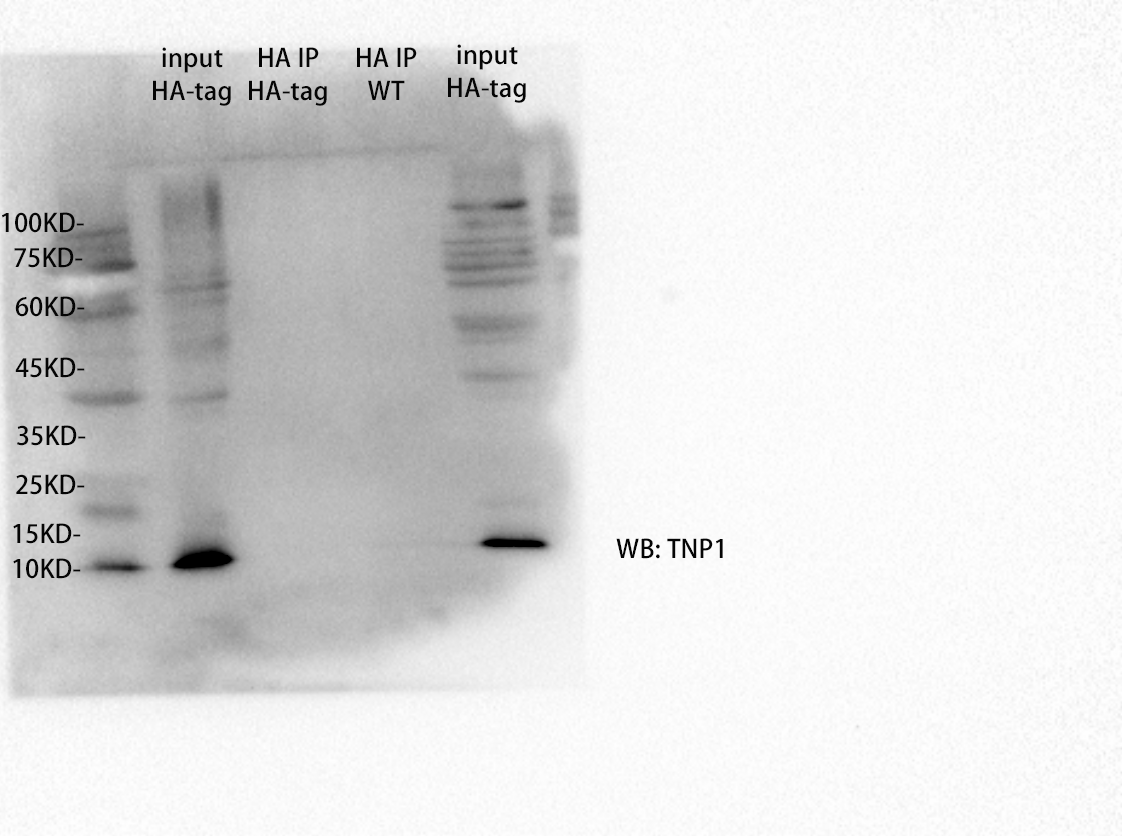

Supplement: Figure 5—source data 1. [file elife-91666-fig5-data1.zip › Figure 5-Figure supplement 4-source data 1/TNP1-labelled.tif]

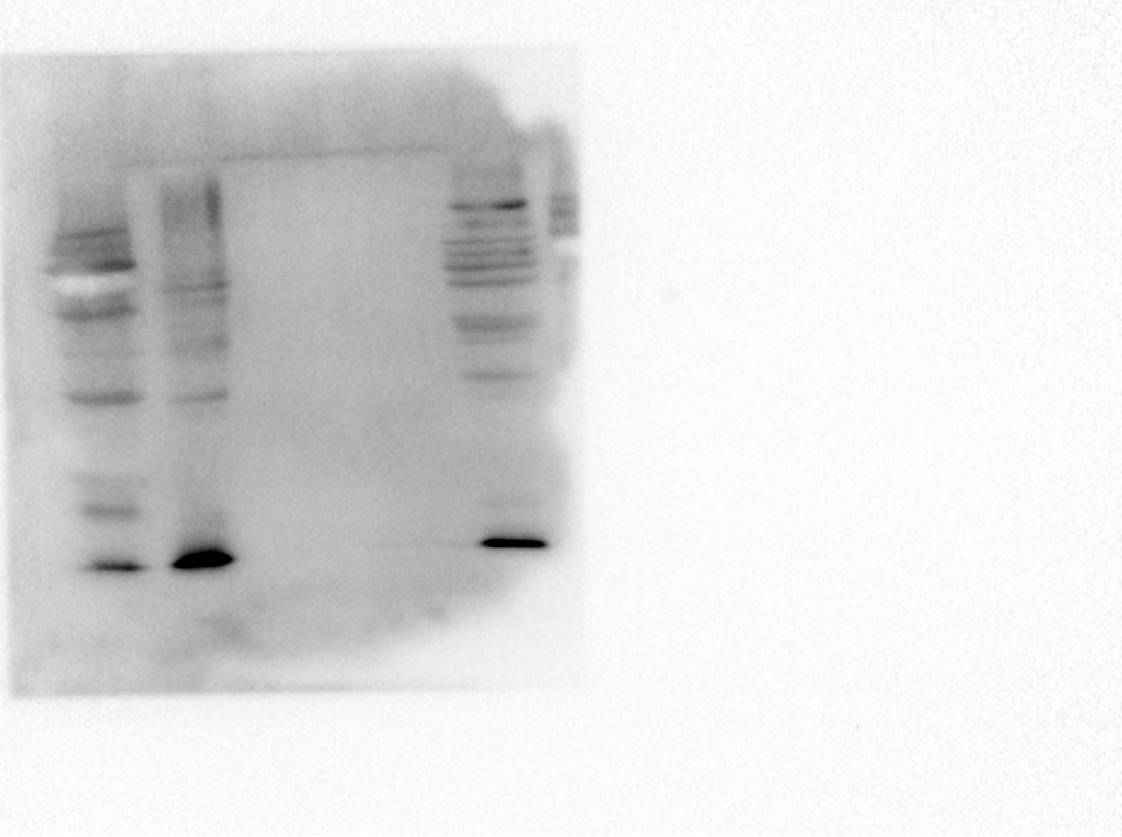

Supplement: Figure 5—source data 1. [file elife-91666-fig5-data1.zip › Figure 5-Figure supplement 4-source data 1/TNP1-unedited.tif]

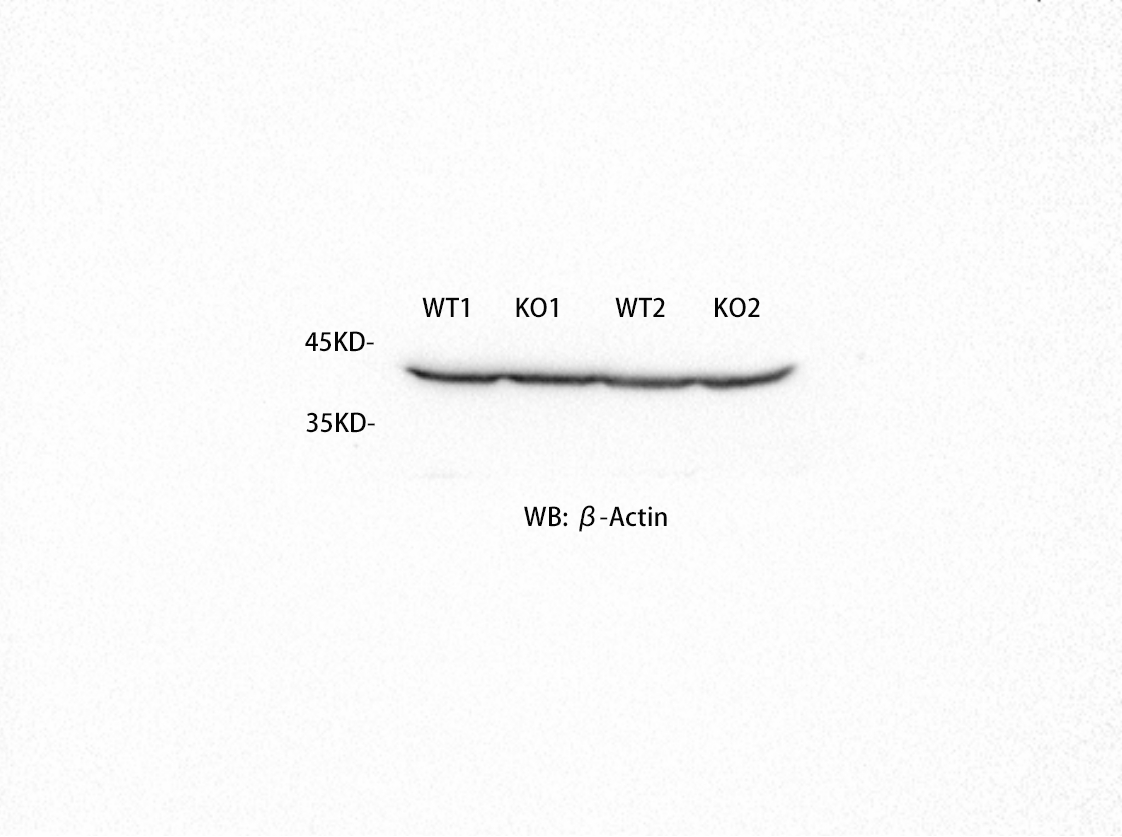

Supplement: Figure 7—source data 1. [file elife-91666-fig7-data1.zip › Figure 7-source data 1/Actin-labelled.tif]

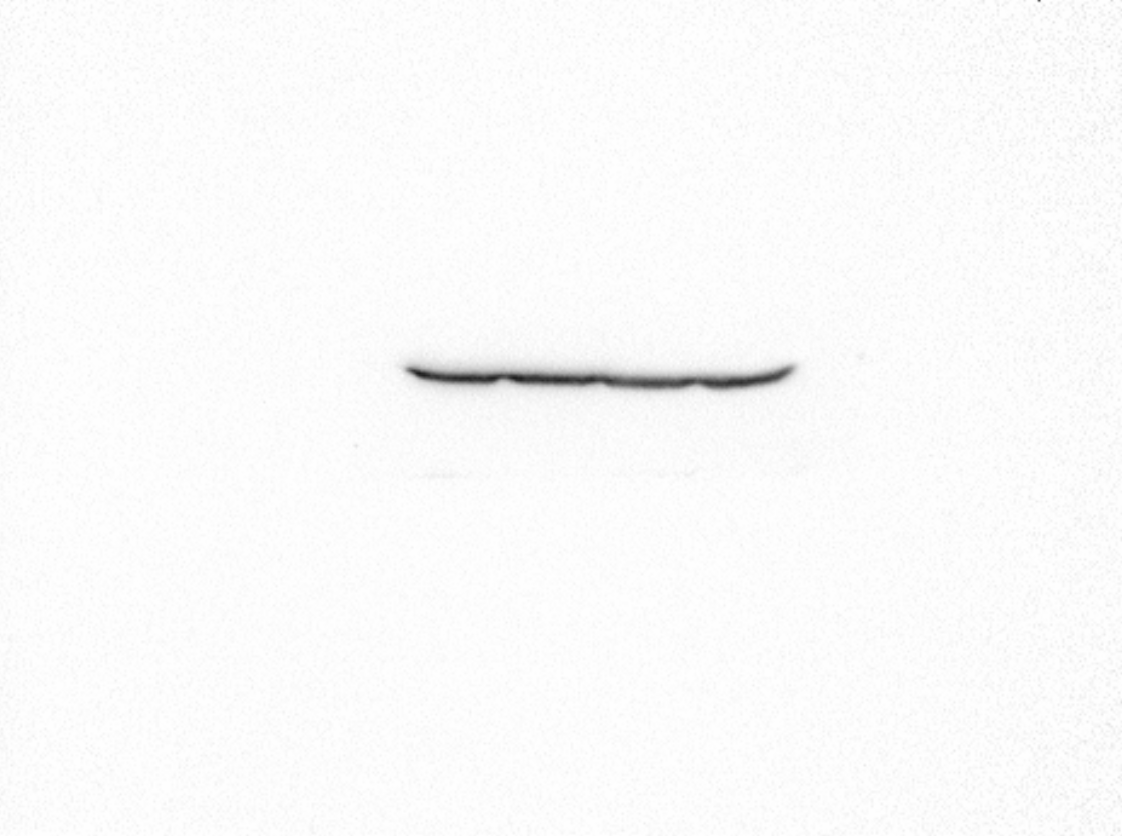

Supplement: Figure 7—source data 1. [file elife-91666-fig7-data1.zip › Figure 7-source data 1/Actin-unedited.tif]

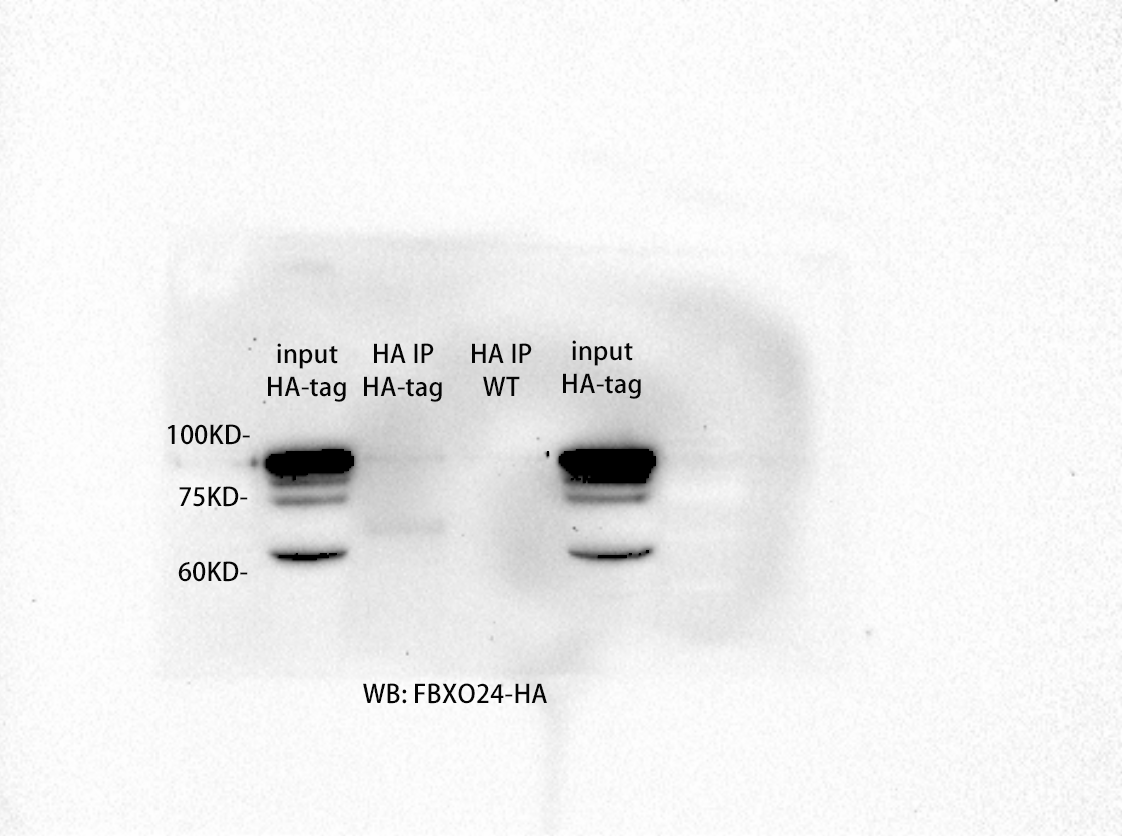

Supplement: Figure 7—source data 1. [file elife-91666-fig7-data1.zip › Figure 7-source data 1/HA-labelled.tif]

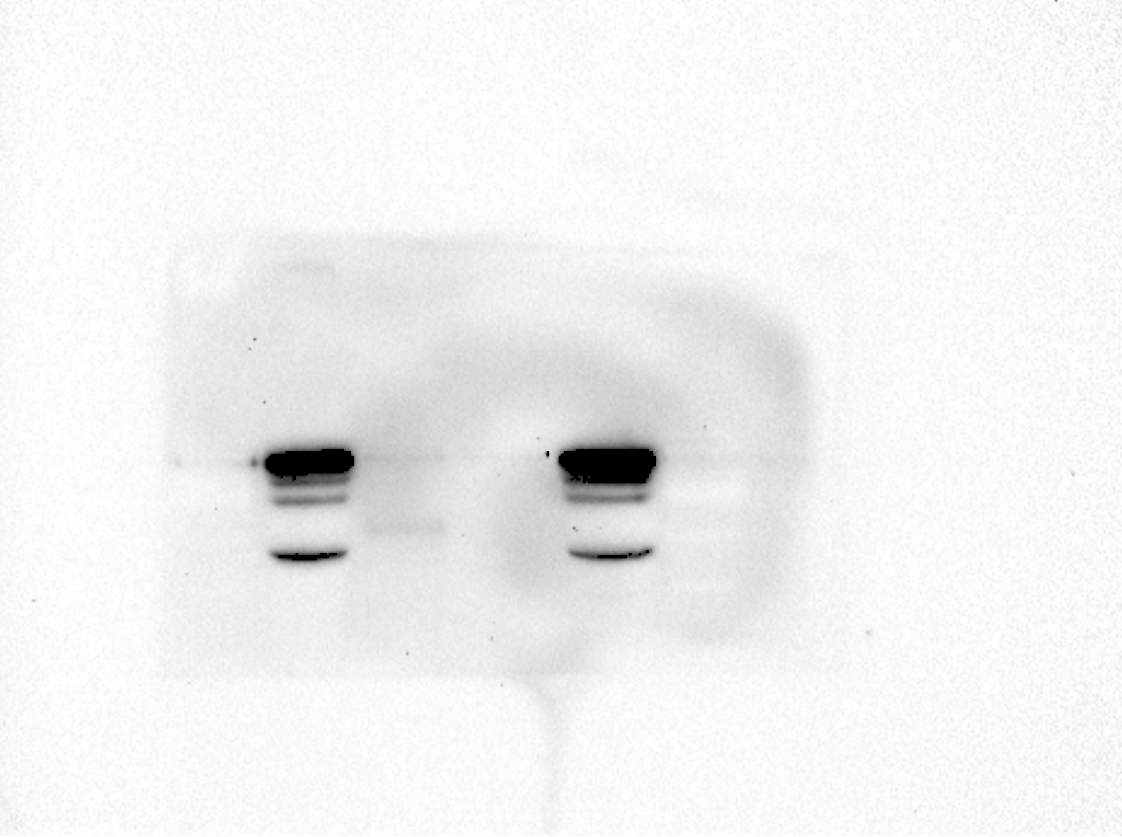

Supplement: Figure 7—source data 1. [file elife-91666-fig7-data1.zip › Figure 7-source data 1/HA-unedited.tif]

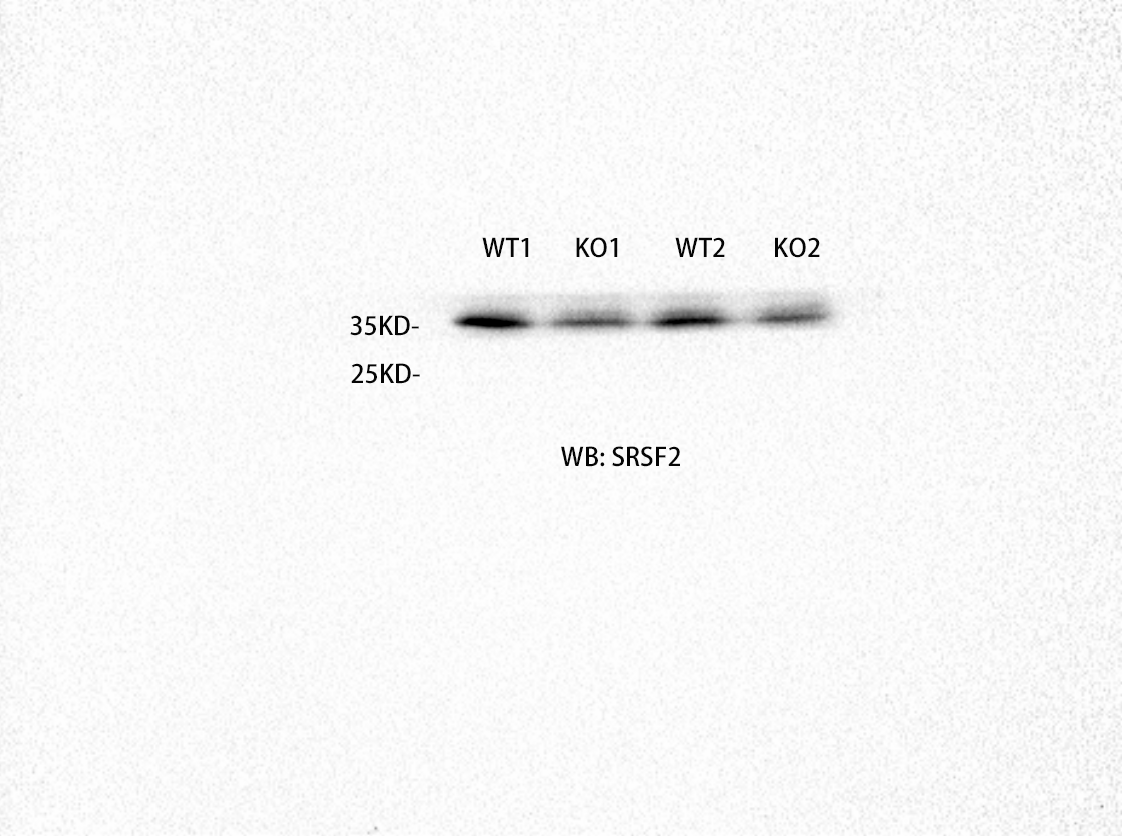

Supplement: Figure 7—source data 1. [file elife-91666-fig7-data1.zip › Figure 7-source data 1/SRSF2(2)-lablelled.tif]

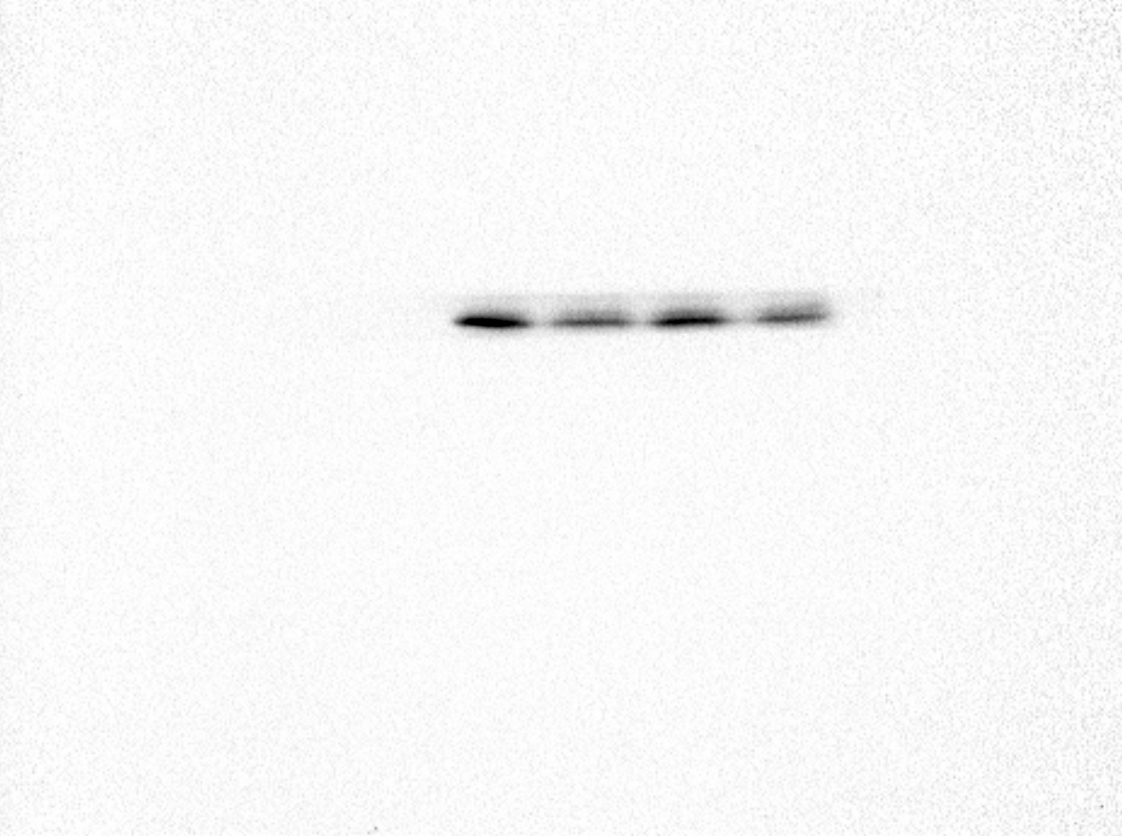

Supplement: Figure 7—source data 1. [file elife-91666-fig7-data1.zip › Figure 7-source data 1/SRSF2(2)-unedited.tif]

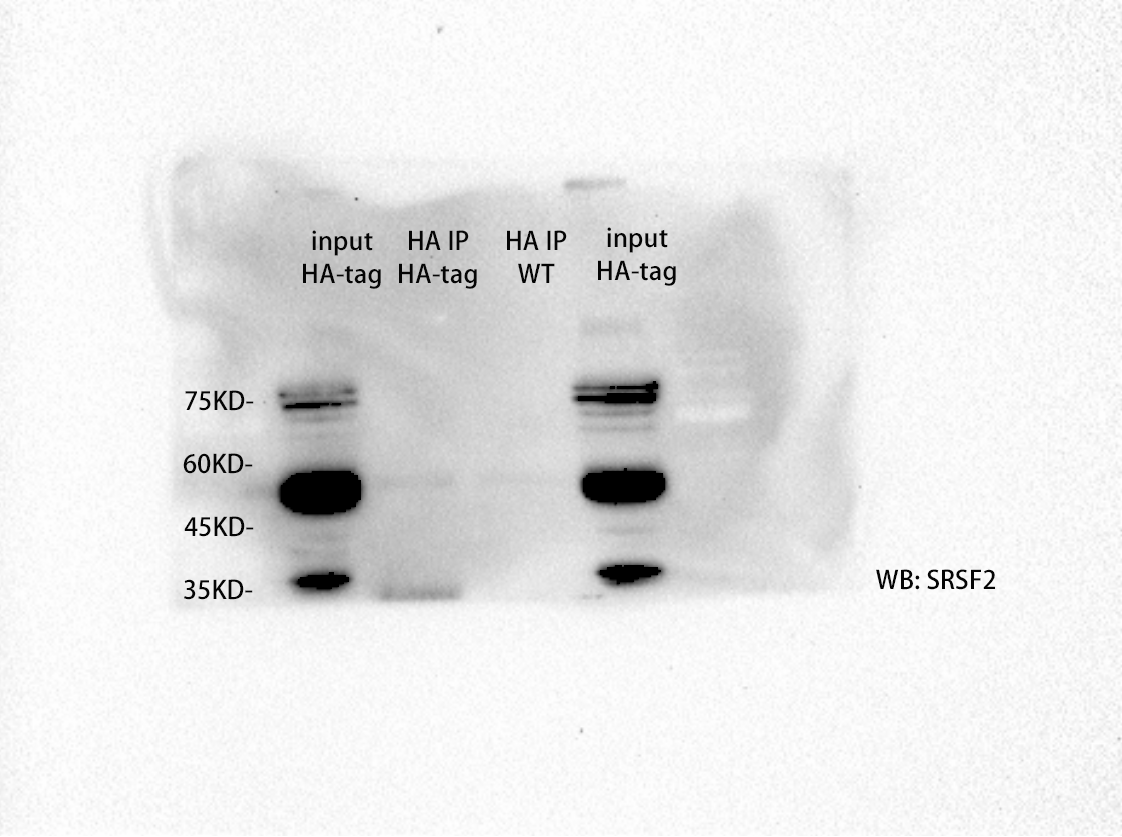

Supplement: Figure 7—source data 1. [file elife-91666-fig7-data1.zip › Figure 7-source data 1/SRSF2-labelled4.tif]

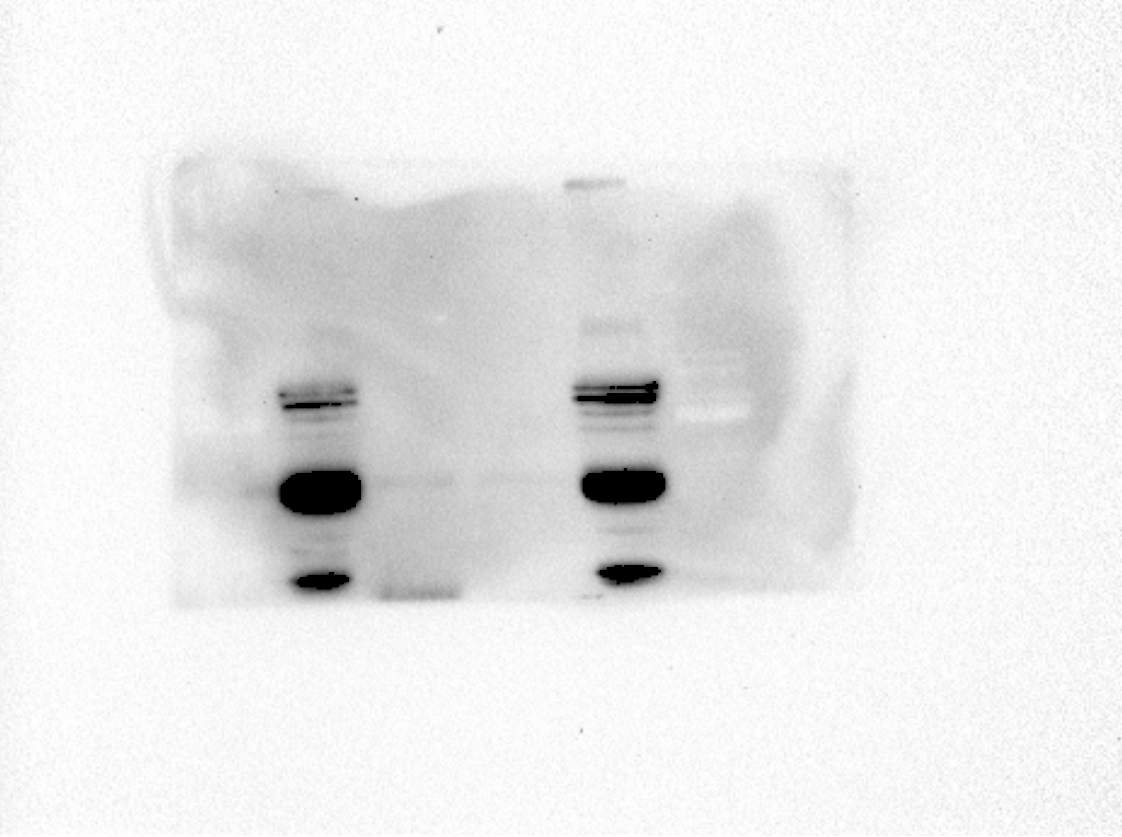

Supplement: Figure 7—source data 1. [file elife-91666-fig7-data1.zip › Figure 7-source data 1/SRSF2-unedited.tif]

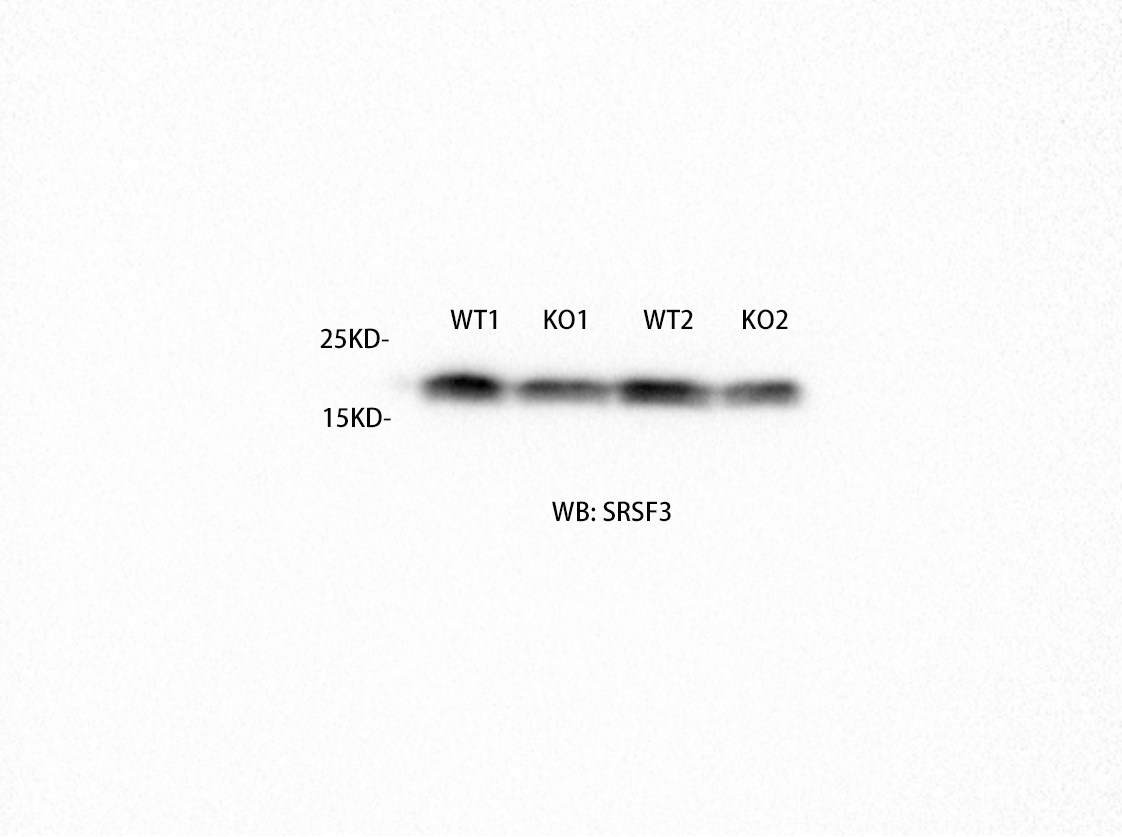

Supplement: Figure 7—source data 1. [file elife-91666-fig7-data1.zip › Figure 7-source data 1/SRSF3(2)-lablelled.tif]

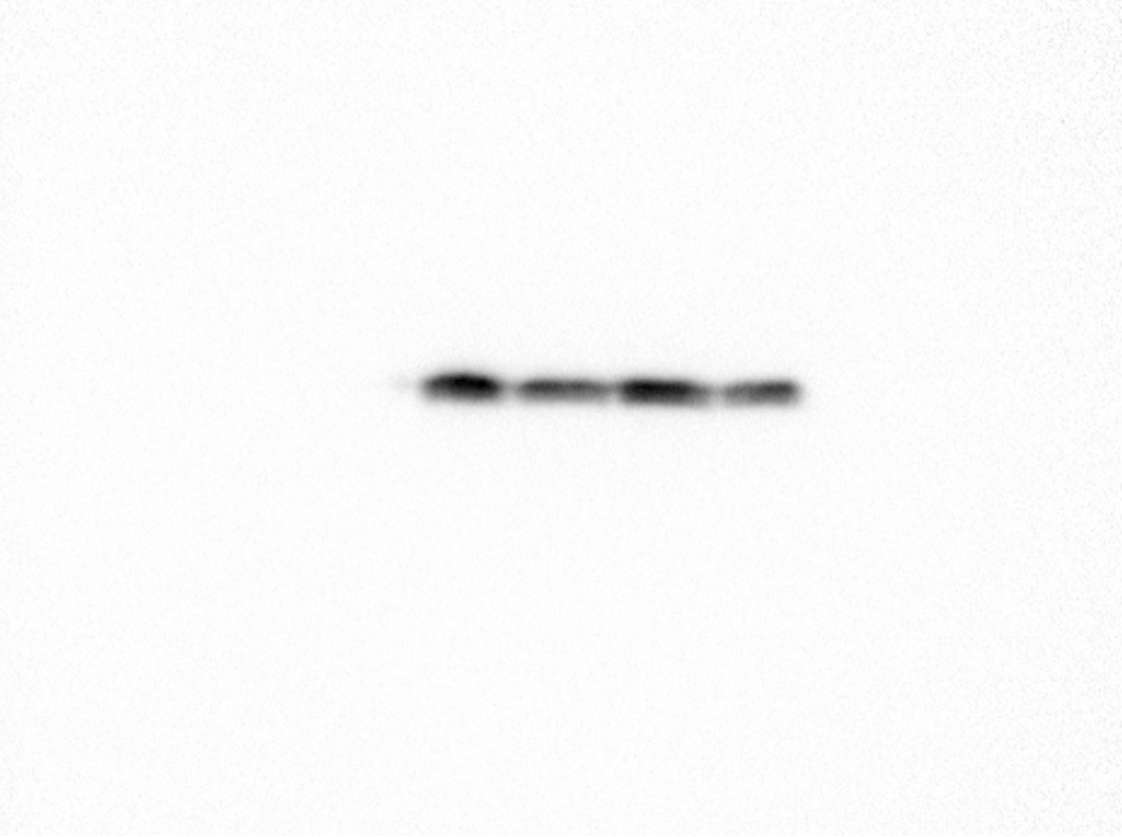

Supplement: Figure 7—source data 1. [file elife-91666-fig7-data1.zip › Figure 7-source data 1/SRSF3(2)-unedited.tif]

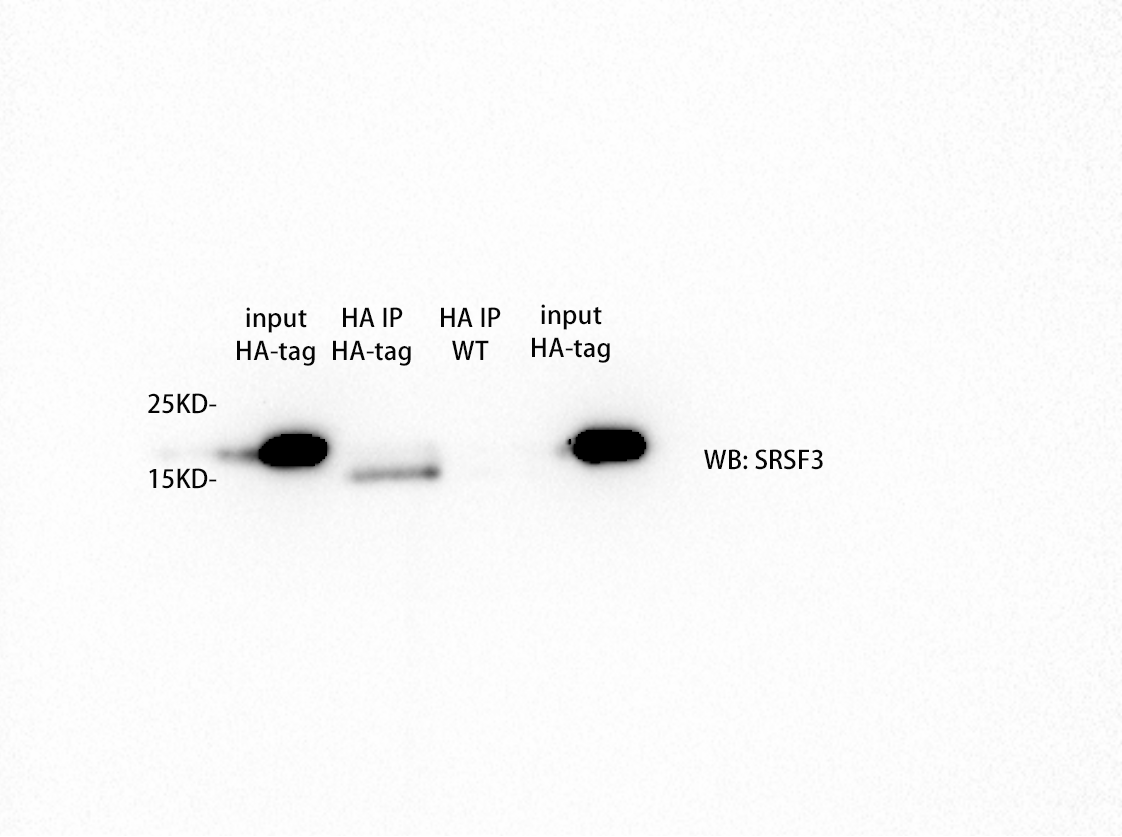

Supplement: Figure 7—source data 1. [file elife-91666-fig7-data1.zip › Figure 7-source data 1/SRSF3-labelled.tif]

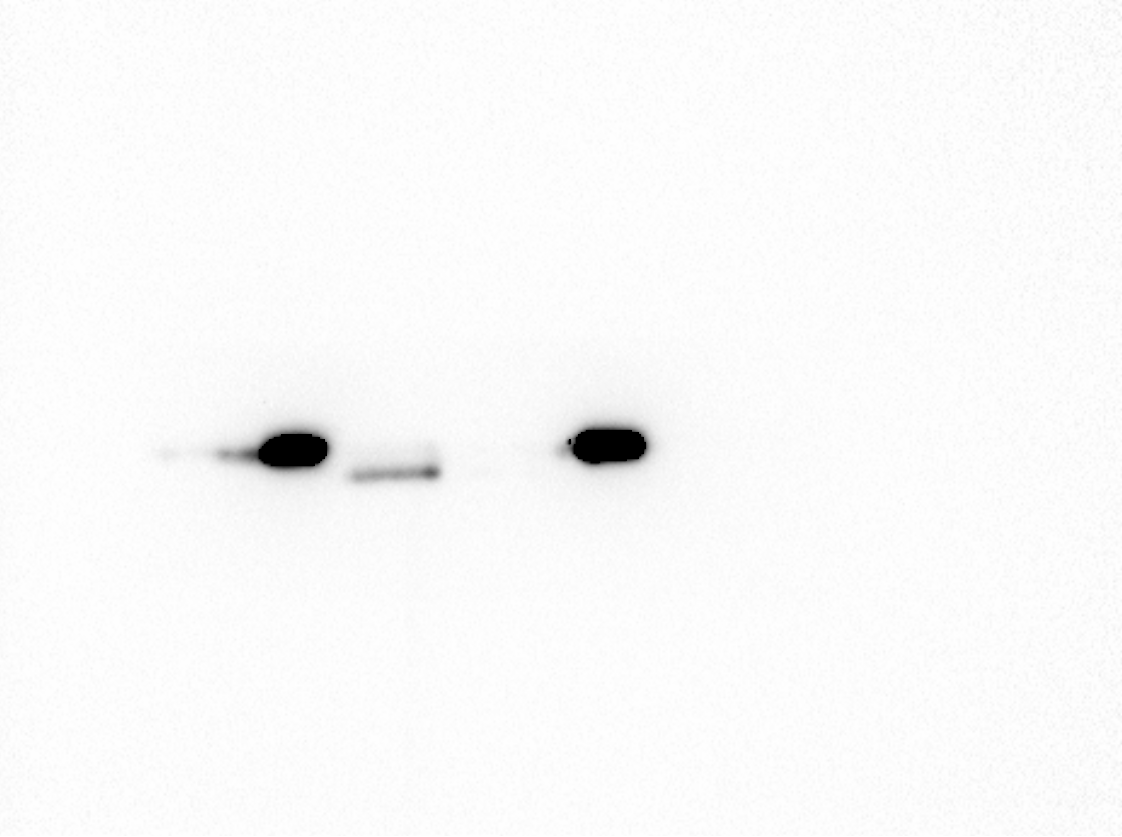

Supplement: Figure 7—source data 1. [file elife-91666-fig7-data1.zip › Figure 7-source data 1/SRSF3-unedited.tif]

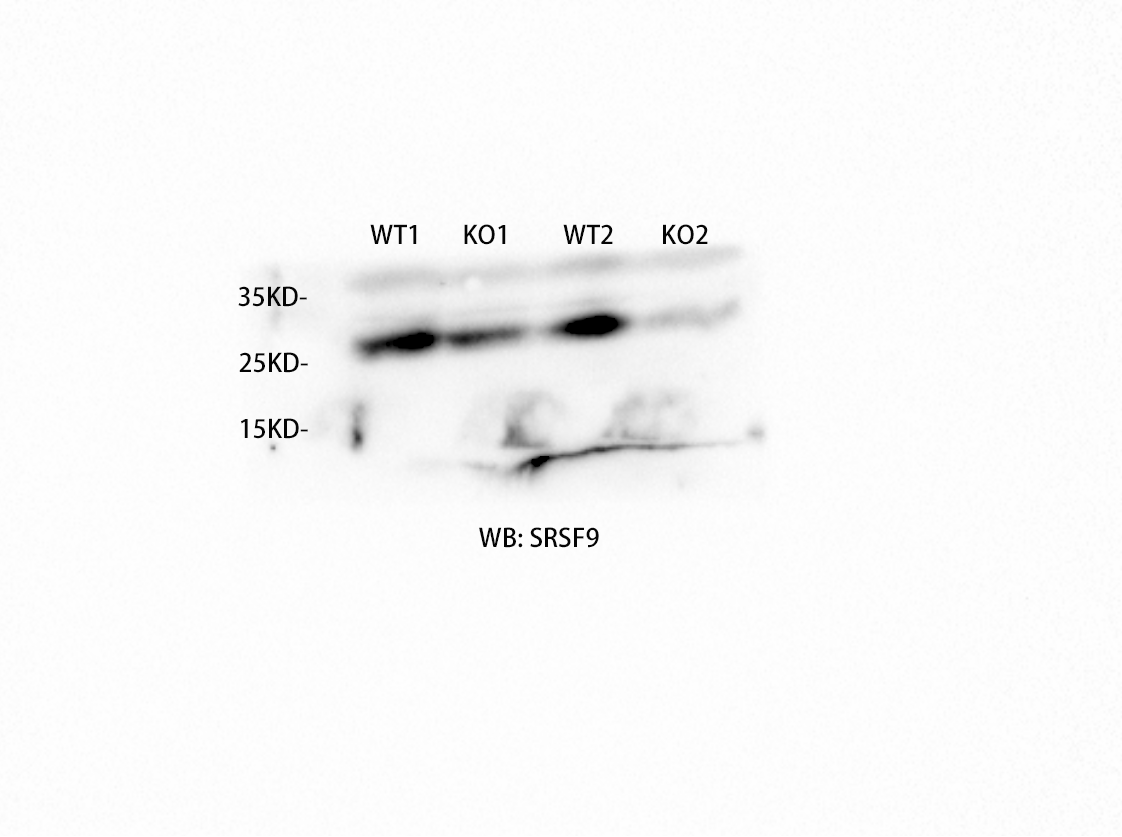

Supplement: Figure 7—source data 1. [file elife-91666-fig7-data1.zip › Figure 7-source data 1/SRSF9(2)-lablelled.tif]

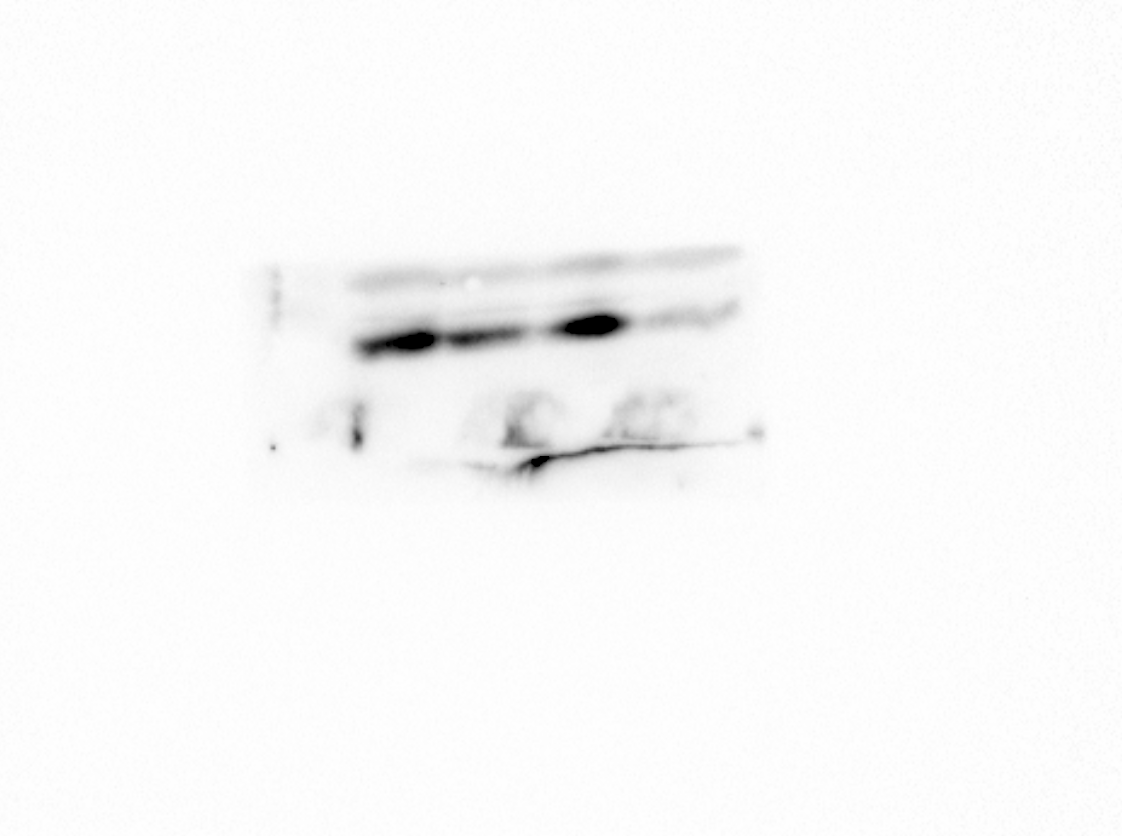

Supplement: Figure 7—source data 1. [file elife-91666-fig7-data1.zip › Figure 7-source data 1/SRSF9(2)-unedited.tif]

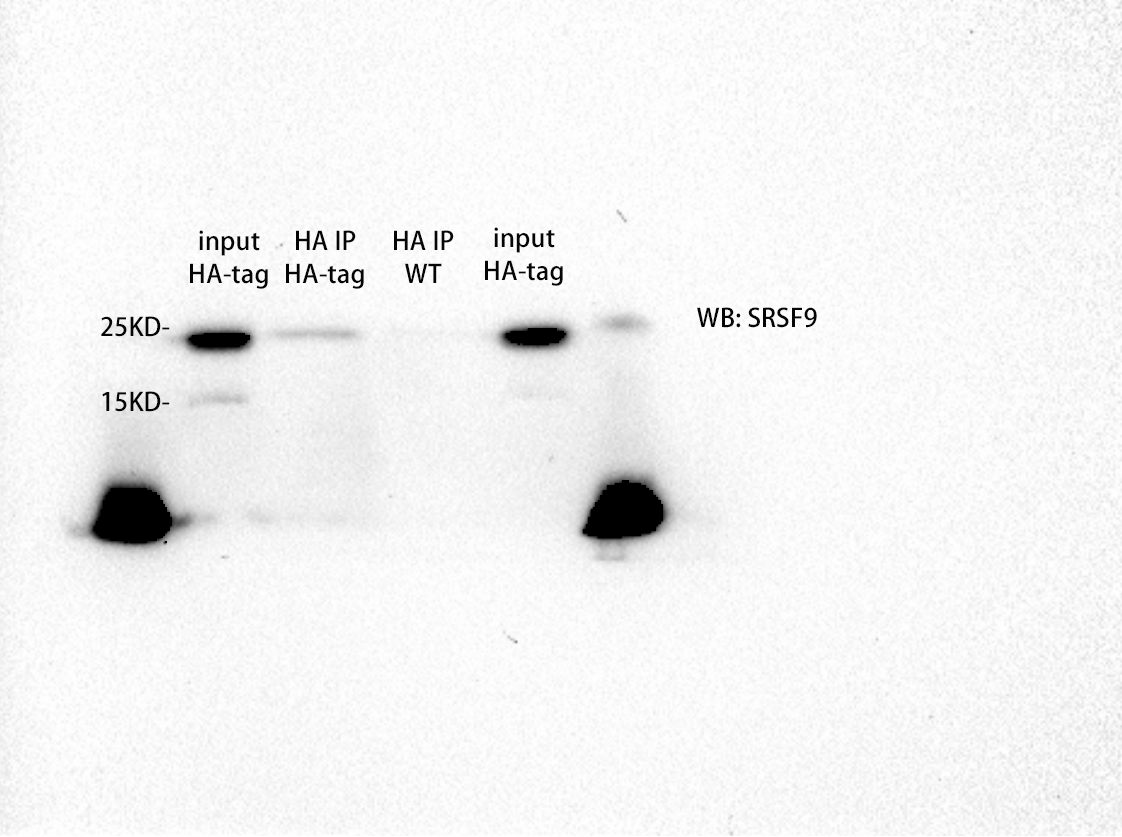

Supplement: Figure 7—source data 1. [file elife-91666-fig7-data1.zip › Figure 7-source data 1/SRSF9-labelled.tif]

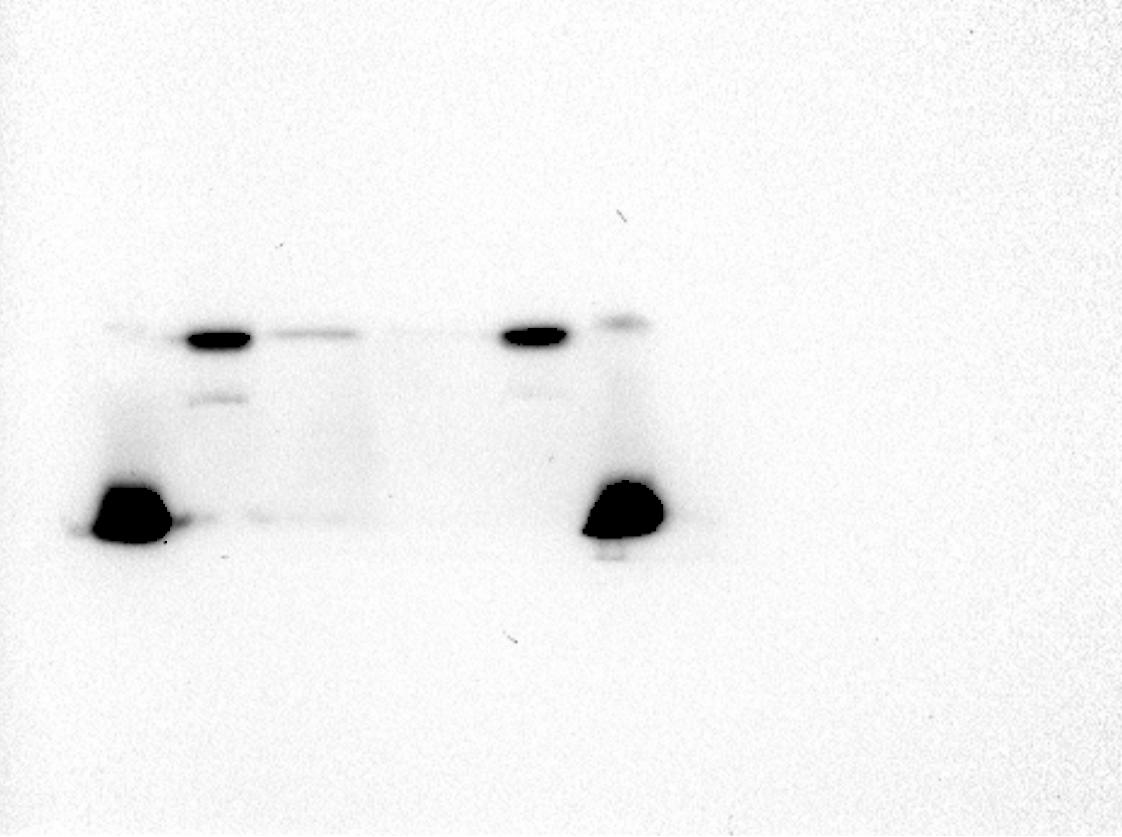

Supplement: Figure 7—source data 1. [file elife-91666-fig7-data1.zip › Figure 7-source data 1/SRSF9-unedited.tif]

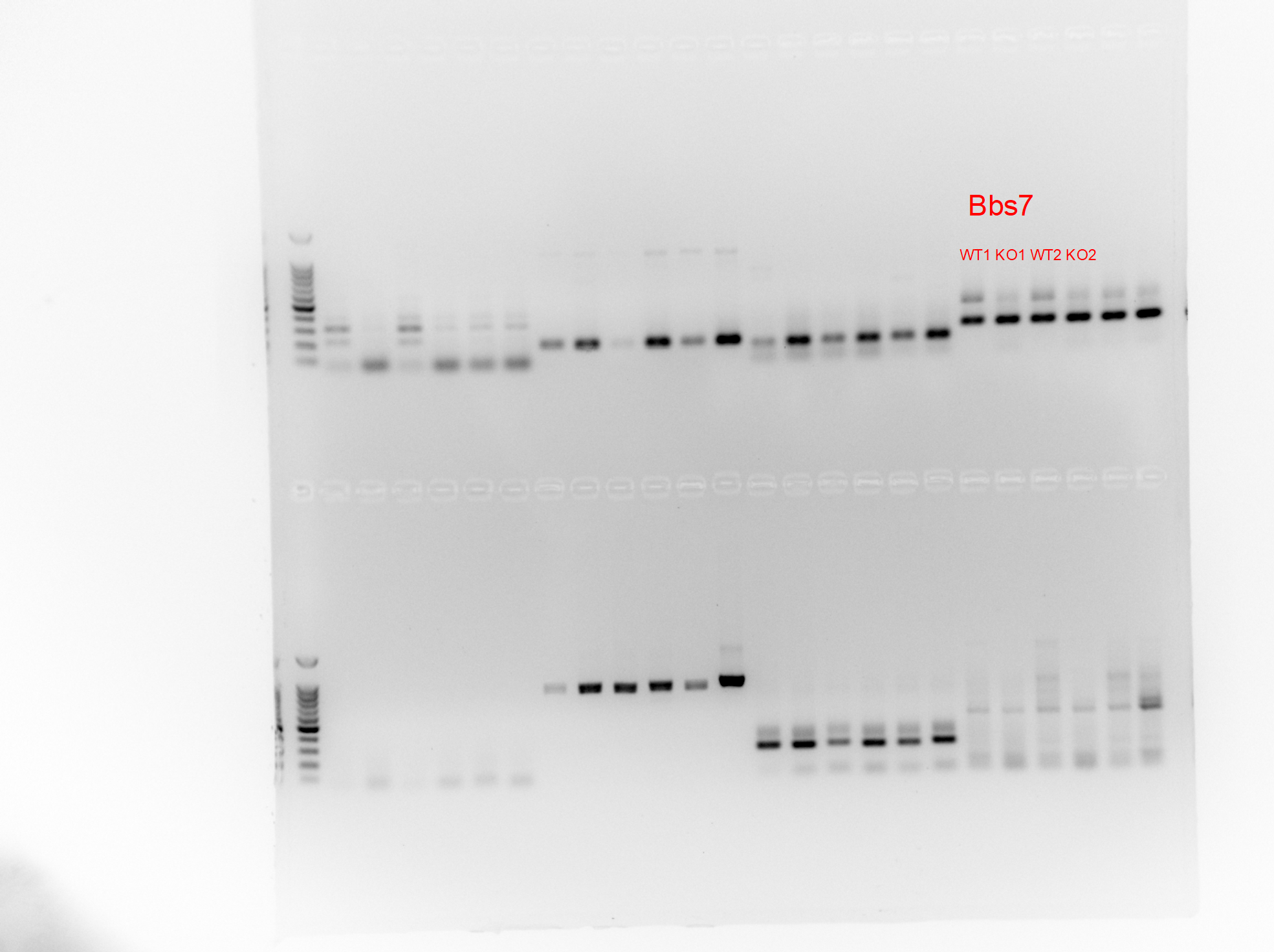

Supplement: Figure 7—source data 2. [file elife-91666-fig7-data2.zip › Figure 7-source data 2/Bbs7-labelled.tif]

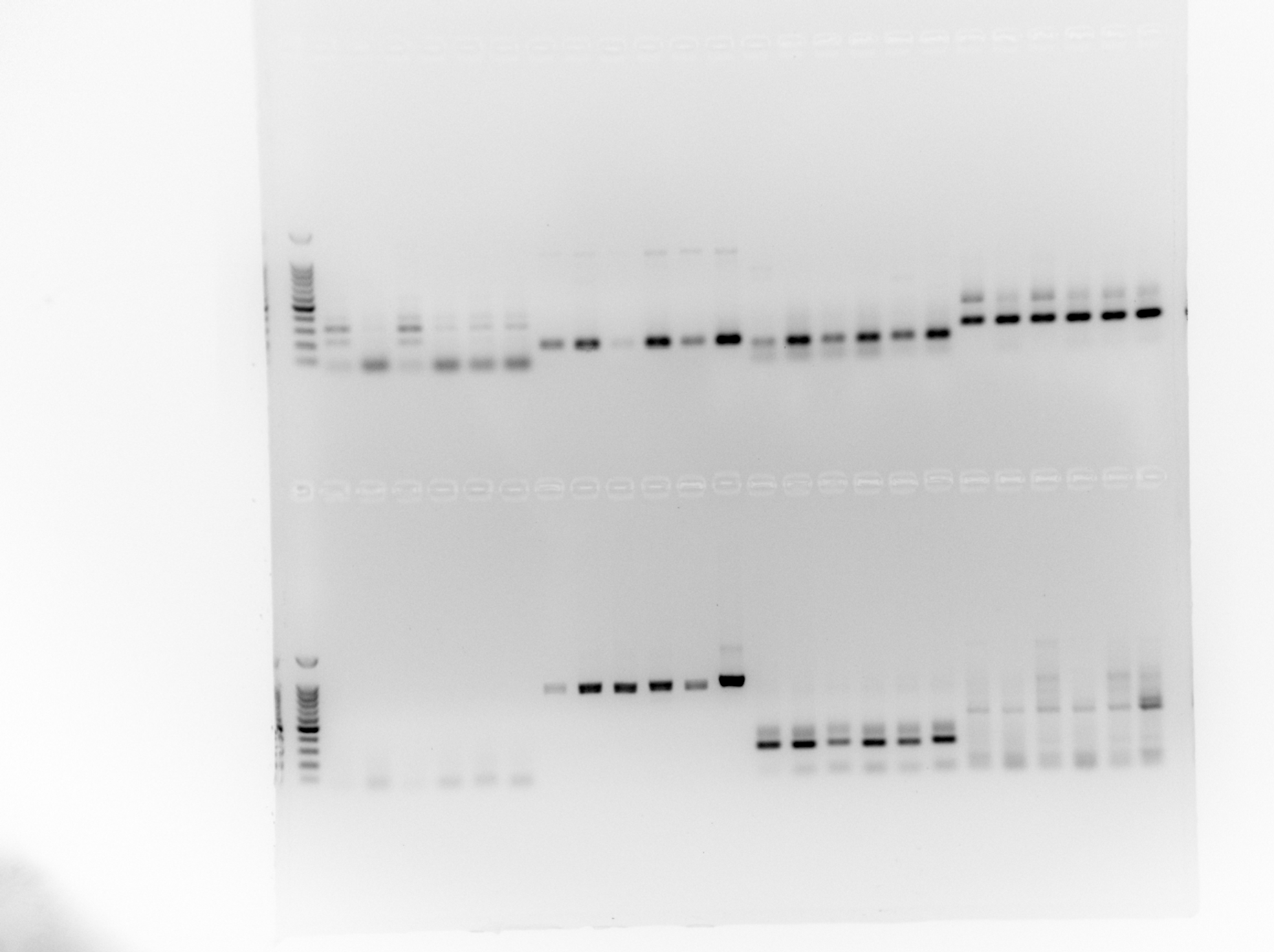

Supplement: Figure 7—source data 2. [file elife-91666-fig7-data2.zip › Figure 7-source data 2/Bbs7-unedited. tif.tif]

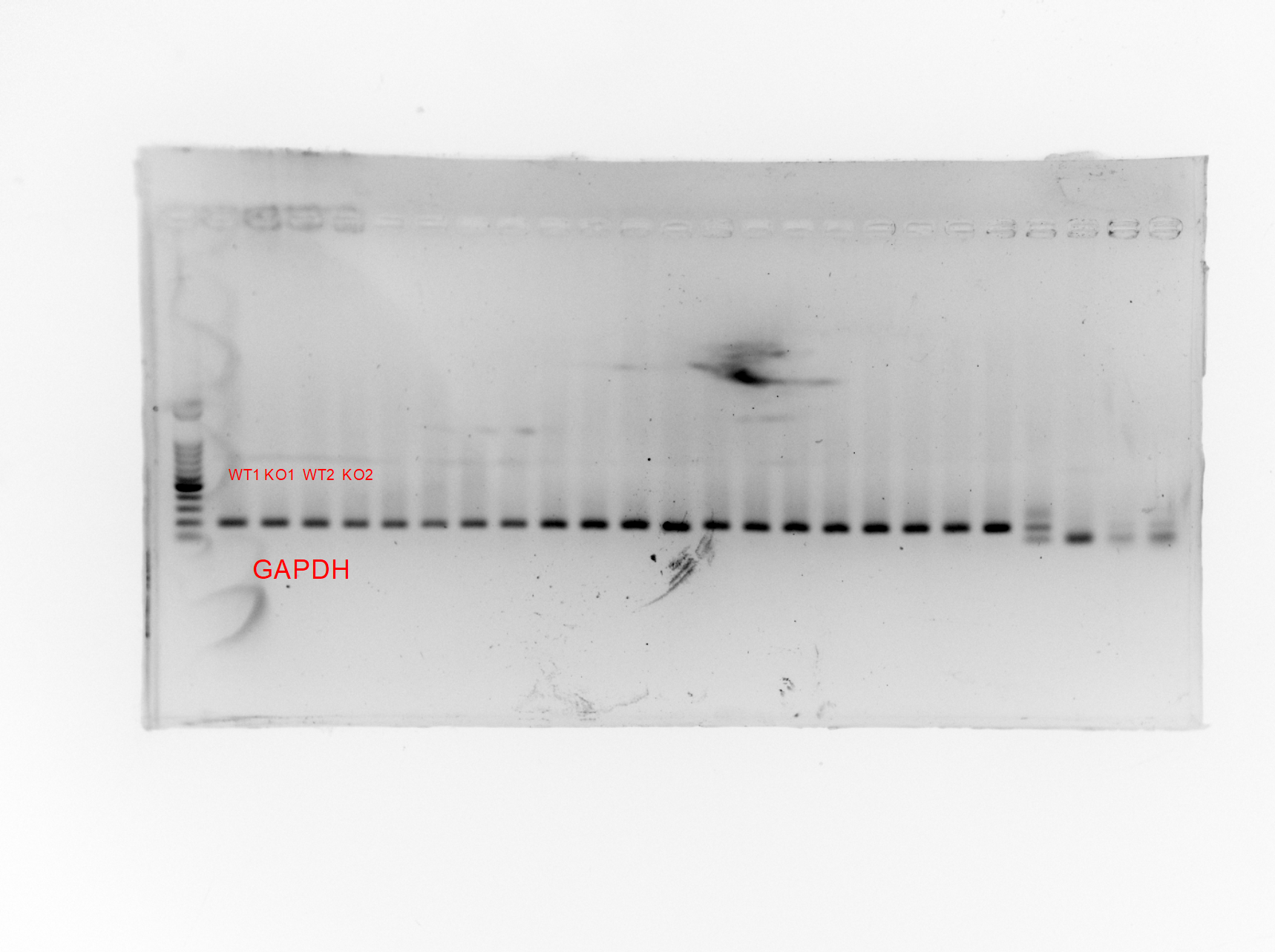

Supplement: Figure 7—source data 2. [file elife-91666-fig7-data2.zip › Figure 7-source data 2/GAPDH(1)-labelled.tif]

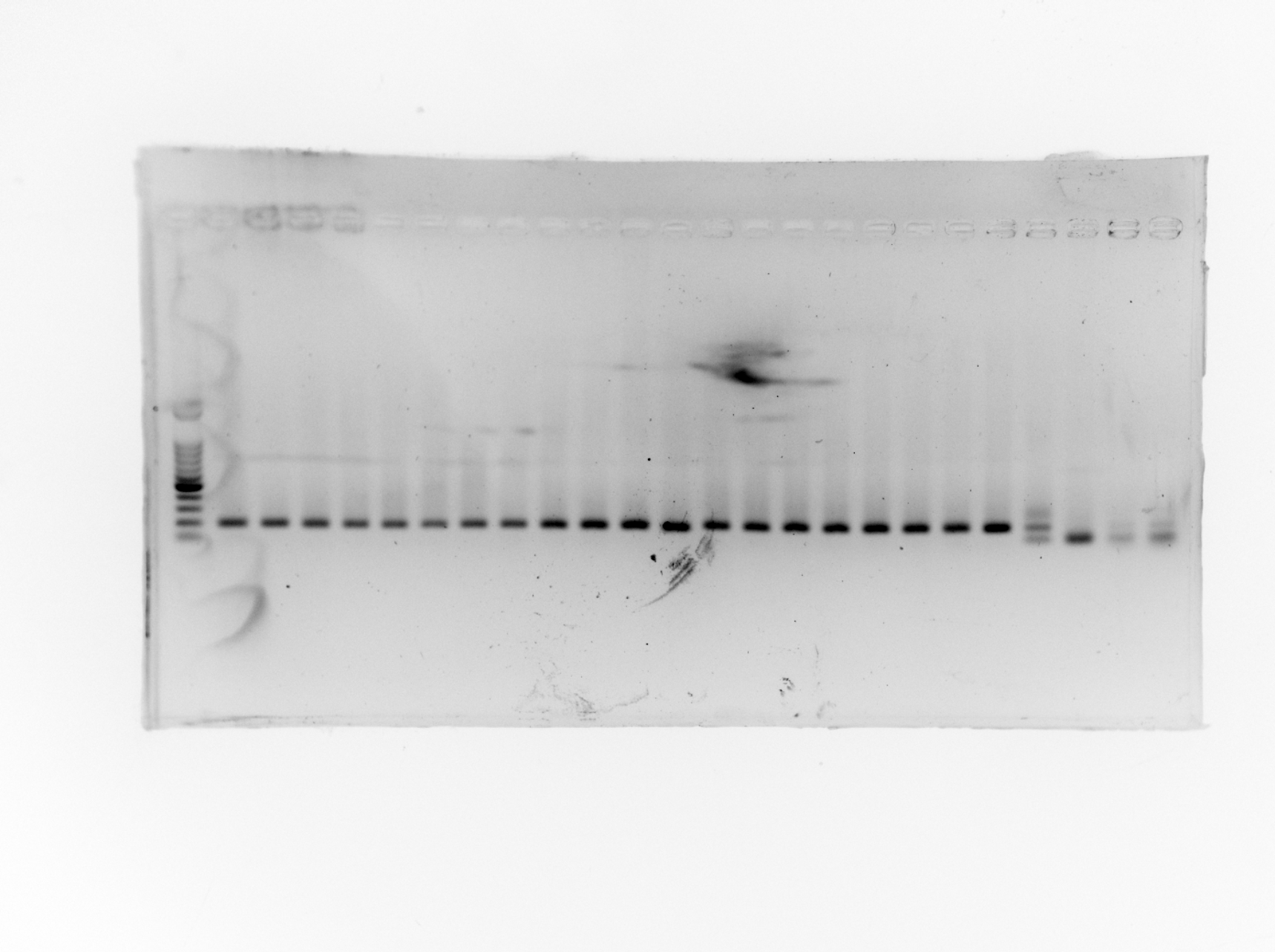

Supplement: Figure 7—source data 2. [file elife-91666-fig7-data2.zip › Figure 7-source data 2/GAPDH(1)-unedited.tif]

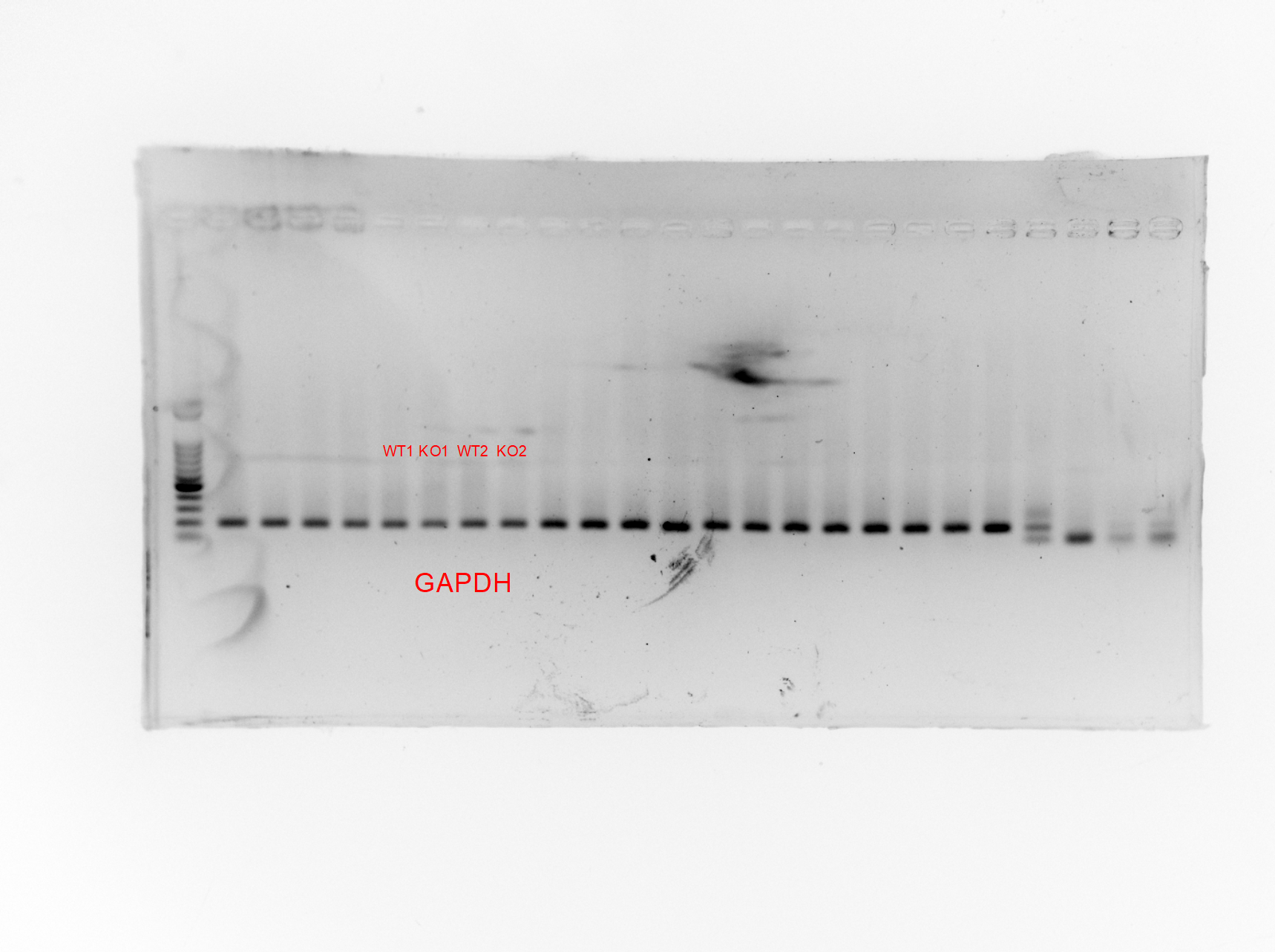

Supplement: Figure 7—source data 2. [file elife-91666-fig7-data2.zip › Figure 7-source data 2/GAPDH(2)-labelled.tif]

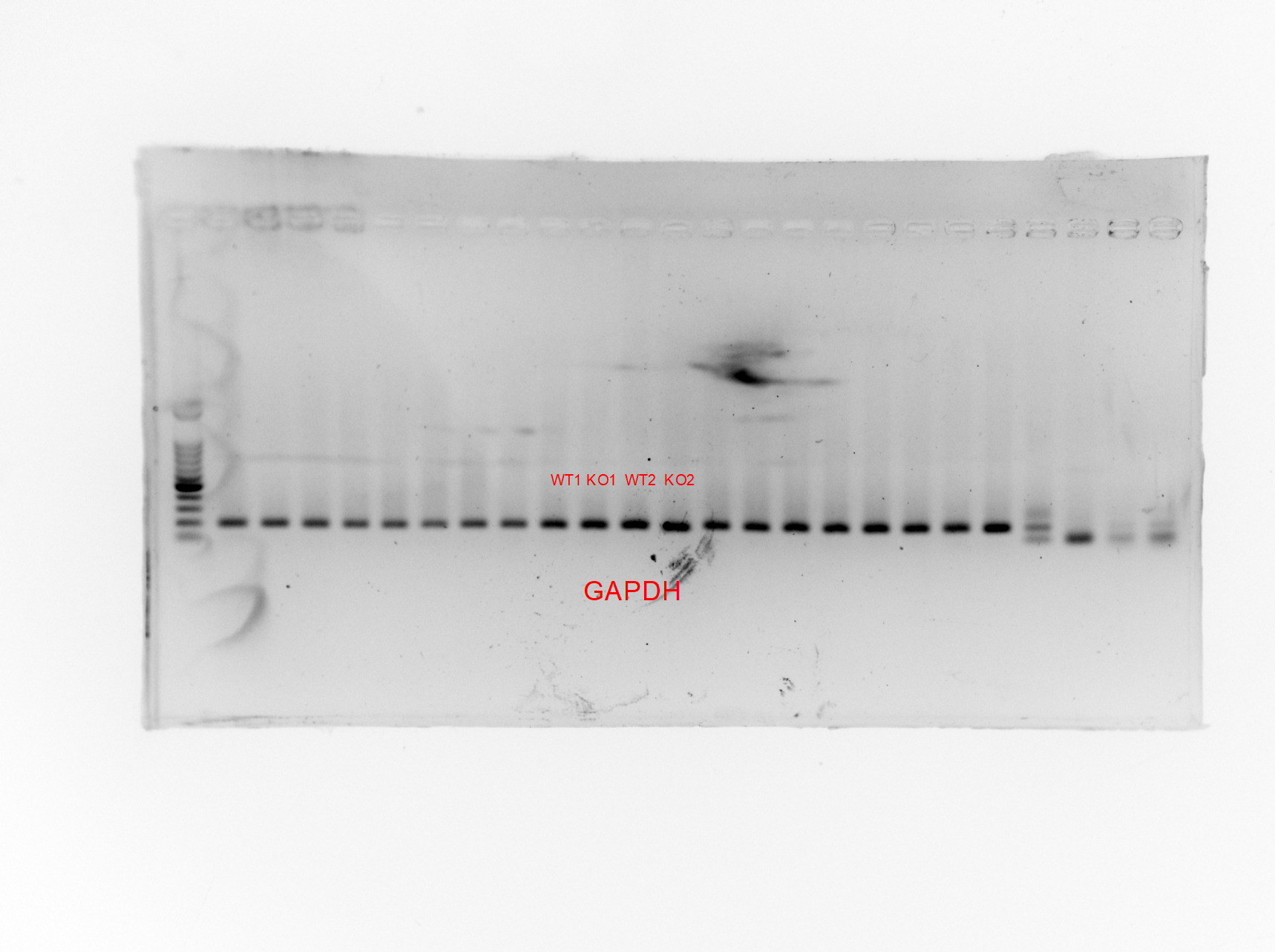

Supplement: Figure 7—source data 2. [file elife-91666-fig7-data2.zip › Figure 7-source data 2/GAPDH(3)-labelled.tif]

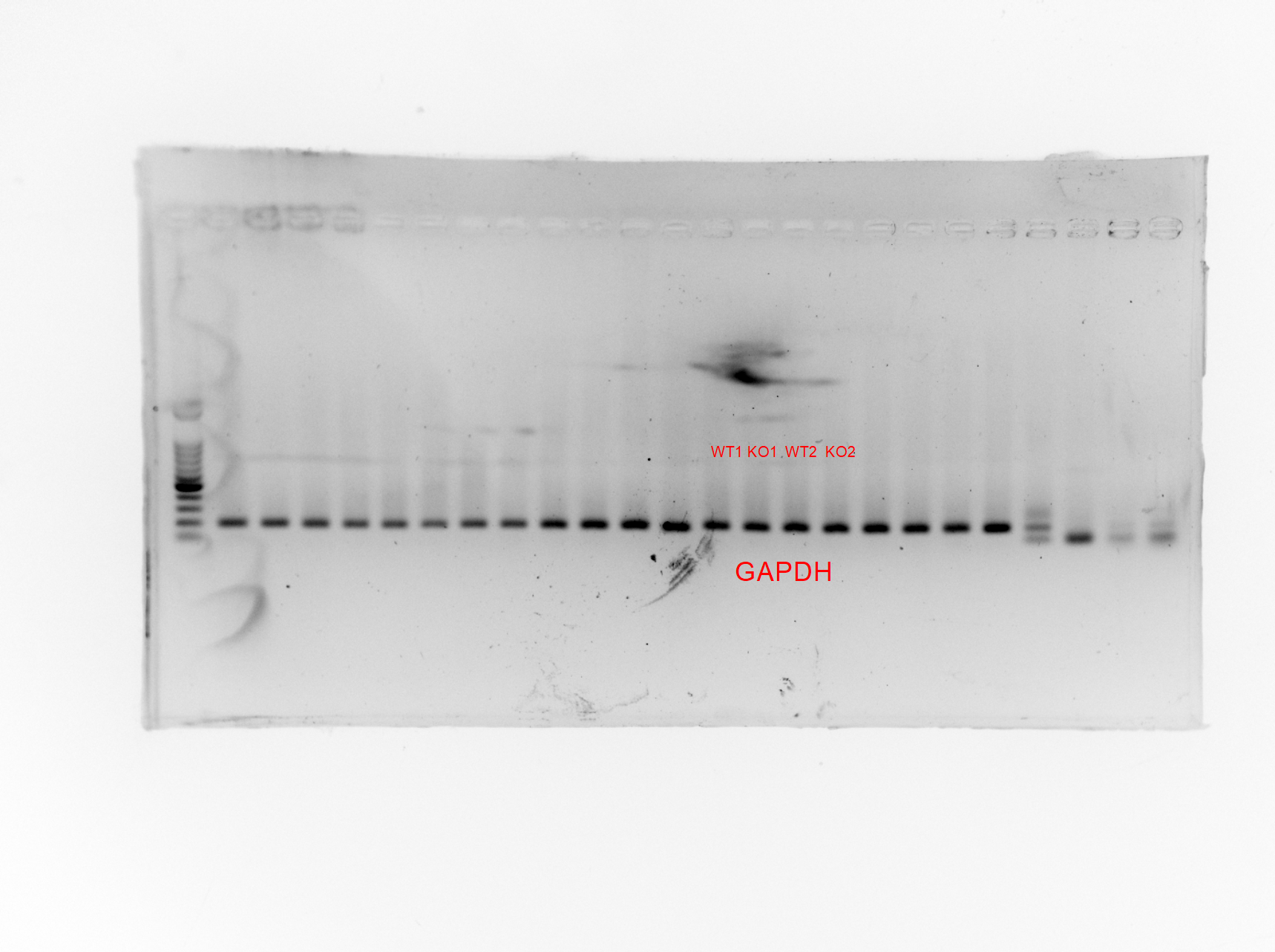

Supplement: Figure 7—source data 2. [file elife-91666-fig7-data2.zip › Figure 7-source data 2/GAPDH(4)-labelled.tif]

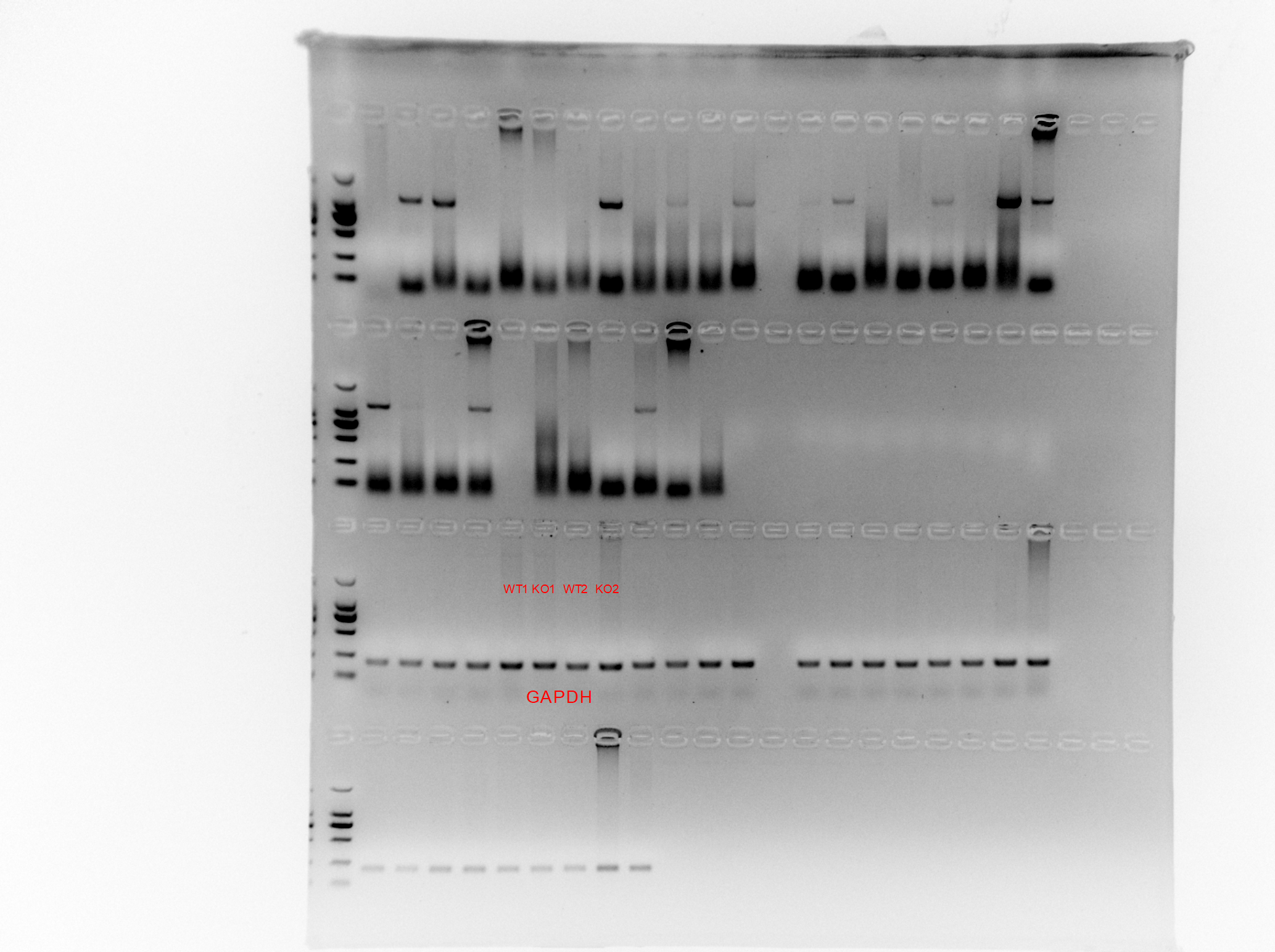

Supplement: Figure 7—source data 2. [file elife-91666-fig7-data2.zip › Figure 7-source data 2/GAPDH(5)-labelled.tif]

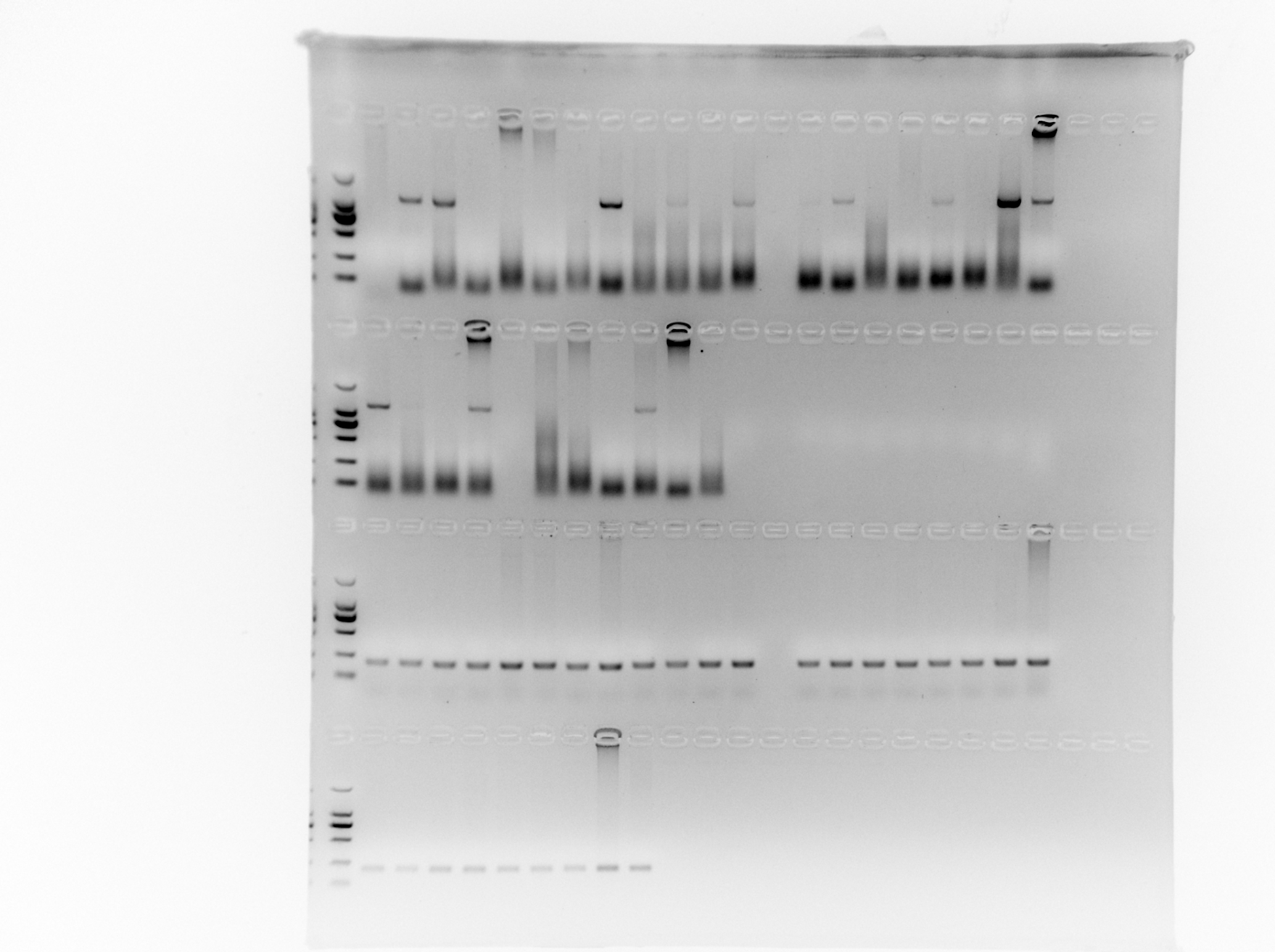

Supplement: Figure 7—source data 2. [file elife-91666-fig7-data2.zip › Figure 7-source data 2/GAPDH(5)-unedited.tif]

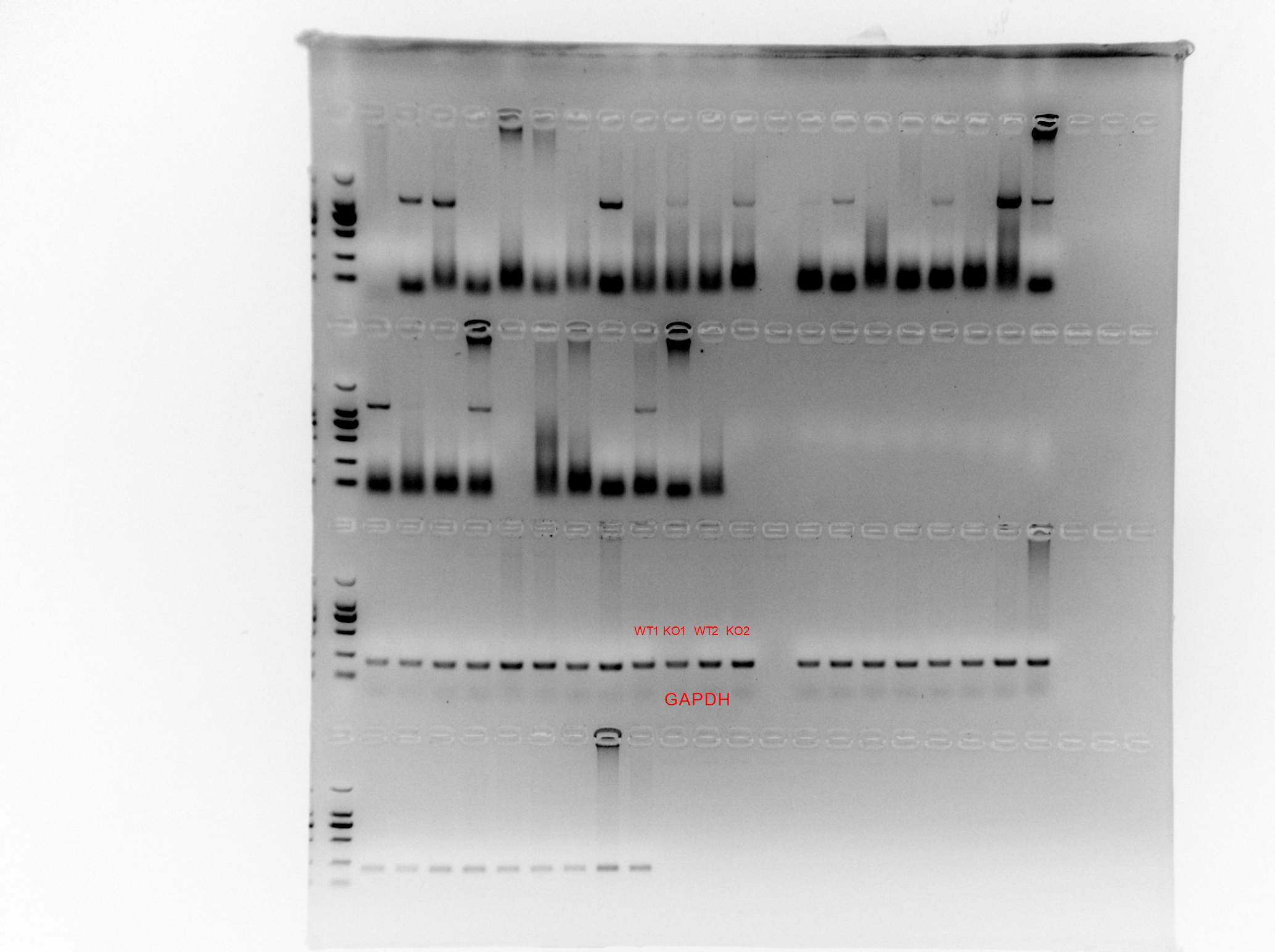

Supplement: Figure 7—source data 2. [file elife-91666-fig7-data2.zip › Figure 7-source data 2/GAPDH(6)-labelled.tif]

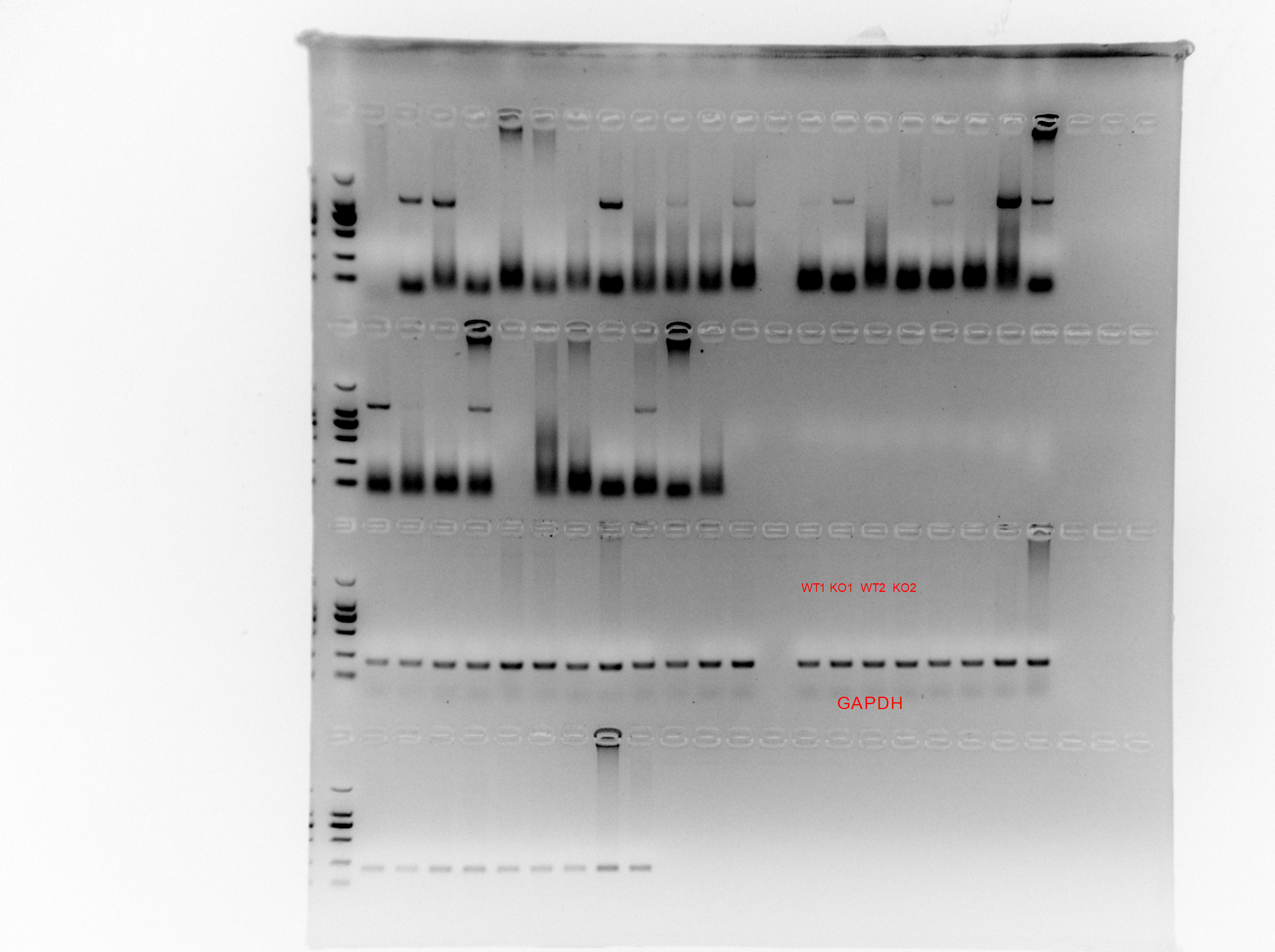

Supplement: Figure 7—source data 2. [file elife-91666-fig7-data2.zip › Figure 7-source data 2/GAPDH(7)-labelled.tif]

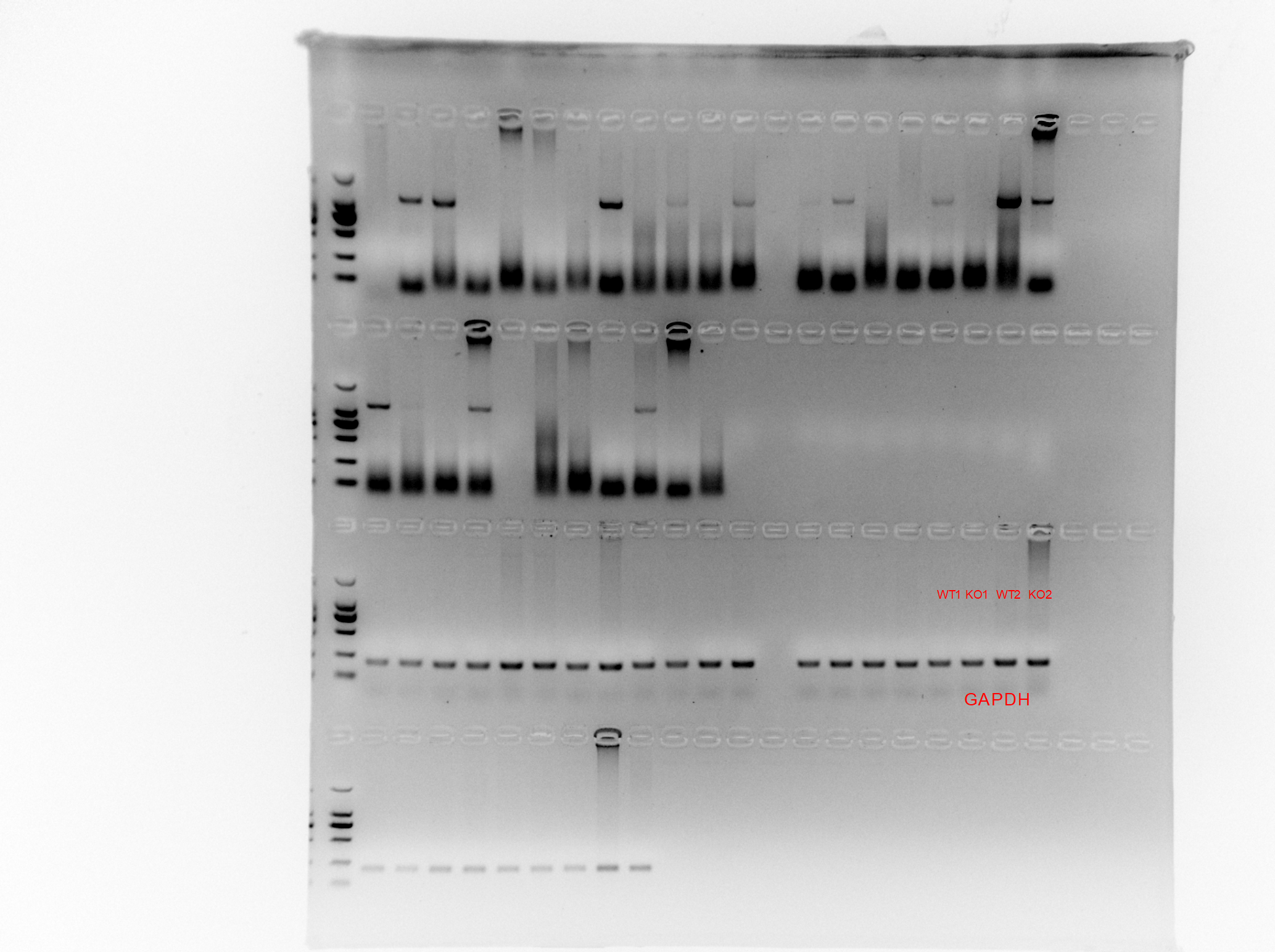

Supplement: Figure 7—source data 2. [file elife-91666-fig7-data2.zip › Figure 7-source data 2/GAPDH(8)-labelled.tif]

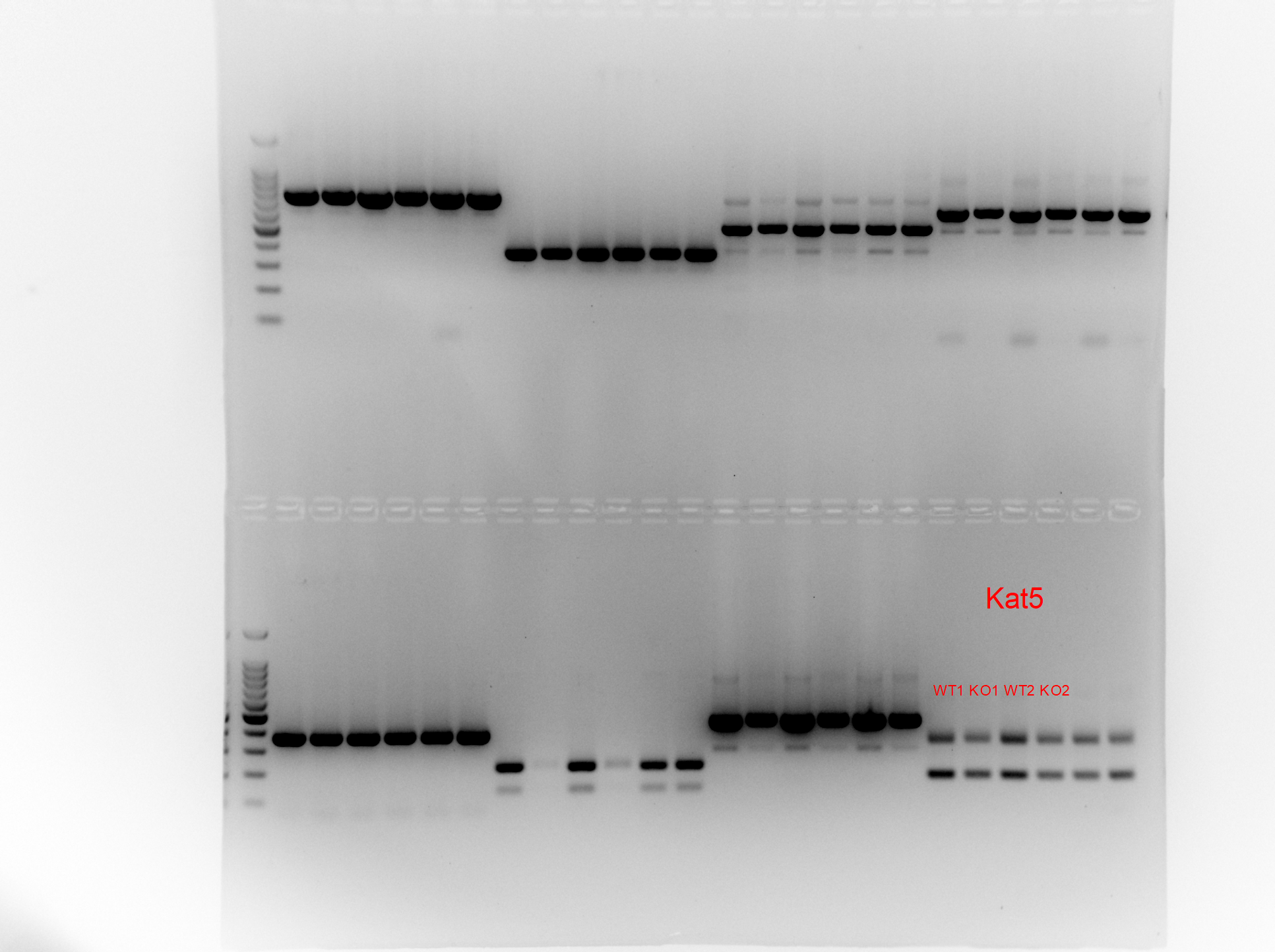

Supplement: Figure 7—source data 2. [file elife-91666-fig7-data2.zip › Figure 7-source data 2/Kat5-labelled.tif]

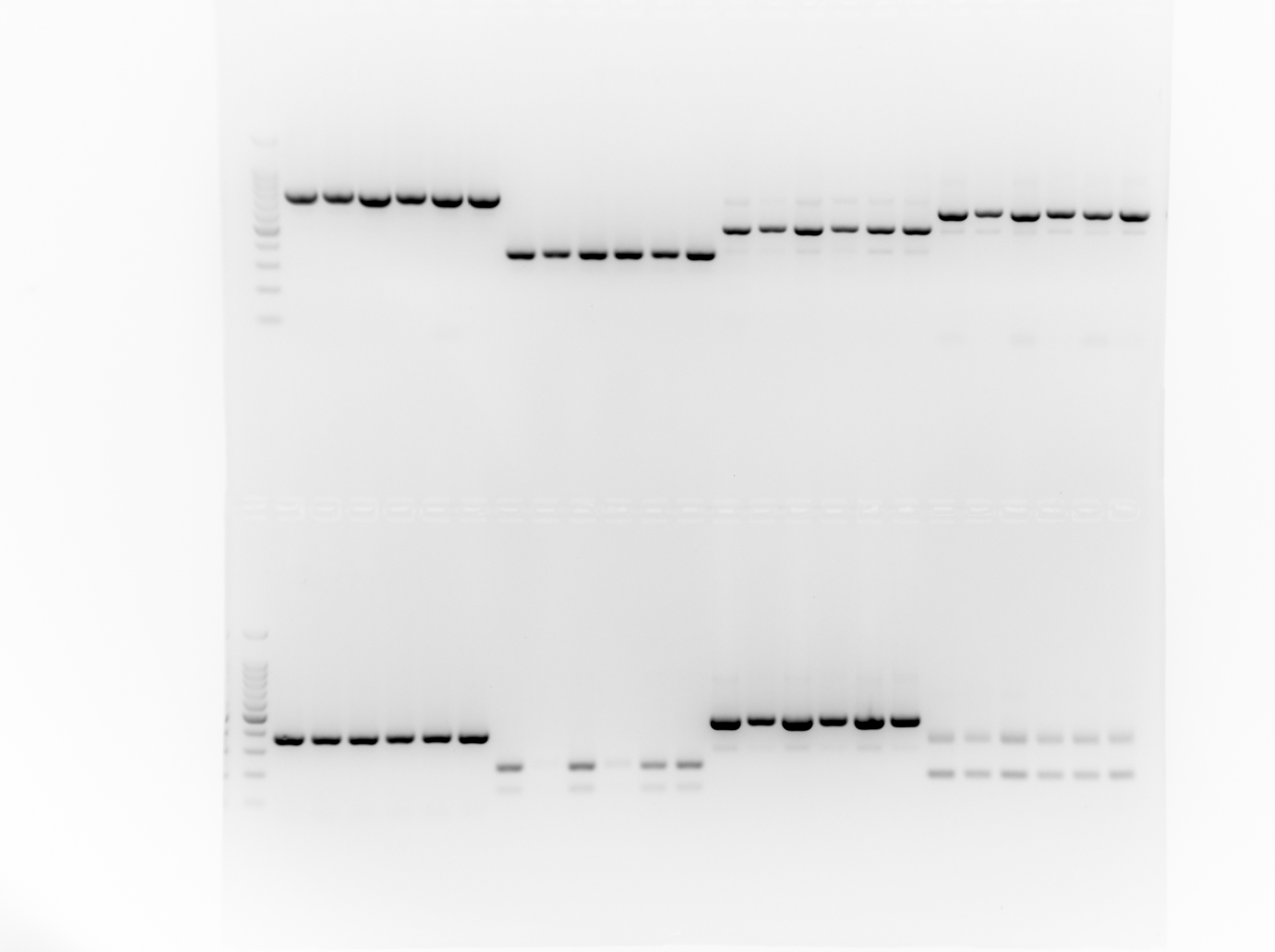

Supplement: Figure 7—source data 2. [file elife-91666-fig7-data2.zip › Figure 7-source data 2/Kat5-unedited.tif]

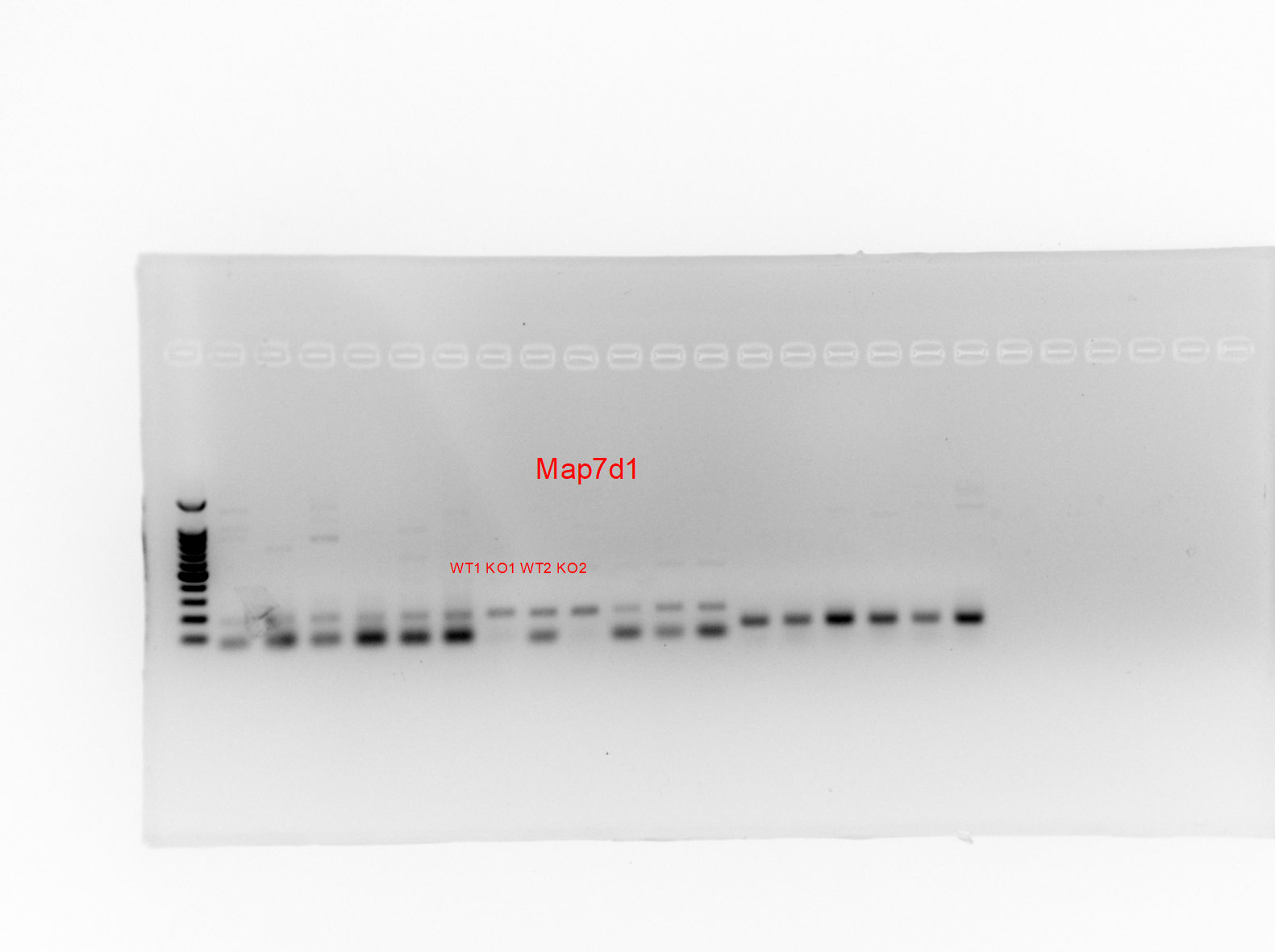

Supplement: Figure 7—source data 2. [file elife-91666-fig7-data2.zip › Figure 7-source data 2/Map7d1-labelled.tif]

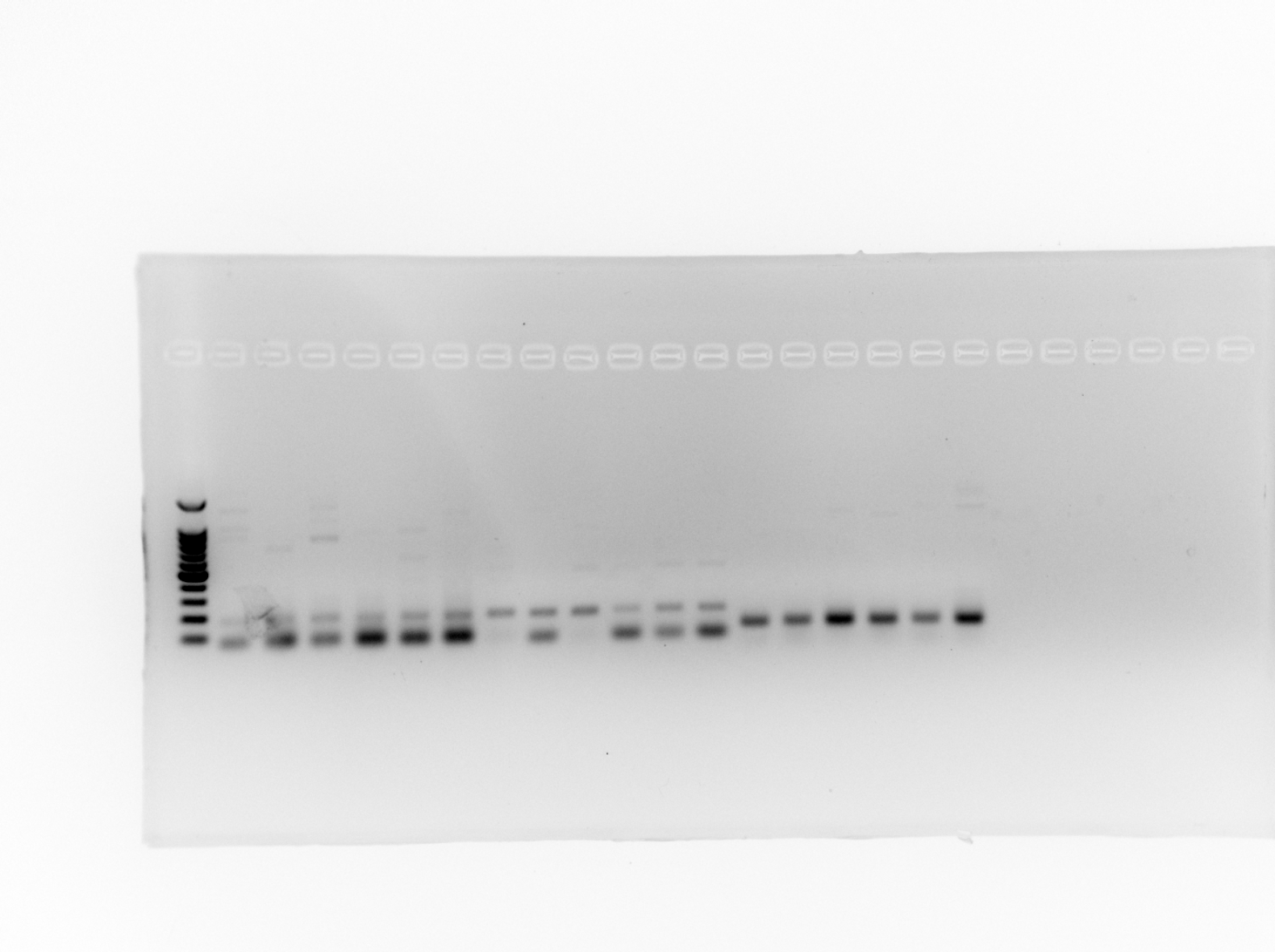

Supplement: Figure 7—source data 2. [file elife-91666-fig7-data2.zip › Figure 7-source data 2/Map7d1-unedited.tif]

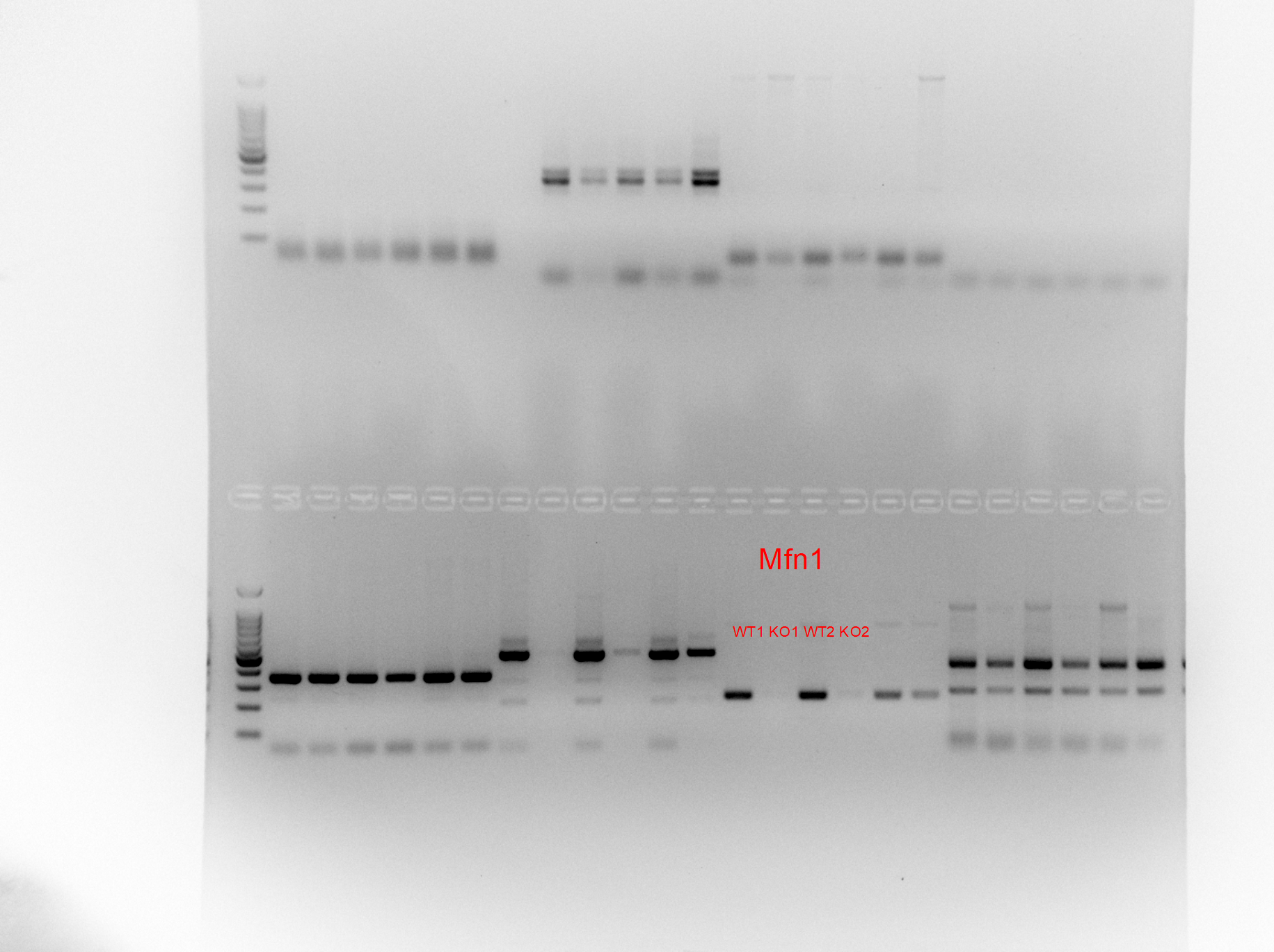

Supplement: Figure 7—source data 2. [file elife-91666-fig7-data2.zip › Figure 7-source data 2/Mfn1-labelled.tif]

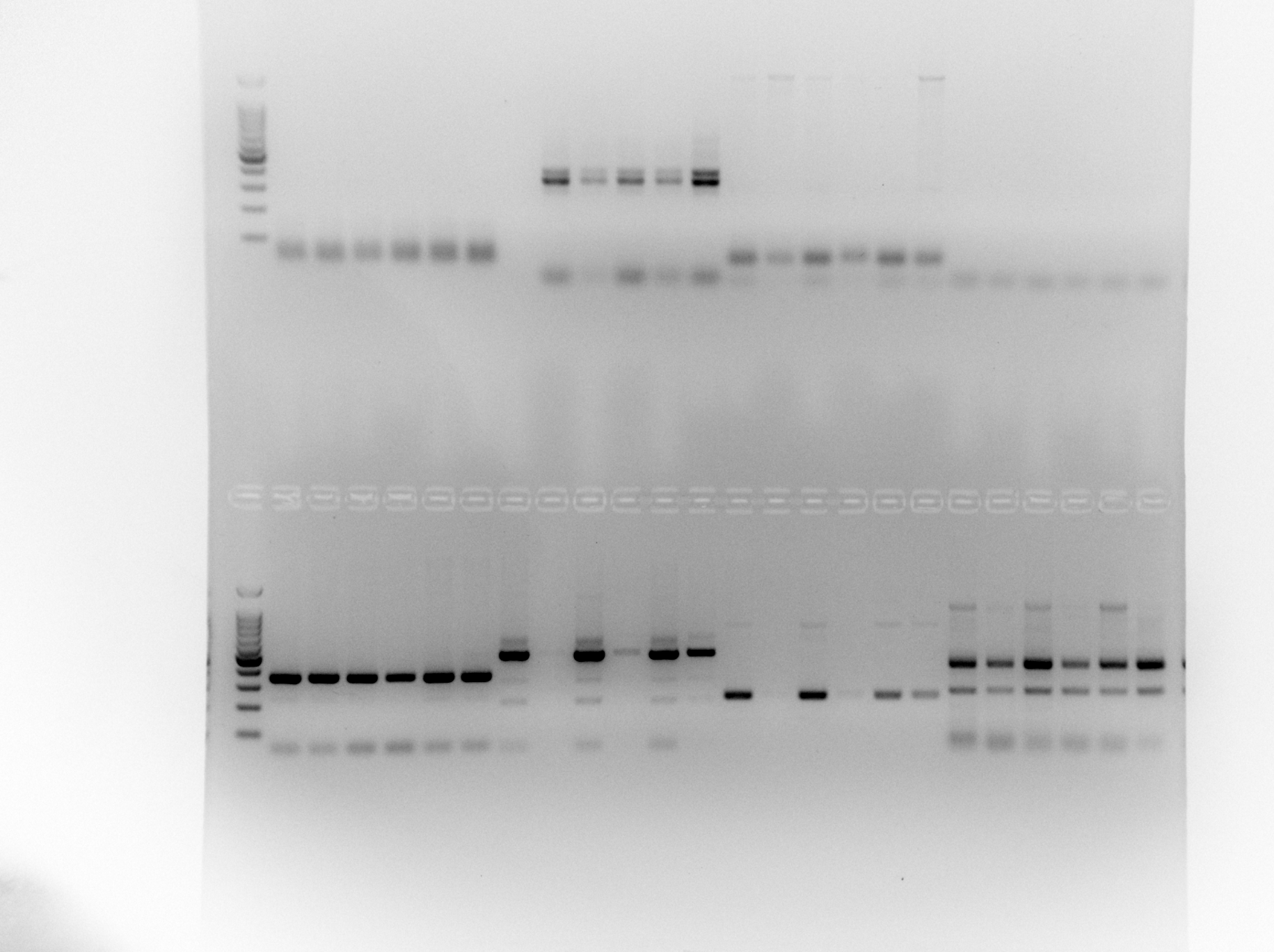

Supplement: Figure 7—source data 2. [file elife-91666-fig7-data2.zip › Figure 7-source data 2/Mfn1-unedited .tif]

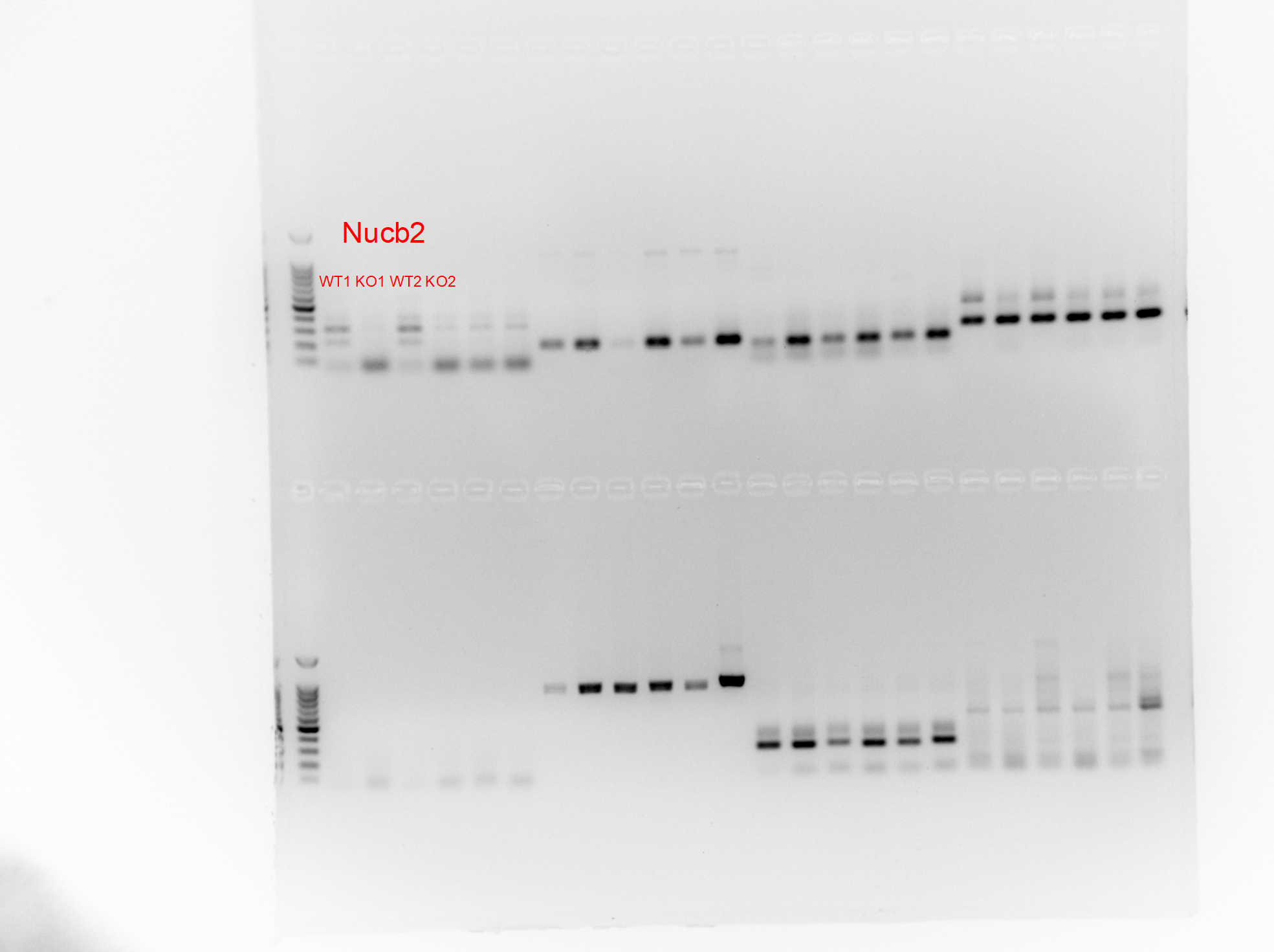

Supplement: Figure 7—source data 2. [file elife-91666-fig7-data2.zip › Figure 7-source data 2/Nucb2-labelled.tif]

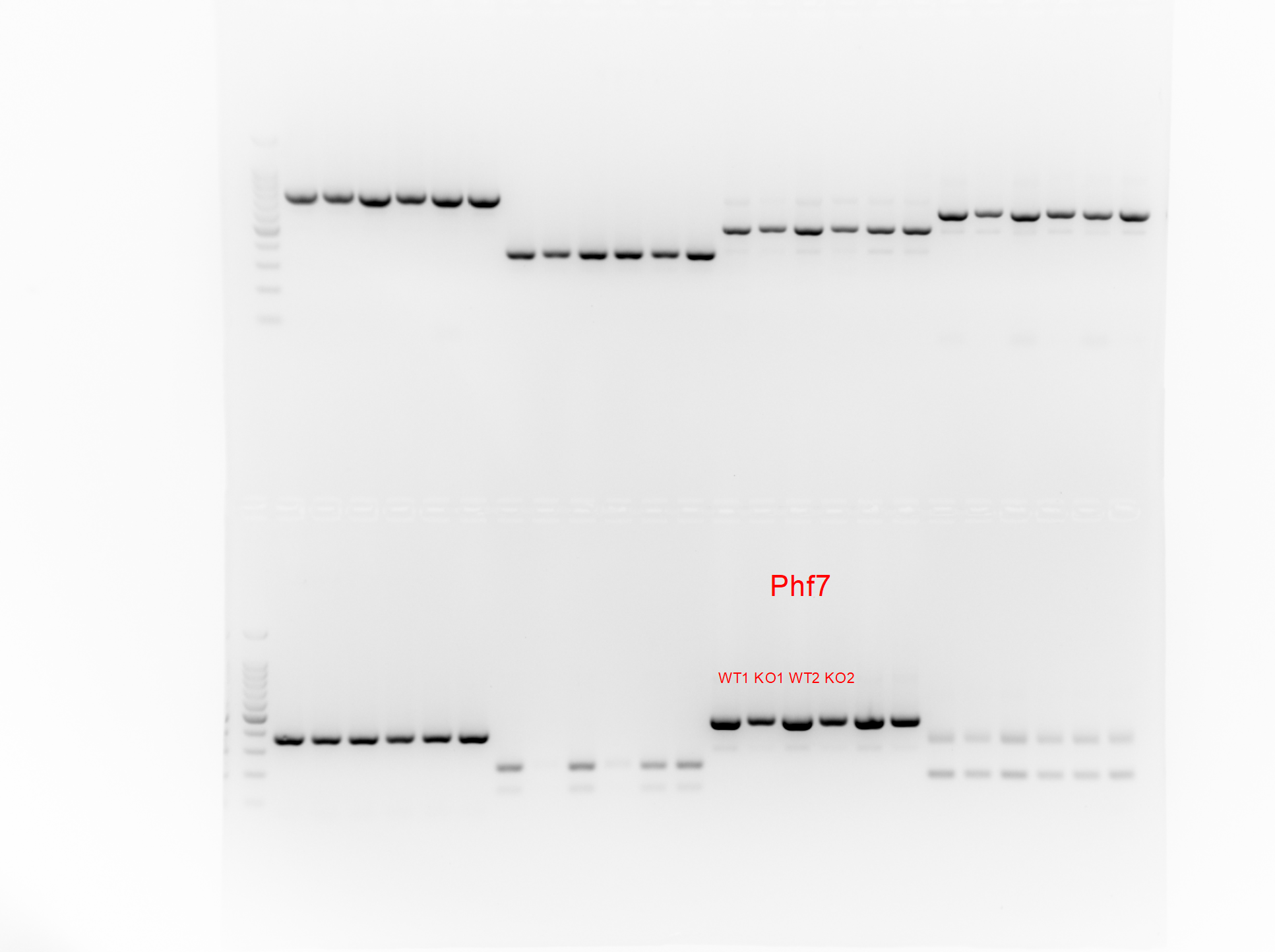

Supplement: Figure 7—source data 2. [file elife-91666-fig7-data2.zip › Figure 7-source data 2/Phf7-labelled.tif]

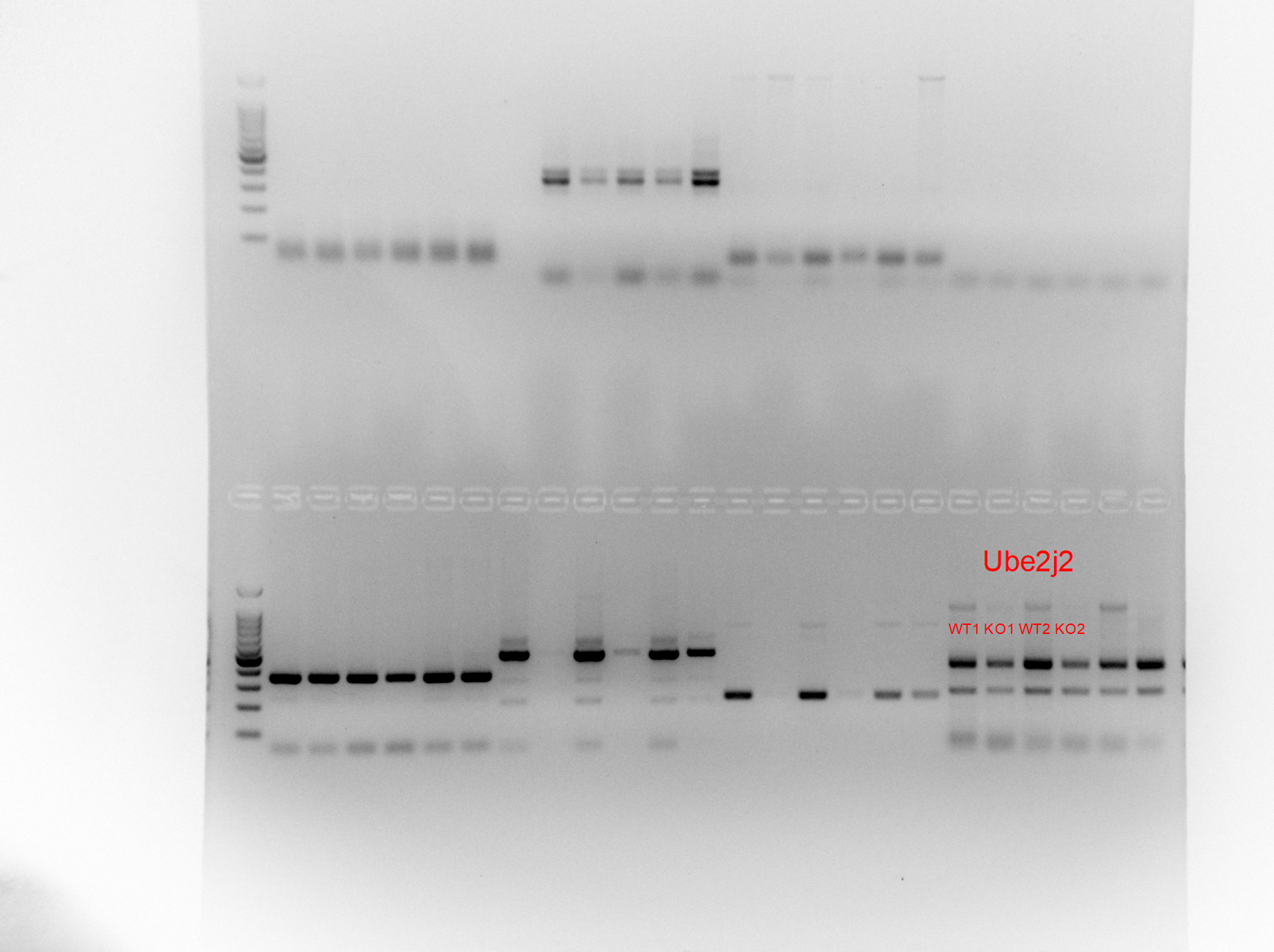

Supplement: Figure 7—source data 2. [file elife-91666-fig7-data2.zip › Figure 7-source data 2/Ube2j2-labelled.tif]

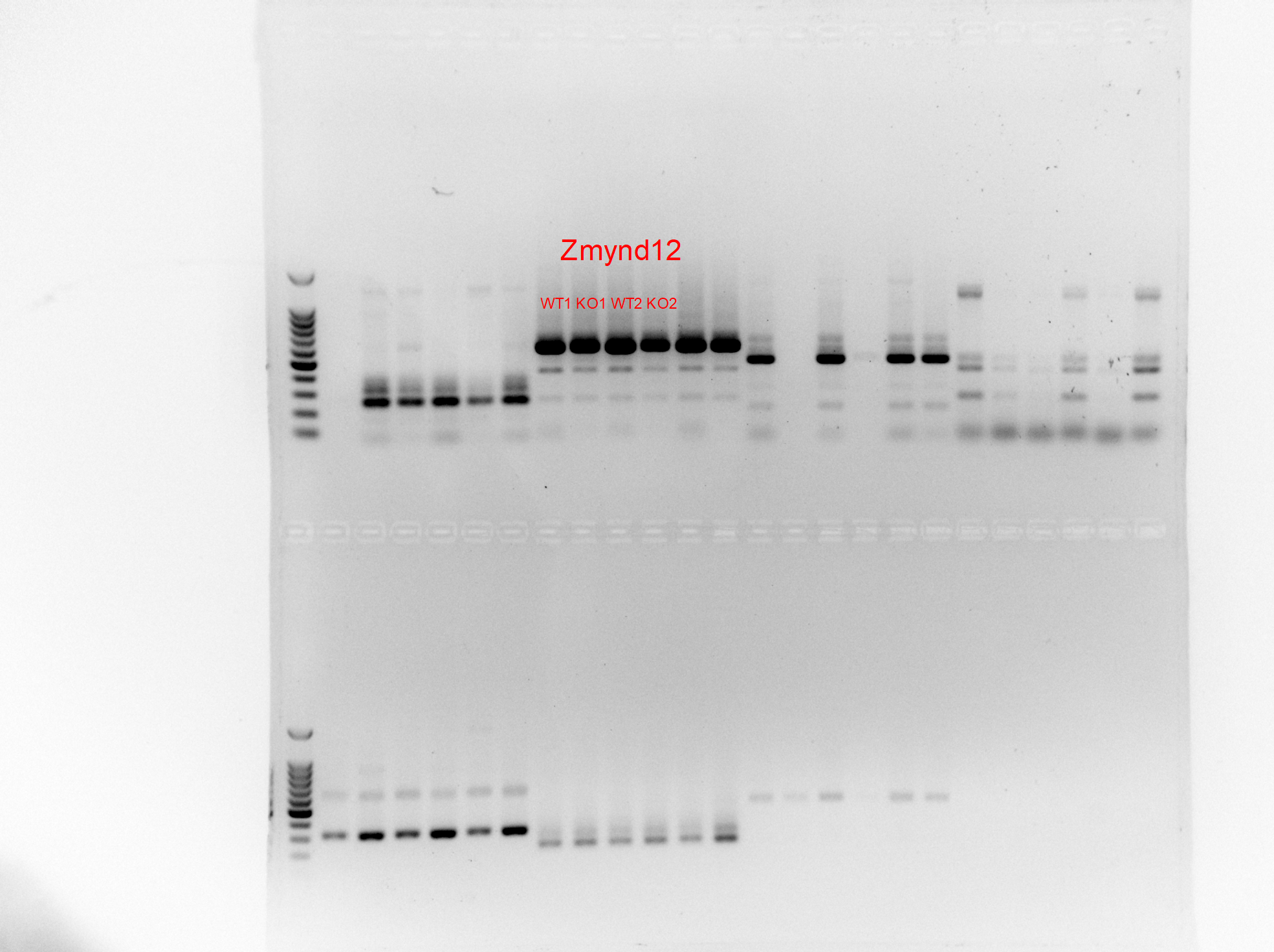

Supplement: Figure 7—source data 2. [file elife-91666-fig7-data2.zip › Figure 7-source data 2/Zmynd12-labelled.tif]

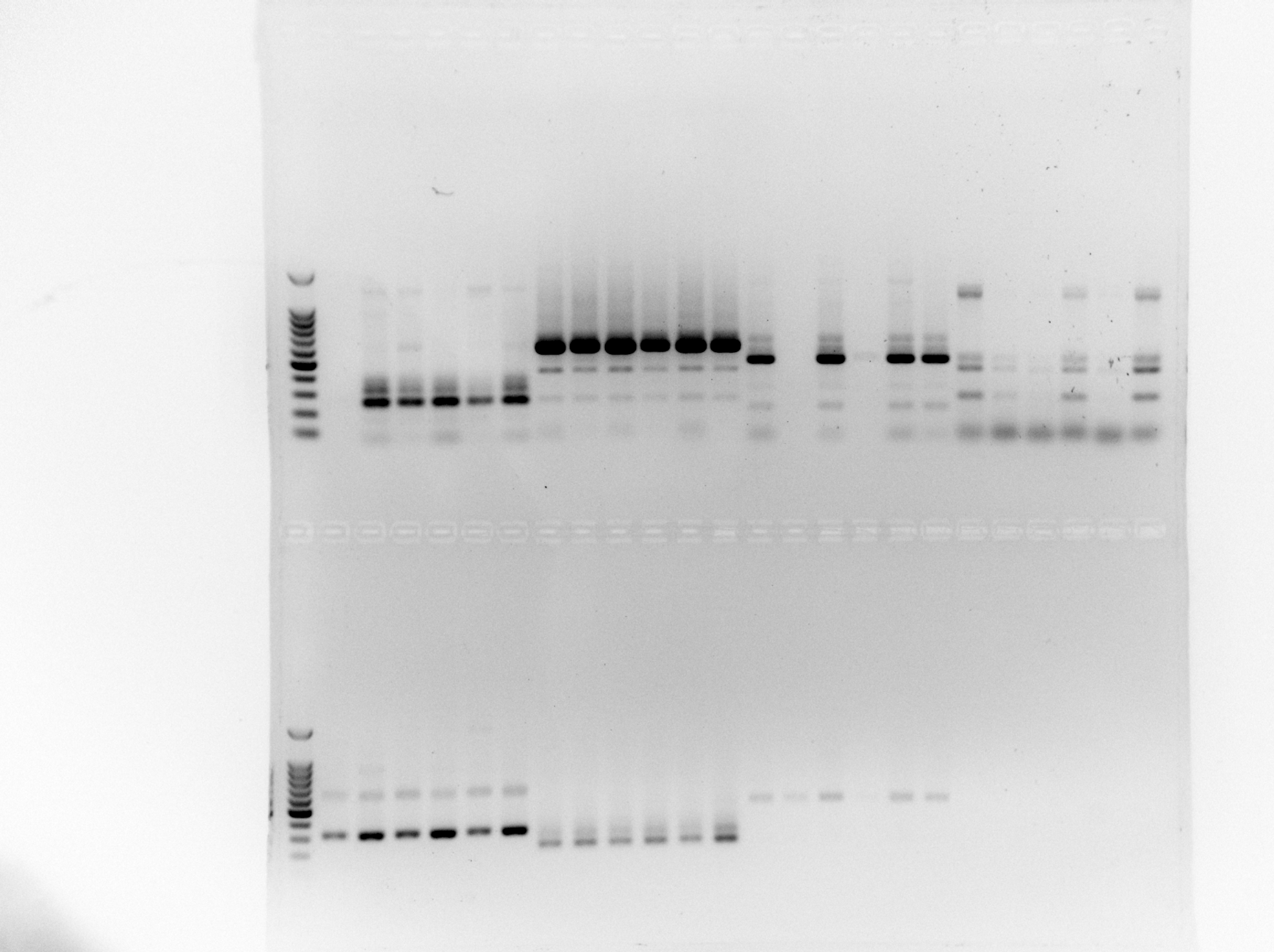

Supplement: Figure 7—source data 2. [file elife-91666-fig7-data2.zip › Figure 7-source data 2/Zmynd12-unedited.tif]

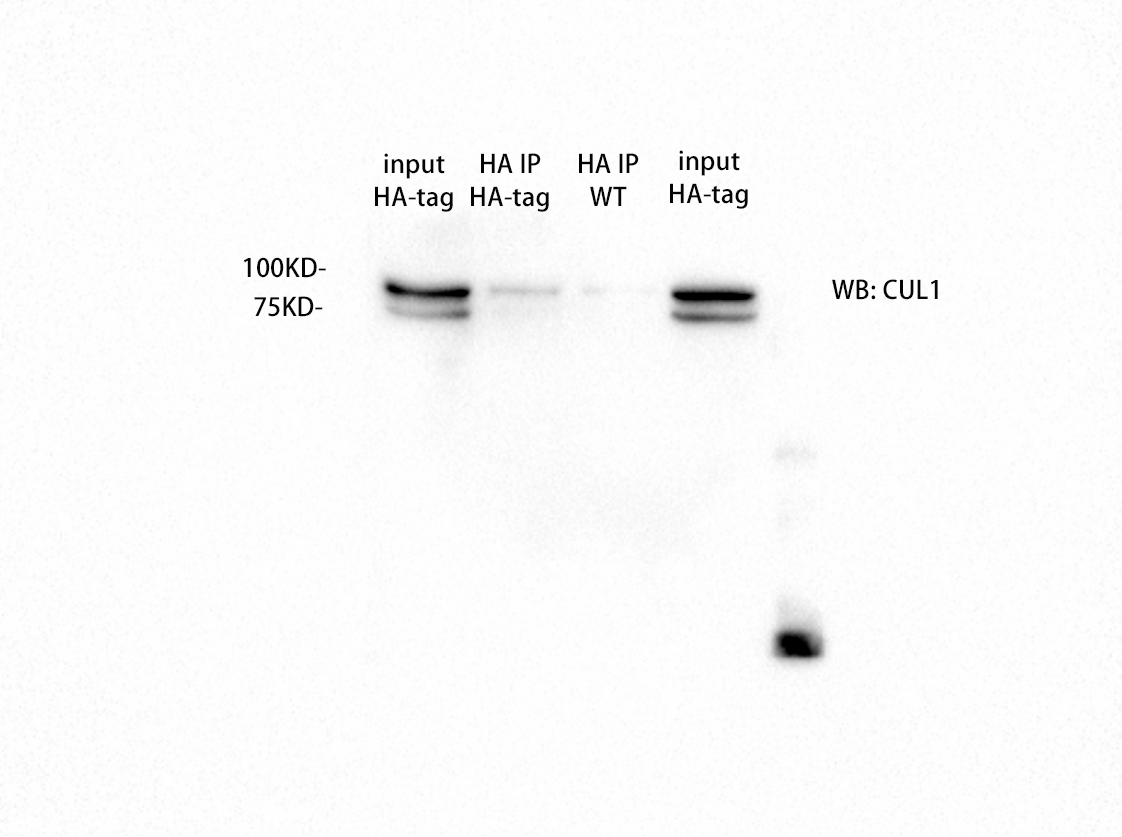

Supplement: Figure 8—source data 1. [file elife-91666-fig8-data1.zip › Figure 8-source data 1/CUL1(2)-labelled.tif]

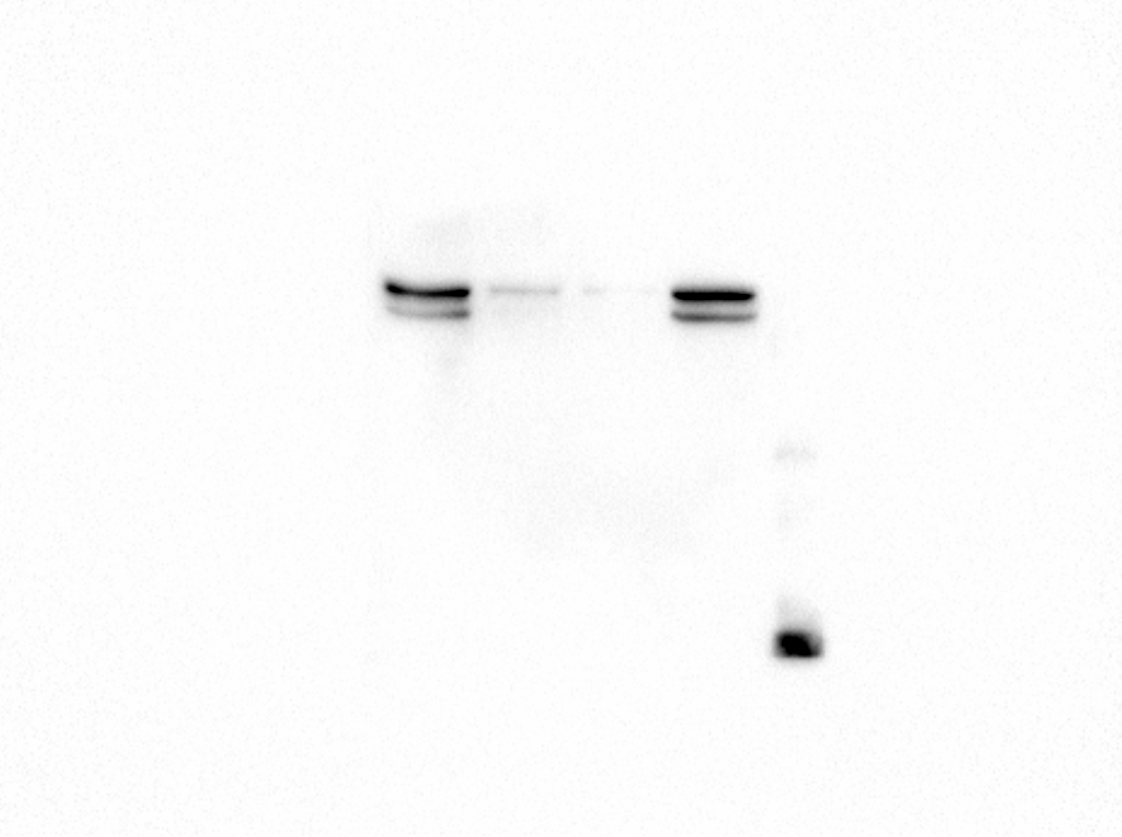

Supplement: Figure 8—source data 1. [file elife-91666-fig8-data1.zip › Figure 8-source data 1/CUL1(2)-unedited.tif]

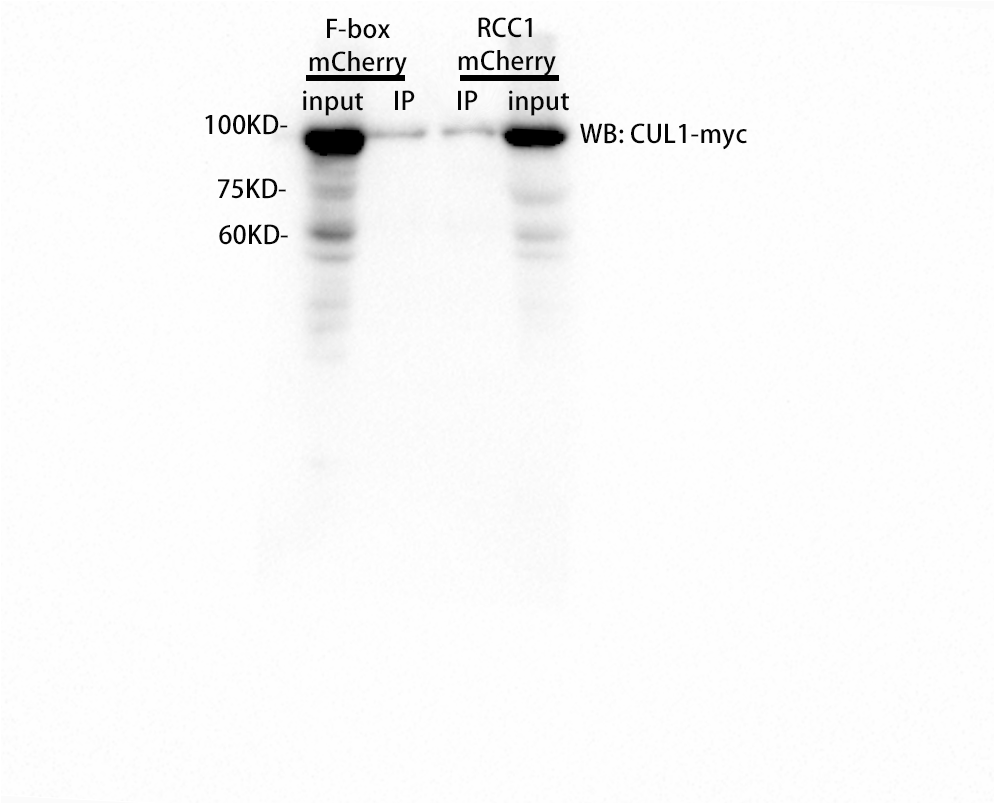

Supplement: Figure 8—source data 1. [file elife-91666-fig8-data1.zip › Figure 8-source data 1/CUL1(3)-labelled.tif]

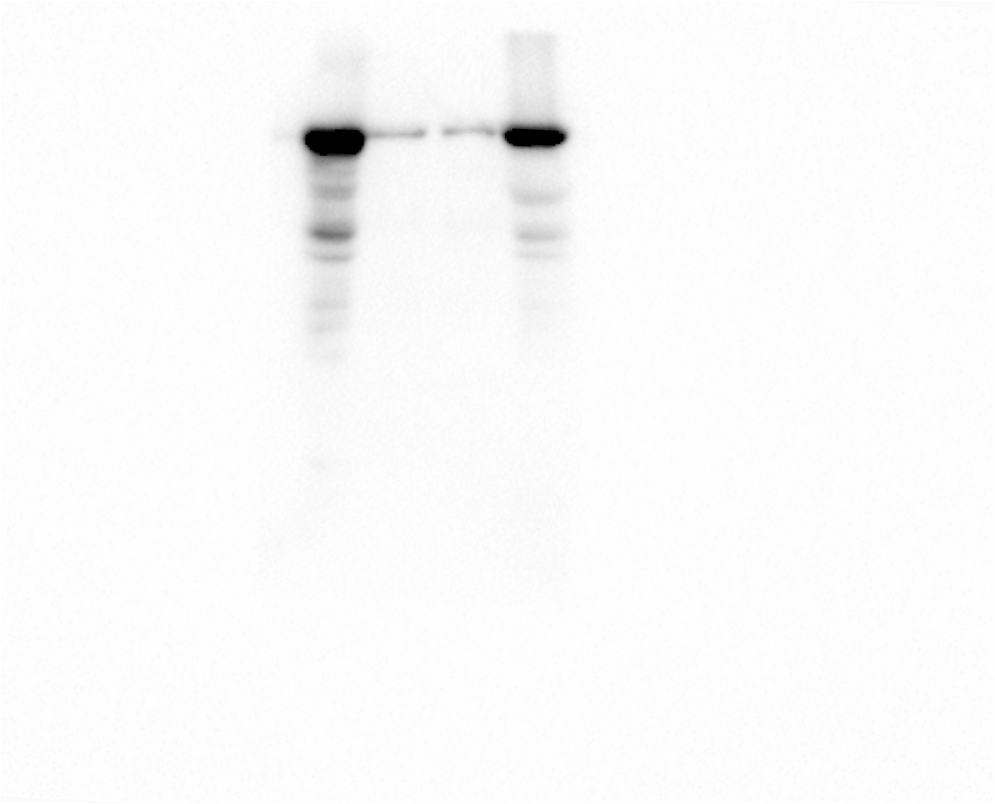

Supplement: Figure 8—source data 1. [file elife-91666-fig8-data1.zip › Figure 8-source data 1/CUL1(3)-unedited.tif]

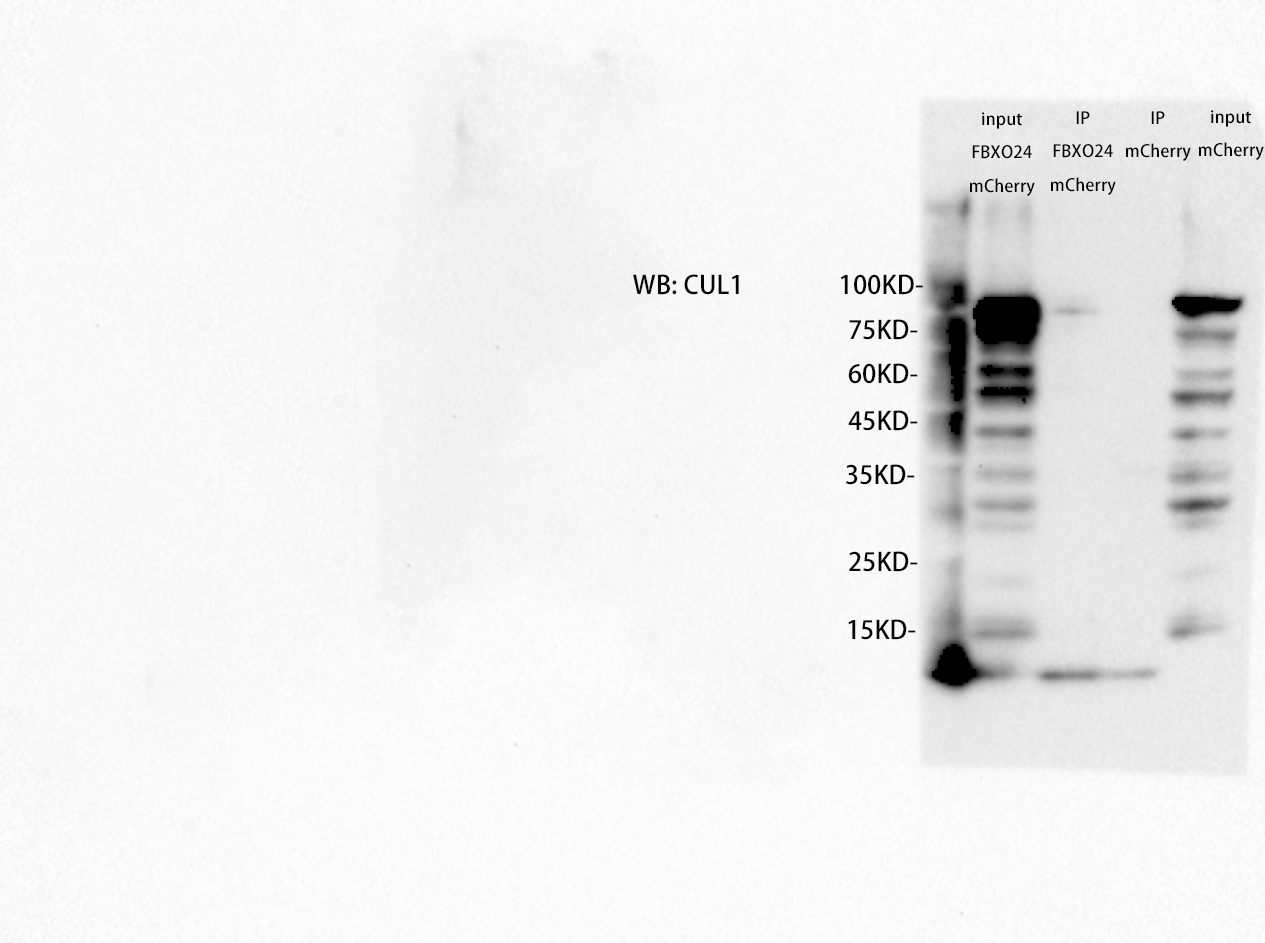

Supplement: Figure 8—source data 1. [file elife-91666-fig8-data1.zip › Figure 8-source data 1/CUL1-labelled.tif]

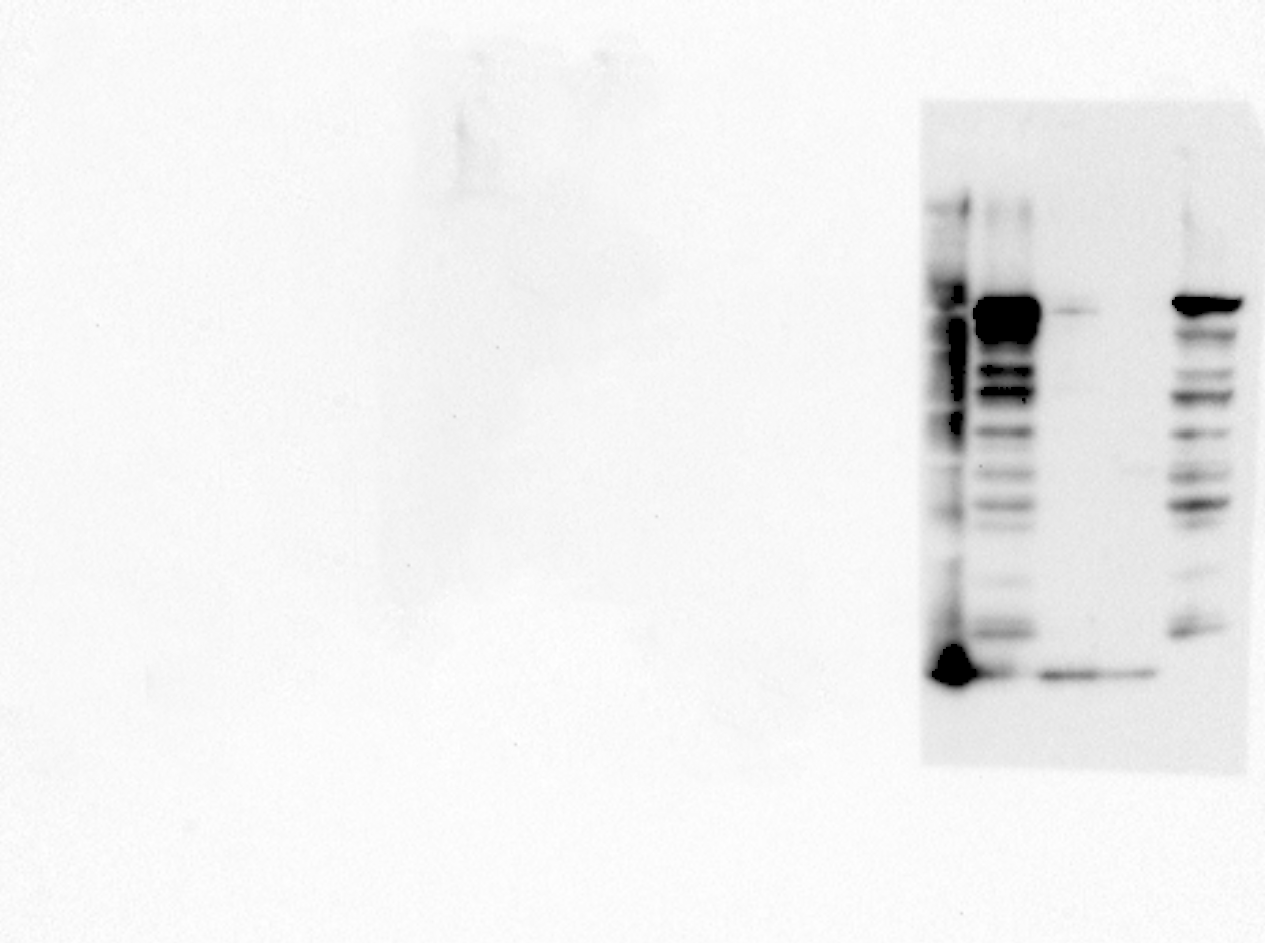

Supplement: Figure 8—source data 1. [file elife-91666-fig8-data1.zip › Figure 8-source data 1/CUL1-unedited.tif]

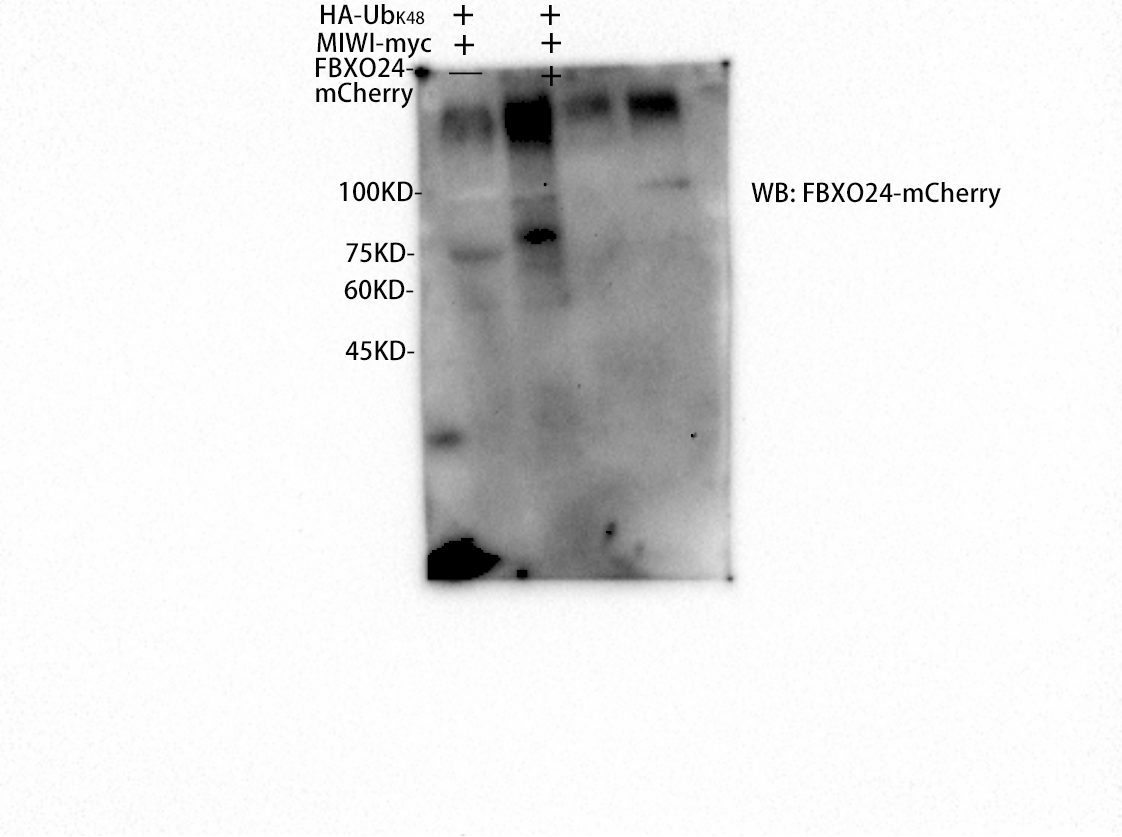

Supplement: Figure 8—source data 1. [file elife-91666-fig8-data1.zip › Figure 8-source data 1/FBXO24-mCherry(2)-labelled.tif]

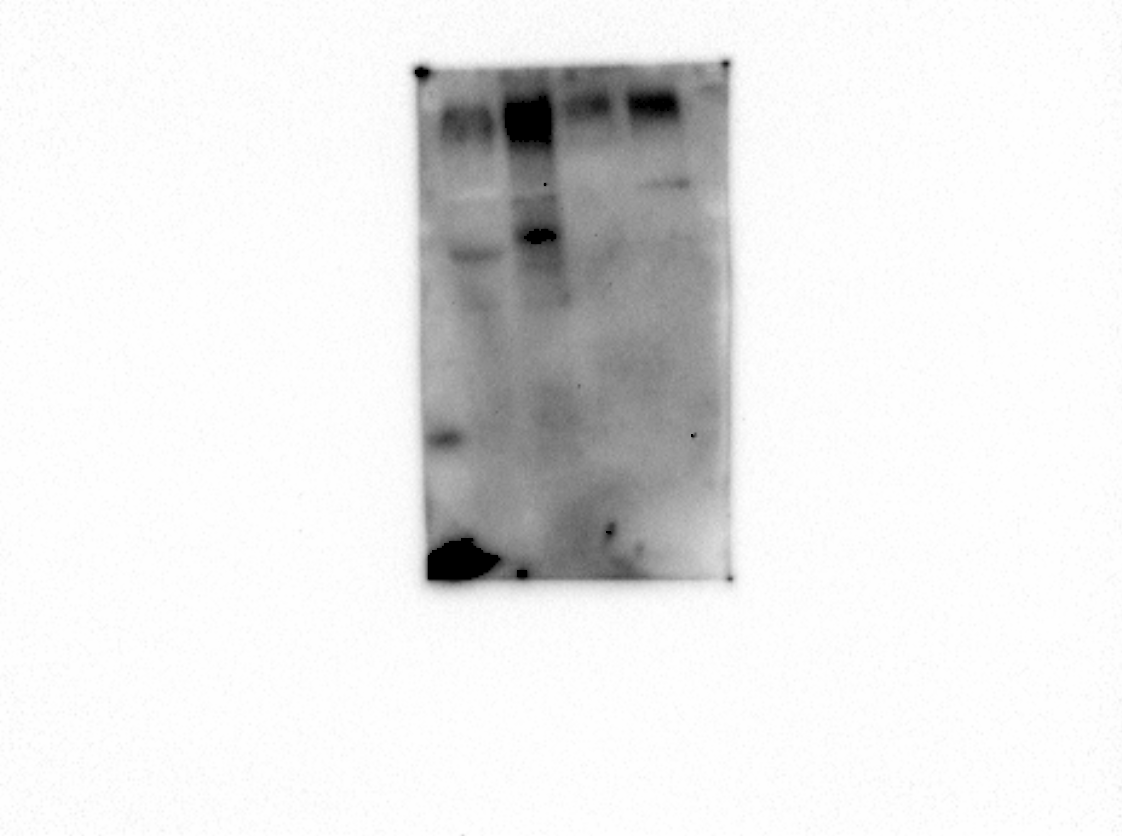

Supplement: Figure 8—source data 1. [file elife-91666-fig8-data1.zip › Figure 8-source data 1/FBXO24-mCherry(2)-unedited.tif]
