# Supplementary figures and images for: FBXO24 modulates mRNA alternative splicing and MIWI degradation and is required for normal sperm formation and male fertility (part 2 of 2)
Source: eLife. 2024 Mar 12;12:RP91666. doi: 10.7554/eLife.91666 (PMC10932545; doi:10.7554/eLife.91666)

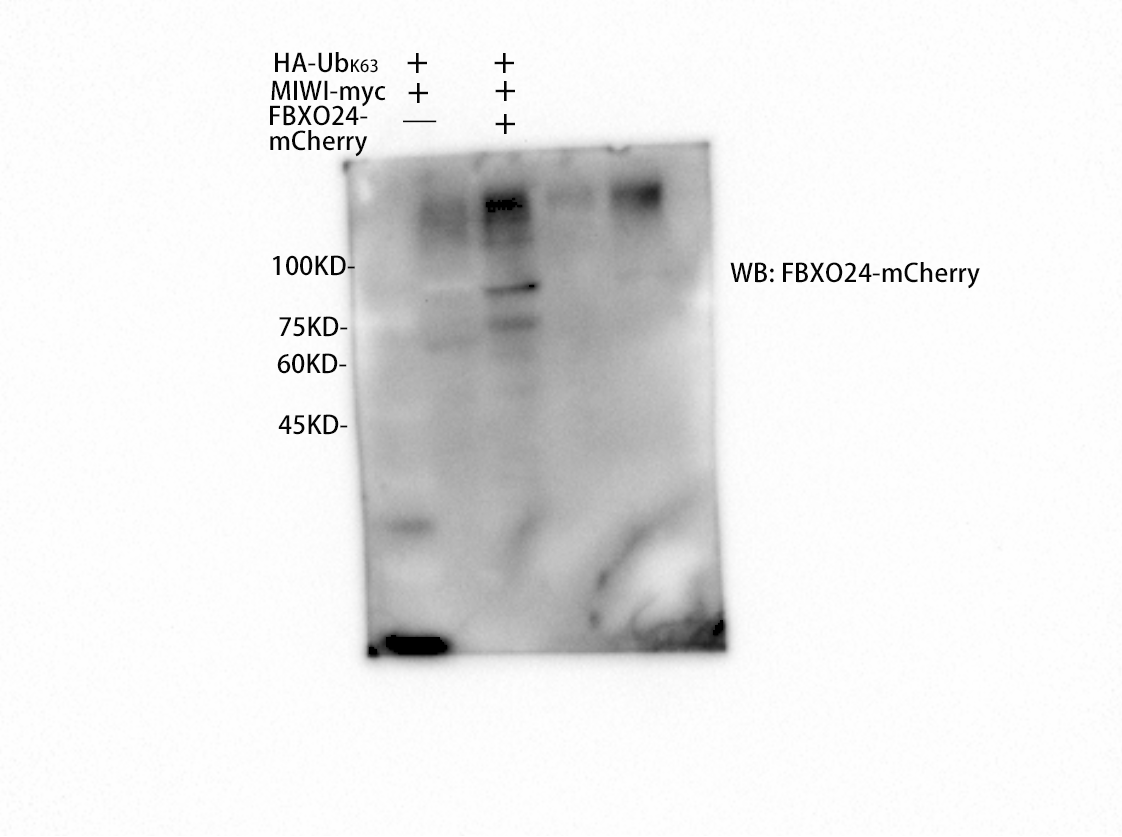

Supplement: Figure 8—source data 1. [file elife-91666-fig8-data1.zip › Figure 8-source data 1/FBXO24-mCherry(3)-labelled.tif]

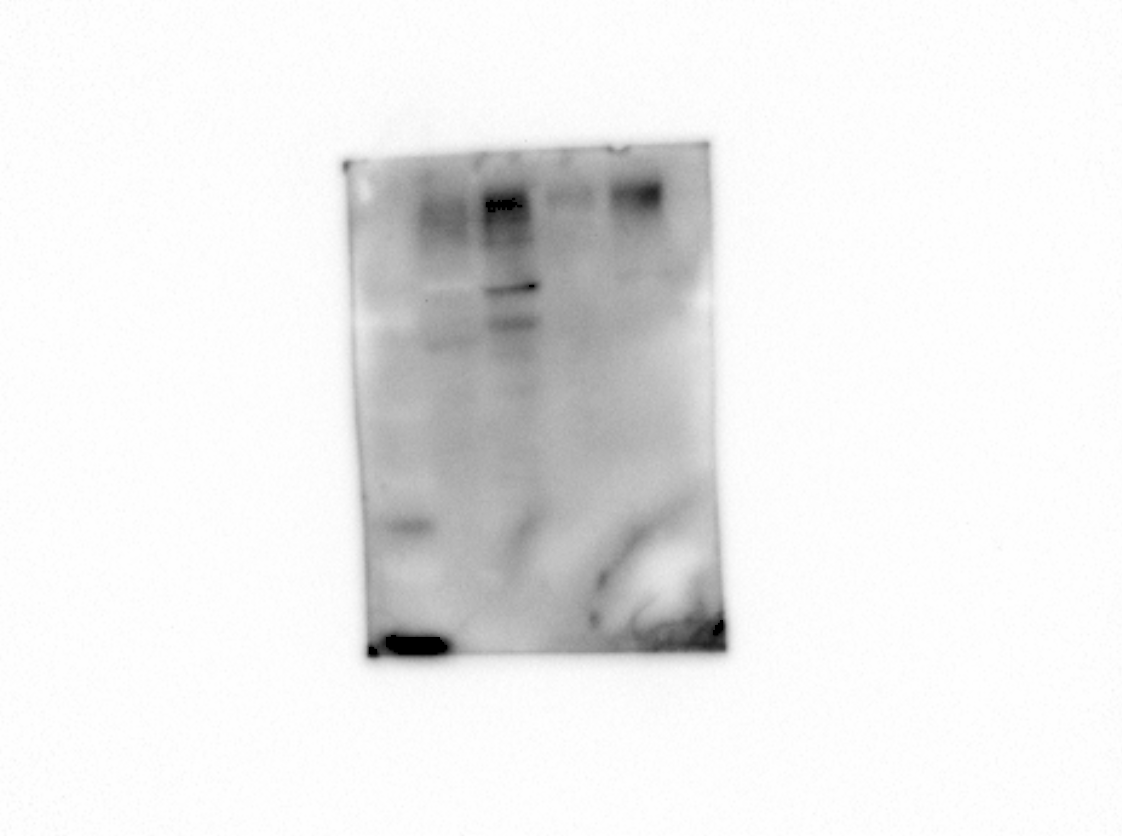

Supplement: Figure 8—source data 1. [file elife-91666-fig8-data1.zip › Figure 8-source data 1/FBXO24-mCherry(3)-unedited.tif]

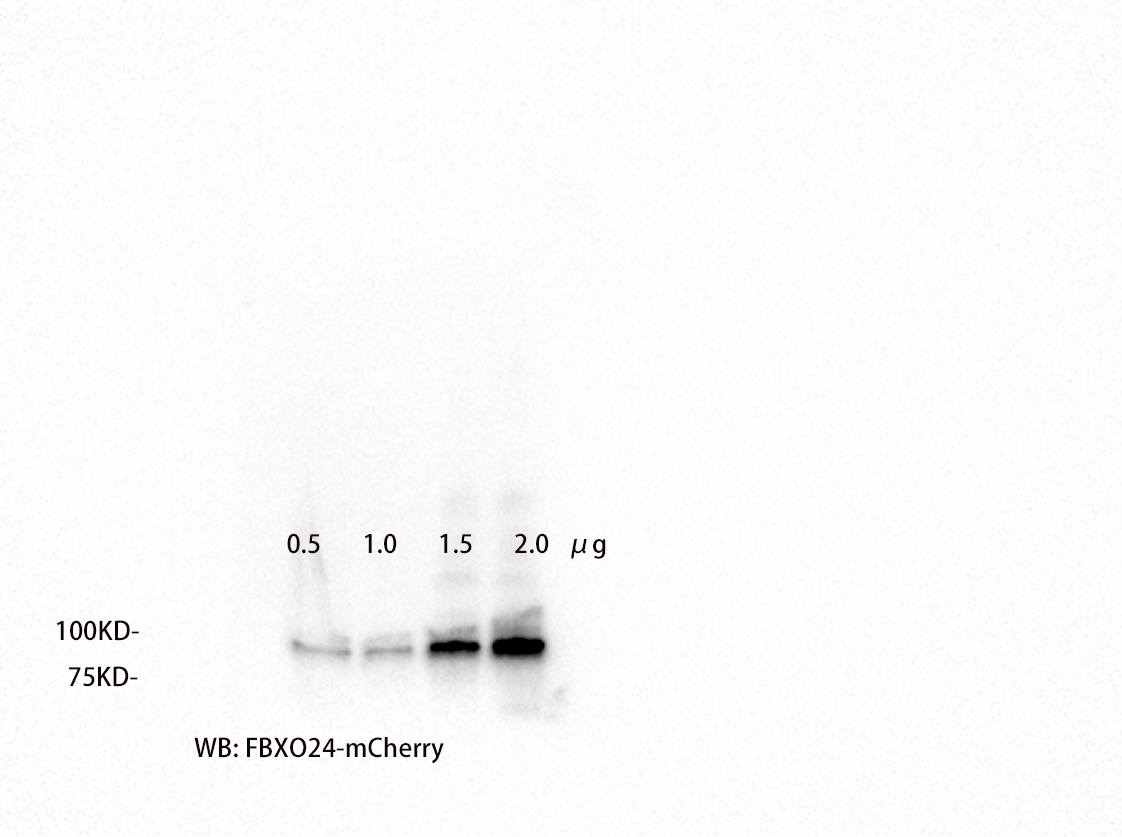

Supplement: Figure 8—source data 1. [file elife-91666-fig8-data1.zip › Figure 8-source data 1/FBXO24-mCherry(4)-labelled.tif]

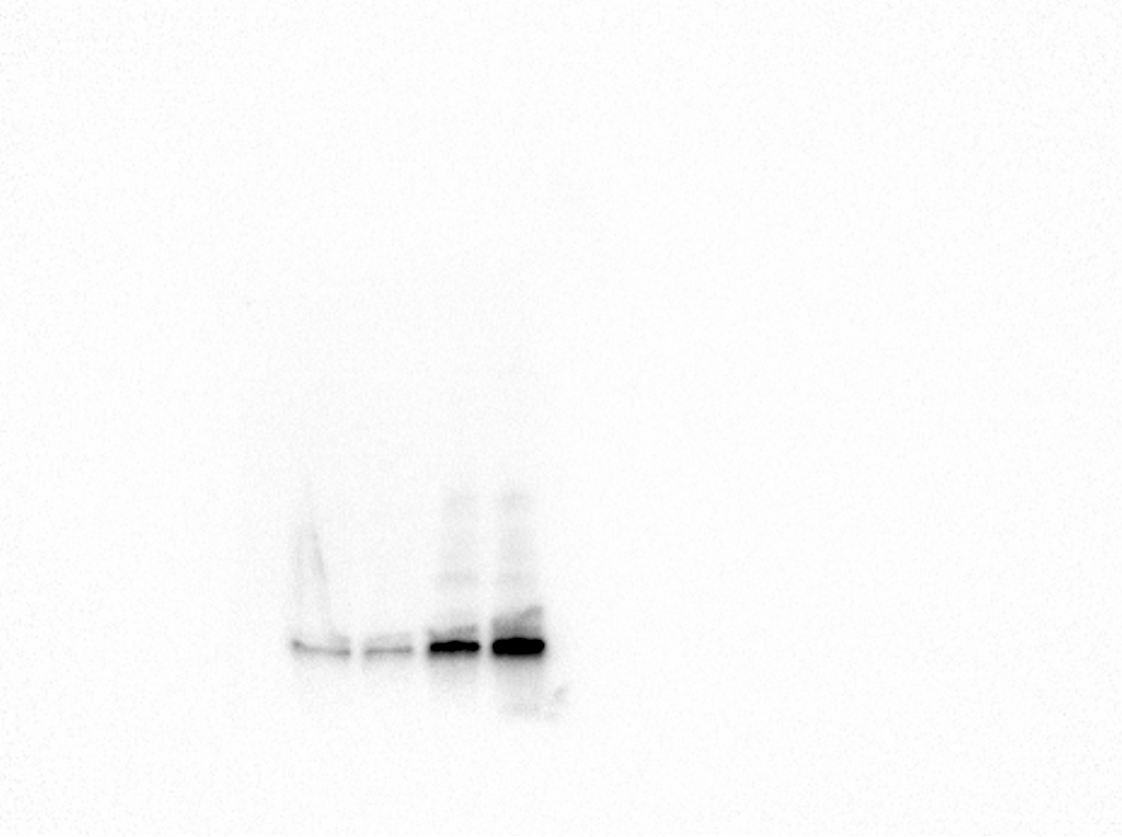

Supplement: Figure 8—source data 1. [file elife-91666-fig8-data1.zip › Figure 8-source data 1/FBXO24-mCherry(4)-unedited.tif]

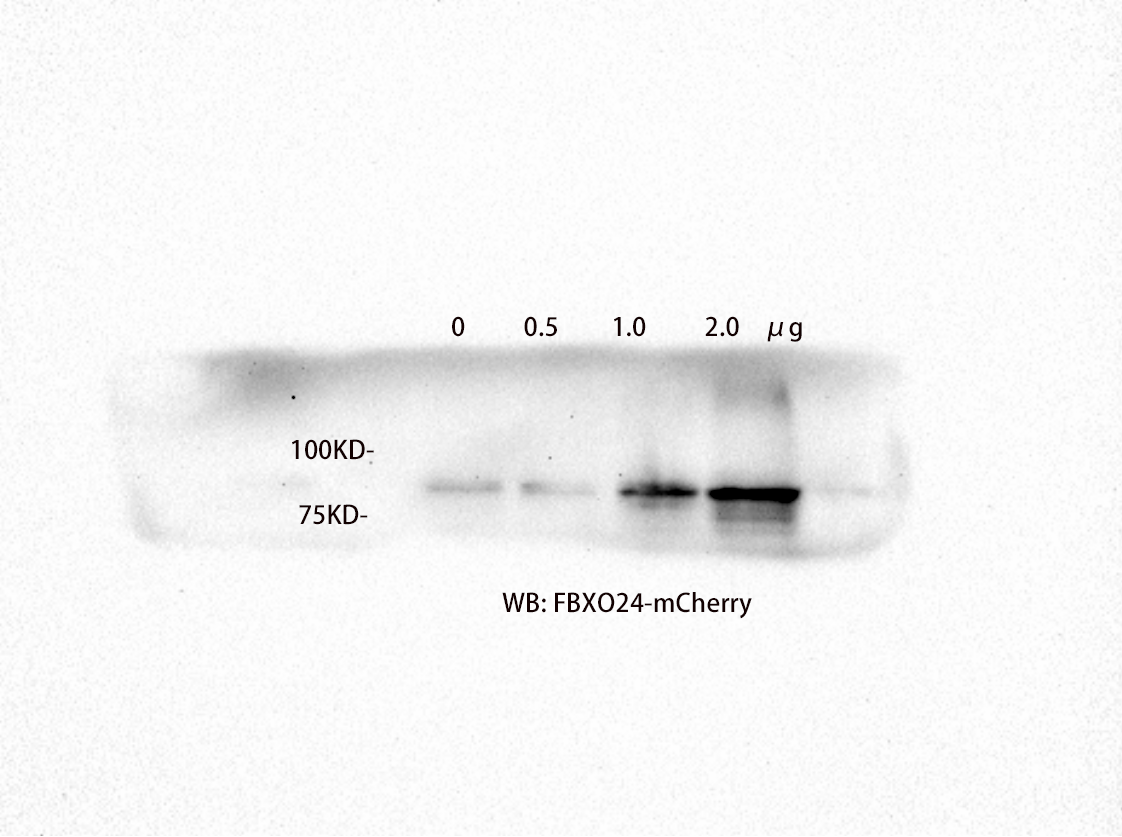

Supplement: Figure 8—source data 1. [file elife-91666-fig8-data1.zip › Figure 8-source data 1/FBXO24-mCherry-labelled.tif]

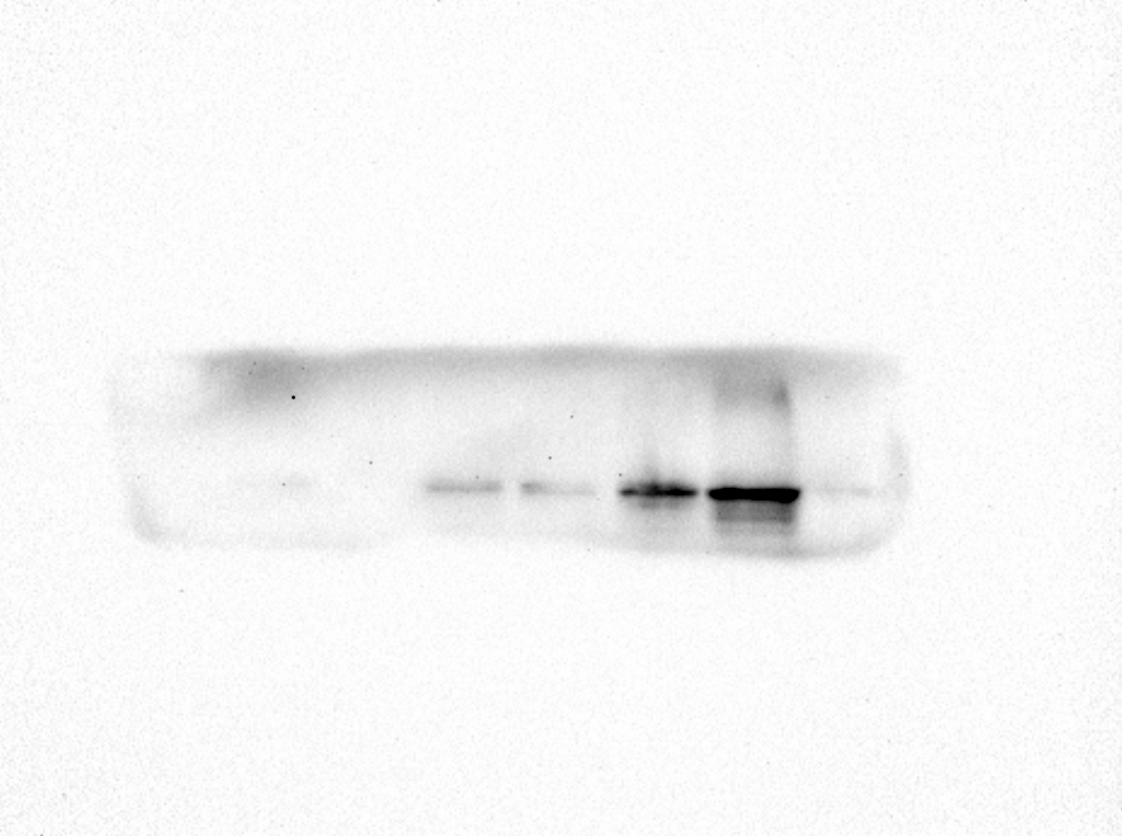

Supplement: Figure 8—source data 1. [file elife-91666-fig8-data1.zip › Figure 8-source data 1/FBXO24-mCherry-unedited.tif]

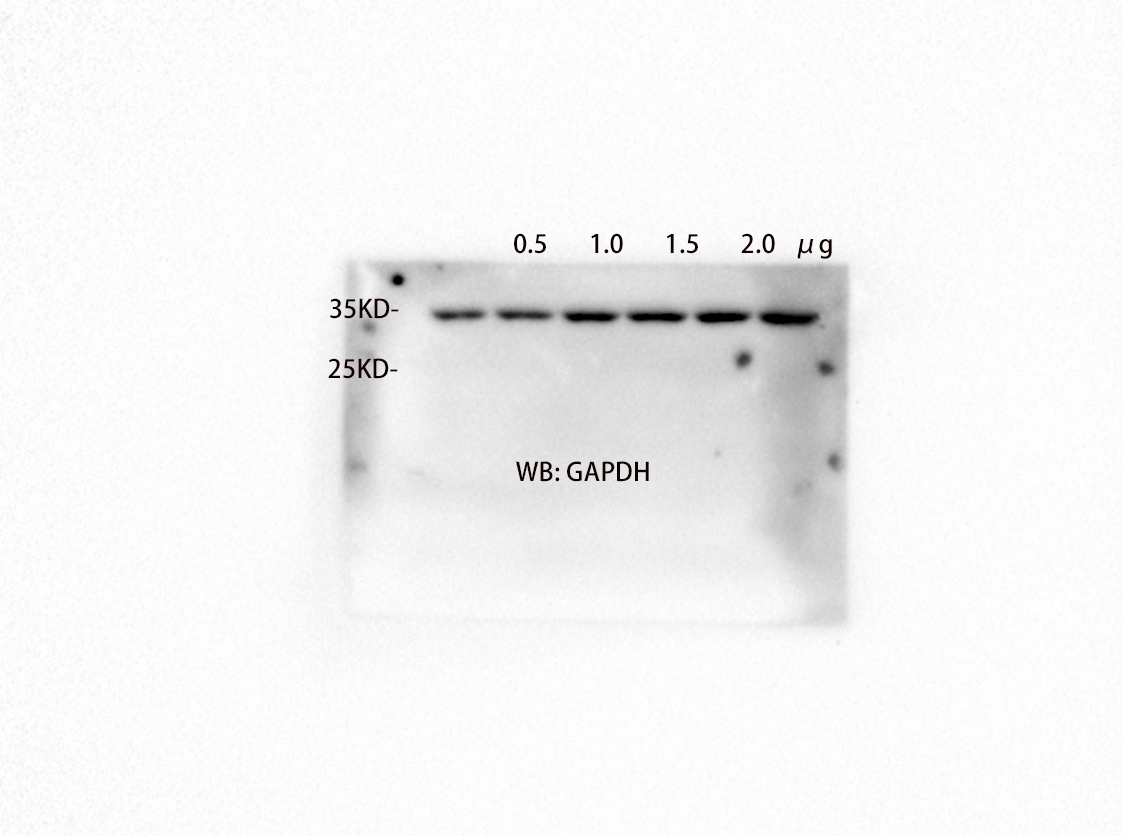

Supplement: Figure 8—source data 1. [file elife-91666-fig8-data1.zip › Figure 8-source data 1/GAPDH(2)-labelled.tif]

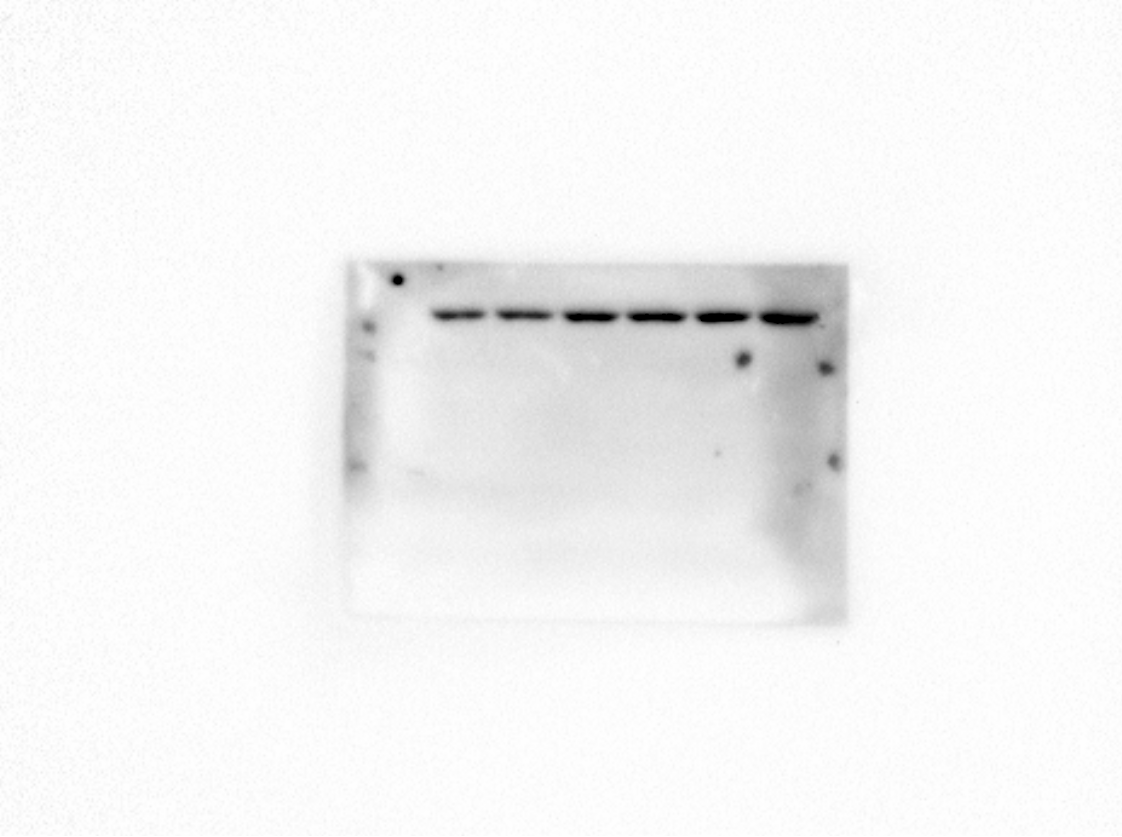

Supplement: Figure 8—source data 1. [file elife-91666-fig8-data1.zip › Figure 8-source data 1/GAPDH(2)-unedited.tif]

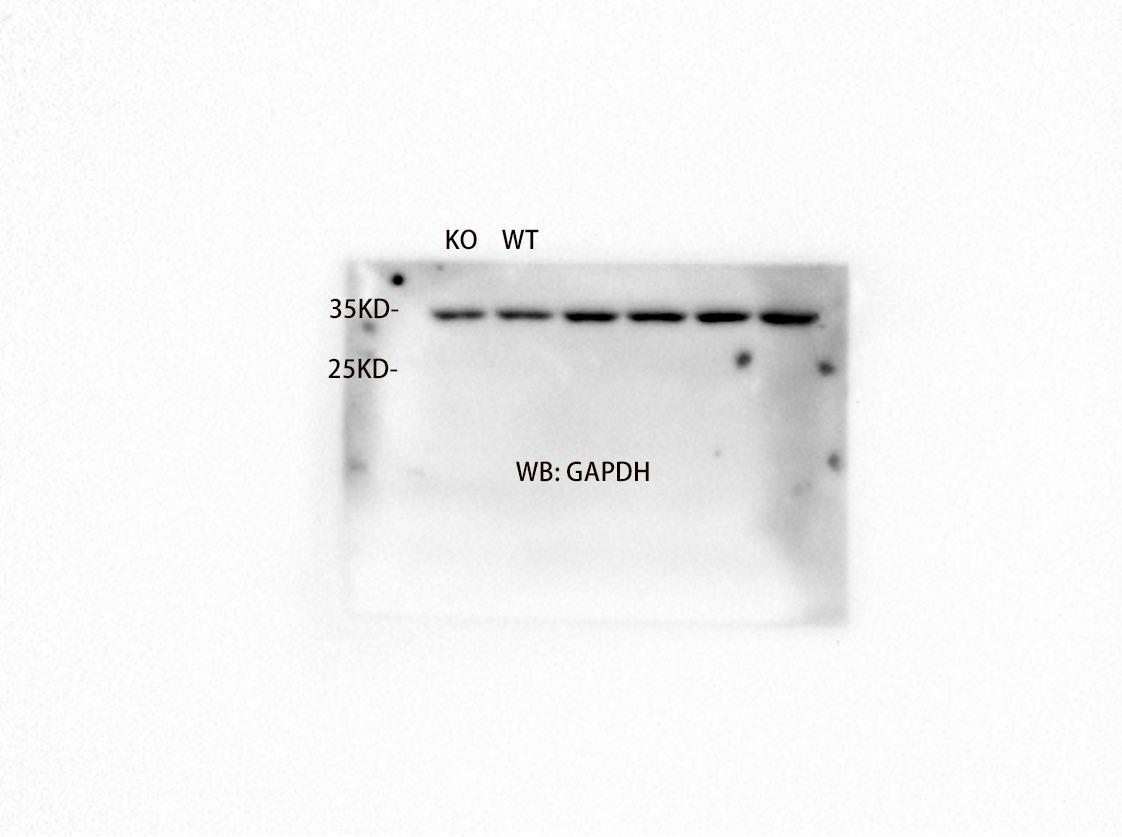

Supplement: Figure 8—source data 1. [file elife-91666-fig8-data1.zip › Figure 8-source data 1/GAPDH(3)-labelled.tif]

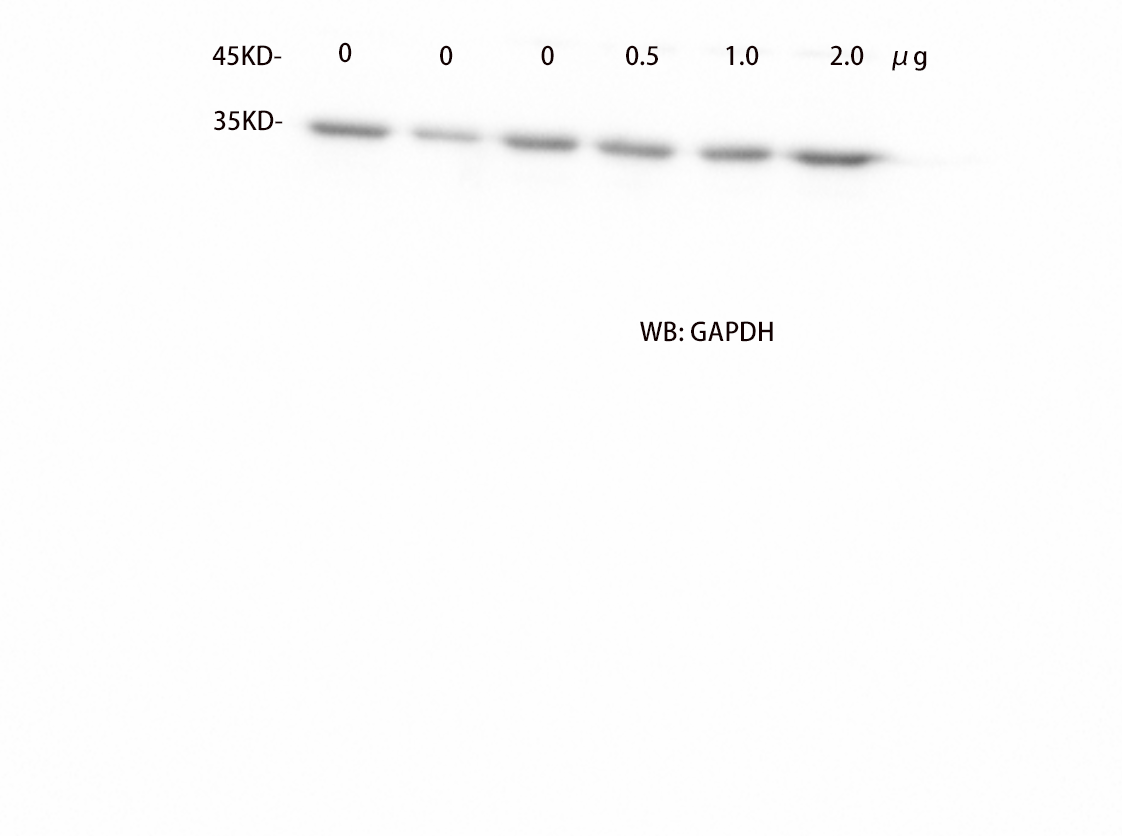

Supplement: Figure 8—source data 1. [file elife-91666-fig8-data1.zip › Figure 8-source data 1/GAPDH-labelled.tif]

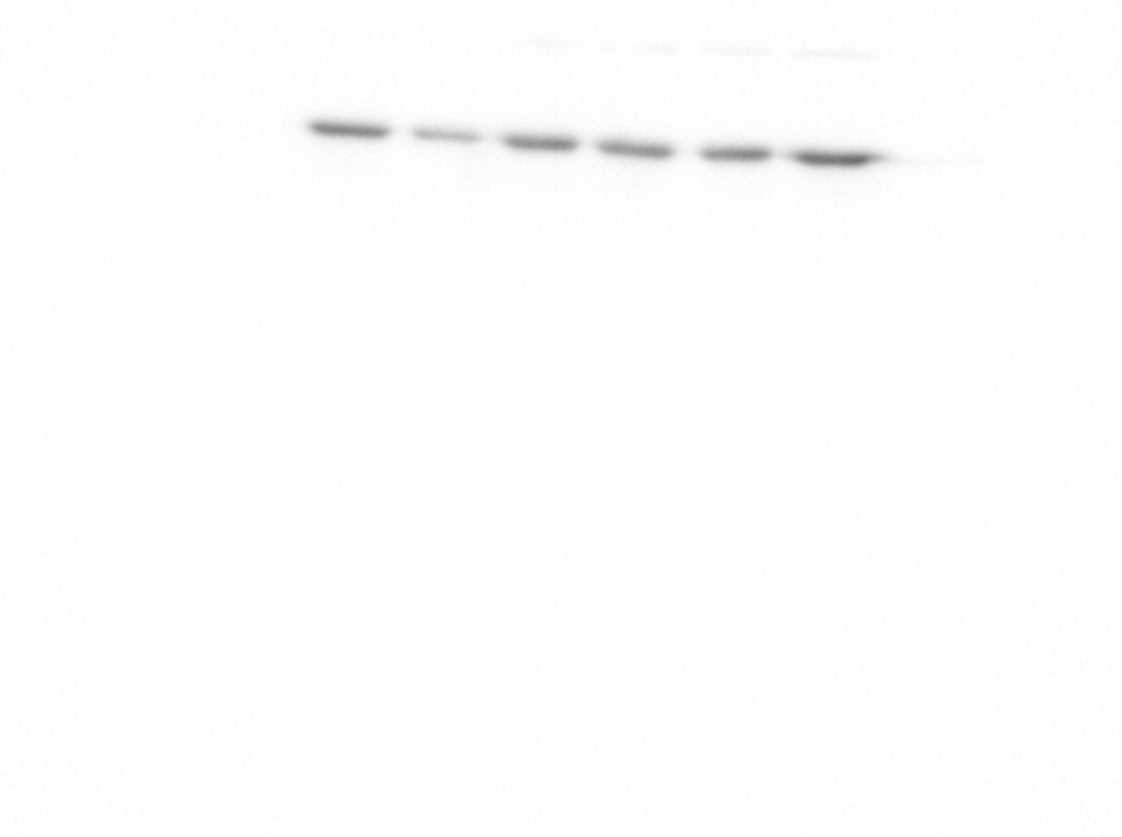

Supplement: Figure 8—source data 1. [file elife-91666-fig8-data1.zip › Figure 8-source data 1/GAPDH-unedited.tif]

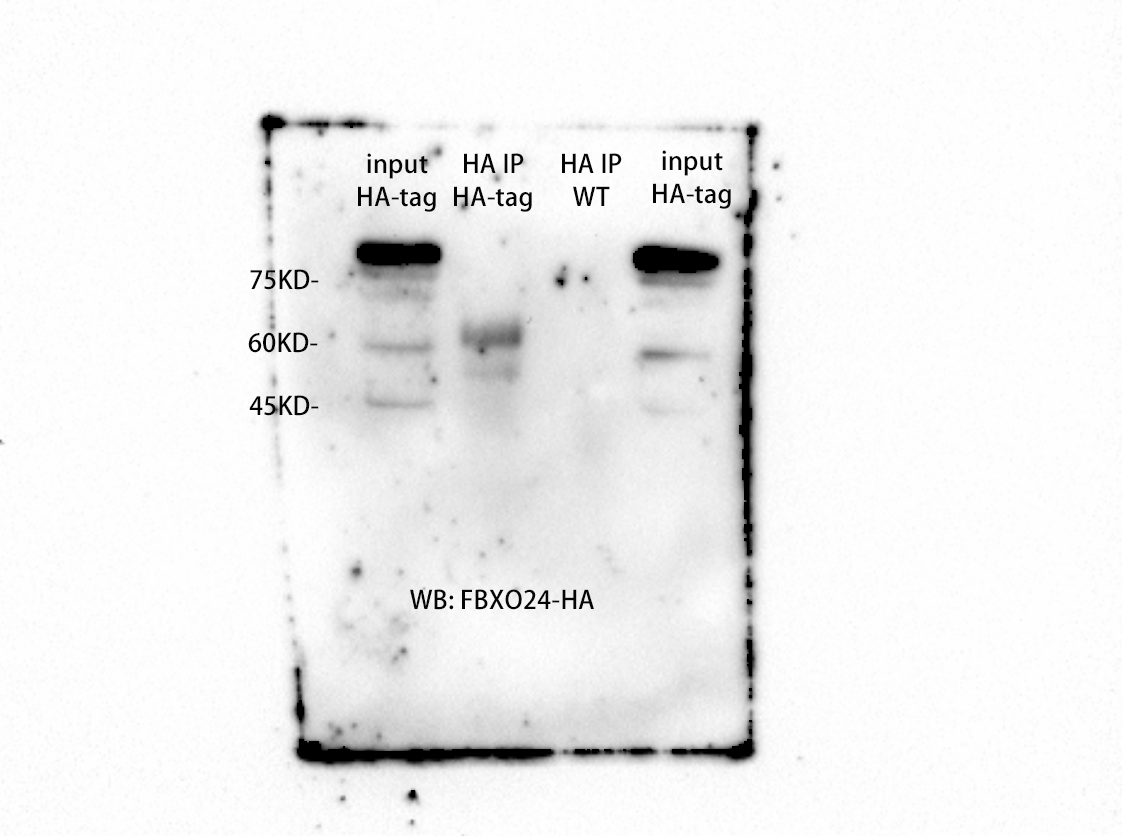

Supplement: Figure 8—source data 1. [file elife-91666-fig8-data1.zip › Figure 8-source data 1/HA-labelled.tif]

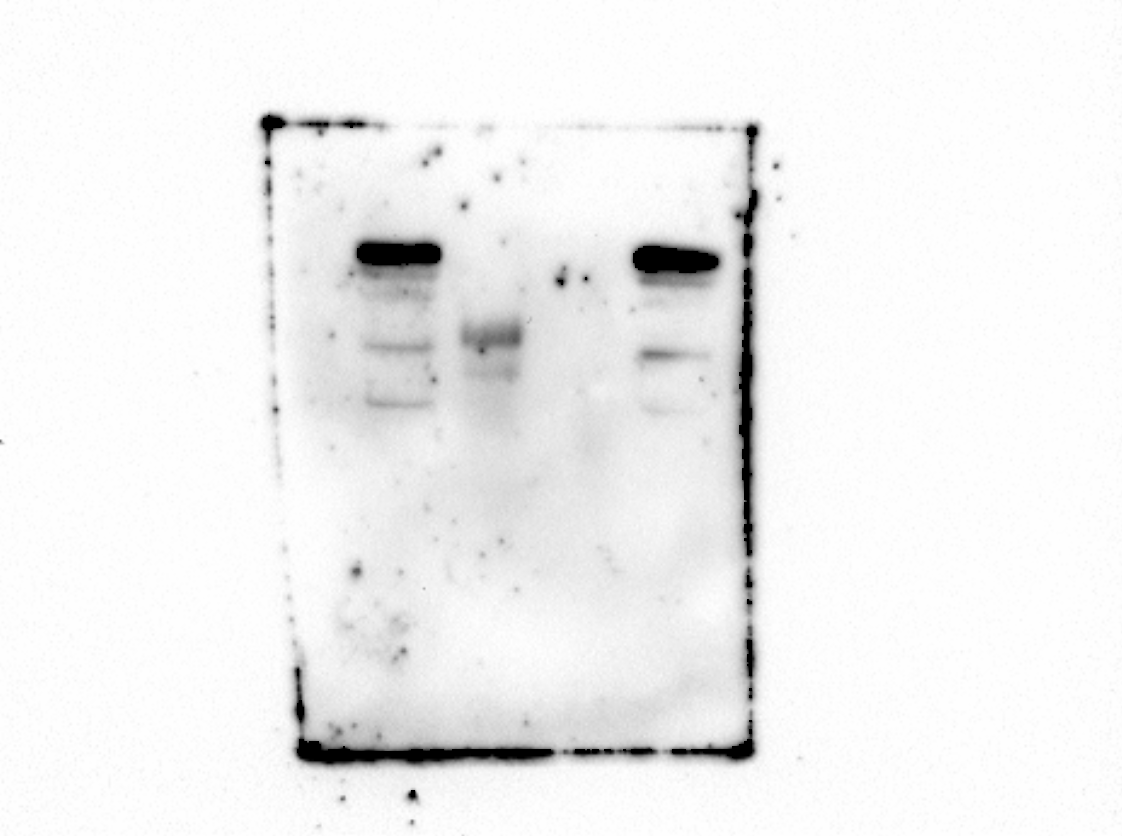

Supplement: Figure 8—source data 1. [file elife-91666-fig8-data1.zip › Figure 8-source data 1/HA-unedited.tif]

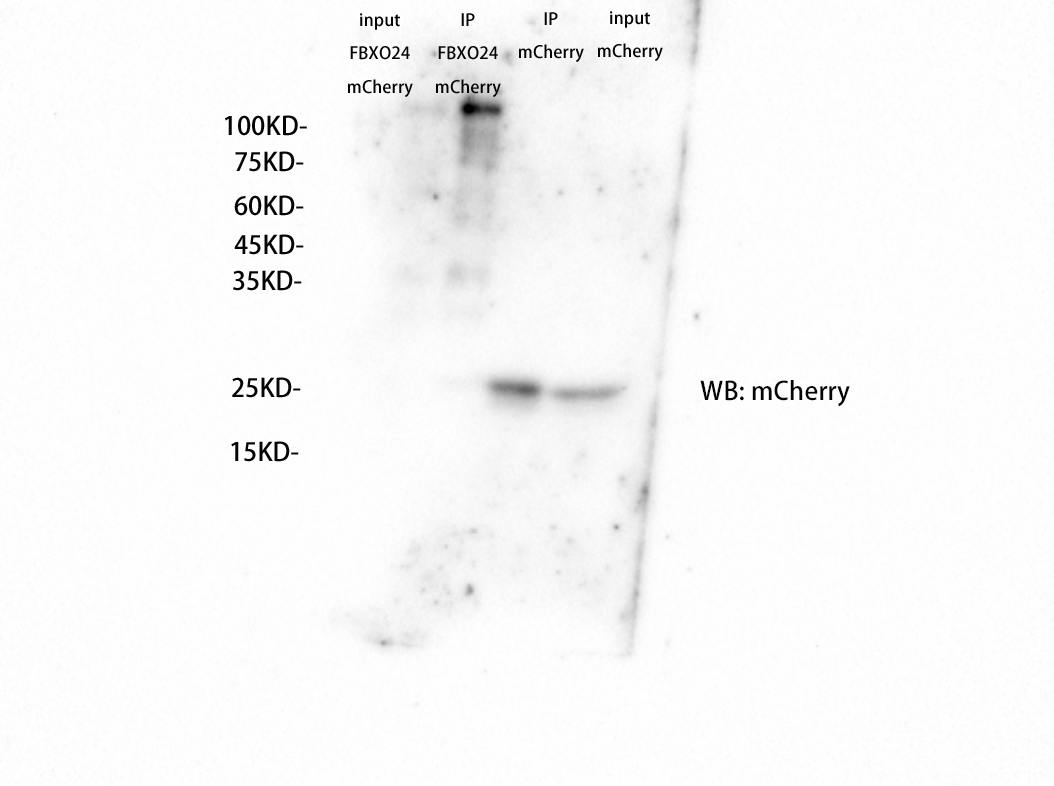

Supplement: Figure 8—source data 1. [file elife-91666-fig8-data1.zip › Figure 8-source data 1/mCherry(2)-labelled.tif]

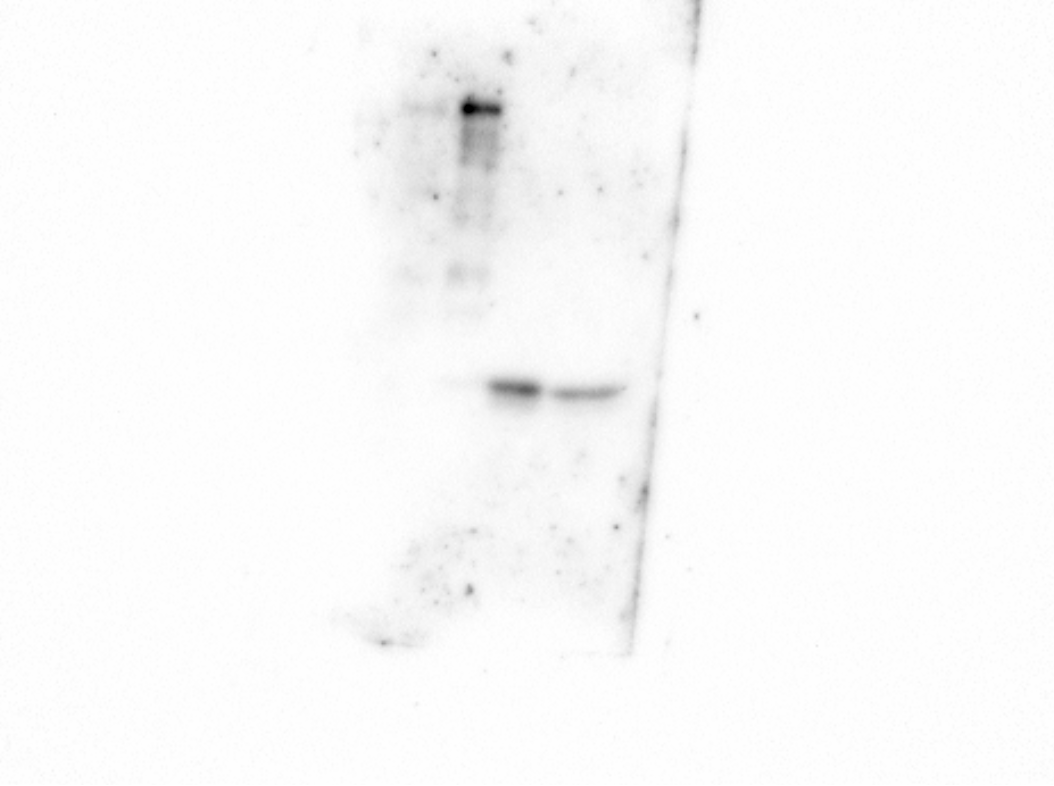

Supplement: Figure 8—source data 1. [file elife-91666-fig8-data1.zip › Figure 8-source data 1/mCherry(2)-unedited.tif]

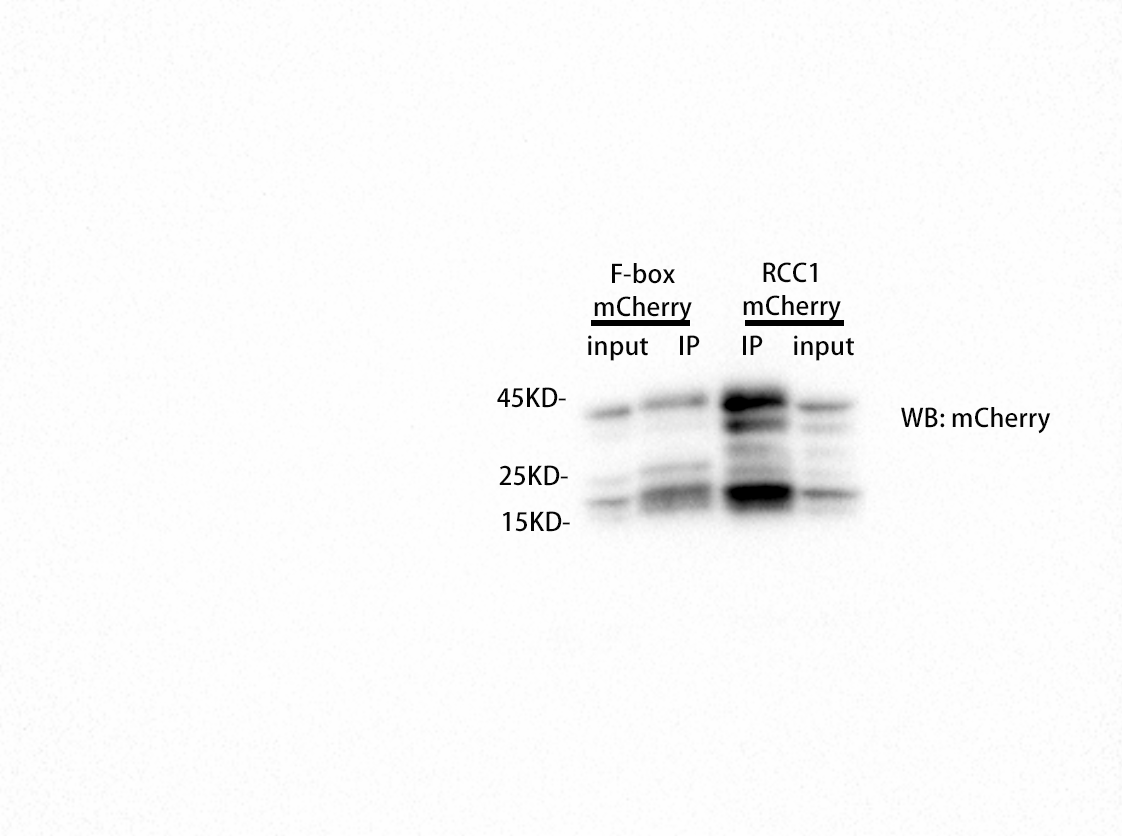

Supplement: Figure 8—source data 1. [file elife-91666-fig8-data1.zip › Figure 8-source data 1/mCherry(3)-labelled.tif]

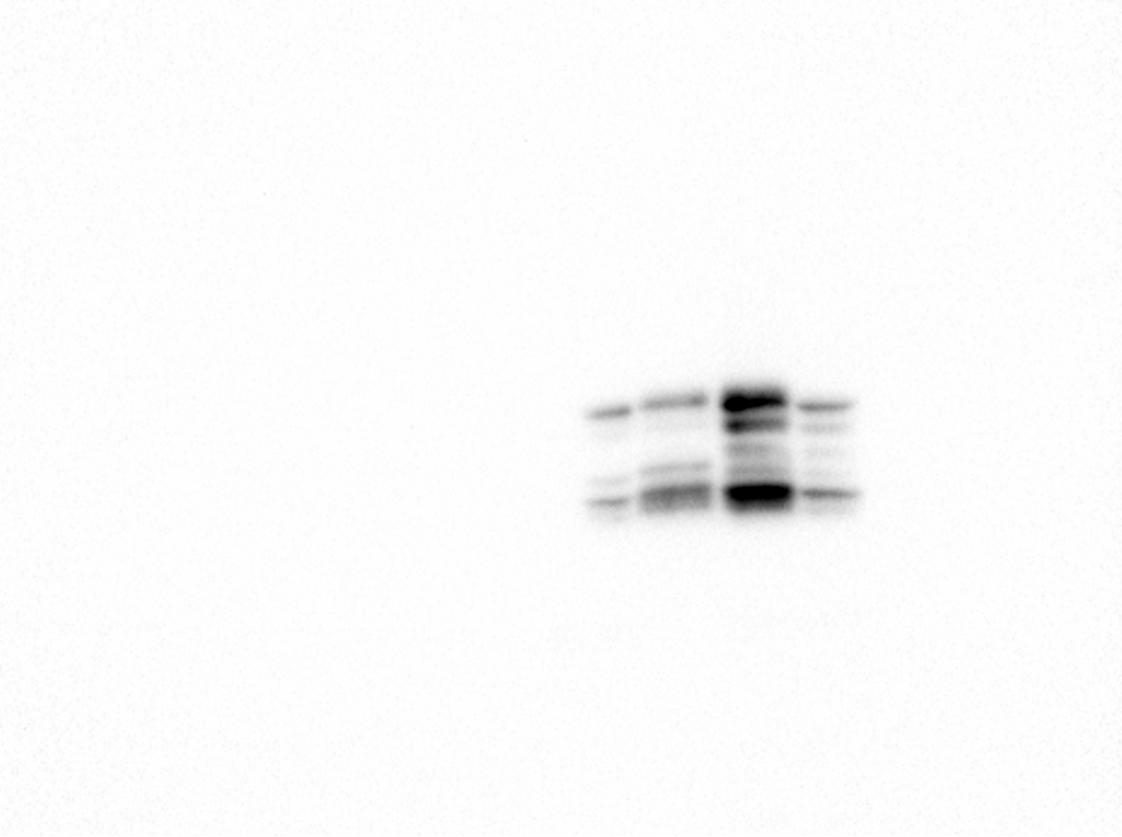

Supplement: Figure 8—source data 1. [file elife-91666-fig8-data1.zip › Figure 8-source data 1/mCherry(3)-unedited.tif]

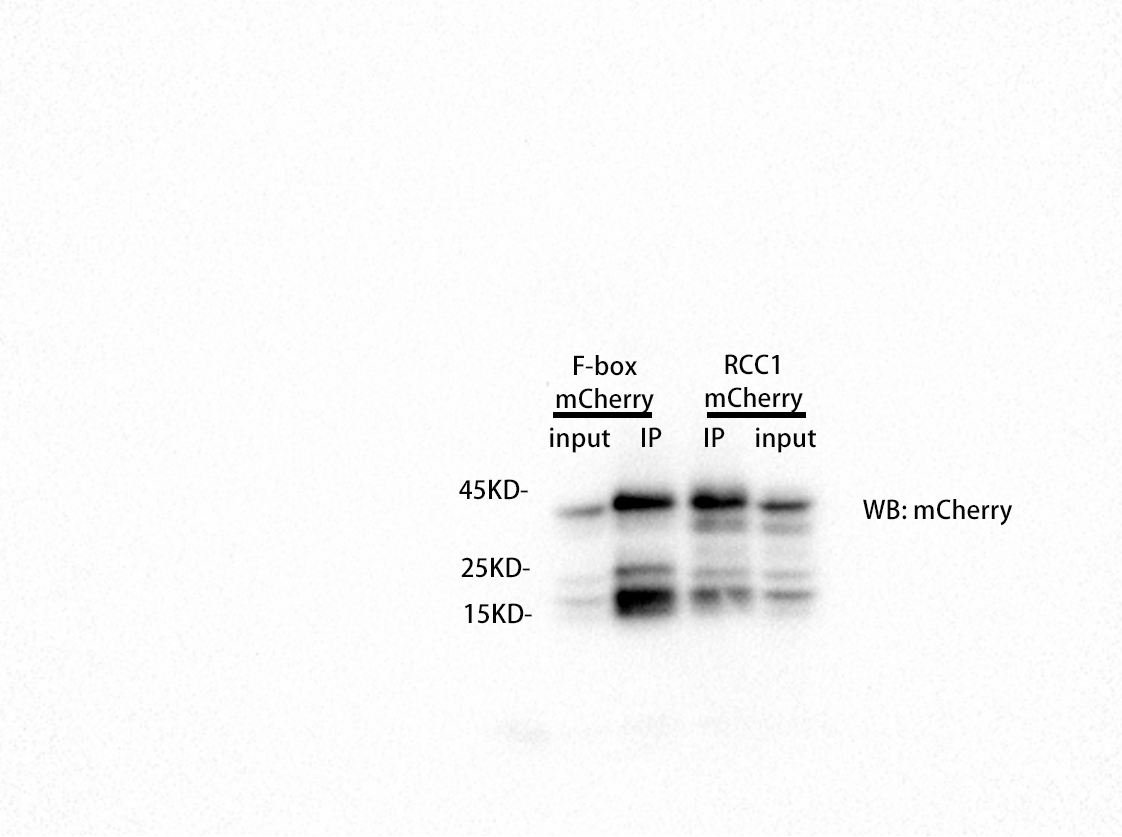

Supplement: Figure 8—source data 1. [file elife-91666-fig8-data1.zip › Figure 8-source data 1/mCherry(4)-labelled.tif]

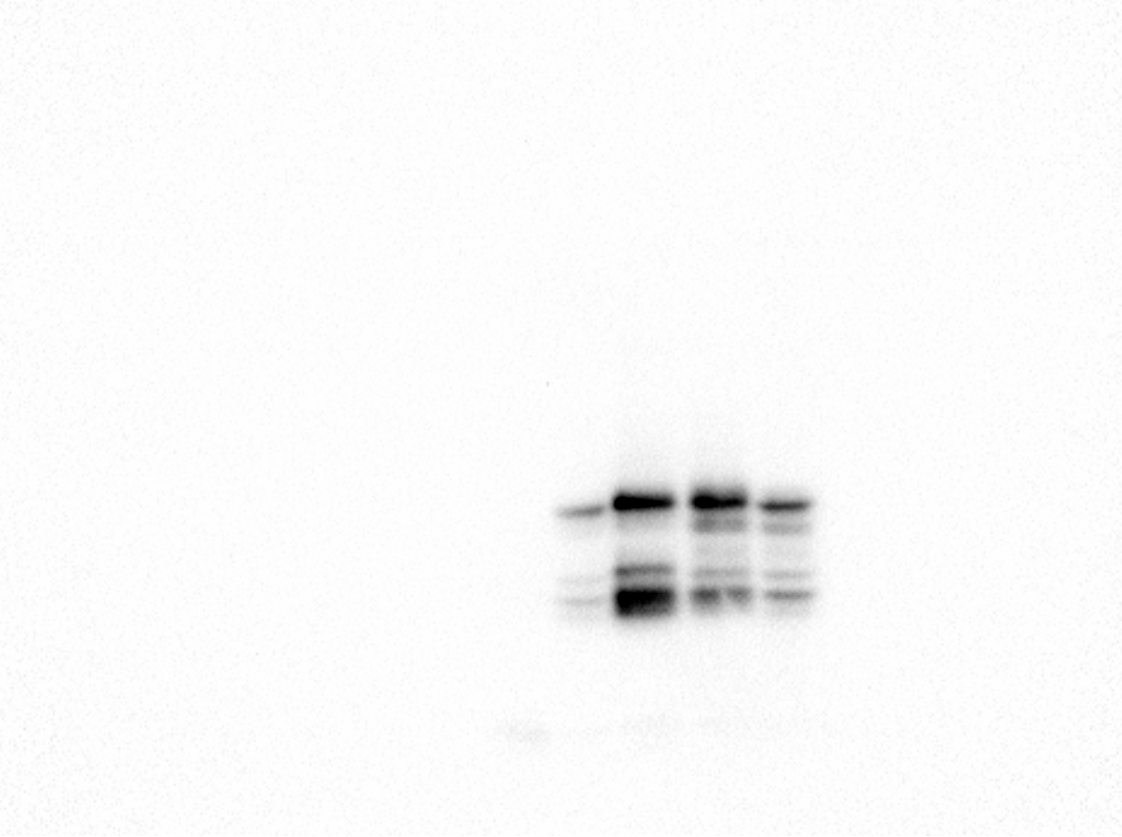

Supplement: Figure 8—source data 1. [file elife-91666-fig8-data1.zip › Figure 8-source data 1/mCherry(4)-unedited.tif]

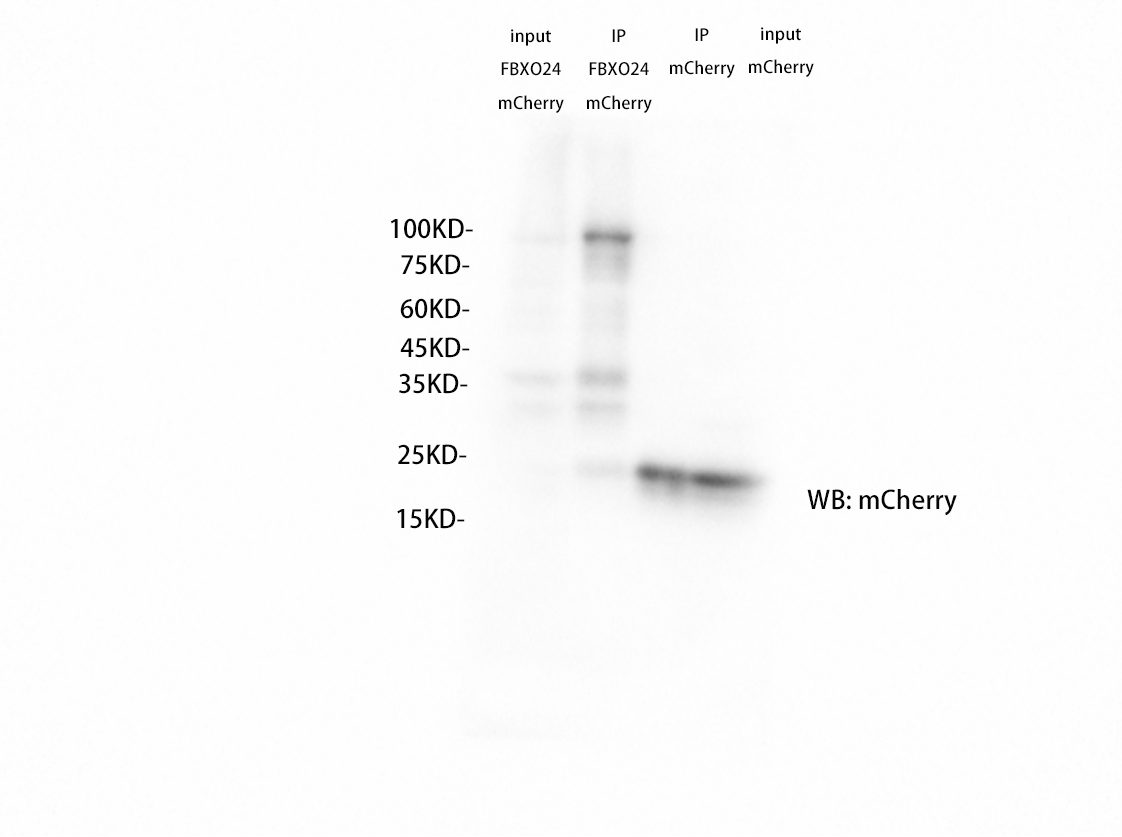

Supplement: Figure 8—source data 1. [file elife-91666-fig8-data1.zip › Figure 8-source data 1/mCherry-labelled.tif]

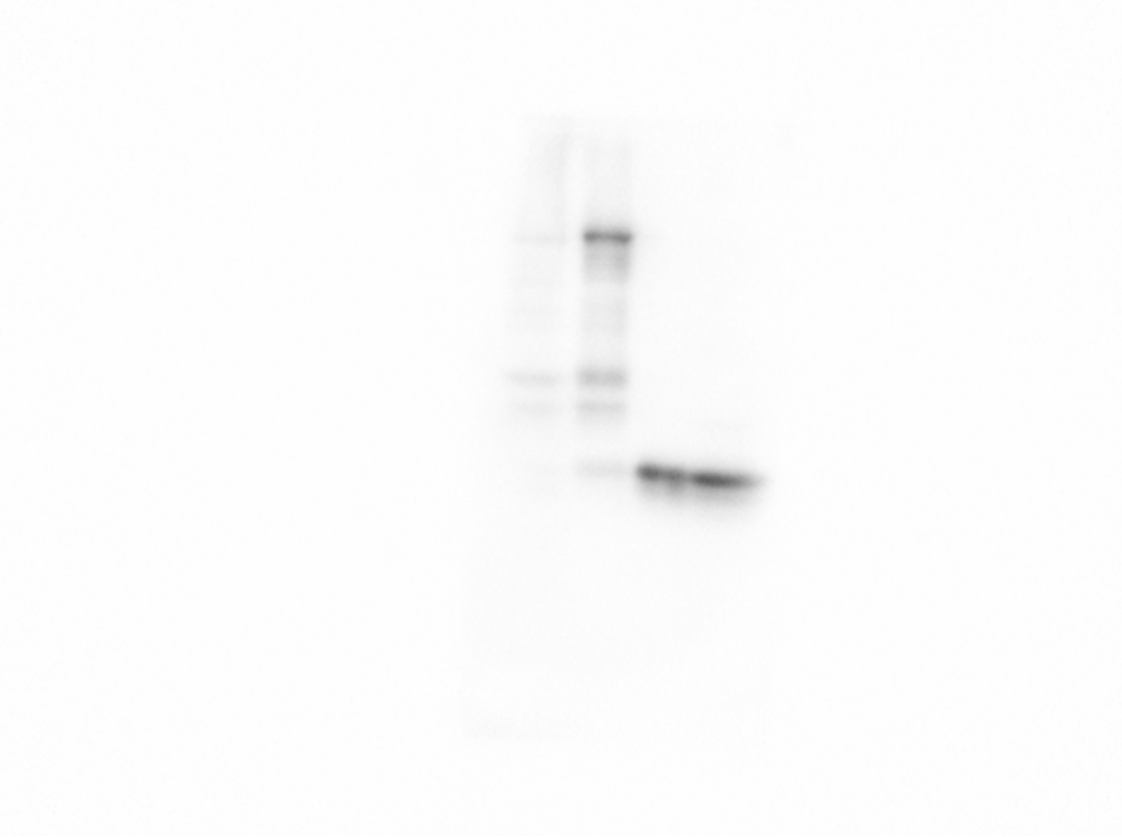

Supplement: Figure 8—source data 1. [file elife-91666-fig8-data1.zip › Figure 8-source data 1/mCherry-unedited.tif]

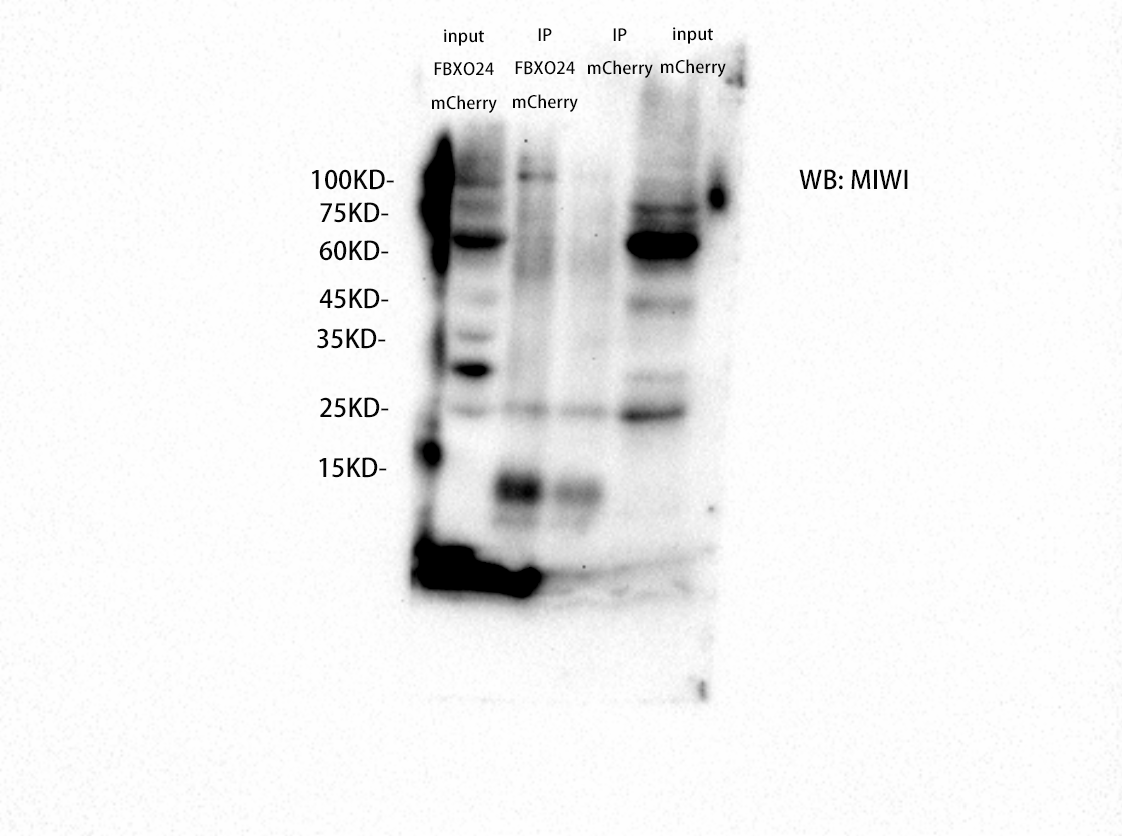

Supplement: Figure 8—source data 1. [file elife-91666-fig8-data1.zip › Figure 8-source data 1/MIWI(1)-labelled.tif]

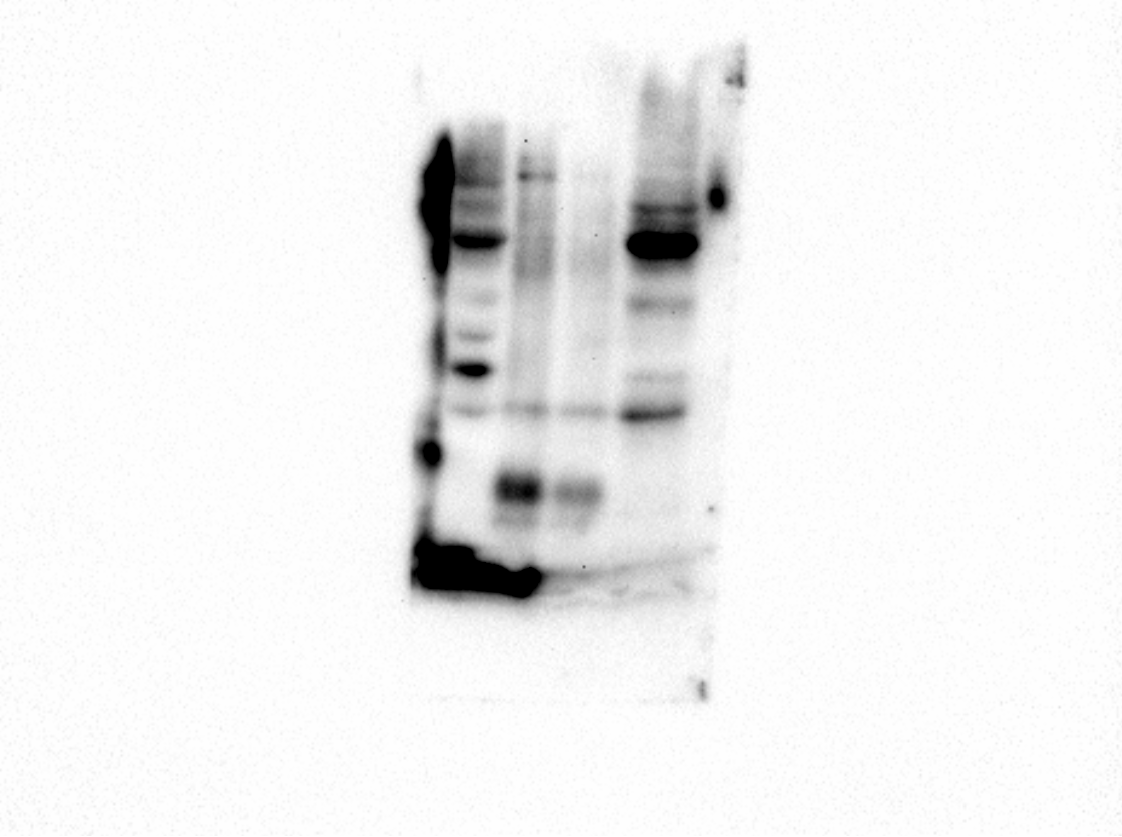

Supplement: Figure 8—source data 1. [file elife-91666-fig8-data1.zip › Figure 8-source data 1/MIWI(1)-unedited.tif]

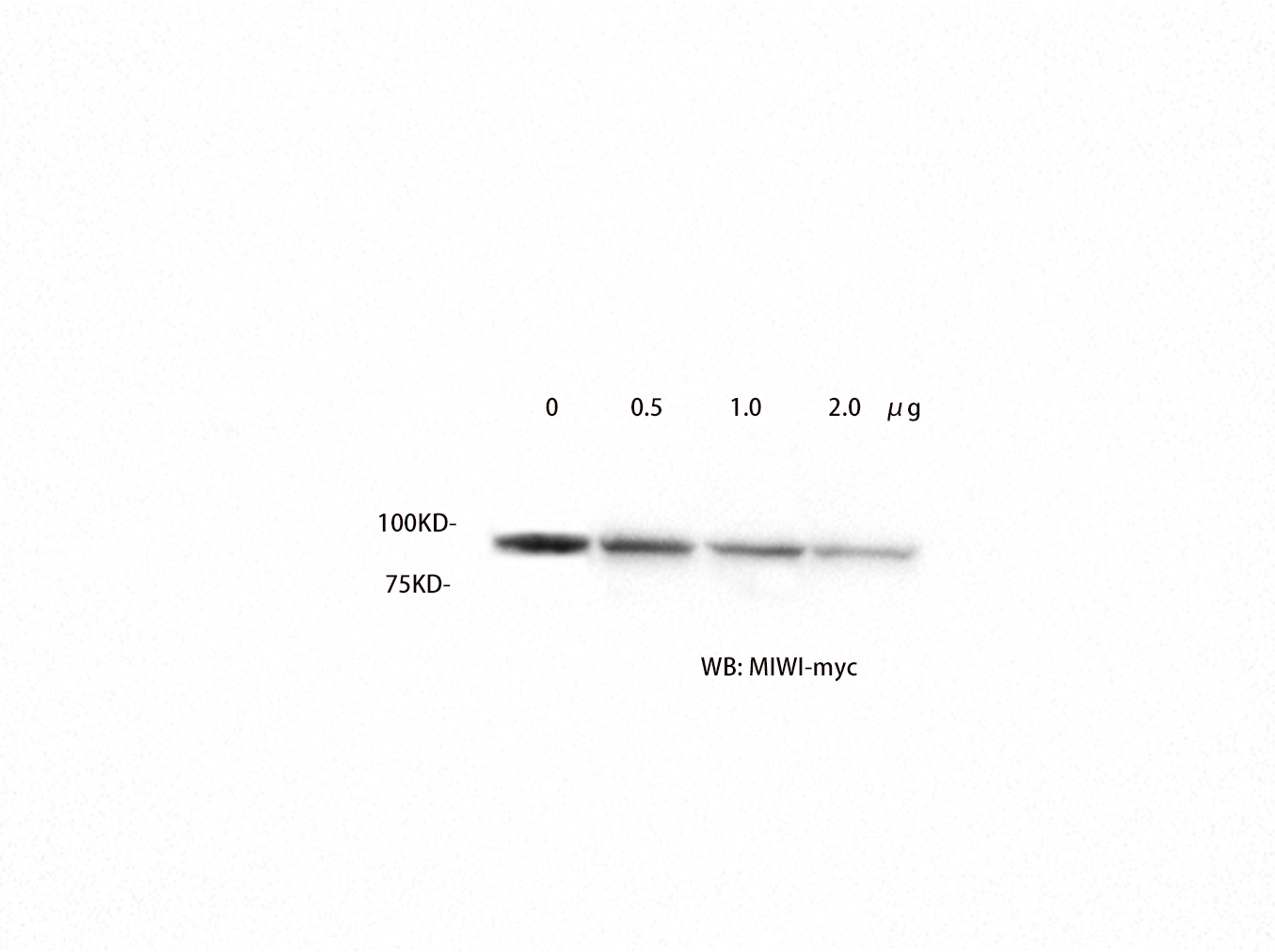

Supplement: Figure 8—source data 1. [file elife-91666-fig8-data1.zip › Figure 8-source data 1/MIWI(2)-myc-labelled.tif]

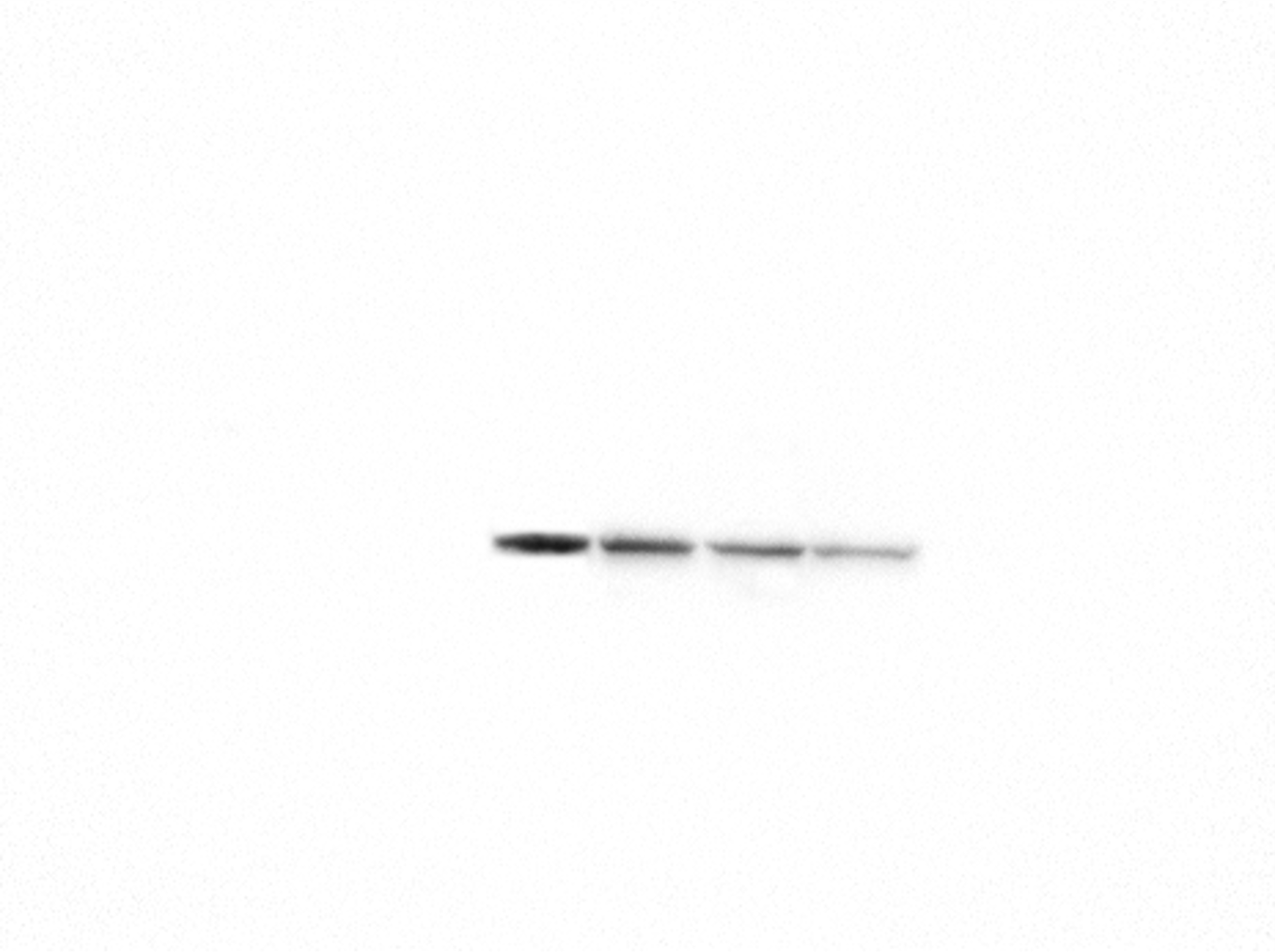

Supplement: Figure 8—source data 1. [file elife-91666-fig8-data1.zip › Figure 8-source data 1/MIWI(2)-myc-unedited.tif]

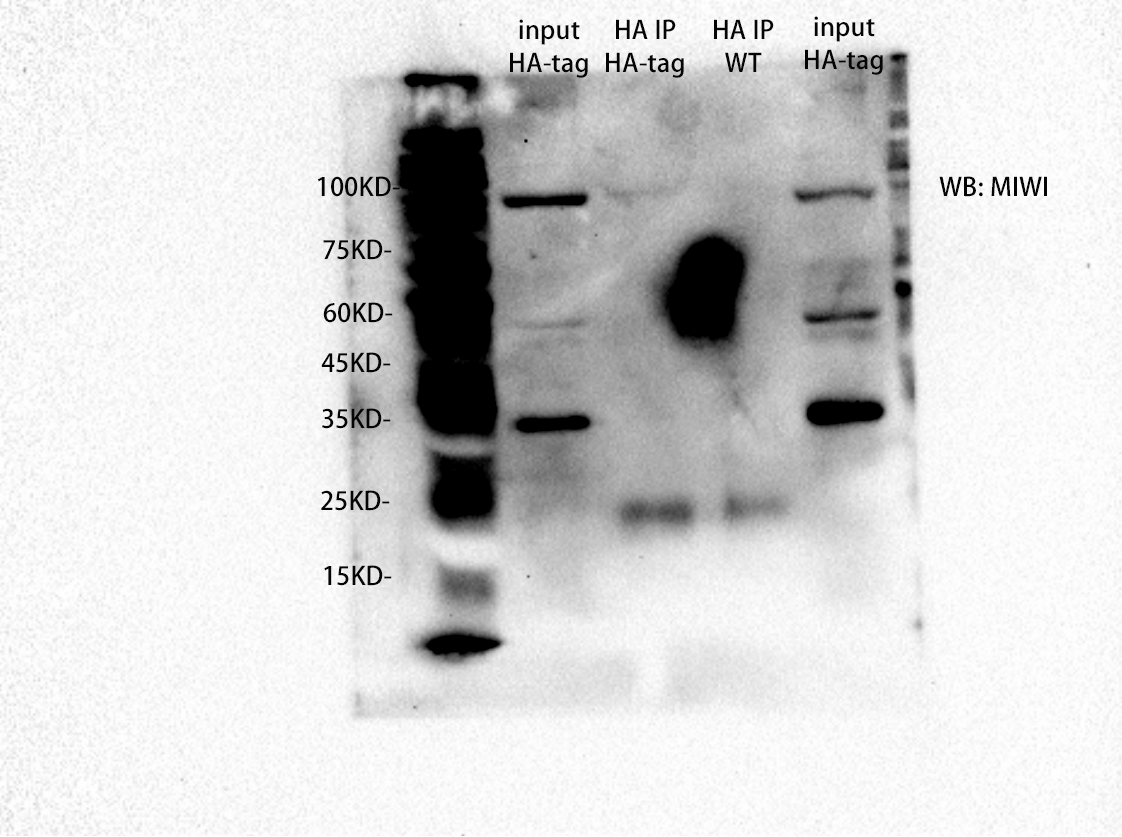

Supplement: Figure 8—source data 1. [file elife-91666-fig8-data1.zip › Figure 8-source data 1/MIWI(3)-labelled.tif]

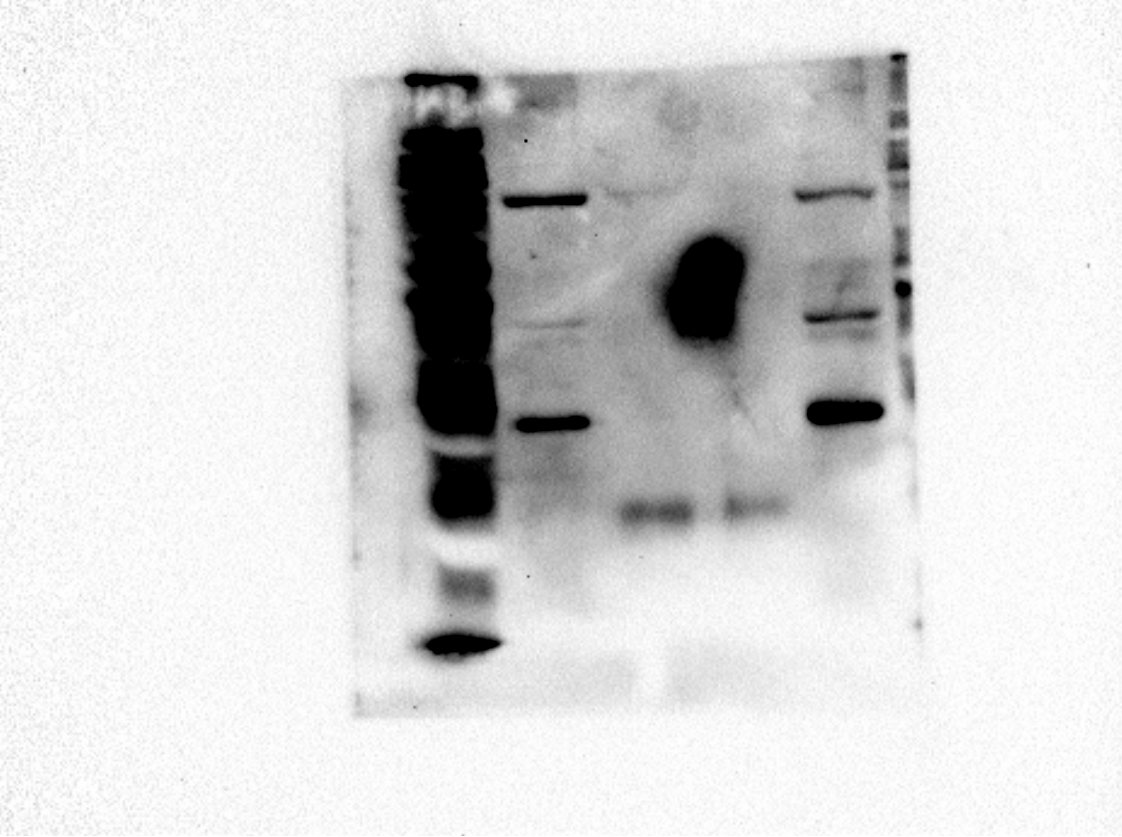

Supplement: Figure 8—source data 1. [file elife-91666-fig8-data1.zip › Figure 8-source data 1/MIWI(3)-unedited.tif]

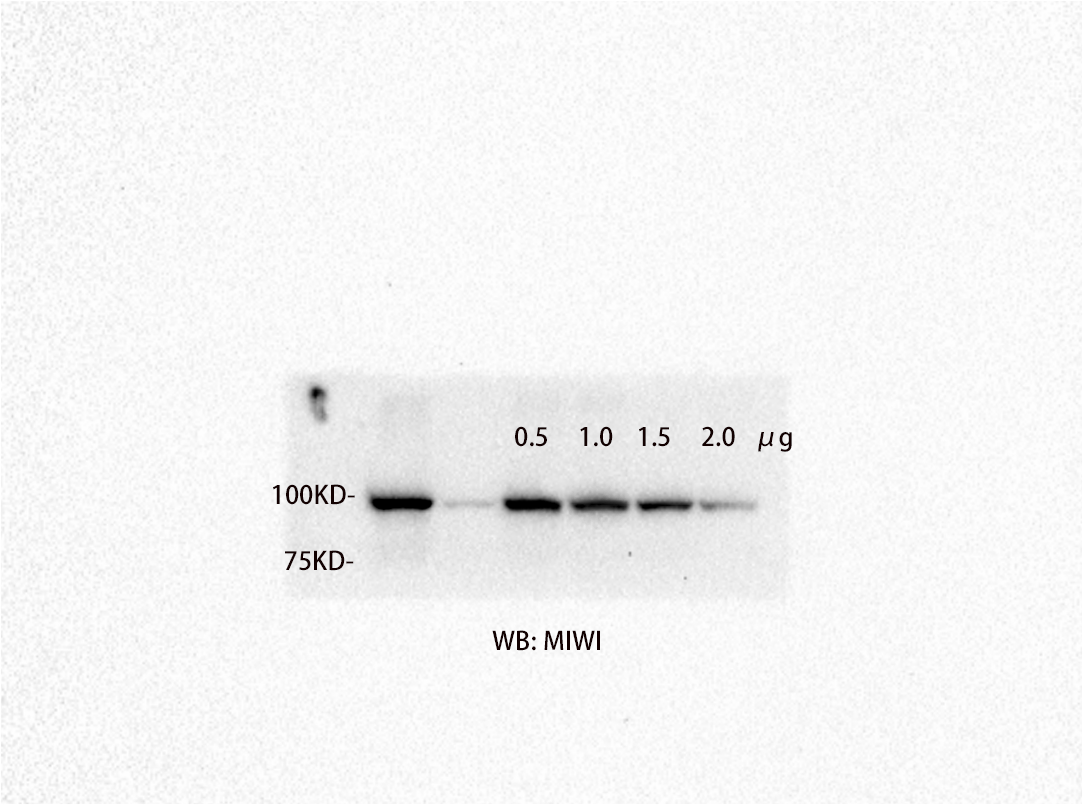

Supplement: Figure 8—source data 1. [file elife-91666-fig8-data1.zip › Figure 8-source data 1/MIWI(4)-labelled.tif]

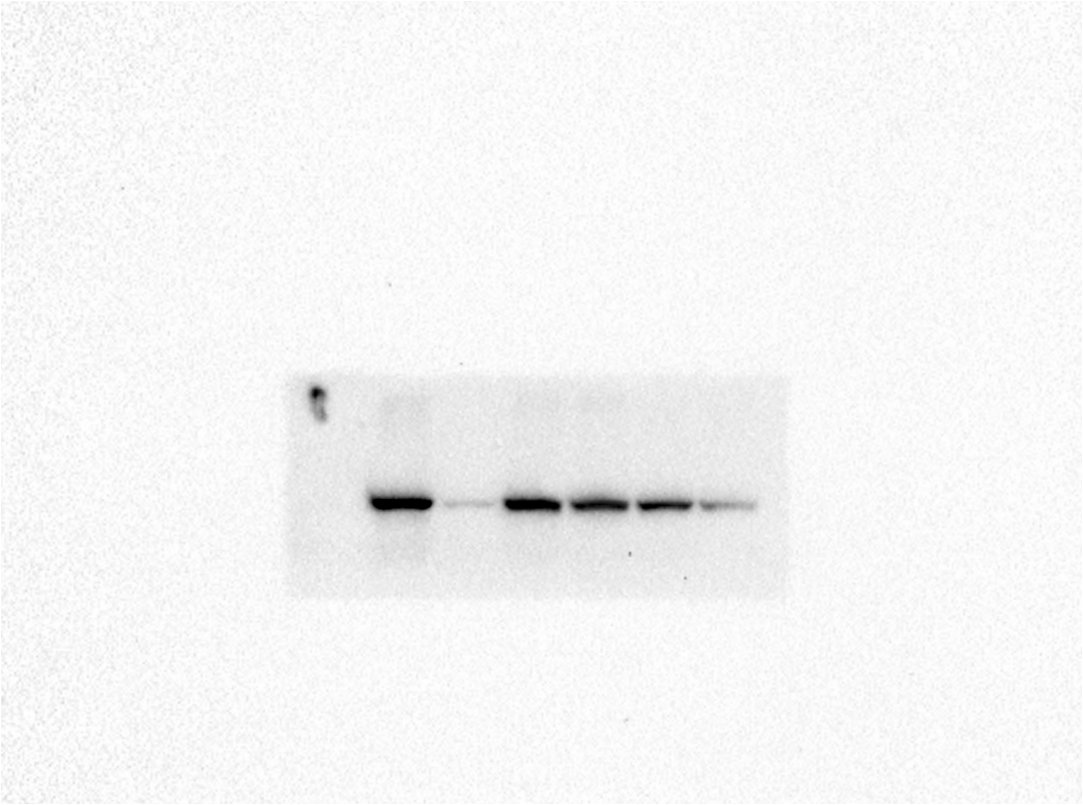

Supplement: Figure 8—source data 1. [file elife-91666-fig8-data1.zip › Figure 8-source data 1/MIWI(4)-unedited.tif]

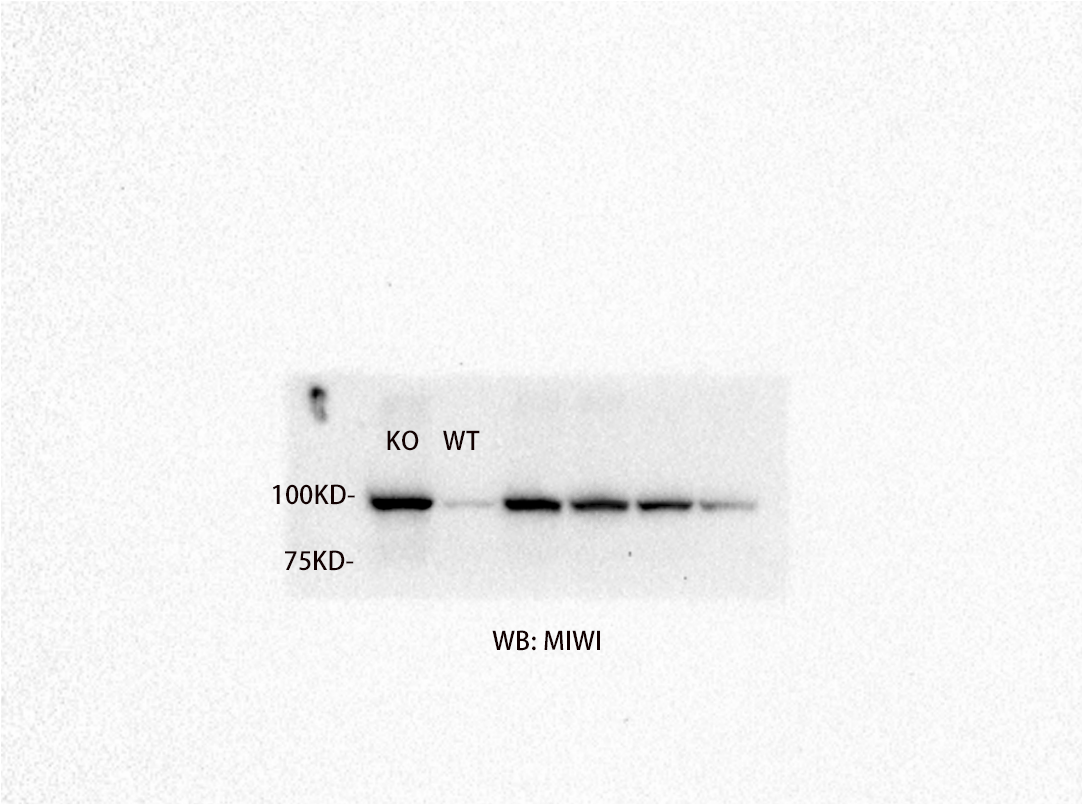

Supplement: Figure 8—source data 1. [file elife-91666-fig8-data1.zip › Figure 8-source data 1/MIWI(5)-labelled.tif]

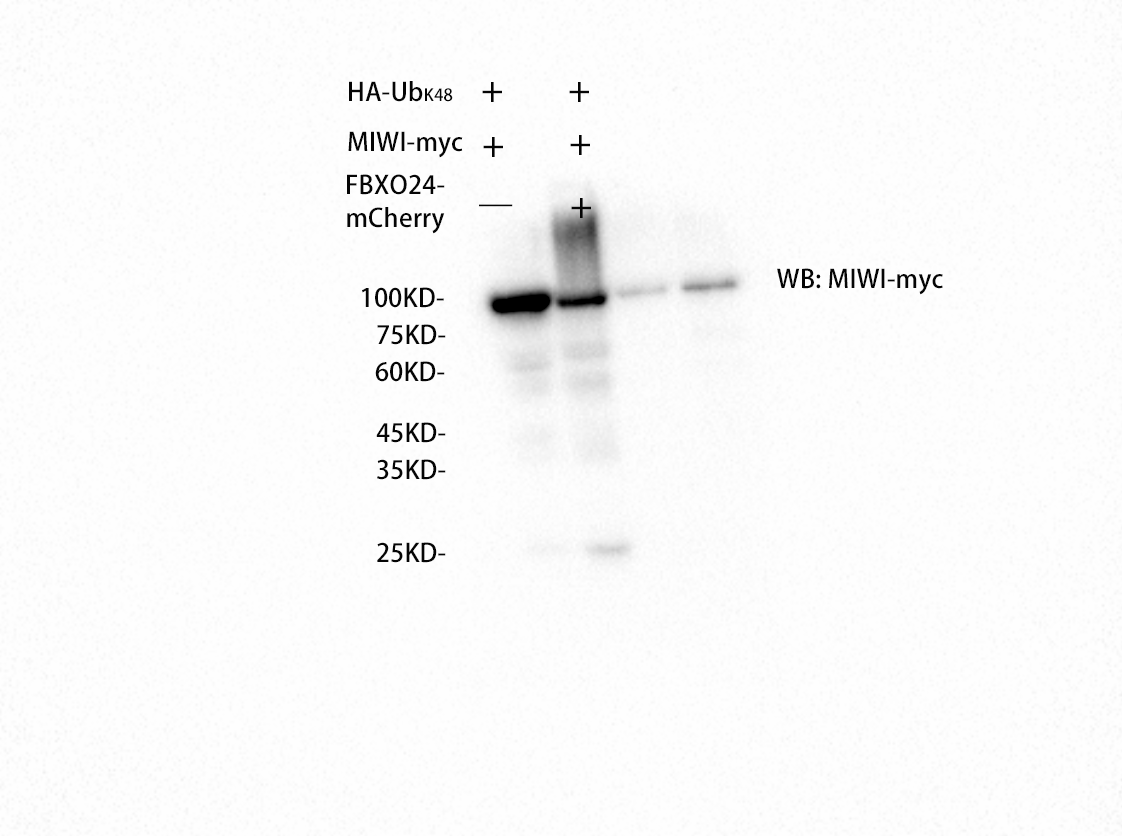

Supplement: Figure 8—source data 1. [file elife-91666-fig8-data1.zip › Figure 8-source data 1/MIWI-myc(2)-labelled.tif]

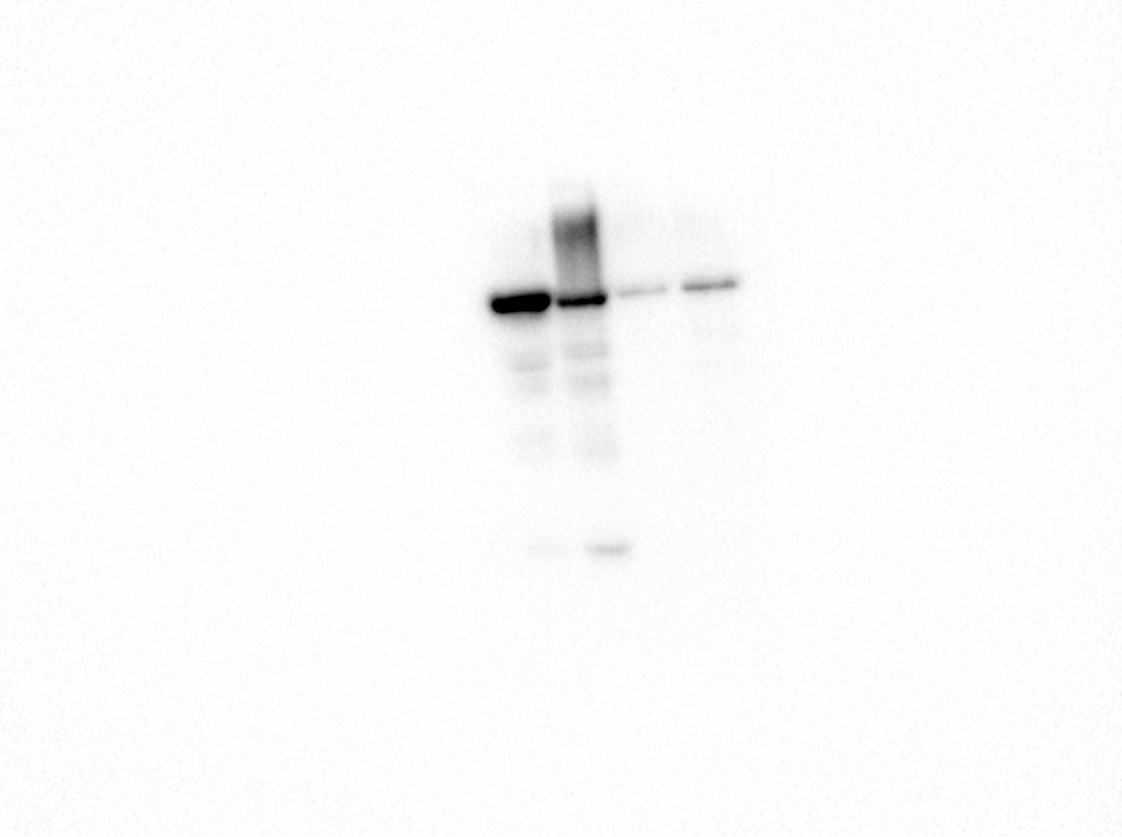

Supplement: Figure 8—source data 1. [file elife-91666-fig8-data1.zip › Figure 8-source data 1/MIWI-myc(2)-unedited.tif]

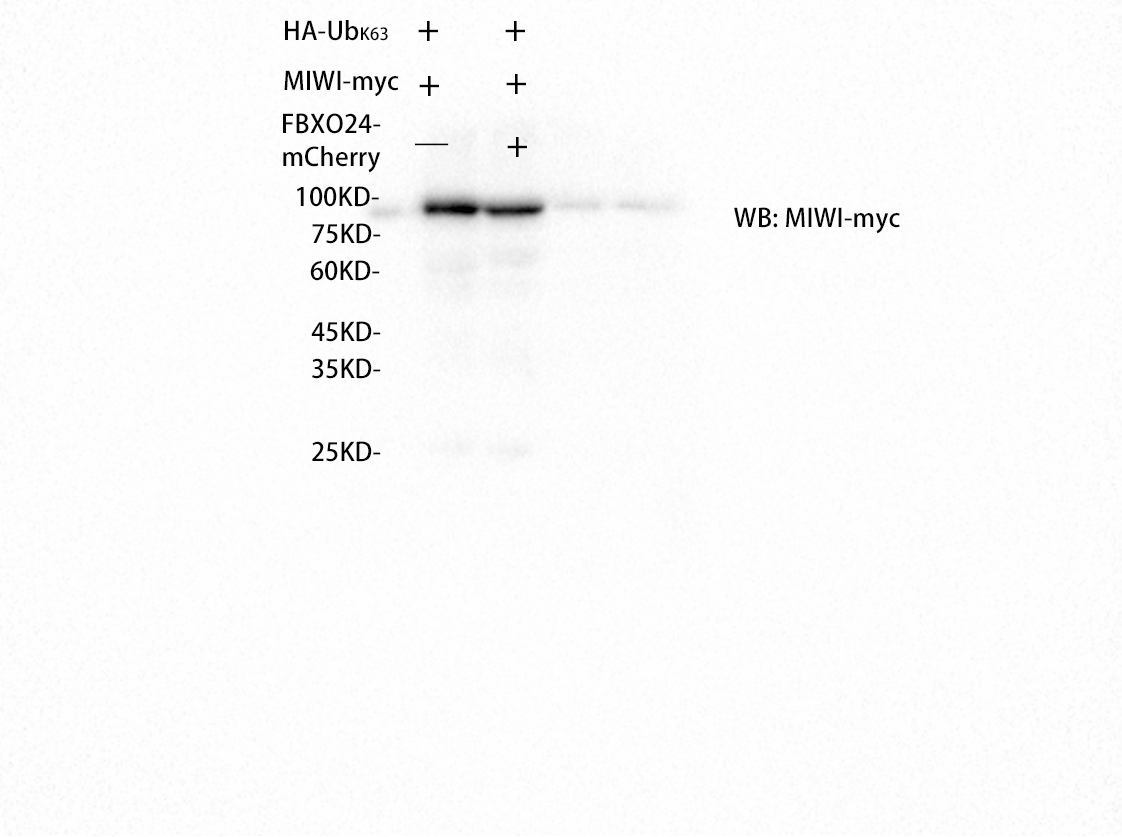

Supplement: Figure 8—source data 1. [file elife-91666-fig8-data1.zip › Figure 8-source data 1/MIWI-myc(3)-labelled.tif]

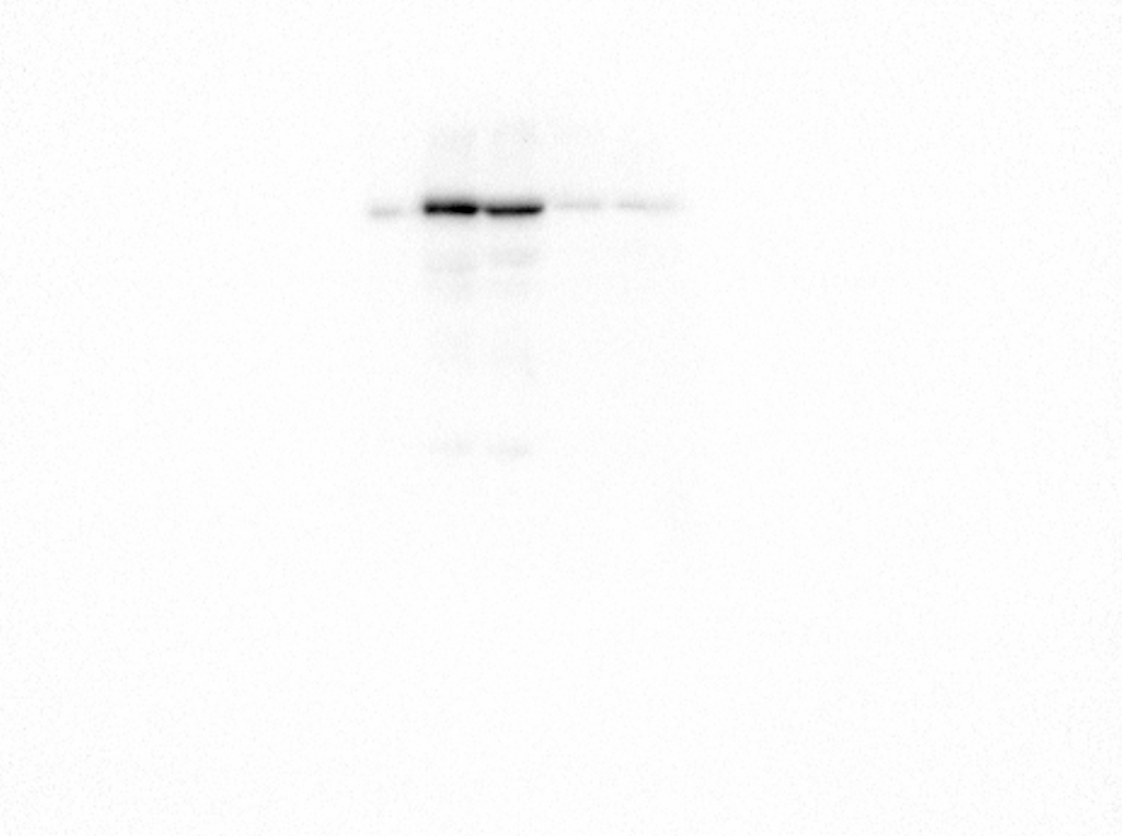

Supplement: Figure 8—source data 1. [file elife-91666-fig8-data1.zip › Figure 8-source data 1/MIWI-myc(3)-unedited.tif]

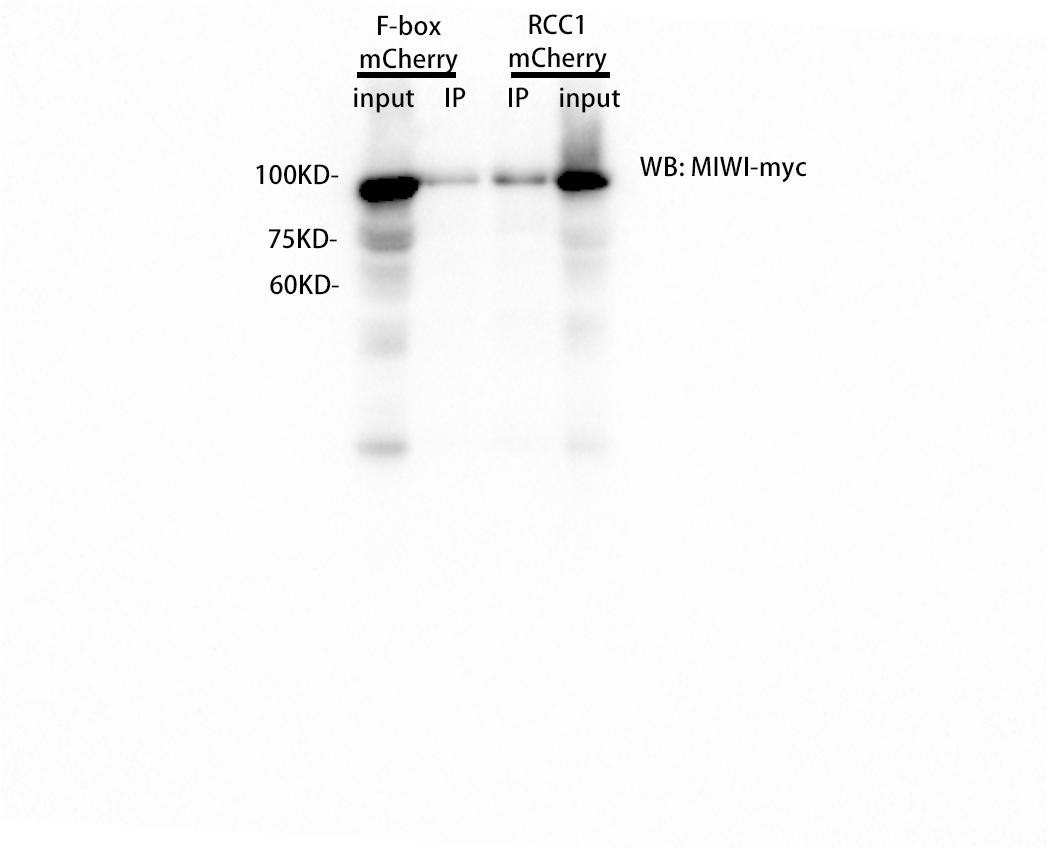

Supplement: Figure 8—source data 1. [file elife-91666-fig8-data1.zip › Figure 8-source data 1/MIWI-myc(4)-labelled.tif]

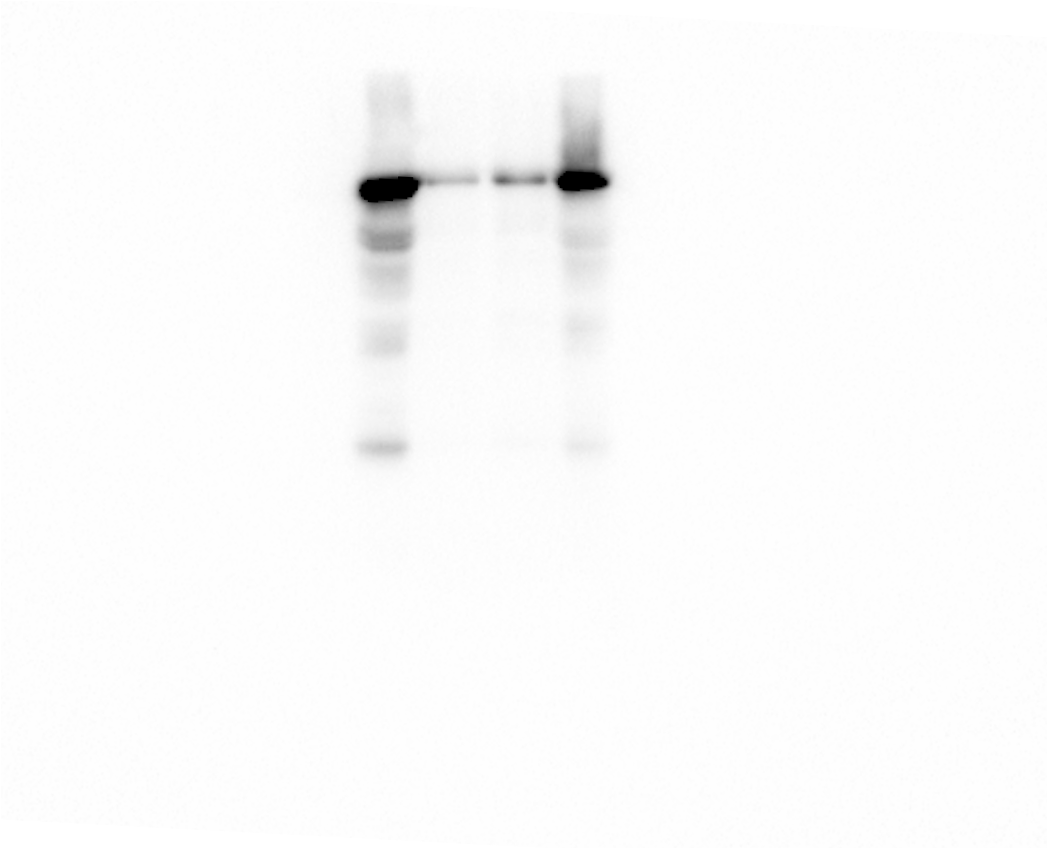

Supplement: Figure 8—source data 1. [file elife-91666-fig8-data1.zip › Figure 8-source data 1/MIWI-myc(4)-unedited.tif]

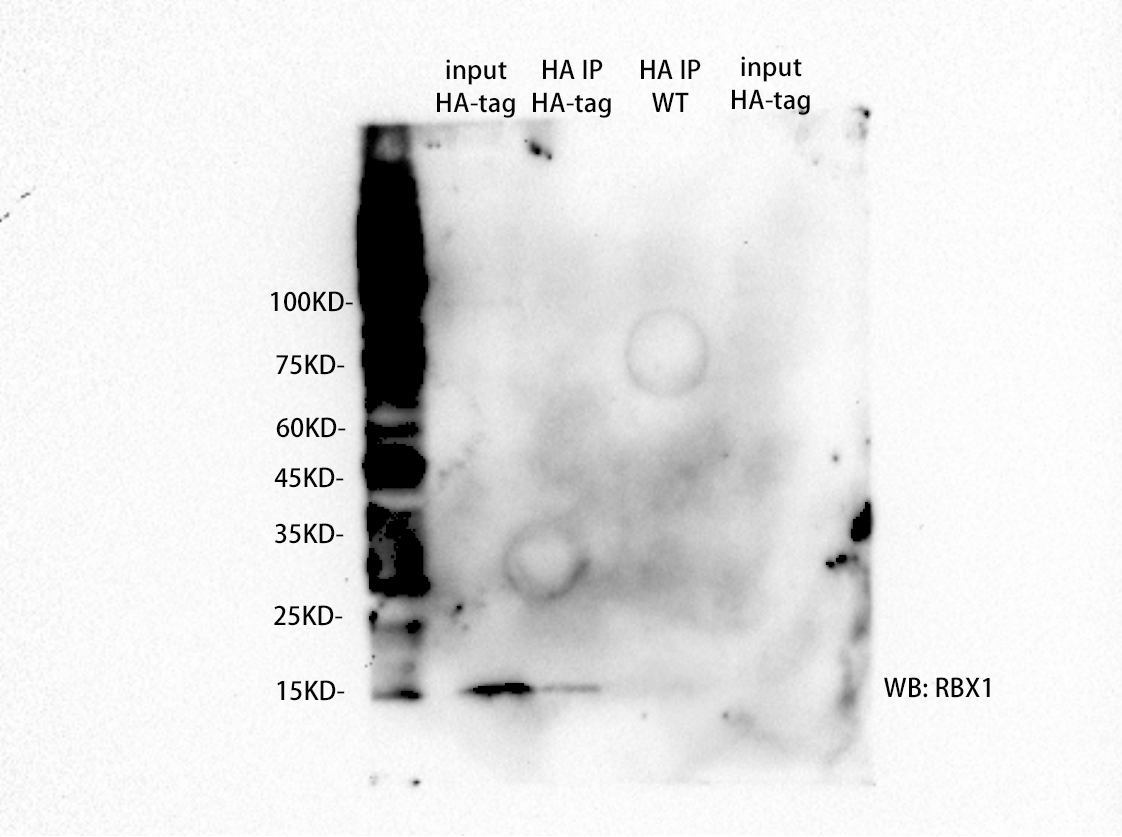

Supplement: Figure 8—source data 1. [file elife-91666-fig8-data1.zip › Figure 8-source data 1/RBX1-labelled.tif]

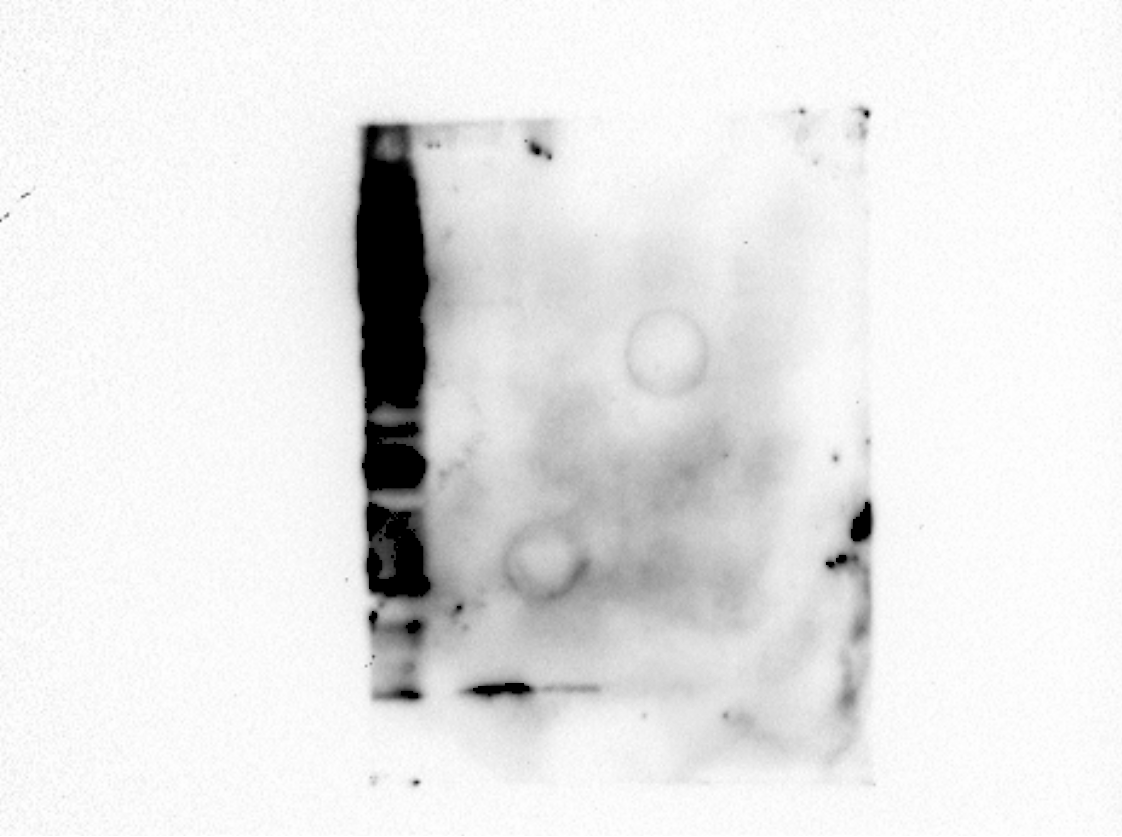

Supplement: Figure 8—source data 1. [file elife-91666-fig8-data1.zip › Figure 8-source data 1/RBX1-unedited.tif]

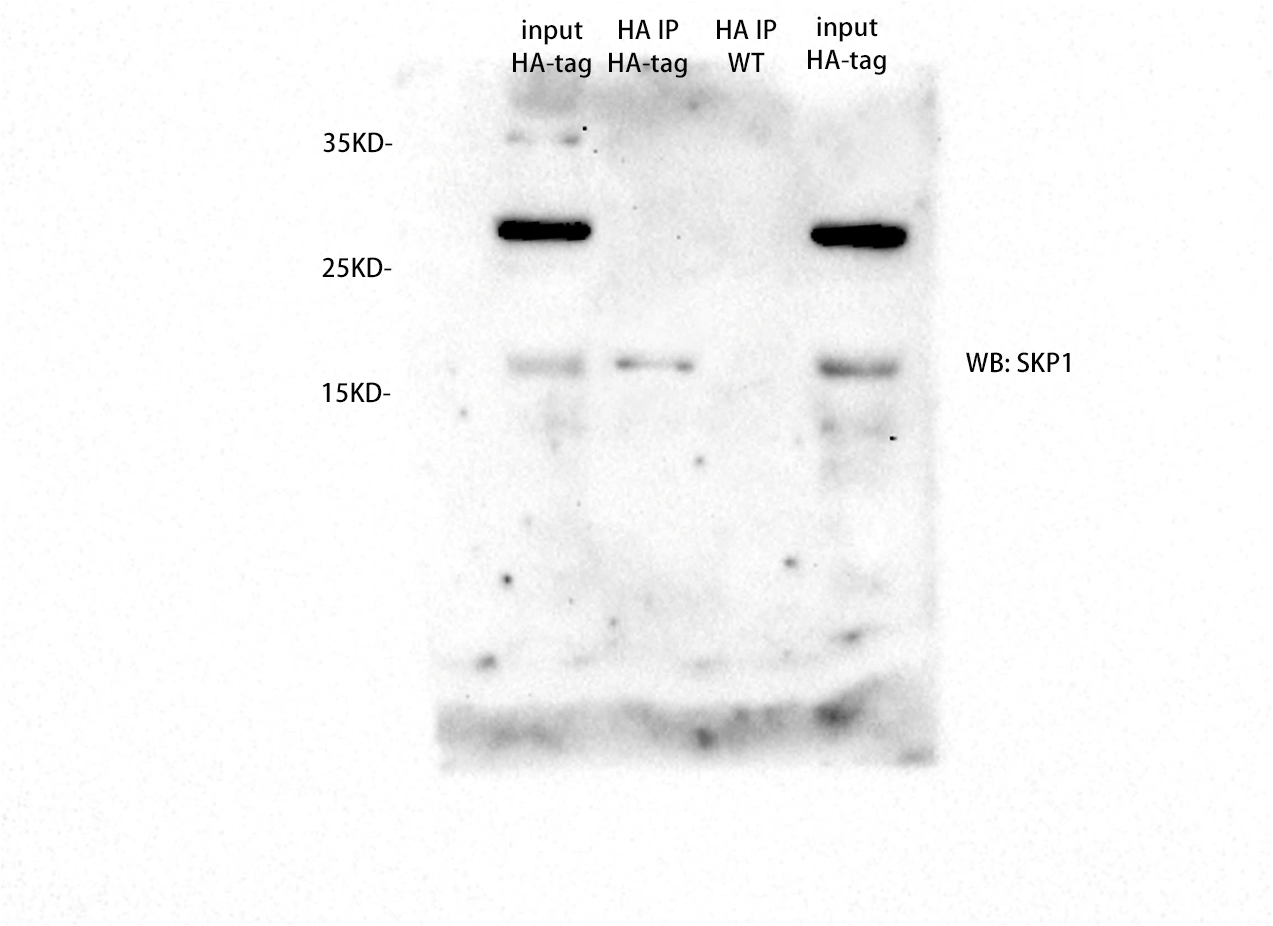

Supplement: Figure 8—source data 1. [file elife-91666-fig8-data1.zip › Figure 8-source data 1/SKP1-labelled.tif]

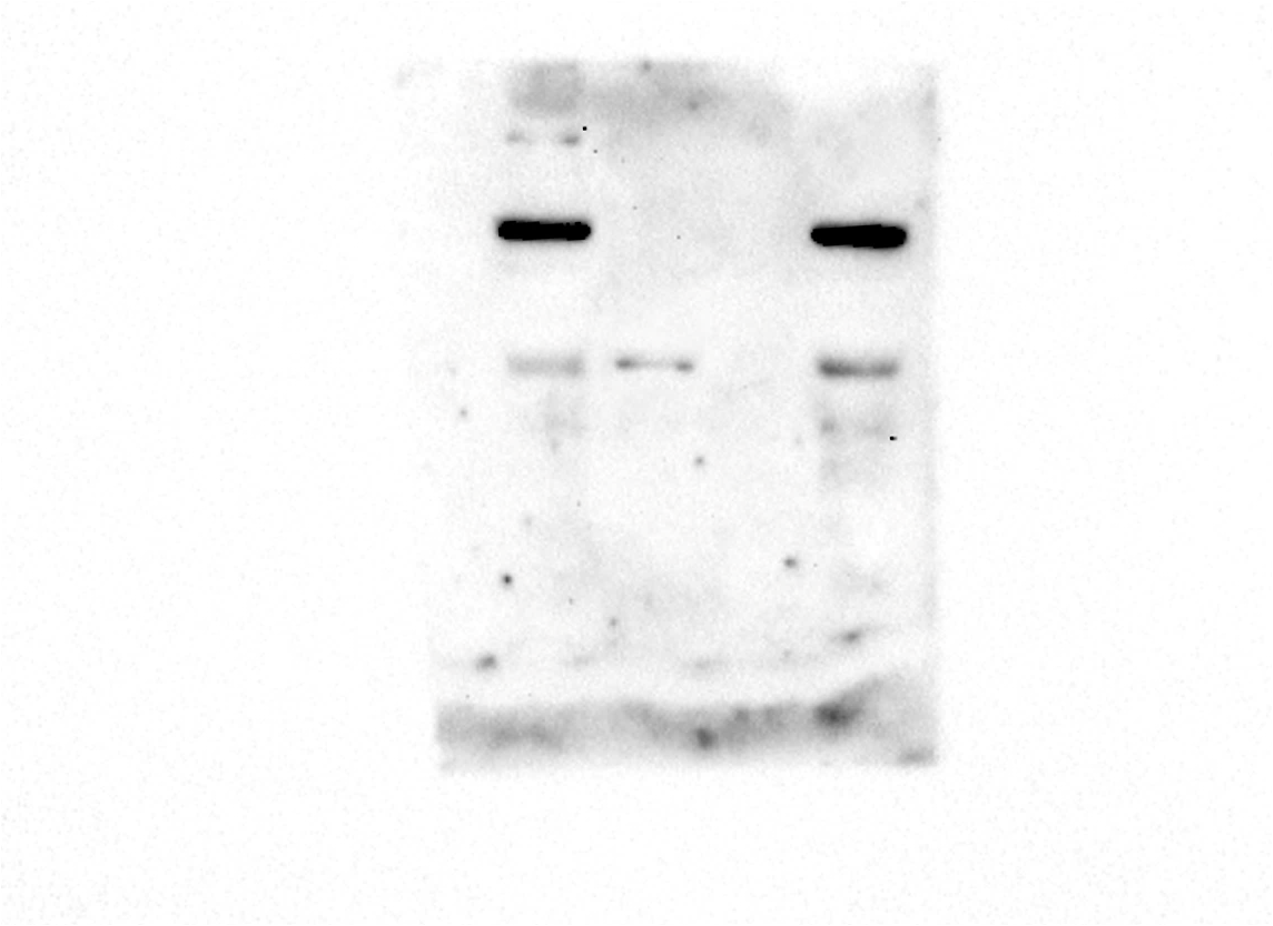

Supplement: Figure 8—source data 1. [file elife-91666-fig8-data1.zip › Figure 8-source data 1/SKP1-unedited.tif]

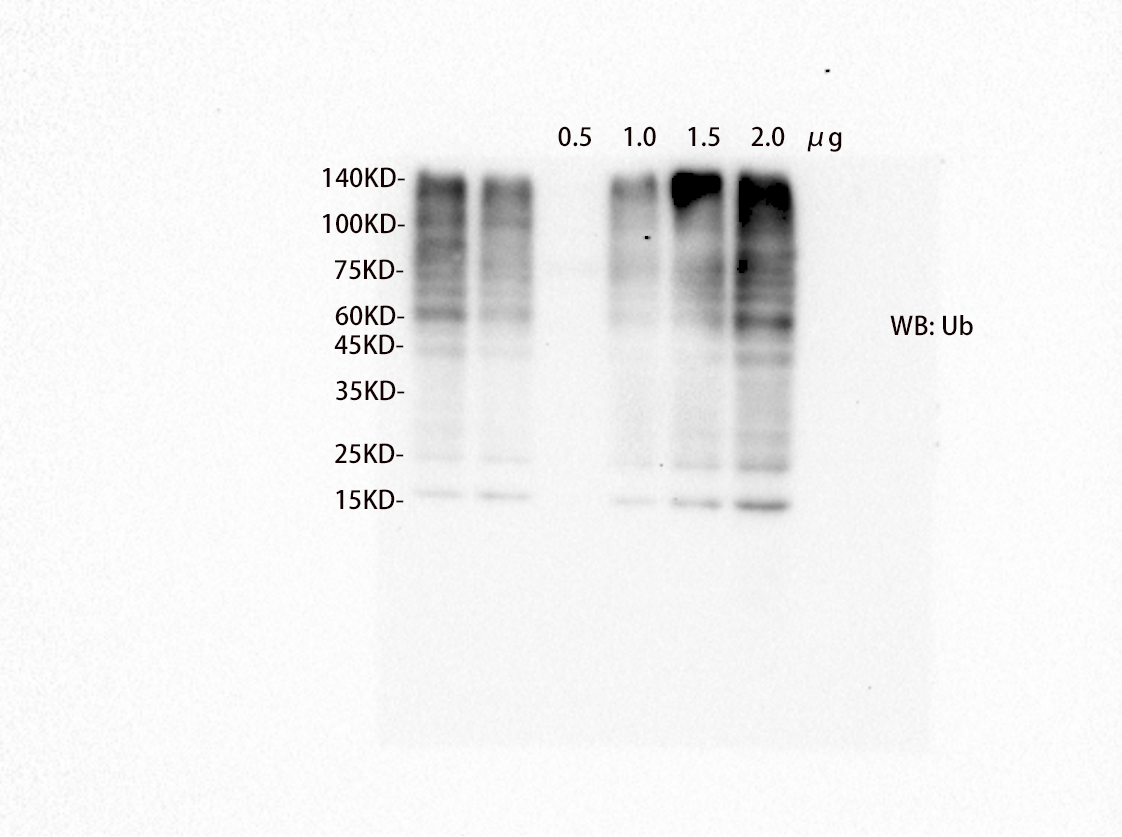

Supplement: Figure 8—source data 1. [file elife-91666-fig8-data1.zip › Figure 8-source data 1/Ub(1)-labelled.tif]

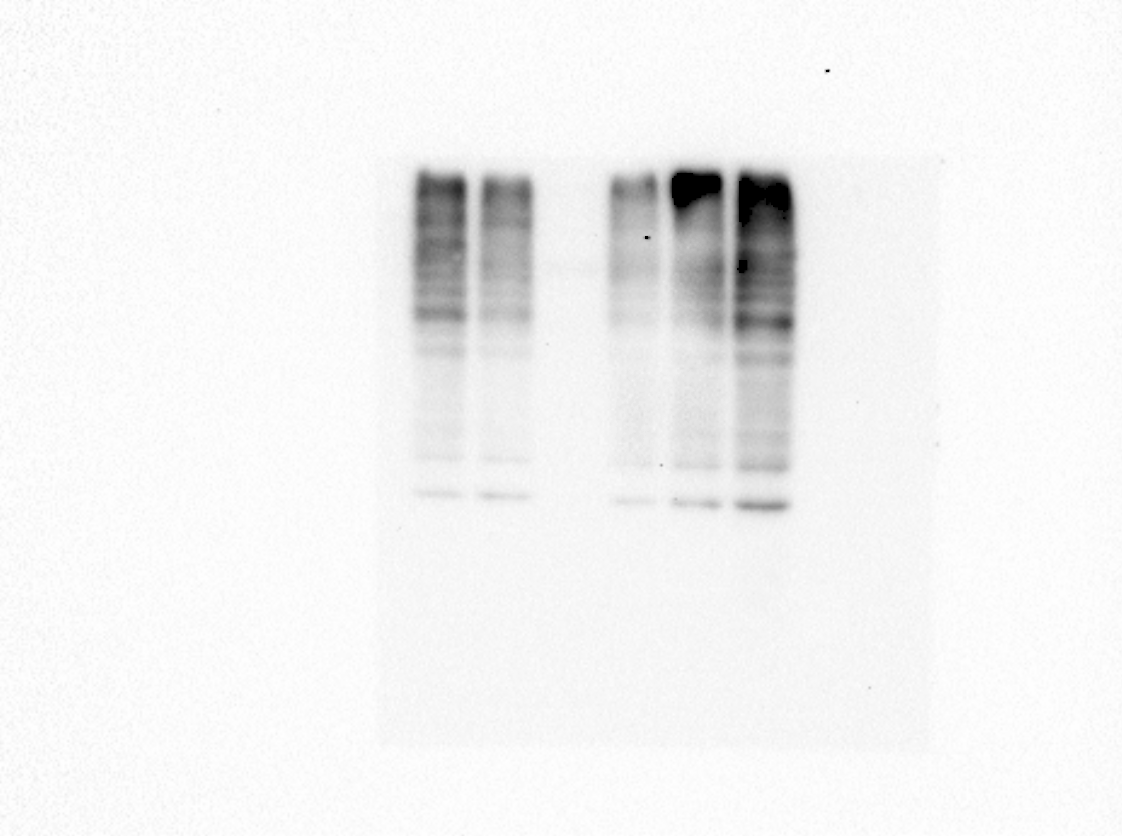

Supplement: Figure 8—source data 1. [file elife-91666-fig8-data1.zip › Figure 8-source data 1/Ub(1)-unedited.tif]

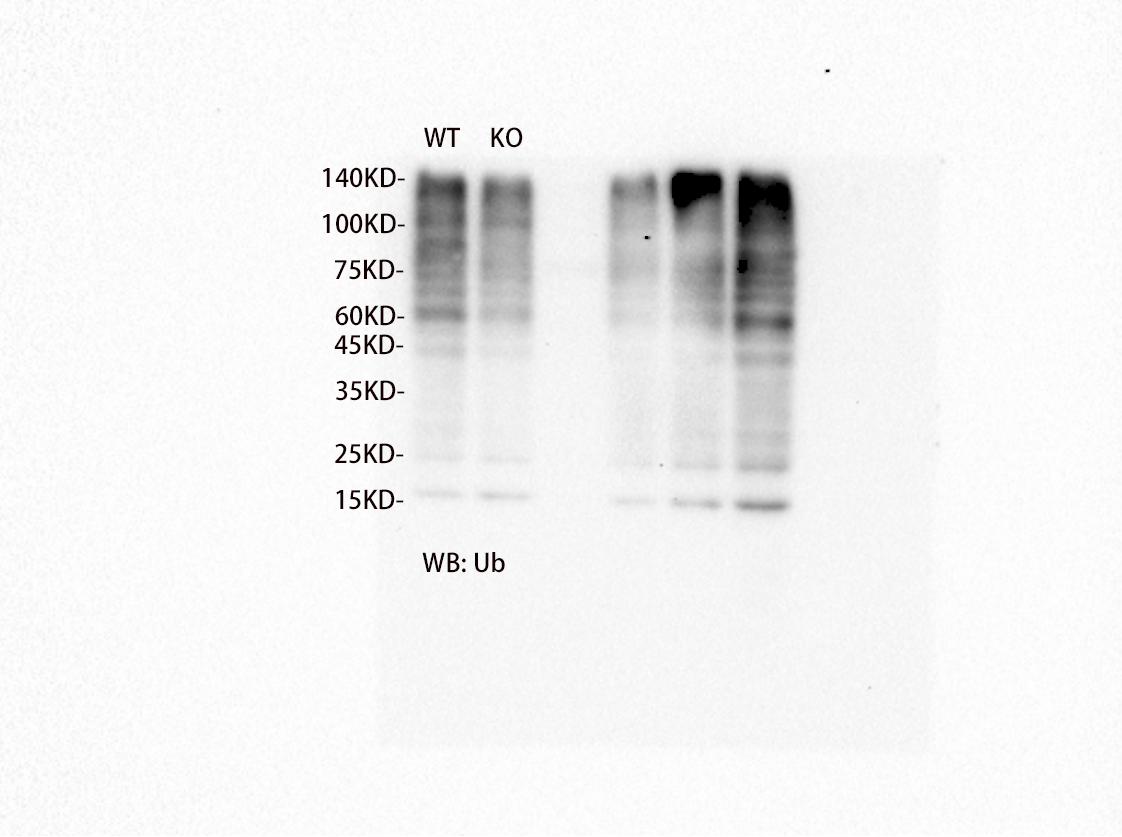

Supplement: Figure 8—source data 1. [file elife-91666-fig8-data1.zip › Figure 8-source data 1/Ub(2)-labelled.tif]

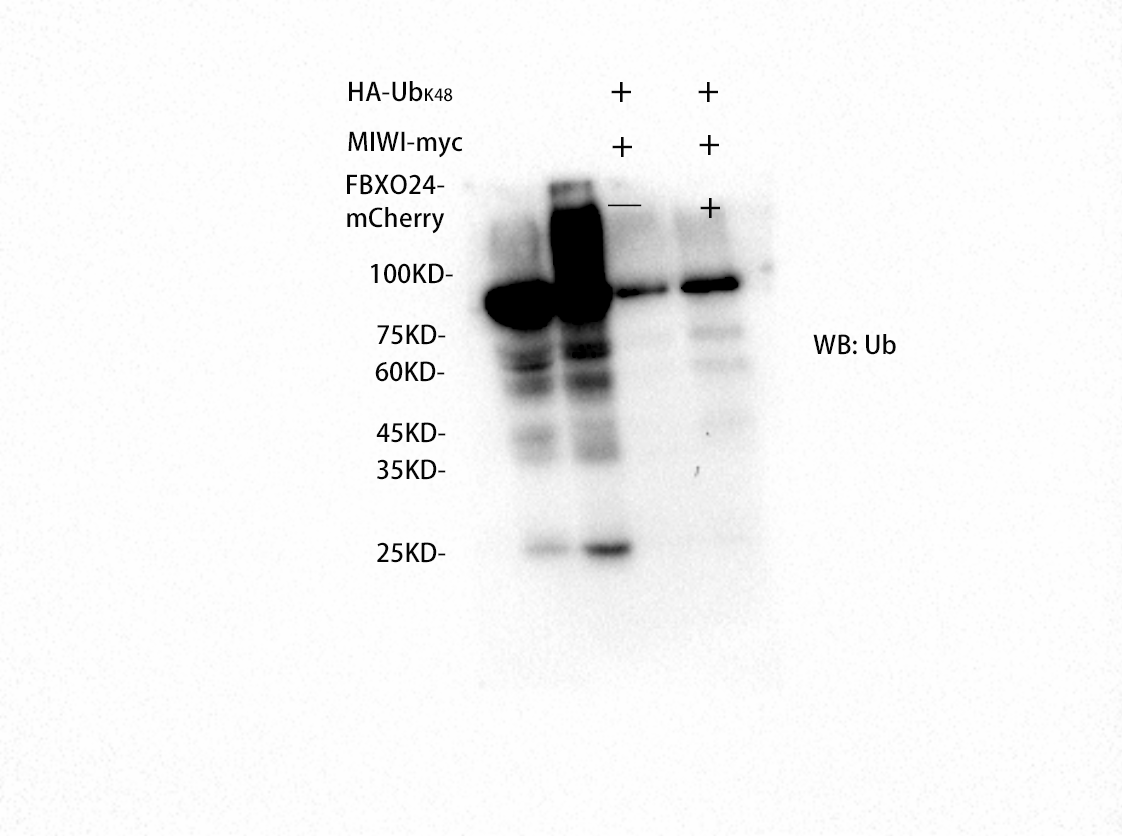

Supplement: Figure 8—source data 1. [file elife-91666-fig8-data1.zip › Figure 8-source data 1/Ub48-labelled.tif]

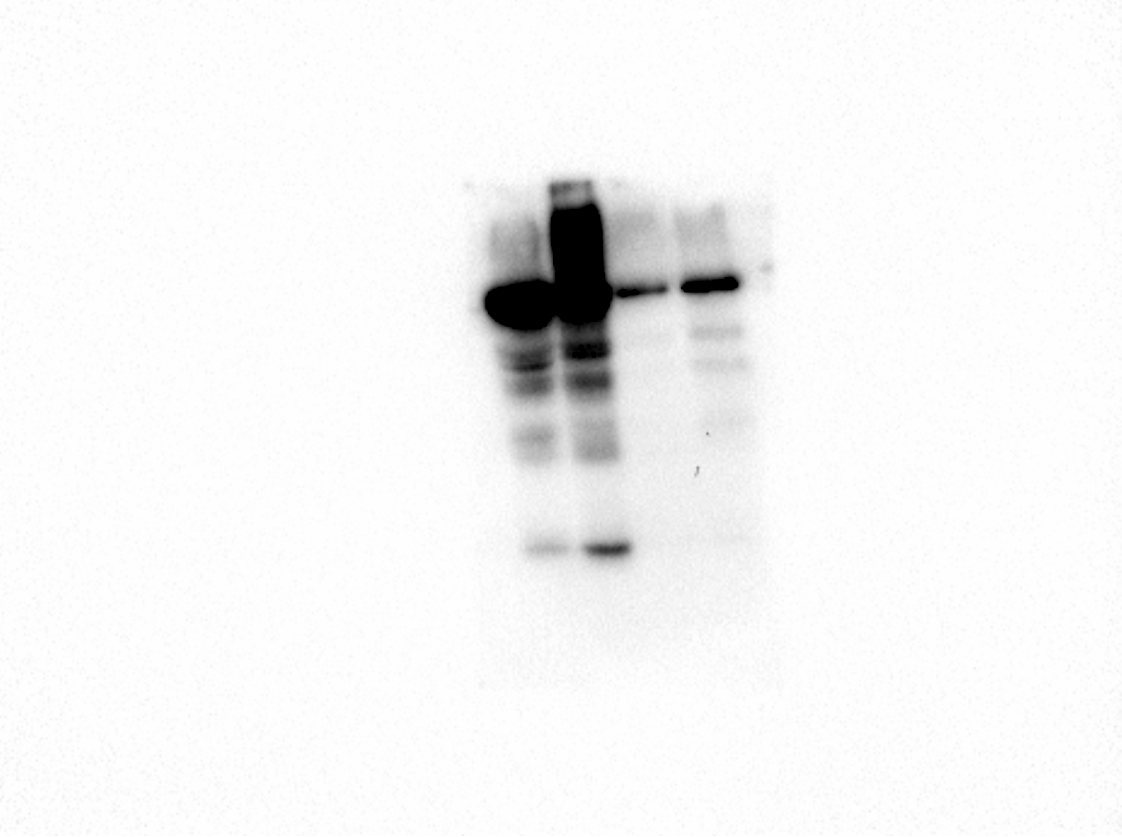

Supplement: Figure 8—source data 1. [file elife-91666-fig8-data1.zip › Figure 8-source data 1/Ub48-unedited.tif]

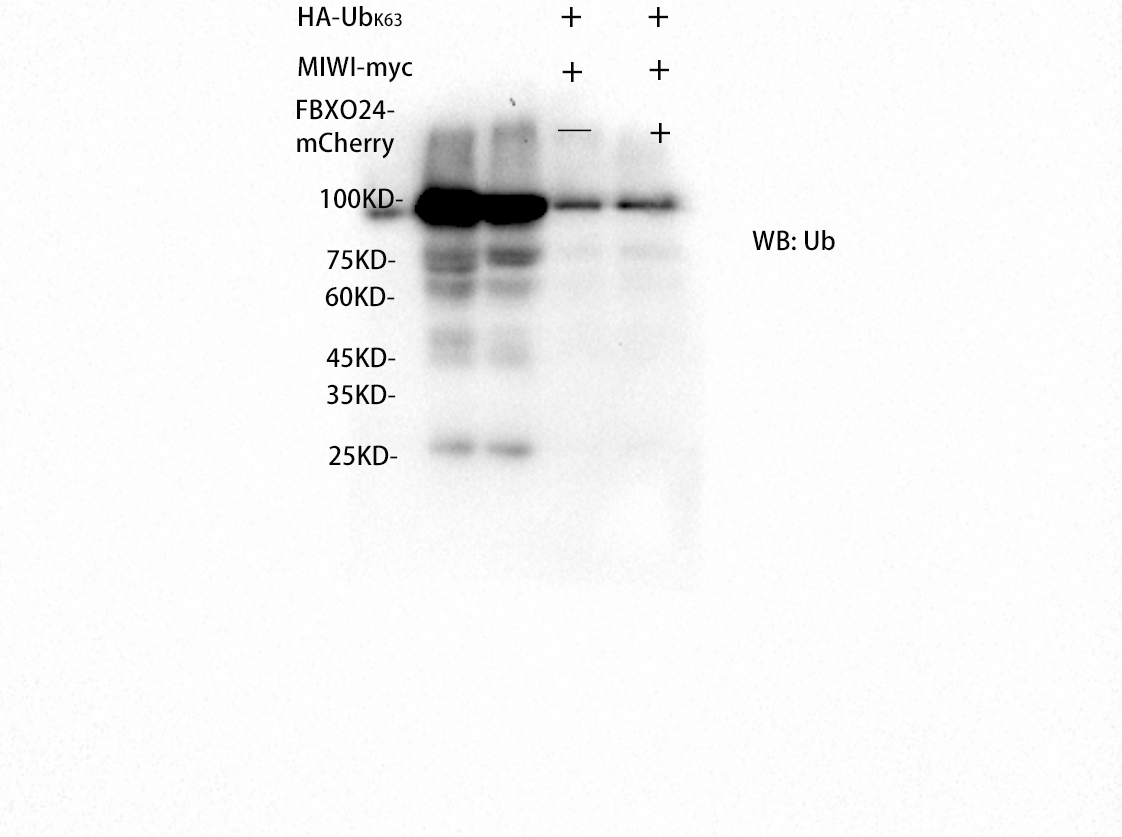

Supplement: Figure 8—source data 1. [file elife-91666-fig8-data1.zip › Figure 8-source data 1/Ub63-labelled.tif]

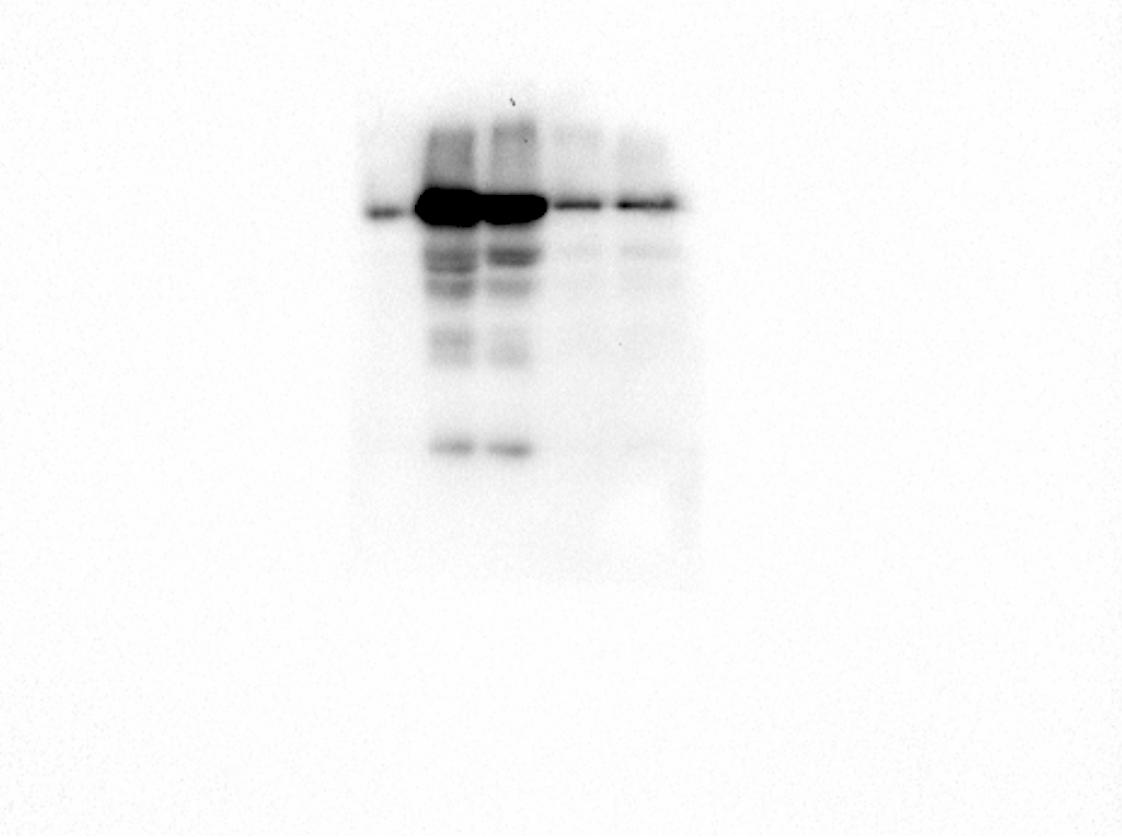

Supplement: Figure 8—source data 1. [file elife-91666-fig8-data1.zip › Figure 8-source data 1/Ub63-unedited.tif]
